# Interim analysis incorporating short‐ and long‐term binary endpoints

**DOI:** 10.1002/bimj.201700281

**Published:** 2019-01-29

**Authors:** Julia Niewczas, Cornelia U. Kunz, Franz König

**Affiliations:** ^1^ Center for Medical Statistics, Informatics, and Intelligent Systems Medical University of Vienna Vienna Austria; ^2^ Department of Mathematics and Statistics Lancaster University Lancaster UK

**Keywords:** adaptive designs, combination test, conditional power, futility stopping, sample size reassessment

## Abstract

Designs incorporating more than one endpoint have become popular in drug development. One of such designs allows for incorporation of short‐term information in an interim analysis if the long‐term primary endpoint has not been yet observed for some of the patients. At first we consider a two‐stage design with binary endpoints allowing for futility stopping only based on conditional power under both fixed and observed effects. Design characteristics of three estimators: using primary long‐term endpoint only, short‐term endpoint only, and combining data from both are compared. For each approach, equivalent cut‐off point values for fixed and observed effect conditional power calculations can be derived resulting in the same overall power. While in trials stopping for futility the type I error rate cannot get inflated (it usually decreases), there is loss of power. In this study, we consider different scenarios, including different thresholds for conditional power, different amount of information available at the interim, different correlations and probabilities of success. We further extend the methods to adaptive designs with unblinded sample size reassessments based on conditional power with inverse normal method as the combination function. Two different futility stopping rules are considered: one based on the conditional power, and one from *P*‐values based on Z‐statistics of the estimators. Average sample size, probability to stop for futility and overall power of the trial are compared and the influence of the choice of weights is investigated.

## 1 INTRODUCTION

The use of interim analyses in clinical trials has become popular in the drug development process. During an interim analysis an ongoing trial can be stopped early for efficacy or futility, or some designs adaptations, such as sample size reassessment or dropping of treatment arms can be performed. Consideration of futility stopping of a trial is seen to be important and useful for both ethical and economic reasons, and therefore widely used (Elsäßer et al., 2014; Hatfield, Allison, Flight, Julious, & Dimairo, 2016; Lin et al., 2016). It is also possible for different endpoints to be considered during an interim analysis and an example of that could be the use of shorter observations on patients. Incorporation of short‐term information into interim analyses of clinical trials has been widely discussed to improve the process of decision making. Different methods have been developed for different types of endpoints. An estimator for binary outcomes that combines short‐ and long‐term data was discussed by Marschner and Becker (2001) with a proposition of extending the topic to group sequential designs or conditional power approaches. Kunz, Wason, and Kieser (2017) applied the estimator to single‐arm phase II oncology trials. Blinded sample size reassessment techniques were discussed by Wüst and Kieser (2005). Whitehead, Sooriyarachchi, Whitehead, and Bolland (2008) compared four methods for incorporating intermediate binary responses into interim analyses for group sequential trials using score and Wald approaches. Similarly methods for continuous data have also been developed by Friede et al. (2011); Kunz, Friede, Parsons, Todd, and Stallard (2015); Stallard (2010); Stallard, Kunz, Todd, Parsons, and Friede (2015) for Phase II/III seamless trials with treatment selection. Hampson and Jennison (2013) discussed use of short‐term data in group sequential tests for delayed responses.

Consider a trial with an interim analysis in which short‐term information on the outcome is incorporated into the analysis when the complete, long‐term observation is not available. Suppose that at the time of interim there are $n_{L}$ patients with complete long‐term observations, *L*. At the same time there are also $n_{S}$ patients that have completed short‐term observations, *S*, which could be a shorter observation time, for example 4 months compared to a year. It is assumed that $n_{S}>n_{L}$ so that there is some additional information available on some patients at the time of interim, that is, *S* but not *L*, is observed. Sometimes the amount of patients with *S* observed can be substantially larger than for *L* and hence once such data is available at interim, it could be beneficial to add it into interim analyses in order to improve decision making and the operating characteristics of the trial design.

We focus on a two‐stage trial with binary endpoints where we consider three estimators: a “classical,” using long‐term data only, one using short‐term observations only, and one, discussed by Marschner and Becker (2001), combining information from both endpoints. The estimators are introduced in Section 2.

Firstly, we consider a design that allows for futility stopping only. The futility stopping has been widely discussed in the literature, see for example DeMets (2006); Jitlal, Khan, Lee, and Hackshaw (2012); Lachin (2005, 2009); Xi, Gallo, and Ohlssen (2017). The analysis is performed using the conditional power approach (Proschan, Lan, & Wittes, 2006) that is discussed in Section 2. The aim is to stop the trial for futility whenever the probability of success at the end of the trial given the results observed thus far is low, and continue otherwise. We discuss two different ways of calculating conditional power as the true effect is unknown: one using the fixed effect (e.g. effect size used when powering the study) and one using the observed effect (estimated effect based on the results observed so far) as a substitute (Bauer & König, 2006). Focus is put on the choice of a threshold for terminating the study based on the conditional power. We show that equivalent cut‐off points resulting in the same stopping rule and therefore overall power can be derived for the two approaches.

In Section 3, we present different simulation scenarios for different effects in the short‐term observations in the experimental treatment group, and then we vary correlation between the endpoints and amount of information available at interim. We also investigate the overall power when the probability of stopping for futility under alternative hypothesis is constant for all estimators.

In Section 4, we further extend the design to unblinded sample size reassessment. Bauer and König (2006) discussed methods for sample size recalculations with use of conditional power arguments and we apply these approaches. As sample size reassessment can inflate type I error we use the combination method in order to maintain α at a prespecified level. There are many existing combination functions and the well‐known examples include Fisher's product (Bauer & Köhne, 1994) and the inverse normal combination function (Lehmacher & Wassmer, 1999). Simulation results are presented in Section 5 that is followed by Discussion in Section 6.

## 2 METHODS

### 2.1 Trial design

Consider a two‐stage trial with binary endpoints and two treatment groups: experimental *E* and control *C*. Let $N_{i}$ (i={E,C}) correspond to the preplanned number of patients in each treatment arm. Let $L_{i}=\left\{0,1\right\}$ denote the outcome in the trial observed on the long‐term primary endpoint, which measures the response for a patient after a prespecified time period $T_{L}$ after randomisation. Define $P_{L_{i}}$ (i={E,C}) to be the probability of a successful outcome at the end of the trial in experimental and treatment groups $\left(Pr\left(L_{i}=1\right)\right)$, and let $M_{L_{i}}$ denote the number of responses in treatment group *i*. Then, an estimate of $P_{L_{i}}$ can be derived from the likelihood function of the Binomial distribution such that ${\hat{P}}_{L_{i}}=M_{L_{i}}/N_{L_{i}}$. We are interested in testing the one sided null hypothesis versus the alternative:

(1) $$H_{0}:\Delta \leq 0\;\text{vs.}\;H_{1}:\Delta >0,$$

at level α with power 1−β, where $\Delta =P_{L_{E}}-P_{L_{C}}$ denotes the treatment difference between the outcomes of *E* and *C*. The final test at the end of the study is carried out using a Z‐statistic with pooled variance compared to $z_{1-\alpha }$ critical value (where $z_{1-\alpha }$ is the (1−α) quantile of the standard normal distribution):

(2) $$Z_{L}=\frac{{\hat{P}}_{L_{E}}-{\hat{P}}_{L_{C}}}{\sqrt{\bar{P_{L}}\left(1-\bar{P_{L}}\right)\left(\frac{1}{N_{E}}+\frac{1}{N_{C}}\right)}},$$

where $\bar{P_{L}}=\frac{{\hat{P}}_{L_{C}}+{\hat{P}}_{L_{E}}}{2}$ and ${\hat{P}}_{L_{i}}$ (i=E,C) denotes the estimate of the outcome as defined above.

Now, suppose that in addition to the long‐term endpoint *L* measured after $T_{L}$, also a short‐term observation, *S* (such that S={0,1} is also a binary variable denoting whether a patient had a response), is observed earlier at a prespecified time $T_{S}$ (with $T_{S}<T_{L}$). At the time of the interim analysis there might be some patients, for whom the short‐term endpoint has already been observed but the long‐term has not. Assume there are $n_{L_{i}}$ patients in each treatment group at interim for the primary endpoint, and $n_{S_{i}}\left(\geq n_{L_{i}}\right)$ patients in each treatment group for the secondary endpoint. There are hence the following possible responses on the patients for sets of *S* and *L* at interim: (S=1,L=1), (S=0,L=1), (S=1,L=0), (S=0,L=0), and additionally (S=1,L=NA) and (S=0,L=NA), when the endpoint *L* is not available (NA). As the amount of patients without the long‐term data may often be substantial, it is of interest to incorporate also the available short‐term data into the analysis. We therefore compare three ways of estimating the response rate at the interim analysis:

- use of long‐term data only, ${\hat{P}}_{L}^{\left(1\right)}$ (based on $n_{L_{E}}+n_{L_{C}}$ patients,
- use of short‐term data only, ${\hat{P}}_{S}^{\left(1\right)}$ (based on $n_{S_{E}}+n_{S_{C}}$ patients),
- or combination of both, ${\hat{P}}_{B}^{\left(1\right)}$ (based on $n_{S_{E}}+n_{S_{C}}$ patients).

Note that the null hypothesis being tested at the end of the trial will always be related to long‐term data only, that is to full observations *L* on the outcome as defined in (1) and using data from the primary endpoint only as defined in (2). Note that ^(1)^ corresponds to the data collected at the time of the interim analysis, where the amount will differ for using just long‐term data, *L*, compared to the latter two approaches (using short‐term outcomes *S* or combining both).

### 2.2 Short‐ and long‐term effect estimates at interim analysis

Let us first consider the long‐term estimator that corresponds to a standard analysis approach. It incorporates information from only complete observations on the primary endpoint. At interim there is information obtained on $n_{L_{i}}$ patients, such that $n_{L_{i}}<N_{i}$ (i={E,C}). Define ${\hat{P}}_{L_{i}}^{\left(1\right)}$ to be the estimate of a successful outcome in treatment arm *i* at the time of interim, and let $m_{L_{i}}$ denote the number of responses at interim. Then, similarly as in (2) but using only patients where $L_{i}$ has been observed, the estimate can be derived from the likelihood function of the Binomial distribution such that ${\hat{P}}_{L_{i}}^{\left(1\right)}=m_{L_{i}}/n_{L_{i}}$. The Z‐statistic of the long‐term interim data is calculated:

(3) $$Z_{L}^{\left(1\right)}=\frac{{\hat{P}}_{L_{E}}^{\left(1\right)}-{\hat{P}}_{L_{C}}^{\left(1\right)}}{\sqrt{{\bar{P}}_{L}^{\left(1\right)}\left(1-{\bar{P}}_{L}^{\left(1\right)}\right)\left(\frac{1}{n_{L_{E}}}+\frac{1}{n_{L_{C}}}\right)}},$$

where ${\bar{P}}_{L}^{\left(1\right)}=\frac{{\hat{P}}_{L_{C}}^{\left(1\right)}+{\hat{P}}_{L_{E}}^{\left(1\right)}}{2}$. Information fraction indicating how far through the trial we are, needed for the interim analysis calculations, can be also easily obtained (see Supplementary Materials Section 1.1). It is the ratio of variances of the estimator at interim and at the end of the trial:

(4) $$t_{L}=\frac{1/N_{E}+1/N_{C}}{1/n_{L_{E}}+1/n_{L_{C}}},$$

and it simplifies to $t_{L}=n_{L}/N$ for equal sample sizes such that $n_{L_{E}}=n_{L_{C}}=n_{L}$ and $N_{E}=N_{C}=N$.

Similar procedures follow when only short‐term observations are used in the interim analysis. Such situation could take place when for example no information on the primary endpoint is available. Let $m_{S_{i}}$ correspond to the number of successes for endpoint *S* and $n_{S_{i}}$ to the number of patients with complete observations on *S* in treatment group *i* (i=E,C). The estimator for short‐term data is obtained in the same way from Binomial distribution such that ${\hat{P}}_{S_{i}}^{\left(1\right)}=m_{S_{i}}/n_{S_{i}}$. However, note that ${\hat{P}}_{S_{i}}^{\left(1\right)}$ is also treated as an estimate of success of $L_{i}$. Similarly, the Z‐statistic and information fraction $t_{S}$ can be obtained for ${\hat{P}}_{S_{i}}^{\left(1\right)}$ in the same way as for ${\hat{P}}_{L_{i}}^{\left(1\right)}$:

(5) $$Z_{S}^{\left(1\right)}=\frac{{\hat{P}}_{S_{E}}^{\left(1\right)}-{\hat{P}}_{S_{C}}^{\left(1\right)}}{\sqrt{{\bar{P}}_{S}^{\left(1\right)}\left(1-{\bar{P}}_{S}^{\left(1\right)}\right)\left(\frac{1}{n_{S_{E}}}+\frac{1}{n_{S_{C}}}\right)}}\;\;t_{S}=\frac{1/N_{E}+1/N_{C}}{1/n_{S_{E}}+1/n_{S_{C}}},$$

such that for equal sample sizes $t_{S}=n_{S}/N$.

Estimator that combines information from both *S* and *L* is derived from three‐binomial distributions (Marschner & Becker, 2001) of $P_{S_{i}}^{\left(1\right)}$, $P_{L_{i}}^{\left(1\right)}$ , and $P_{SL_{i}}^{\left(1\right)}=Pr\left(L_{i}=1|S_{i}=1\right)$. Consider patients for whom *L* has been observed and define $n_{SL_{i}}$ to be the number of patients for whom $S_{i}=1$ and similarly $s_{SL_{i}}$ to be the number of patients for whom $S_{i}=0$. Then, define $m_{SL_{i}}$ and $r_{SL_{i}}$ to be the number of subjects for whom $\left(L_{i}=1,S_{i}=1\right)$ and $(L_{i}=1,S_{i}=0),$ respectively. An estimate of $Pr\left(L_{i}=1\right)={\hat{P}}_{B_{i}}^{\left(1\right)}$ can be derived (Marschner & Becker, 2001):

$${\hat{P}}_{B_{i}}^{\left(1\right)}=\frac{m_{SL_{i}}m_{S_{i}}}{n_{SL_{i}}n_{S_{i}}}+\frac{r_{SL_{i}}\left(n_{S_{i}}-m_{S_{i}}\right)}{s_{SL_{i}}n_{S_{i}}}.$$

In case of ${\hat{P}}_{B}^{\left(1\right)}$, the variance is obtained from the asymptotic distribution of the likelihood function (see Marschner and Becker (2001)) and it can be simplified to the following form:

$$\text{Var}\left({\hat{P}}_{B_{i}}^{\left(1\right)}\right)=\frac{1}{n_{L_{i}}}{\hat{P}}_{B_{i}}^{\left(1\right)}\left(1-{\hat{P}}_{B_{i}}^{\left(1\right)}\right)\left(1-{\hat{\phi_{i}}}^{2}\times \left(1-\frac{n_{L_{i}}}{n_{S_{i}}}\right)\right),$$

where $\hat{\phi_{i}}$ is the estimate of the correlation (Phi coefficient) between *S* and *L* (defined by Cramér (1946)):

$$\hat{\phi_{i}}=\frac{{\hat{P}}_{SL_{i}}^{\left(1\right)}{\hat{P}}_{S_{i}}^{\left(1\right)}-{\hat{P}}_{B_{i}}^{\left(1\right)}{\hat{P}}_{S_{i}}^{\left(1\right)}}{\sqrt{{\hat{P}}_{B_{i}}^{\left(1\right)}\left(1-{\hat{P}}_{B_{i}}^{\left(1\right)}\right){\hat{P}}_{S_{i}}^{\left(1\right)}\left(1-{\hat{P}}_{S_{i}}^{\left(1\right)}\right)}}.$$

Again, the Z‐statistic is obtained:

$$Z_{B}^{\left(1\right)}=\frac{{\hat{P}}_{B_{E}}^{\left(1\right)}-{\hat{P}}_{B_{C}}^{\left(1\right)}}{\sqrt{{\bar{P}}_{B}^{\left(1\right)}\left(1-{\bar{P}}_{B}^{\left(1\right)}\right)\left(\frac{1-{\hat{\phi_{E}}}^{2}\times \left(1-\frac{n_{L_{E}}}{n_{S_{E}}}\right)}{n_{L_{E}}}+\frac{1-{\hat{\phi_{C}}}^{2}\times \left(1-\frac{n_{L_{C}}}{n_{S_{C}}}\right)}{n_{L_{C}}}\right)}},$$

where ${\bar{P}}_{B}^{\left(1\right)}=\frac{{\hat{P}}_{B_{C}}^{\left(1\right)}+{\hat{P}}_{B_{E}}^{\left(1\right)}}{2}$, $\hat{\phi_{i}}$ is the estimate of the correlation between *S* and *L* and $\text{Var}({\hat{P}}_{B_{i}}^{\left(1\right)})$ is the variance obtained from the asymptotic distribution of the likelihood function (see Marschner and Becker (2001)). The information fraction is obtained from the ratio of variances, so it is therefore dependent on the correlation between *S* and *L*. It can be simplified to the following form (see Supplementary Materials Section 1.4 for derivation):

(6) $${\hat{t}}_{B}=\frac{\frac{1}{N_{C}}+\frac{1}{N_{E}}}{\frac{1-{\hat{\phi }}_{E}^{2}\left(1-\frac{n_{L_{E}}}{n_{S_{E}}}\right)}{n_{L_{E}}}+\frac{1-{\hat{\phi }}_{C}^{2}\left(1-\frac{n_{L_{C}}}{n_{S_{C}}}\right)}{n_{L_{C}}}},$$

where ${\hat{\phi }}_{E}^{2},{\hat{\phi }}_{C}^{2}$ are the estimated correlations between *S* and *L* in each treatment group. It simplifies to $t_{L}$ when $\phi_{E,C}=0$ and to $t_{S}$ when $\phi_{E,C}=1$.

In some cases, the estimator is however not defined. This happens when either $n_{SL_{i}}$ or $s_{SL_{i}}$ are equal to 0. In such a case both the estimator and variance are obtained using the long‐term endpoint only: ${\hat{P}}_{B_{i}}^{\left(1\right)}=\frac{m_{L_{i}}}{n_{L_{i}}}$ and $\text{Var}\left({\hat{P}}_{B_{i}}^{\left(1\right)}\right)=\frac{{\hat{P}}_{B_{i}}^{\left(1\right)}\left(1-{\hat{P}}_{B_{i}}^{\left(1\right)}\right)}{n_{L_{i}}}$.

### 2.3 Futility stopping based on conditional power arguments

The interim futility stopping rule is based on the conditional power (CP), that is “conditional probability that the final result will exceed a critical value given the data observed thus far” (Proschan et al., 2006). This means that once we have interim data available, we want to calculate the chance to have a successful outcome at the end of the trial conditional on the observed effects at the interim. If such a probability is below some threshold *c*, we stop the trial for futility. Otherwise the trial is continued until all the information on patients has been gathered.

It can be calculated using the Brownian motion structure and the B‐value that is a combination of the Z‐statistic and the fraction of information: $B\left(t\right)=Z\left(t\right)\sqrt{t},\;0<t\leq 1$. CP is then obtained from conditioning on $B\left(1\right)>z_{\alpha }$ (where $z_{\alpha }$ is the α quantile of the standard normal distribution) given that B(t) is equal to some *b* at time *t* (see Proschan et al. (2006) for derivation):

$$\text{CP}\left(t\right)=1-\Phi \left(\frac{z_{1-\alpha }-\text{E}\left\{B\left(1\right)|B\left(t\right)=b\right\}}{\sqrt{1-t}}\right),$$

where $z_{1-\alpha }$ is the (1−α) quantile of the standard normal distribution. However, CP should be calculated using the true treatment effect that is unknown. Therefore we use two approaches for its estimation (Bauer & König, 2006). The first one assumes the true effect to be equal to the effect size used when powering the study (fixed effect or alternative hypothesis conditional power). The conditional probability under the fixed effect is hence equal to (the derivation can be found in Supplementary Materials Section 1.5):

(7) $$CP_{\theta_{D}}\left(t_{i}\right)=1-\Phi \left(\frac{z_{1-\alpha }-\sqrt{t_{i}}Z_{i}^{\left(1\right)}}{\sqrt{1-t_{i}}}-\left(z_{1-\alpha }+z_{1-\beta }\right)\sqrt{1-t_{i}}\right),$$

where i={L,S,B} corresponds to a given estimator.

The second approach uses the effect size from data observed thus far (observed effect or current trend) and the conditional power is in such a case equal to:

$$CP_{\hat{\theta }}\left(t_{i}\right)=1-\Phi \left(\frac{z_{1-\alpha }-Z_{i}^{\left(1\right)}/\sqrt{t_{i}}}{\sqrt{1-t_{i}}}\right).$$

The Z‐statistics and information fractions defined in the previous section are then be plugged into the conditional power formulas. If CP<c, the trial is stopped for futility, which formally means that we have to retain the null hypothesis, as defined in (1). If CP>c, we proceed with the trial and after all long‐term data on $N_{i}$ patients is observed, the null hypothesis is tested by (2).

## 3 SIMULATION SETTINGS

Consider a two‐stage trial with two parallel treatment groups with $N_{i}=200$ patients per treatment arm, that is with equal patient allocation ratio. We wish to claim efficacy in the experimental arm while controlling the type I error rate at level α=0.025 (one‐sided) with power of 1−β=0.8. The outcome of interest is a binary response. At the interim analysis long‐ and short‐term observations are available on patients. We assume that 25% of the patients have complete observations on the long‐term outcome such that $t_{L}=0.25$ and 50% of short‐term observations are available, that is $t_{S}=0.5$. The response rate for both the long‐ and short‐term outcomes in the control group was set to be equal such that $P_{L_{C}}=P_{S_{C}}=0.2$. From $P_{L_{C}}$ and $N_{i}=200$ (assuming power of 80%, one‐sided α=0.025 and equal sample size allocation ratio), the required response rate in the experimental arm was obtained with $P_{L_{E}}\approx 0.323$. We considered four types of outcomes for the short‐term outcome in the experimental treatment group: no effect, moderate effect, effect equal to long‐term outcome, and larger effect than for the long‐term outcome with the following probabilities: (0.2,0.285,0.323,0.365). These effects would correspond to 2.5%, 50%, 80%, and 95% power, if a χ^2^ test was performed for a one stage trial testing the hypothesis *H*_0_ defined in (1). The correlation between long‐ and short‐outcomes was fixed to be the same in both treatment groups and equal to $\phi_{E}=\phi_{C}=0.5$. We ran 100,000 simulations in R under all scenarios discussed in further sections.

In the Supplementary Materials Section 2.2 further correlations were investigated (namely $\phi_{C}=\phi_{E}=\left(0.2,0.65\right)$). We also looked at a scenario with “nested'' outcomes in which it was assumed $Pr(L_{i}=1|S_{i}=0)=0$, so that if there is no successful outcome for the short‐term endpoint, there would be no successful outcome for the long‐term one. In such a scenario, the correlation between *S* and *L* is induced by design. Results in such a setting can be found in Supplementary Materials in Section 2.3. Note that under such scenarios the correlation between $S_{C}$ and $L_{C}$, as well as $S_{E}$ and $L_{E}$ changes for each setting. The R program is available as Supplementary Material.

### 3.1 Simulation results

**Figure 1:**
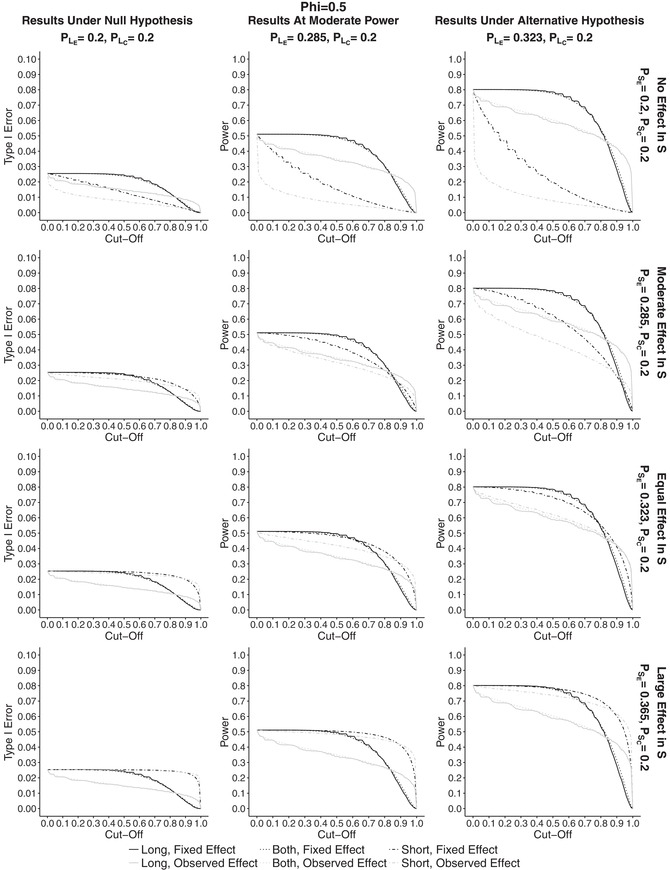
Plots showing the power plotted against cut‐off points for different effect sizes in $P_{S_{E}}$ for $\phi_{E}=\phi_{C}=0.5$, assuming the effects in the control group to be equal to $P_{L_{C}}=P_{S_{C}}=0.2$. First column corresponds to the results under the null hypothesis, middle column to the simulations at power of 50% and right to the simulations under alternative hypothesis for $P_{L_{E}}$. The rows correspond to increasing effects in $P_{S_{E}}$, that is to no effect, moderate effect, effect equal to the one of $P_{L_{E}}$ under the alternative hypothesis, and a higher effect than for $P_{L_{E}}$ respectively. Gray lines correspond to observed effect conditional power, $CP_{\hat{\theta }}$, whereas black to fixed effect conditional power, $CP_{\theta_{D}}$. ${\hat{P}}_{B}^{\left(1\right)}$ is denoted by dotted lines, ${\hat{P}}_{L}^{\left(1\right)}$ by solid and ${\hat{P}}_{S}^{\left(1\right)}$ by dot‐dashed

**Figure 2:**
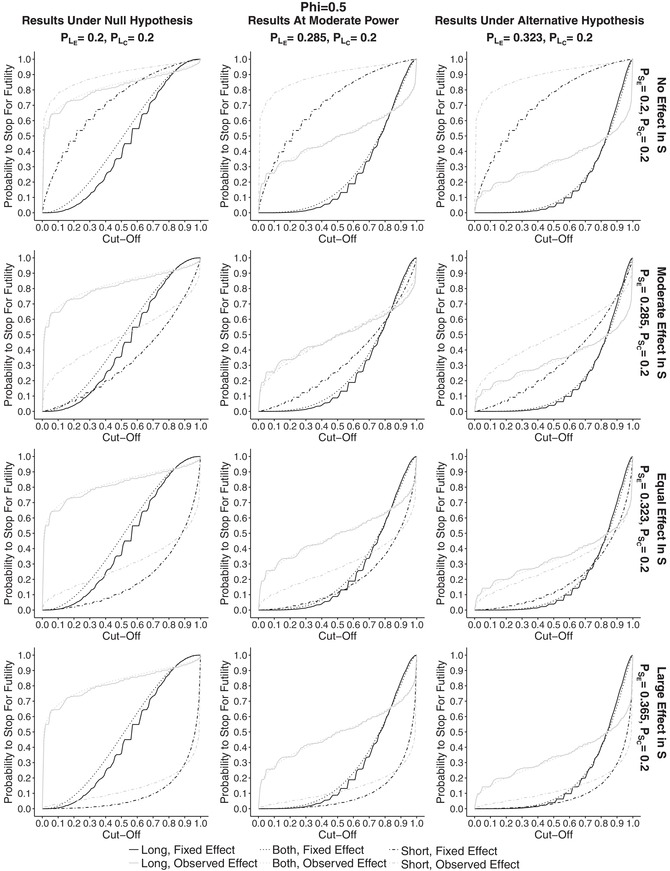
Plots showing the probability to stop for futility plotted against cut‐off points for different effect sizes in $P_{S_{E}}$ for $\phi_{E}=\phi_{C}=0.5$, assuming the effects in the control group to be equal to $P_{L_{C}}=P_{S_{C}}=0.2$. First column corresponds to the results under the null hypothesis, middle column to the simulations at power of 50% and right to the simulations under alternative hypothesis for $P_{L_{E}}$. The rows correspond to increasing effects in $P_{S_{E}}$, that is to no effect, moderate effect, effect equal to the one of $P_{L_{E}}$ under the alternative hypothesis, and a higher effect than for $P_{L_{E}}$ respectively. Gray lines correspond to observed effect conditional power, $CP_{\hat{\theta }}$, whereas black to fixed effect conditional power, $CP_{\theta_{D}}$. ${\hat{P}}_{B}^{\left(1\right)}$ is denoted by dotted lines, ${\hat{P}}_{L}^{\left(1\right)}$ by solid and ${\hat{P}}_{S}^{\left(1\right)}$ by dot‐dashed

Altogether 12 scenarios were considered: for the effects for the long‐term outcome under the null hypothesis, for moderate results (at power of 50%) and under the alternative hypothesis. At the same time we varied the success probabilities for $P_{S_{E}}$ so that they were equal to (0.2,0.285,0.323,0.365). We were interested in the impact of the cut‐off point on the overall power of the trial as well as the probability to stop for futility (futility stopping, FS). We also reported probabilities conditional on the interim decision, that is probability of rejecting the null hypothesis given the trial was continued; if the trial was stopped, the probability of not having rejected the null hypothesis, if the trial had been continued; and the probability of making the correct decision (i.e. stopping the trial if there was no rejection or continuation and being able to reject *H*_0_ at the end of the trial). The results can be found in Supplementary Materials in Section 2.1. The results for the overall power plotted against cut‐off points are shown in Figure 1 and those for the probability to stop for futility in Figure 2. In both, the first column corresponds to the results under the null hypothesis, the second one to the results at moderate power and the last one to the results under the alternative hypothesis. Note that the scale under the null hypothesis scenario is plotted from 0 to 0.1. The rows correspond to different probabilities of success for the short‐term outcome in the experimental treatment group. Black lines on the plots correspond to fixed effect conditional power ($CP_{\theta_{D}}$), whereas gray lines correspond to observed effect ($CP_{\hat{\theta }}$). Solid curves represent the results for the estimator using long‐term data only, ${\hat{P}}_{L}^{\left(1\right)}$, dot‐dashed curves correspond to the results for the estimator using short‐term data only, ${\hat{P}}_{S}^{\left(1\right)}$, and finally dotted lines to the estimator combining both endpoints, ${\hat{P}}_{B}^{\left(1\right)}$.

The overall power is decreasing for all approaches with increasing cut‐off points and the fixed design conditional power outperforms the observed effect approach up to cut‐off point c=0.8 under all scenarios. It can be seen that for both approaches incorporating long‐term data for the fixed effect CP, the overall power under the alternative hypothesis is still around the design power up to cut‐off points of around 0.5. This is also the case for the estimator using short‐term data only when the effect in *S* is at least as high as the one in *L*. For the observed effect CP, we can see a large drop in power for low values of *c*, with a drop of around at least 10% at c=0.1 under all scenarios (with the exception a large effect in the short‐term outcome and ${\hat{P}}_{S}^{\left(1\right)}$). If there is a small or no effect in *S* , then there is a severe loss of power due to stopping too easily for futility.

At the first glance the estimator combining short‐ and long‐term data has more or less the same power results as the long‐term one. It is quite robust and does not get heavily influenced by the effect in short‐term outcome. The estimator using short‐term data is dependent on $P_{S_{E}}$ only so that if there is no effect in the short‐term outcome, there is a large drop in power. When $P_{S_{E}}=0.365$, that is the effect in the short‐term outcome is larger than for the long‐term, the estimator has the highest power, equal to the design one of 80% (or slightly lower) for all cut‐off points. However, this is only due to the fact that the conditional power using only short‐term information is being overestimated, which results in the futility boundary being crossed too easily and hence almost never stopping the trial. For the observed effect approach, ${\hat{P}}_{B}^{\left(1\right)}$ has higher power than ${\hat{P}}_{L}^{\left(1\right)}$ for all cut‐off points and all scenarios for the effect in *S*. For high values of $P_{S_{E}}$ the estimator using short‐term data only has the highest results. Similar trends can be seen at moderate power for both conditional power approaches, but the results are simply equivalently lower, with the highest power of 50%.

In Figure 3 in Supplementary Materials in Section 2.1 the probability to make the correct decision at interim is plotted for all discussed scenarios. Under the alternative hypothesis ${\hat{P}}_{L}^{\left(1\right)}$ and ${\hat{P}}_{B}^{\left(1\right)}$ have similar results for all effects in *S*. The probability is equal to at least 80% up to cut‐off points of around 0.65 under the fixed effect. At moderate power the estimator using both *S* and *L* has a higher probability than ${\hat{P}}_{L}^{\left(1\right)}$. Both achieve the peak for c=0.75 that results in probability of 65–67%. The probability is highest for the observed effect approach for almost all cut‐off points. Under the null hypothesis, the probability increased with cut‐off points and is highest for $CP_{\hat{\theta }}$.

For the probability to stop for futility (FS), we can see similar patterns as for the overall power plotted against cut‐off points but they go in the opposite direction, that is probability to stop for futility is increasing with the increase of the cut‐off point values. Under the null hypothesis FS is highest for the observed effect approach, and when there is no effect in *S* the probability is at least 60% for a cut‐off point of 0.1 The highest probability is obtained by using ${\hat{P}}_{S}^{\left(1\right)}$. Similar patterns can be seen for other probabilities of $P_{S_{E}}$ for ${\hat{P}}_{L}^{\left(1\right)}$ and ${\hat{P}}_{B}^{\left(1\right)}$ . The estimator using only short‐term information has, however, a decreasing probability to stop for futility with an increasing effect in $P_{S_{E}}$. Under the fixed effect approach the values are much lower and do not change much depending on $P_{S_{E}}$ for ${\hat{P}}_{B}^{\left(1\right)}$.

The results at moderate power and under alternative hypothesis are similar for both conditional power approaches for all estimators but FS is slightly higher at the moderate power. Again, for ${\hat{P}}_{S}^{\left(1\right)}$ the probability to stop for futility varies a lot with value of $P_{S_{E}}$.

We also looked at the overall power for different correlation structures between *L* and *S*, namely $\phi_{E}=\phi_{C}=\left\{0.2,0.65\right\}$ in order to further investigate the behavior of the estimators. Plots can be found in Supplementary Materials. What was found is that, the lower the correlation between *L* and *S*, the lower is the power for the estimators incorporating information from short‐term outcomes, and again, the higher the correlation, the higher the overall power. The increase in power is higher for the observed effect conditional power approach. Thus, in order to benefit from incorporation of *S*, the effect sizes need to be similar or with a high correlation between *S* and *L*.

### 3.2 Equivalence of cut‐off points

Choice of a threshold for CP is an important factor when designing a study. If the cut‐off point is chosen to be too high then the trial will be stopped too frequently resulting in loss of power and what comes with it losing the opportunity of claiming efficacy on an effective drug. Similarly, if *c* is chosen to be too low, the trial will be stopped too rarely posing risk at patients. What is more, the choice of the same cut‐off point for both conditional power approaches, fixed and observed effect, results in higher power for the fixed effect. This happens because the fixed effect assumes a more optimistic scenario, that is, regardless of the first stage data, the second stage data is assumed to have the effect size under the alternative hypothesis. On the other hand, the observed effect approach uses the assumption that the second stage data will have the effect size equal to the interim one that is lower as the sample size for which the Z‐statistic is obtained, corresponds only to a fraction of patients. However, it is possible to find equivalent cut‐off points for the two approaches that will result in the same overall power. This is done by rearranging the conditional power equations for the Z‐statistic, which is the same for both approaches and then solving for either of the cut‐off points. The equivalent cut‐off point of the observed effect for the fixed is hence as following (the derivation can be found in Supplementary Materials in Section 1.6):

$$c_{\theta_{D}}=1-\Phi \left(\frac{z_{1-\alpha }-\left(z_{1-\alpha }+z_{1-\beta }\right)\left(1-t\right)-\left(z_{1-\alpha }-\Phi^{-1}\left(1-c_{\hat{\theta }}\right)\sqrt{1-t}\right)t}{\sqrt{1-t}}\right),$$

where $c_{\theta_{D}}$ is the cut‐off point for the fixed effect conditional power and $c_{\hat{\theta }}$ is the cut‐off point for the observed effect conditional power. Similarly the equivalent cut‐off point for the observed effect is:

$$c_{\hat{\theta }}=1-\Phi \left(\frac{z_{1-\alpha }}{\sqrt{1-t}}-\frac{z_{1-\alpha }-\Phi^{-1}\left(1-c_{\theta_{D}}\right)\sqrt{1-t}-\left(z_{1-\alpha }+z_{1-\beta }\right)\left(1-t\right)}{t\sqrt{1-t}}\right).$$

**Figure 3:**
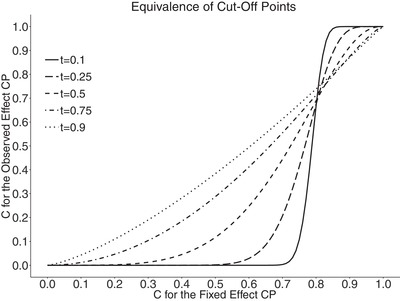
Figure showing the equivalence of cut‐off points in terms of achieving the same stopping rule and overall power for two proposals of calculating conditional power: using observed effect (y‐axis) and fixed effect (x‐axis) for different information fraction *t*

This can be applied to all estimators. Figure 3 below shows examples of equivalent thresholds at different times of interim analysis, t=(0.1,0.25,0.5,0.75,0.9) for α=0.025, 1−β=0.8. From the plot it can be seen that the higher *t*, the more linear is the relationship between fixed and observed effects. At t=0.9 it is almost linear. The reason for that is that the rest of the data cannot have a large impact on the final decision and for both approaches the conditional power values should be close to either 0 or 1. We can also see that when *c* for fixed effect ($c_{\theta_{D}}$) is around the design power or higher, the equivalent threshold for observed effect ($c_{\hat{\theta }}$) is actually higher. For $c_{\hat{\theta }}$ at t=0.5, the equivalent values are almost 0 up to $c_{\theta_{D}}$ of around 0.25. This is even stricter at t=0.1 where the equivalent $c_{\hat{\theta }}$ is close to 0 up to $c_{\theta_{D}}\approx 0.7$. Therefore, it is important to bear in mind that if we choose for example $c_{\theta_{D}}=0.2$ at time t=0.5, we will have to choose a much lower $c_{\hat{\theta }}$ in order to achieve the same power.

### 3.3 Varying correlation and information fraction

As we have shown equivalent cut‐off points can be easily found for the fixed and observed effect conditional power approaches, we will only consider the fixed effect, $CP_{\theta_{D}}$ in the remainder of this section. The choice of *c* influences not only the overall power of the trial but also the probability to stop for futility. It can be seen in Figures 1 and 2 that the choice of the same *c* for the three estimators results in different values for both the power and probability to stop for futility (futility stopping, FS). For this reason, we decided to look at the power results, where the probability to stop for futility is equal for all estimators.

Two scenarios were considered: with $t_{L}=0.25$ and $t_{S}=0.5$ and with a larger difference between the amount of data available at interim, namely $t_{L}=0.25$ and $t_{S}=0.75$. The correlation between *S* and *L* was also investigated and for this reason the probabilities of success for *S* and *L* were equalized in both treatment groups: $P_{L_{C}}=P_{S_{C}}=0.2$ and $P_{L_{E}}=P_{S_{E}}=0.323$. The following correlations were considered: $\phi_{i}=\left(0,0.2,0.5,0.7,0.9\right)$ (i=E,C). For simplicity of comparison the correlations were always equal for both treatment groups. The probability to stop for futility was set to 10% under the alternative hypothesis. In order to obtain a cut‐off point that results in such a probability, we simulated data 100,000 times for a range of cut‐off points from 0 to 1. We then searched for values of *c* at which FS was equal to 10% and then looked at the corresponding power. For ${\hat{P}}_{L}^{\left(1\right)}$ such a *c* is equal to 0.61 under both simulation scenarios (as ${\hat{P}}_{L}^{\left(1\right)}$ is independent of *S* and hence has a constant power under both scenarios and for all correlations). ${\hat{P}}_{S}^{\left(1\right)}$ has the probability to stop for futility of 10% for c=0.46 when $t_{S}=0.5$ and for c=0.31 when $t_{S}=0.75$. We can see that the higher the information fraction for *S*, the lower the cut‐off point that results in FS of 10%. As the information fraction of ${\hat{P}}_{B}^{\left(1\right)}$ is dependent on the correlation between *S* and *L*, cut‐off points resulting in FS of 10% vary with the correlation. So under the first scenario with $t_{L}=0.25$ and $t_{S}=0.5$, the cut‐off points were found to be (0.59,0.59,0.57,0.54,0.51) for the correlations (0,0.2,0.5,0.7,0.9), and under the second scenario with $t_{L}=0.25$ and $t_{S}=0.75$ the cut‐off points were (0.59,0.58,0.56,0.52,0.43). It can be seen, that the higher the correlation and the higher the amount of short‐term outcomes available at interim, the lower is cut‐off point resulting in probability to stop for futility of 10%.

**Figure 4:**
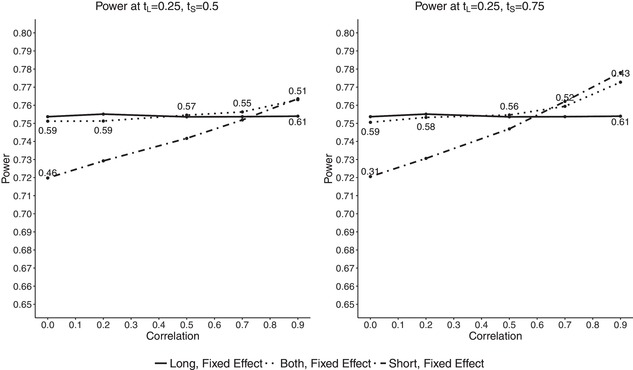
Overall power plotted against correlations for fixed effect conditional power at probability to stop for futility of 10% with $P_{LE}=P_{SE}=0.323$ for a range of correlations from $\Phi_{E}=\Phi_{C}=0$ to 0.9. The cut‐off points at which the probability occurs are denoted by the numbers above the lines. Left plot shows results at $t_{L}=0.25$ and $t_{S}=0.5$ and right plot at $t_{L}=0.25$ and $t_{S}=0.75$. ${\hat{P}}_{B}^{\left(1\right)}$ is denoted by dotted lines, ${\hat{P}}_{L}^{\left(1\right)}$ by solid and ${\hat{P}}_{S}^{\left(1\right)}$ by dot‐dashed

Figure 4 shows the overall power achieved at given cut‐off points for the estimators using $CP_{\theta_{D}}$. The left plot shows the results at $t_{L}=0.25,t_{S}=0.5$ and the right one at $t_{L}=0.25,t_{S}=0.75$. Results for ${\hat{P}}_{L}^{\left(1\right)}$ are plotted as solid lines, for ${\hat{P}}_{S}^{\left(1\right)}$ as dot‐dashed and for ${\hat{P}}_{B}^{\left(1\right)}$ as dotted. The numbers on the plots correspond to the cut‐off points resulting in the same FS. As discussed above, the results for ${\hat{P}}_{L}^{\left(1\right)}$ are constant under both scenarios and for all correlations. The differences in the plots are simply due to simulation error. It can be seen that the overall power increases with correlation for both approaches incorporating short‐term outcomes. When the correlation is equal to 0, the estimator using short‐term data only has a lower power than the other two of around 3% points. When the correlation is very high, ${\hat{P}}_{S}^{\left(1\right)}$ has overall power that is slightly larger than that of ${\hat{P}}_{L}^{\left(1\right)}$, equal to round 75.7%. For low correlations ${\hat{P}}_{B}^{\left(1\right)}$ has power that is slightly lower than ${\hat{P}}_{L}^{\left(1\right)}$ but the drop is not higher than 0.2% points. When the correlation increases to 0.5, it outperforms ${\hat{P}}_{L}^{\left(1\right)}$, and for ϕ=0.9 it has the highest overall power among all estimators, equal to round 76%.

When the difference between $t_{L}$ and $t_{S}$ increases we can see a larger increase in power for the estimators incorporating short‐term data. For low to intermediate correlations, we can see similar results as for $t_{S}=0.5$ but when the correlation between *S* and *L* increases, ${\hat{P}}_{S}^{\left(1\right)}$ has a much larger increase in power, and for ϕ=0.9 it has the highest power of above 77%. The overall power of ${\hat{P}}_{B}^{\left(1\right)}$ increases slightly for ϕ=0.9 compared to $t_{S}=0.5$. It can be seen that if the correlation between *S* and *L* is high, we can gain power by using ${\hat{P}}_{B}^{\left(1\right)}$ while using a lower cut‐off point value, and in a situation when there is no correlation between short‐ and long‐term data, ${\hat{P}}_{B}^{\left(1\right)}$ still achieves high values.

**Figure 5:**
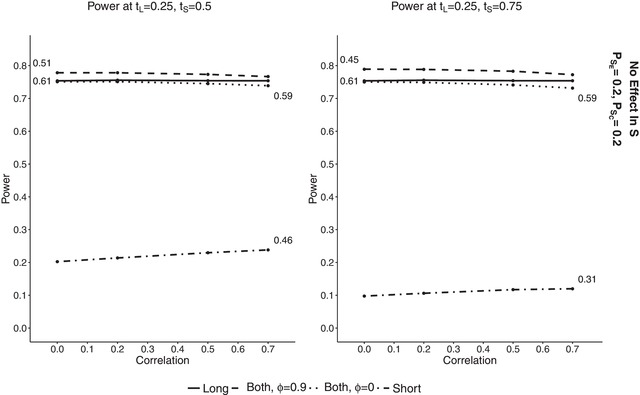
Overall power plotted against correlations for fixed effect conditional power for different correlations when there is no effect for the short‐term outcome, $P_{S_{E}}=0.2$ and with $P_{L_{E}}=0.323$. The cut‐off points at which the probability occurs are denoted by the numbers above the lines. Left plot shows results at $t_{L}=0.25$ and $t_{S}=0.5$ and right plot at $t_{L}=0.25$ and $t_{S}=0.75$. ${\hat{P}}_{B}^{\left(1\right)}$ is denoted by dotted lines, ${\hat{P}}_{L}^{\left(1\right)}$ by solid and ${\hat{P}}_{S}^{\left(1\right)}$ by dot‐dashed

However, as the correlation between *S* and *L* might be unknown and it should be prespecified for the planning stage, it would be of interest to see what happens when the cut‐off point for ${\hat{P}}_{B}^{\left(1\right)}$ is chosen for the wrong correlation or what happens if the effect for the short‐term outcome is either much larger or smaller than for the long‐term one. In Figure 5 and in the Supplementary Materials in Section 2.4, we can see how changing the probability of success for the short‐term endpoint *S* influences the overall power of the trial. In this scenario, we decided to have a look at the overall power, when our cut‐off point values for ${\hat{P}}_{S}^{\left(1\right)}$ and ${\hat{P}}_{B}^{\left(1\right)}$ were chosen assuming equal probabilities of success for both, short‐ and long‐term endpoints, either assuming no correlation between the endpoints or a high one (i.e. $P_{S_{E}}=P_{L_{E}}=0.323$ and corresponding *c*'s). So $P_{S_{E}}$ was set to (0.2, 0.285, 0.323, 0.365) that corresponds to no effect, moderate effect, alternative hypothesis effect and a large effect. Figure 5 shows the results with $P_{S_{E}}=0.2$ that corresponds to no effect in *S*. In the case of no effect the maximum correlation between $P_{S_{E}}$ and $P_{L_{E}}$ is just over 0.7, so the maximum plotted value for the correlation was set to 0.7.

For ${\hat{P}}_{L}^{\left(1\right)}$ the corresponding cut‐off point for futility stopping was set to 0.61. For ${\hat{P}}_{S}^{\left(1\right)}$ it was equal to 0.46 for $t_{S}=0.5$ and to 0.31 for $t_{S}=0.75$. For ${\hat{P}}_{B}^{\left(1\right)}$ we considered two cut‐off points: assuming no correlation, and assuming that the endpoints are highly correlated. So the resulting values were: 0.59 assuming no correlation and 0.51 assuming high correlation for $t_{S}=0.5$, and 0.59 assuming no correlation and 0.45 assuming high for $t_{S}=0.75$. In Figure 5, we can see the results when there is no effect for the short‐term outcome, that is for $P_{S_{E}}=0.2$. It can be seen that for both $t_{S}=0.5$ and $t_{S}=0.75$ scenarios, if we choose the cut‐off point as if we assumed no correlation between *S* and *L* (c=0.51 and c=0.45) we achieve the highest power among all approaches.

The reason for that is that the cut‐off point is much lower than for ${\hat{P}}_{L}^{\left(1\right)}$ or ${\hat{P}}_{S}^{\left(1\right)}$, meaning that the trial has a lower FS caused by a high conditional power. If c=0.59 is chosen for ${\hat{P}}_{B}^{\left(1\right)}$ the results are either equal to the ones of ${\hat{P}}_{L}^{\left(1\right)}$ or slightly lower when the correlation is large. In case of $t_{s}=0.75$ and high correlation (0.7), we can see a larger decrease in overall power of ${\hat{P}}_{B}^{\left(1\right)}$ equal to around 2% points when compared to ${\hat{P}}_{L}^{\left(1\right)}$. This is caused by the fact that again, the conditional power of ${\hat{P}}_{B}^{\left(1\right)}$ is dependent on the information fraction. So if the actual correlation between *S* and *L* is high, the value of $t_{B}$ will increase that will result in lower values of the conditional power. And as it was seen before, if the correlation is high between the outcomes, a smaller value of a cut‐off point should be chosen in order to achieve the same probability to stop for futility. Therefore, it would be recommended not to assume a very low correlation structure between the endpoints if there is a chance that it might be higher as this might result in loss of power. For ${\hat{P}}_{S}^{\left(1\right)}$ a large decrease in power can be seen that is caused by no effect in the short‐term outcome.

In the Supplementary Materials in Section 2.4, we can see that ${\hat{P}}_{B}^{\left(1\right)}$ is not heavily influenced by the effect size in *S*. It was also found that the power of ${\hat{P}}_{S}^{\left(1\right)}$ increases with an increasing effect in $P_{S_{E}}$. Plots can be seen in the Supplementary Materials in Section 2.4.

#### Recommendations

If the same cut‐off point value is used for all approaches, it results in a different probability to stop for futility. Especially if we compare the fixed and observed effect conditional power approaches for each estimator. When using the observed effect, one is either too optimistic or too pessimistic and it has been shown that the distribution of the conditional power is not symmetrical (Bauer & König, 2006), which would result in too frequent futility stopping. This means that one has to use a more cautious (lower) *c* when using the observed effect approach compared with the fixed effect. Therefore, we would recommend to use the fixed effect conditional power or to adapt *c* accordingly.

The approach using *L* only results in highest power, however its probability to stop for futility is the smallest. This is simply because more data is still yet to come and only a small part of long‐term data is not yet able to distinguish between sample paths that will eventually reject the null hypothesis and those that will not. It can be seen that using both *S* and *L* results in similar power values but also a higher probability to stop for futility. Using *S* only is not encouraged as it only works if the effect sizes in *S* are similar to those in *L*. This is the advantage of using both endpoints because the estimator takes into account the effect sizes via the correlation, meaning that if there a large discrepancies between *S* and *L* we would end up with a similar decision as if we used *L* only. If *S* and *L* are correlated, we benefit from more precise interim effect estimates. Therefore, we recommend to use ${\hat{P}}_{B}^{\left(1\right)}$, especially if the difference between the amount of data available at interim for *S* and *L* is large.

In the case where cut‐off point for ${\hat{P}}_{B}^{\left(1\right)}$ is assumed to be the one for ϕ=0 or ϕ=0.9 (resulting in a lower or higher cut‐off point for the interim decision), the power of ${\hat{P}}_{B}^{\left(1\right)}$ is at least as high as the one of ${\hat{P}}_{L}^{\left(1\right)}$. In cases where the lower cut‐off point is chosen (i.e. assuming high correlation between *S* and *L*), the power gain can be substantially larger. Therefore, it would be recommended to use ${\hat{P}}_{B}^{\left(1\right)}$ assuming higher rather than low correlation structure.

## 4 SAMPLE SIZE REASSESSMENT BASED ON CONDITIONAL POWER

The methods presented above allow only for futility stopping incorporating both short‐ and long‐term endpoints but no further design adaptations in case it is decided that the trial is continued. If an interim analysis is conducted anyhow, it will be tempting to redesign an ongoing clinical trial based on the observed data, for example to increase the sample size in case the conditional power is moderate. However, if adaptations like a change of the sample size are performed, the usual test statistic simply pooling the data from both stages cannot be applied because as it is well‐known there might be an inflation of the type I error (Proschan, 1999). To achieve strict type I error control for the confirmatory test of the long‐term endpoint at the end of the trial, we will extend the adaptive combination test proposed by Bauer (1989); Bauer and Köhne (1994).

### 4.1 Adaptive combination test

Instead of pooling the data and calculating a pooled test statistic, adaptive combination test could be used at an interim analysis. It allows for flexibility while controlling type I error rate and combines the information via stage‐wise test statistics and predefined combination function (Bretz, König, Brannath, Glimm, & Posch, 2009). The combination test can be obtained using the weighted inverse normal combination function (Lehmacher & Wassmer, 1999), which can be simply written as a sum of two weighted Z‐statistics:

$$Z^{*}=\sqrt{w}Z^{\left(1\right)}+\sqrt{1-w}Z^{\left(2\right)},$$

where *w* denotes the prespecified weight that can be chosen arbitrarily as long as 0≤w≤1, and $Z^{\left(j\right)}$ (j=1,2) corresponds to the Z‐statistics of the two stages. One may use for example the predefined timing of the interim analysis, that is the information fraction from the planning stage of the trial as the weight.

Consider a simple setting, where no early rejection of the null hypothesis is allowed at the end of the first stage but an adaptation of the design such as sample size reassessment or nonbinding futility stopping can be performed (Bauer, Bretz, Dragalin, König, & Wassmer, 2016; Bretz et al., 2009; Lin et al., 2016). The adaptive combination test rejects the null hypothesis of interest if $Z^{*}>z_{1-\alpha }$.

**Figure 6:**
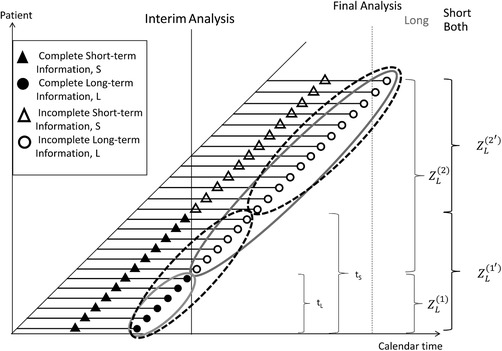
Plot showing recruitment in a trial incorporating short‐term information with combination test. At the final analysis, the *P*‐values of only long‐term estimator are combined. However, for the estimators incorporating short‐term information (${\hat{P}}_{S}^{\left(1\right)}$ and ${\hat{P}}_{B}^{\left(1\right)}$), the number of patients is higher than for using long‐term data only, ${\hat{P}}_{L}^{\left(1\right)}$. The first stage *P*‐values are calculated using the number of patients with complete‐short‐term observations at interim

We will keep the notation as introduced in Section 2, meaning that $N_{i}$ (i={E,C}) is the preplanned total sample size and the interim analysis is performed after $n_{L_{i}}$ and $n_{S_{i}}$ patients. In the interim analysis the total sample size per treatment group might be changed to ${\tilde{N}}_{i}$. In our approach the stage‐wise *P*‐values used for statistical hypothesis testing for the primary long‐term endpoint should be based on only long‐term data and not incorporate any short‐term. This procedure is straightforward when only long‐term data is used at the interim analysis, and more complex for estimators incorporating also short‐term outcomes. For ${\hat{P}}_{L}$ (where only long‐term data is used for decision‐making at interim) first stage observations correspond to the number of patients at interim $n_{L_{i}}$ per treatment group (where the long‐term outcome was observed) and the remaining observations ${\tilde{N}}_{i}-n_{L_{i}}$ (where the primary outcome was observed after the interim analysis) are included in the second stage Z‐statistic. This can be seen in Figure 6 that shows an example of recruitment and division of patients for the stage‐wise test statistics (shown as solid lines for ${\hat{P}}_{L}$). Therefore, for the final combination test we have:

$$Z_{L}^{*}=\sqrt{w}Z_{L}^{\left(1\right)}+\sqrt{1-w}Z_{L}^{\left(2\right)},$$

where $Z_{L}^{\left(1\right)}$ is obtained as defined defined in Equation (2), and $Z_{L}^{\left(2\right)}$ is a Z‐statistic obtained from from second‐stage data only:

$$Z_{L}^{\left(2\right)}=\frac{{\hat{P}}_{L_{E}}^{\left(2\right)}-{\hat{P}}_{L_{C}}^{\left(2\right)}}{\sqrt{{\bar{P}}_{L}^{\left(2\right)}\left(1-{\bar{P}}_{L}^{\left(2\right)}\right)\left(\frac{1}{{\tilde{N}}_{E}-n_{L_{E}}}+\frac{1}{{\tilde{N}}_{C}-n_{L_{C}}}\right)}},$$

where ^(2)^ corresponds to the data from the second stage from the remaining ${\tilde{N}}_{i}-n_{L_{i}}$ patients (i={E,C}).

For estimators incorporating also short‐term observations the procedure is more complex. There will be some patients for whom the short‐term information has been observed but the long‐term has not. If we included these patients in the second stage Z‐statistic, the stage‐wise test statistics would not be independent any longer. However, statistical hypothesis testing is only performed at the end of the trial and not at the interim analysis. Therefore, also the first‐stage Z‐statistic needs to be available only at the end of the trial and the combination method is applied only then. We apply the same idea as Friede et al. (2011) and Jenkins, Stone, and Jennison (2011), that is first‐stage Z‐statistics to be included in the combination test of both ${\hat{P}}_{S}^{\left(1\right)}$ and ${\hat{P}}_{B}^{\left(1\right)}$ are calculated using the number of patients with complete short‐term observations ($n_{S_{i}}$) at interim, but the data used comes from the primary endpoint. The second stage Z‐statistic is obtained from the remaining ${\tilde{N}}_{i}-n_{S_{i}}$ patients so that:

$$Z_{L}^{{*}^{\prime }}=\sqrt{w}Z_{L}^{\left(1^{\prime }\right)}+\sqrt{\left(1-w\right)}Z_{L}^{\left(2^{\prime }\right)}),$$

where $Z_{L}^{\left(1^{\prime }\right)}$ and $Z_{L}^{\left(2^{\prime }\right)}$ are the stagewise Z‐statistics obtained from the following equations:

$$Z_{L}^{\left(1^{\prime }\right)}=\frac{{\hat{P}}_{L_{E}}^{\left(1^{\prime }\right)}-{\hat{P}}_{L_{C}}^{\left(1^{\prime }\right)}}{\sqrt{{\bar{P}}_{L}^{\left(1^{\prime }\right)}\left(1-{\bar{P}}_{L}^{\left(1^{\prime }\right)}\right)\left(\frac{1}{n_{S_{E}}}+\frac{1}{n_{S_{C}}}\right)}},$$

for the first stage data, where ${}^{\left(1^{\prime }\right)}$ corresponds to the patients with long‐term observations for $n_{S_{i}}$ patients, and

$$Z_{L}^{\left(2^{\prime }\right)}=\frac{{\hat{P}}_{L_{E}}^{\left(2^{\prime }\right)}-{\hat{P}}_{L_{C}}^{\left(2^{\prime }\right)}}{\sqrt{{\bar{P}}_{L}^{\left(2^{\prime }\right)}\left(1-{\bar{P}}_{L}^{\left(2^{\prime }\right)}\right)\left(\frac{1}{{\tilde{N}}_{E}-n_{S_{E}}}+\frac{1}{{\tilde{N}}_{C}-n_{S_{C}}}\right)}},$$

for the second stage data where ${}^{\left(2^{\prime }\right)}$ corresponds to the patients with long‐term observations for ${\tilde{N}}_{i}-n_{S_{i}}$ patients. The division of patients for the stage‐wise Z‐statistics is shown in Figure 6 in dashed lines.

We propose that the weight for the combination test is based on the pre‐planned sample size $N_{i}$ and the amount of information used at interim (coming from *L* and/or *S*). This means that when we use only long‐term information for ${\hat{P}}_{L}^{\left(1\right)}$ we have $w_{L}=t_{L}$ as defined in (4) and similarly for ${\hat{P}}_{S}^{\left(1\right)}$ we have $w_{S}=t_{S}$ as defined in (5). For ${\hat{P}}_{B}^{\left(1\right)}$ two types of weights are considered. The first approach uses the information fraction of the estimator, namely $w_{B}=t_{B}$. However, as the weight has to be prespecified in the planning phase, it has to rely on the assumed correlation between *L* and *S* and cannot depend on the observed information fraction. It is calculated as in (6) in Section 2 but the assumed correlation is plugged in. The second one is a simplification that does not depend on the the correlation between *S* and *L* and is equal to the information fraction of the estimator using short‐term outcomes only so that we have $w_{B^{\prime }}=t_{S}$. We denote the approach with this choice of weights with ${\hat{P}}_{B^{\prime }}$.

For testing the binary endpoint, we used the Z‐test approximation. For small sample sizes this might not be justified. Therefore, please note that one could use exact test like Fisher's instead, for example for the combination test we would transform the stage‐wise *P*‐values *p* by using the inverse normal function in order to obtain stage‐wise Z‐statistics. This will guarantee strict type I error control.

### 4.2 Sample size reassessment based on conditional power arguments

The adaptive designs and combination test are used in order to perform a sample size reassessment during the course of the trial. At first, at the time of interim the trial a decision to either continue or stop the trial early for futility is made. If the trial is continued sample reassessment is performed. We consider two approaches for early stopping at interim. First one is based on the value of conditional power discussed in previous sections. The trial is stopped whenever CP<c, where the cut‐off points are fixed for all estimators, ${\hat{P}}_{L}^{\left(1\right)}$, ${\hat{P}}_{S}^{\left(1\right)}$, ${\hat{P}}_{B}^{\left(1\right)}$ , and ${\hat{P}}_{B^{\prime }}^{\left(1\right)}$. If the trial is continued, the Z‐statistics of first‐ and second‐stage data are calculated at the end of the trial (they are obtained from long‐term data only as discussed before) and substituted into the combination function.

In the second approach, the stopping rule is chosen to be the same for all estimators. At the first stage, a *P*‐value is calculated based on the Z‐statistic of one of the estimators, that is $Z_{L}^{\left(1\right)}$, $Z_{S}^{\left(1\right)}$ , or $Z_{B}^{\left(1\right)}$, and the stopping rule is applied to all estimators considered. This approach can make the comparisons of operating characteristics easier because the probability to stop the trial early is the same for all estimators. Then, again, if the trial is continued, the sample size reassessment is performed for each estimator.

Using the same methodology as Bauer and König (2006) for sample size reassessment, the second stage sample size is chosen in such a way that it solves the conditional power equation to be equal to the prespecified design power, 1−β under the assumption of independent increments. We assume that the second stage sample size has equal allocation ratio in treatment groups so that from now on the index *i* in sample sizes will be dropped. Equation for sample size reassessment from Bauer and König (2006) can be easily rearranged using combination test weights for both conditional power approaches. Here, we assume that the weights, are equal to the information fractions of the estimators (however any values can be chosen). The rearrangement of the formulae from Bauer and König (2006) can be found in Section 1.7 of Supplementary Materials. Note that the first stage sample size for both estimators using short‐term data is assumed to be equal to $n_{S}$. In general, for the fixed effect we have:

(8) $$\tilde{N}-n_{k}={\left(\frac{\frac{z_{1-\alpha }-\sqrt{w}Z^{\left(1\right)}}{\sqrt{1-w}}-z_{\beta }}{\left(z_{1-\alpha }+z_{1-\beta }\right)/\sqrt{N}}\right)}^{2},$$

where *Z* ^(1)^ is the interim Z‐statistic (can be $Z_{L}^{\left(1\right)}$, $Z_{S}^{\left(1\right)}$ or $Z_{B}^{\left(1\right)}$), $\tilde{N}-n_{k}$ corresponds to the adapted second stage sample size with $n_{k}$ (k={L,S}) being the first stage sample size. Similarly, for observed effect we have:

(9) $$\tilde{N}-n_{k}={\left(\frac{\frac{z_{1-\alpha }-\sqrt{w}Z^{\left(1\right)}}{\sqrt{1-w}}-z_{\beta }}{Z^{\left(1\right)}/\sqrt{tN}}\right)}^{2},$$

where *t* (can be $t_{L}$, $t_{S}$ or $t_{B}$) is the information fraction that was estimated at interim of the trial. For each approach corresponding interim Z‐statistics and information fractions can be substituted into the respective second stage sample size formula.

## 5 SIMULATIONS FOR SAMPLE SIZE REASSESSMENT

In the following simulations, we performed a nonbinding futility stopping based on either the conditional power or the Z‐statistic stopping rules, as well as sample size adaptations. We again set one‐sided α=0.025 with power 1−β=0.8 and N=200 patients per treatment arm. The second stage sample size per treatment arm was bounded to be at least half of the planned sample size, that is 0.5*N* and to be maximum 6*N*.

### 5.1 Simulation results

Similarly as for stopping for futility only, we simulated a scenario for a correlation between *S* and *L* of 0.5 in both treatment groups. A cut‐off point value for stopping for futility was chosen to be c=0.3. Probabilities of success, sample size, type I error, design power, and resulting correlations under the alternative hypothesis were chosen to be the same as for the previous examples, that is: $P_{L_{C}}=P_{S_{C}}=0.2$, $\phi_{E}=0.5$, and $\phi_{C}=0.5$. Information fractions at interim were set to be $t_{L}=0.25$ and $t_{S}=0.5$. The simulations were run using different effect sizes (0.2,0.285,0.323,0.365) for both the long‐ and short‐term endpoints for the experimental treatment group. In the paper, we show the simulation results for equal effect sizes in *L* and *S* so that $P_{L_{E}}=P_{S_{E}}$. All the other combinations are included in the Supplementary Materials in Section 3.2.

At first, we considered a simulation scenario with futility stopping based on the cut‐off point, c=0.3. Results of the simulations are summarized in Table 1. The operating characteristics of the trial that were considered include probability to stop for futility (FS), overall power, and average sample size per treatment group over both stages (ASN) including its standard deviation (reported in brackets). The first row in the table corresponds to a reference power of a one stage trial, where no interim analyses or adaptations were performed. The following rows correspond to operating characteristics of trials with futility stopping with or without sample size reassessment using ${\hat{P}}_{L}^{\left(1\right)}$, ${\hat{P}}_{B}^{\left(1\right)}$ with two sets of weights ($t_{B}$ or $t_{S}$), and ${\hat{P}}_{S}^{\left(1\right)}$. For ${\hat{P}}_{L}^{\left(1\right)}$ we also looked at the average sample size in case of no sample size reassessment. We can see that in all cases the type I error rate is controlled and it drops whenever interim analysis is performed.

**Table 1:** Operating characteristics of a trial with sample size reassessment based on fixed effect conditional power with c=0.3 as a futility stopping rule: overall power, probability to stop for futility, and average sample size per treatment arm over both stages (ASN) and its standard deviation (in brackets)

		**Probability of Success**			
	$P_{L_{E}}$	0.2	0.285	0.323	0.365
	$P_{S_{E}}$	0.2	0.285	0.323	0.365
	**Power Single Stage Trial**	0.0255	0.5112	0.8014	0.9594
**Long**	Probability to Stop for Futility	0.1163	0.0131	0.0041	9e‐04
	Power SSR	0.0248	0.5506	0.822	0.9547
	ASN SSR	262 (±88)	222 (±66)	200 (±57)	181 (±45)
	Power NO SSR	0.0254	0.5101	0.8002	0.9588
	ASN NO SSR	183 (±49)	199 (±18)	200 (±10)	200 (±5)
**Both** $t_{B}$	Probability to Stop for Futility	0.1895	0.0251	0.008	0.0018
	Power SSR	0.0255	0.6088	0.8527	0.9596
	ASN SSR	285 (±110)	259 (±76)	234 (±68)	207 (±58)
	Power NO SSR	0.0253	0.5088	0.7994	0.9584
**Both** $t_{S}$	Probability to Stop for Futility	0.6017	0.2128	0.108	0.0428
	Power SSR	0.0217	0.5205	0.7846	0.9291
	ASN SSR	174 (±100)	210 (±85)	205 (±74)	190 (±61)
	Power NO SSR	0.0221	0.4654	0.7501	0.9268
**Short**	Probability to Stop for Futility	0.6071	0.122	0.0403	0.0082
	Power SSR	0.018	0.5042	0.7628	0.9199
	ASN SSR	174 (±100)	209 (±77)	191 (±61)	170 (±43)
	Power NO SSR	0.0183	0.4781	0.7797	0.9529

Under the alternative hypothesis ($P_{L_{E}}=0.323$) with sample size reassessment, there is an increase in power for ${\hat{P}}_{L}^{\left(1\right)}$, ${\hat{P}}_{B}^{\left(1\right)}$ , and ${\hat{P}}_{B^{\prime }}^{\left(1\right)}$, when they are compared with their designs without sample size reassessment. ${\hat{P}}_{S}^{\left(1\right)}$ has a slight drop in power for effects 0.323 and 0.365 and an increase when $P_{L_{E}}=P_{S_{E}}=0.285$. Additionally ${\hat{P}}_{B}^{\left(1\right)}$ (weight $t_{B}$) and ${\hat{P}}_{L}^{\left(1\right)}$ achieve higher power than the classical one‐stage trial when sample size reassessment is performed. ${\hat{P}}_{B}^{\left(1\right)}$ has a large increase in power to over 85% that occurs at the cost of an increased average sample size of 234 patients per treatment group. ${\hat{P}}_{L}^{\left(1\right)}$ has a slight increase in power and the same ASN for the target effect of 0.323 (200 patients per treatment group). ${\hat{P}}_{B^{\prime }}^{\left(1\right)}$ (weight $t_{S}$) has an increase in ASN to 205 patients per treatment group and almost 3.5% points increase in the overall power, when compared to the same approach with no sample size reassessment.

The probability to stop for futility (FS) is however different for all four approaches that might make it difficult to compare. The highest FS is achieved by ${\hat{P}}_{B^{\prime }}^{\left(1\right)}$ with weights $t_{S}$, followed by ${\hat{P}}_{S}^{\left(1\right)}$. The probability to stop for futility for the other two estimators is less than 1% under the alternative hypothesis and is lowest for ${\hat{P}}_{L}^{\left(1\right)}$.

As comparisons between the estimators are difficult when their probability to stop for futility is different, another method was used to simulate and compare the data. The trial was stopped for futility in the same way for all estimators, based on a *P*‐value obtained from one of the estimators at the interim that was set to P=0.45. This means that the conditional power was used only for sample size reassessment (if the trial was continued). Note that here a futility stopping rule based on a beta spending function (Jennison & Turnbull, 2000; Wassmer & Brannath, 2016) could be used instead of using an arbitrary value. Data were simulated under three scenarios, with futility stopping based on a *P*‐value of a Z‐statistic of each estimator: ${\hat{P}}_{L}^{\left(1\right)}$, ${\hat{P}}_{S}^{\left(1\right)}$ , and ${\hat{P}}_{B}^{\left(1\right)}$. The results are summarized in Table 2. Again, we simulated a scenario with no sample size reassessment and in this case the interim futility stopping is also based on the *P*‐value of a chosen Z‐statistic. At the end of the trial the combination test was also performed as in the case of sample size reassessment.

**Table 2:** Operating characteristics of a trial with sample size reassessment based on fixed effect conditional power: overall power, probability to stop for futility, and average sample size per treatment arm over both stages (ASN) and its standard deviation (in brackets). Simulations with three different interim stopping approaches are shown: results with a *P*‐value based on $Z_{L}$, second one with a *P*‐value based on $Z_{B}$ and the last one with *P*‐value based on $Z_{S}$ as a stopping rule

		**Probability of success**			
	$P_{L_{E}}$	0.2	0.285	0.323	0.365
	$P_{S_{E}}$	0.2	0.285	0.323	0.365
	**Power single stage trial**	0.0255	0.5112	0.8014	0.9594
**Stopping rule with the** *P* **‐value based on** ${\hat{P}}_{L}$					
	Probability to stop for futility	0.5484	0.1885	0.098	0.0395
	Power NO SSR	0.0225	0.4714	0.7548	0.9288
	ASN NO SSR	118 (±75)	172 (±59)	186 (±45)	195 (±30)
**Long**	Power SSR	0.0216	0.471	0.7456	0.9176
	ASN SSR	157 (±68)	181 (±54)	179 (±46)	172 (±37)
**Both** $t_{B}$	Power SSR	0.0208	0.5097	0.7721	0.9219
	ASN SSR	183 (±98)	215 (±75)	210 (±63)	197 (±52)
**Both** $t_{S}$	Power SSR	0.0225	0.537	0.7943	0.9325
	ASN SSR	195 (±120)	220 (±95)	210 (±80)	192 (±65)
**Short**	Power SSR	0.0227	0.517	0.7425	0.8981
	ASN SSR	237 (±186)	216 (±108)	191 (±77)	169 (±49)
**Stopping rule with the** *P* **‐value based on** ${\hat{P}}_{B}$					
	Probability to stop for futility	0.549	0.1747	0.0843	0.0311
	Power NO SSR	0.023	0.4781	0.765	0.9364
	ASN NO SSR	118 (±75)	174 (±57)	188 (±42)	196 (±27)
**Long**	Power SSR	0.0223	0.4824	0.7598	0.9263
	ASN SSR	160 (±73)	186 (±58)	183 (±49)	174 (±40)
**Both** $t_{B}$	Power SSR	0.021	0.5181	0.7833	0.9304
	ASN SSR	180 (±93)	216 (±72)	212 (±61)	199 (±52)
**Both** $t_{S}$	Power SSR	0.0231	0.5448	0.8058	0.9405
	ASN SSR	190 (±110)	221 (±90)	212 (±78)	194 (±64)
**Short**	Power SSR	0.023	0.5236	0.753	0.9053
	ASN SSR	235 (±184)	218 (±108)	192 (±77)	170 (±49)
**Stopping rule with the** *P* **‐value based on** ${\hat{P}}_{S}$					
	Probability to stop for futility	0.5358	0.0923	0.028	0.0054
	Power NO SSR	0.0198	0.489	0.7882	0.9554
	ASN NO SSR	120 (±75)	187 (±44)	196 (±25)	200 (±11)
**Long**	Power SSR	0.0191	0.5204	0.8066	0.9506
	ASN SSR	183 (±105)	211 (±73)	198 (±59)	181 (±46)
**Both** $t_{B}$	Power SSR	0.0183	0.5751	0.8375	0.9562
	ASN SSR	209 (±133)	248 (±88)	231 (±72)	207 (±59)
**Both** $t_{S}$	Power SSR	0.0195	0.6076	0.8618	0.967
	ASN SSR	240 (±188)	268 (±131)	239 (±106)	205 (±81)
**Short**	Power SSR	0.0198	0.5241	0.7742	0.9229
	ASN SSR	195 (±114)	218 (±81)	195 (±65)	171 (±44)

For all approaches the type I error rate is controlled. It can be seen that for all futility stopping approaches, the highest power under the alternative hypothesis effect size is obtained by the estimator combining data from both short‐ and long‐term endpoints that happens at the cost of the highest average sample size obtained by both ${\hat{P}}_{B}^{\left(1\right)}$ and ${\hat{P}}_{B^{\prime }}^{\left(1\right)}$. The lowest results are achieved by ${\hat{P}}_{S}^{\left(1\right)}$. The highest probability to stop for futility is achieved in the approach using *P*‐value of $Z_{L}$ and the lowest with the use of *P*‐value of $Z_{S}$.

It can be seen that the trends for the three stopping rules are similar, that is ASN and the power are the highest for ${\hat{P}}_{B^{\prime }}^{\left(1\right)}$. The differences are in the probability to stop for futility and the higher it is, the lower are the overall power values and the higher the average sample size. ${\hat{P}}_{L}^{\left(1\right)}$ tends to have the lowest power and resulting average sample size among all approaches. It can be seen that in case of moderate power results, the estimator using both *S* and *L* has the highest increase in power, up to 10% points when compared to no sample size reassessment. Estimator incorporating both, short‐ and long‐term information has an increase in sample size, whereas the other approaches (${\hat{P}}_{L}^{\left(1\right)}$ and ${\hat{P}}_{S}^{\left(1\right)}$) have a decrease for probabilities of 0.323 and 0.365. If the effect sizes in *S* and *L* are not similar (see Supplementary Materials Section 3.1), basing the sample size reassessment on short‐term data only would result in either too low or too high power depending on the difference between short‐ and long‐term data. Again, using both *S* and *L* with weight $t_{B}$ seems to be the most robust approach resulting in consistently higher power compared to using *L* only for the price of increased ASN. The sample size increase in ${\hat{P}}_{B^{\prime }}^{\left(1\right)}$ can be explained by the fact that we have to plug in a guess for the first stage test statistic that uses the information from an equivalent of $t_{B}N$ patients.

### 5.2 Sample size reassessment for observed effect conditional power

The operating characteristics of a trial with sample size reassessment with observed effect conditional power were also investigated with the same simulation settings as in the previous section (when using the fixed effect). When using conditional power for futility stopping with c=0.3, much smaller power values are obtained even if sample size reassessment is performed. This is due to more frequent futility stopping. Such a behavior might be preferred if there is no or a small effect in *L*. Otherwise a more cautious cut‐off value would have to be used (this is in line with Section 3.2). However, when the futility stopping is based on a *P*‐value (P=0.45), the resulting average sample size would be much higher than when using fixed effect conditional power for sample size reassessment. So if one wants to employ such a sample size reassessment as a fixed rule, a better strategy would be to optimize the sample size reassessment as suggested by (Jennison & Turnbull, 2015). The results for all scenarios can be found in Supplementary Materials in Section 3.2.

### 5.3 Choice of weights

Finally, it was investigated how the choice of weights for the analysis influences the operating characteristics of the trial. Here, only a scenario under the alternative hypothesis was simulated, again for the futility stopping using *P*‐values based on $Z_{L}$, $Z_{S},$ and $Z_{B}$ at interim. We considered weights for the combination test varying from 0 to 1 in steps of 0.1. As the only difference in the approaches for ${\hat{P}}_{B}^{\left(1\right)}$ and ${\hat{P}}_{B^{\prime }}^{\left(1\right)}$ was the weight in the combination test and sample size reassessment, the results are the same if the weight is chosen to be equal for all estimators. Therefore, we only used ${\hat{P}}_{B}^{\left(1\right)}$ notation in this simulation scenario. The simulation setting was set to be the same as in the previous sections, that is $P_{L_{E}}=P_{S_{E}}=0.323$, $P_{L_{C}}=P_{S_{C}}=0.2$, N=200, $\phi_{E}=\phi_{C}=0.5$, $t_{L}=0.25$, $t_{S}=0.5$. Table 3 shows the results of simulations for the futility stopping based on *P*‐values.

**Table 3:** Operating characteristics (probability to stop for futility (FS), overall power, and average sample size (ASN) and its standard deviation) of a trial with sample size reassessment based on fixed effect conditional power with *P*‐value futility stopping rule based on *P*‐vales of $Z_{L}$, $Z_{S},$ and $Z_{B}$ for a different choice of weights for the combination test and sample size reassessment

**First stage weight**		$\sqrt{0}$	$\sqrt{0.1}$	$\sqrt{0.2}$	$\sqrt{0.3}$	$\sqrt{0.4}$	$\sqrt{0.5}$	$\sqrt{0.6}$	$\sqrt{0.7}$	$\sqrt{0.8}$	$\sqrt{0.9}$	$\sqrt{1}$
**Power single stage trial**		0.8014	0.8014	0.8014	0.8014	0.8014	0.8014	0.8014	0.8014	0.8014	0.8014	0.8014
**Stopping rule with the** *P* **‐value based on** ${\hat{P}}_{L}$												
	FS	0.098	0.098	0.098	0.098	0.098	0.098	0.098	0.098	0.098	0.098	0.098
	Power NO SSR	0.614	0.7268	0.7492	0.7579	0.7572	0.7498	0.7339	0.7032	0.6515	0.5602	0.2878
	ASN NO SSR	186 (45)	186 (45)	186 (45)	186 (45)	186 (45)	186 (45)	186 (45)	186 (45)	186 (45)	186 (45)	186 (45)
**Long**	Power SSR	0.7248	0.7282	0.7391	0.7524	0.7625	0.7711	0.7772	0.7824	0.785	0.7854	0.2725
	ASN SSR	237 (45)	188 (41)	180 (44)	179 (48)	181 (54)	186 (63)	194 (76)	209 (99)	240 (143)	335 (270)	1093 (328)
**Both**	Power SSR	0.725	0.7542	0.7638	0.7727	0.7845	0.7946	0.8018	0.8085	0.8096	0.8052	0.482
	ASN SSR	282 (60)	229 (54)	216 (58)	209 (64)	208 (71)	210 (80)	216 (94)	228 (117)	258 (163)	348 (279)	1093 (328)
**Short**	Power SSR	0.7249	0.7191	0.7186	0.7247	0.734	0.7417	0.749	0.7531	0.7508	0.743	0.483
	ASN SSR	282 (60)	217 (52)	201 (57)	193 (62)	190 (68)	191 (77)	194 (91)	204 (115)	229 (160)	310 (265)	1093 (328)
**Stopping rule with the** *P* **‐value based on** ${\hat{P}}_{B}$												
	FS	0.0843	0.0843	0.0843	0.0843	0.0843	0.0843	0.0843	0.0843	0.0843	0.0843	0.0843
	Power NO SSR	0.628	0.7392	0.7604	0.7672	0.7649	0.7552	0.7369	0.7039	0.6509	0.5602	0.2877
	ASN NO SSR	188 (42)	188 (42)	188 (42)	188 (42)	188 (42)	188 (42)	188 (42)	188 (42)	188 (42)	188 (42)	188 (42)
**Long**	Power SSR	0.7403	0.7416	0.7539	0.7672	0.776	0.7838	0.791	0.7951	0.798	0.7908	0.2729
	ASN SSR	239 (42)	191 (42)	184 (47)	183 (52)	186 (60)	191 (71)	201 (88)	218 (116)	254 (169)	355 (297)	1108 (306)
**Both**	Power SSR	0.7357	0.7637	0.7745	0.7852	0.7968	0.8063	0.8142	0.8212	0.8225	0.8182	0.4844
	ASN SSR	285 (56)	231 (51)	218 (57)	212 (62)	210 (69)	212 (78)	218 (91)	231 (112)	260 (155)	355 (277)	1108 (306)
**Short**	Power SSR	0.7358	0.7318	0.7294	0.7352	0.7436	0.7525	0.7598	0.763	0.7599	0.7524	0.4852
	ASN SSR	285 (56)	219 (51)	202 (56)	194 (61)	192 (68)	192 (77)	196 (92)	206 (116)	232 (161)	314 (266)	1108 (306)
**Stopping rule with the** *P* **‐value based on** ${\hat{P}}_{S}$												
	FS	0.028	0.028	0.028	0.028	0.028	0.028	0.028	0.028	0.028	0.028	0.028
	Power NO SSR	0.667	0.7708	0.7863	0.7872	0.7785	0.7623	0.7382	0.7	0.6445	0.5546	0.2855
	ASN NO SSR	196 (25)	196 (25)	196 (25)	196 (25)	196 (25)	196 (25)	196 (25)	196 (25)	196 (25)	196 (25)	196 (25)
**Long**	Power SSR	0.7852	0.7883	0.8001	0.8129	0.8214	0.8298	0.8368	0.8407	0.8392	0.8073	0.2703
	ASN SSR	247 (25)	202 (42)	197 (54)	199 (65)	205 (79)	215 (98)	230 (125)	257 (168)	308 (240)	425 (354)	1170 (182)
**Both**	Power SSR	0.7793	0.8167	0.8282	0.8383	0.8511	0.8629	0.8713	0.8784	0.8795	0.869	0.4836
	ASN SSR	296 (34)	246 (47)	235 (61)	231 (74)	232 (88)	239 (106)	251 (132)	274 (172)	319 (238)	430 (345)	1170 (182)
**Short**	Power SSR	0.7822	0.7678	0.7624	0.7621	0.7678	0.7743	0.7777	0.7775	0.7721	0.759	0.4849
	ASN SSR	296 (34)	226 (40)	208 (48)	199 (53)	195 (58)	195 (65)	198 (75)	207 (93)	230 (130)	316 (246)	1170 (182)

The probabilities to stop for futility are again the same as in the previous two sections. The lowest is equal to 2.8% for ${\hat{P}}_{S}^{\left(1\right)}$ and the highest is equal to 9.8% for ${\hat{P}}_{L}^{\left(1\right)}$. It can be seen that the overall power for all approaches with sample size reassessment is increasing with higher values of the first stage weight up to the values of the weight of $\sqrt{0.8}$ or $\sqrt{0.9}$ depending on the estimator and futility stopping rule. The lowest values are obtained whenever the first stage weight is converging to 1 for all approaches as assuming the first stage weight of 1 meas that second stage data is not used for hypothesis testing (vice versa for a weight of 0 only the second stage data is used). ${\hat{P}}_{B}^{\left(1\right)}$ has an increase in average sample size under all scenarios for weights from $\sqrt{0.1}$ to $\sqrt{0.8}$ varying from 8 to 119 extra patients in each treatment group depending on the scenario of interest.

When no sample size reassessment is performed, the highest overall power is achieved for the first stage weight equal to $\sqrt{0.3}$. Depending on the futility stopping rule the overall power varies from 75.79% for the stopping based on the *P*‐value of $Z_{L}$ to 78.72% for the stopping rule based on $Z_{S}$. When sample size reassessment is performed with such a choice of weights, in all the cases we see an increase in power as well as in the average sample size for ${\hat{P}}_{B}^{\left(1\right)}$ and the opposite for ${\hat{P}}_{S}^{\left(1\right)}$, that is a decrease in ASN and power. With the stopping rule based on the *P*‐value of $Z_{L}$, ${\hat{P}}_{L}^{\left(1\right)}$ has a slight decrease of power of 0.5% points and a decrease of ASN from 186 to 179 patients per treatment group. The lower the FS is, the higher is the power for all estimators and the larger is the increase in average sample size. Power of ${\hat{P}}_{B}^{\left(1\right)}$ is always increased when compared to no sample size reassessment that happens at an increase of the average sample size from recruitment of 9 to 31 extra patients. ${\hat{P}}_{S}^{\left(1\right)}$ has a decrease in both, power and ASN.

We also investigated the influence of the choice of weights for the observed effect conditional power approach, and the results are included in the Supplementary Materials at the end of Section 3.2.

#### Recommendations

The sample size reassessment based on conditional power can be performed using two approaches: fixed and observed effects. The estimator incorporating *S* and *L* has a higher power than the other estimators in most of the cases, however this happens at the cost of an increased sample size.

Even if the number of short‐term data is much larger than long‐term data using just short‐term data for both stopping for futility and sample size reassessment is not the preferred choice. This is because the quality of the decisions depends not only on the sample sizes but also on the correlation between *S* and *L* and whether the effect sizes are in a similar range. Especially, if the effect sizes are quite different, one could be heavily misguided by the larger amount of data. Therefore, it is recommended to use also *L*. The use of combination of both *S* and *L* is much more robust than simply using *S*. If the effect sizes are similar and the endpoints are correlated, one benefits from using more data with a higher precision. If the effect sizes are not similar, then the impact of the additional (misleading) data on *S* is downweighted due to consideration of observed association between *S* and *L*.

It was shown that the weights within the intervals between $t_{L}$ and $t_{S}$ result in highest power values for all approaches. Higher power is achieved for ${\hat{P}}_{B}^{\left(1\right)}$ whenever $t_{S}$ is chosen to be the weight for the sample size reassessment and combination test. Such results are often achieved with the same ASN. The reason for this is the fact that the stage‐wise *P*‐values for the combination test correspond to $n_{S}$ and $\tilde{N}-n_{S}$ of patients that makes the procedure more consistent.

## 6 DISCUSSION

Interim analyses are being widely used in drug development process for both ethical and economic reasons. By performing an interim analysis, we can stop the trial early for either efficacy or safety, or we can apply some adaptations to the design. Often, at the time of the analysis, only a small proportion of patients might have the primary, long‐term information available. However, there might be some additional patients for whom short‐term information (they have been simply observed for a shorter amount of time) is available. And as we would like to utilize as much information as possible at interim, it could be useful to add such data into the analysis. Therefore, we looked at clinical trial designs in which such data could be incorporated and investigated their operating characteristics. A two‐stage design with binary endpoints with futility stopping and sample size adaptations was considered and decision‐making process was based on conditional power.

Three different estimators were compared for two different approaches of calculating the conditional power, that is using fixed effect from planning the study and observed effect based on the results observed so far. It was shown however, that equivalent thresholds can be easily found for the approaches. If CP arguments are just used to stop for futility, then a much smaller cut‐off point for the observed approach compared to fixed approach has to be chosen. Otherwise, the trial would stop too easily for futility, even if there was a high effect.

At first, the scenario with correlation between *S* and *L* of 0.5 was considered and operational characteristics of the estimators were considered. We looked at the overall power and probability to stop for futility under 12 different simulation scenarios for each estimator and conditional power approach, where we varied the effect sizes in both long‐ and short‐term endpoints. It was seen that the estimator incorporating both outcomes was not influenced when the effect in *S* was smaller than expected. We further investigated different correlations between *S* and *L* and a small increase in power was seen, when the correlation between *S* and *L* was high. For the fixed effect approach with the same cut‐off points for all estimators, the highest power was achieved by ${\hat{P}}_{L}^{\left(1\right)}$. ${\hat{P}}_{B}^{\left(1\right)}$ had almost as high power as ${\hat{P}}_{L}^{\left(1\right)}$ for the benefit of higher probability to stop for futility.

As the same cut‐off point value resulted not only in different overall power but also in different probabilities to stop for futility, we also looked at the overall power whenever the probability to stop for futility was equal for all estimators. The data was simulated under the alternative hypothesis for different correlations between *S* and *L* (varying from no to very high correlation) and we searched for cut‐off points for which the probability to stop for futility was around 10% for all estimators. This resulted in the same overall power for the two approaches of conditional power for each estimator. The resulting cut‐off points were different for ${\hat{P}}_{B}^{\left(1\right)}$ for different correlations as the information fraction of ${\hat{P}}_{B}^{\left(1\right)}$ that is used for conditional power calculations depends on the correlation between short‐ and long‐term outcomes. It was seen that the higher the correlation between *S* and *L*, the higher the gain in overall power and it was more robust for ${\hat{P}}_{B}^{\left(1\right)}$ when cut‐off points changed with the correlation. Also, the higher the difference between amount of data available for *S* and *L* was, the higher was the power increase. For medium and high correlations ${\hat{P}}_{B}^{\left(1\right)}$ gains power over ${\hat{P}}_{L}^{\left(1\right)}$. However, it should be emphasized that the choice of the cut‐off point *c* for ${\hat{P}}_{B}^{\left(1\right)}$ relies on good knowledge of the correlation that is unknown. Therefore, we also looked at the overall power when the same cut‐off point was chosen irrespective of the correlation between *S* and *L*. We looked at two values of *c* for ${\hat{P}}_{B}^{\left(1\right)}$, when correlation of 0 and 0.9 was assumed. It was seen that in the case when no correlation structure between *S* and *L* was assumed, but there was a high effect in the short‐term outcome, there was a slight decrease in the overall power. We would recommend not to assume no correlation between *S* and *L* when designing a study, as this would result in higher cut‐off points, and hence more rigorous stopping rules.

Sample size reassessment techniques based on conditional power were also investigated. The combination test was applied in order to control the type I error, and was used for the final analysis in the trial. Overall power, probability to stop for futility and average sample size were obtained for different effect sizes (from no to very high, corresponding to 95% power) in a simulated trial. We looked at sample size reassessment where the futility stopping rule was based on a cut‐off point *c*. However, such results again returned different probability to stop for futility for all estimators, making the comparisons between the estimators difficult. Under this scenario, ${\hat{P}}_{B}$ yielded the highest power, at the cost of the highest average sample size. When the probability to stop for futility was set to be equal for all estimators, and based on *P*‐value of ${\hat{P}}_{L}^{\left(1\right)}$, the estimator incorporating short‐term data had higher overall power, again at the cost of the average sample size.

Use of the observed effect for sample size reassessment resulted in higher power than the use of the fixed effect, however it happened at the cost of much higher average sample size. Finally, we investigated how the choice of the weights for the combination test, and sample size reassessment influences the operating characteristics of the trial. It could be seen, that if the same weights were to be chosen for all estimators, ${\hat{P}}_{B}^{\left(1\right)}$ would result in the highest overall power, at a cost of sample size increase when compared to a trial with no sample size reassessment or ${\hat{P}}_{L}^{\left(1\right)}$. For the choice of weights between 0.2 and 0.5 with *P*‐value stopping futility rule, ${\hat{P}}_{B}^{\left(1\right)}$ achieves higher power than the other two estimators. It can be concluded that incorporation of short‐term information into interim analyses could be beneficial in terms of power under some scenarios but would result in a sample size increase.

Using short‐term information could be a valuable approach in conducting interim analyses and could increase the overall power of the trial. If only futility stopping is considered at interim, then ${\hat{P}}_{B}^{\left(1\right)}$ when chosen with appropriate cut‐off points can achieve at least the power of ${\hat{P}}_{L}^{\left(1\right)}$. We can see that the estimator incorporating information from both, short‐ and long‐term outcomes achieves higher power, whenever the effect size in the short‐term outcome is close to the long‐term one. If sample size reassessment is performed, there can be a substantial gain in power, however this happens at the cost of an increased sample size. For the choice of futility stopping rules there is a trade‐off between overall power and saving sample sizes in case of expected futility. One may define utility functions to balance these events. As pointed out by one reviewer this could be done by applying the expected net present value (difference between the expected rewards and sampling costs of a trial) (Antonijevic et al., 2013). Such utility function could capture how well a futility rule is performing in terms of balancing the competing aims of abandoning quickly an ineffective drug with little chance of success and completing the development of an effective drug that is likely to succeed and yield a large reward. In the context of adaptive interim analysis with sample size reassessment such utility functions could use to optimize the stopping as well as the sample size reassessment rule (Jennison & Turnbull, 2006, 2015).

There is a number of limitations and potential extensions concerning this work. The setting was only considered within the frequentist framework. It could be extended to Bayesian approaches, such as the use of predictive power (Spiegelhalter, Freedman, & Blackburn, 1986). What is more, one could extend the sample size reassessment methodology to different type of endpoints, for example continuous or survival. The design setting within the optimisation framework could be also considered, in which the gains/losses of a trial could be investigated in terms of for example expected net present value (Antonijevic et al., 2013). One could for example assign the cost of recruiting one additional patient in the trial and the reward of gaining 1% of power and find an optimal design for a given set of rewards.

To summarize, incorporating short‐term information improves decision making at an interim analysis both for futility stopping and sample size reassessment, especially if there is a large difference in the amount of data available on short‐ and long‐term endpoints. Our investigation showed that there is no substantial difference between basing the interim analysis on the short‐term endpoint only or on combination of both, as long as the true (but unknown) efficacy on *S* and *L* is similar and the data are correlated. However, if this is not the case, the method incorporating both is preferable as in such cases it automatically downweighs the impact of the short‐term endpoint.

## CONFLICT OF INTEREST

The authors have declared no conflict of interest.

## Supporting information

Supporting Information
