# Supplementary material for: Interim analysis incorporating short‐ and long‐term binary endpoints
Source: Biom J. 2019 Jan 29;61(3):665–87. doi: 10.1002/bimj.201700281 (PMC6590444; doi:10.1002/bimj.201700281)
Supplement: Supplementary file 1 — Supporting Information [file BIMJ-61-665-s001.zip › Reproducibility_Julia_Niewczas/README.pdf]

Output: Interim analysis incorporating short- and long-term binary endpoints

Introduction

In this file, all the steps for reproducing plots and tables in the main body of the article can be found. The document is divided into subsections, each corresponding to a given Figure/Table. The output is produced using the R-Package `binfutssr`.

The package consists of a set of functions which calculate the operating characteristics of simulated clinical trials with interim analyses incorporating short- and long-term binary endpoints.

In order to create the first plot, load the package `binfutssr` (with its dependencies) and type in the following code. The code is also available in a seperate file called 'output.R'. One scenario of 100,000 simulations takes approximately 2 min for interim analysis with futility stopping and 2.5 min for sample size reassessments. If one has access to multiple cores, the packages `parallel` and `doParallel` can be loaded and the number of cores can be specified using function `cl <- makeCluster(m)` with m coresponding to the number of cores. The cores can be registered using `registerDoParallel(cl)`. The code for provision of graphics uses the `foreach` function which if more than 1 core is specified parallises the procedure.

At first, fix the setting for trial simulation

```
library(psych)
library(ggplot2)
```

```
##
## Attaching package: 'ggplot2'

## The following objects are masked from 'package:psych':
##
##     %+%, alpha
```

```
library(foreach)
library(mvtnorm)
library(stringr)
library(gridExtra)
library(parallel)
library(doParallel)
```

```
## Loading required package: iterators
```

```
library(xtable)
library(knitr)
library(kableExtra)
library(binfutssr) # need to install this locally from .tar.gz file
```

```
## Loading required package: grid
```

```
sessionInfo()
```

```
## R version 3.2.4 (2016-03-10)
## Platform: x86_64-pc-linux-gnu (64-bit)
## Running under: CentOS Linux 7 (Core)
##
## locale:
```

```
##  [1] LC_CTYPE=en_US.UTF-8       LC_NUMERIC=C
##  [3] LC_TIME=en_US.UTF-8        LC_COLLATE=en_US.UTF-8
##  [5] LC_MONETARY=en_US.UTF-8    LC_MESSAGES=en_US.UTF-8
##  [7] LC_PAPER=en_US.UTF-8       LC_NAME=C
##  [9] LC_ADDRESS=C               LC_TELEPHONE=C
## [11] LC_MEASUREMENT=en_US.UTF-8 LC_IDENTIFICATION=C
##
## attached base packages:
## [1] grid      parallel  stats     graphics  grDevices utils     datasets
## [8] methods   base
##
## other attached packages:
##  [1] binfutssr_0.1.0   kableExtra_0.9.0  knitr_1.20
##  [4] xtable_1.8-2      doParallel_1.0.10 iterators_1.0.8
##  [7] gridExtra_2.2.1   stringr_1.2.0     mvtnorm_1.0-5
## [10] foreach_1.4.3     ggplot2_2.2.1     psych_1.5.8
##
## loaded via a namespace (and not attached):
##  [1] Rcpp_0.12.12      rstudioapi_0.7    xml2_1.2.0
##  [4] magrittr_1.5      rvest_0.3.2       mnormt_1.5-4
##  [7] munsell_0.4.3     viridisLite_0.2.0 colorspace_1.2-6
## [10] R6_2.1.2          httr_1.1.0        plyr_1.8.3
## [13] tools_3.2.4       gtable_0.2.0      htmltools_0.3.5
## [16] yaml_2.1.13       lazyeval_0.2.0    rprojroot_1.2
## [19] digest_0.6.9      tibble_1.3.0      readr_1.0.0
## [22] codetools_0.2-14  evaluate_0.10     rmarkdown_1.10
## [25] stringi_1.1.6     scales_0.4.1      backports_1.0.2
```

Futility Stopping

Now define a scenario from the main article and simulate data

```
p_se <- c(0.2, 0.285, 0.3227348, 0.365)

m <- length(p_se) # if multiple cores are available set this value to length of p_se
cl <- parallel::makeCluster(m)
doParallel::registerDoParallel(cl)
```

Simulate the scenario under alternative hypothesis

```
output_cp_alt <- foreach(i = 1:length(p_se), .packages="binfutssr") %dopar%
  (cp(nsim = 100000,        #number of simulations
      alpha = 0.025,         # one-sided alpha
      beta = 0.2,            # type 2 error such that 1-beta is the power
      p_le = 0.3227348,      # probability of success for long-term endpoint in E
      p_lc = 0.2,            # probability of success for long-term endpoint in C
      p_se = p_se[i],        # i-th probability of success for short-term endpoint in E
      p_sc = 0.2,            # probability of success for short-term endpoint in C
      n = 200,               # sample size per treatment arm
      fr_lo = 0.25,          # amounut of long-term information available at interim
      fr_sh = 0.5,           # amounut of short-term information available at interim
      phi_e = 0.5,           # correlation in E
      phi_c = 0.5,           # correlation in C
      c = seq(0, 1, 0.01)))  #sequence of cut-off points for stopping based on cp
```

Simulate clinical trial for moderate power (~50%)

```
output_cp_mod <- foreach(i = 1:length(p_se), .packages="binfutssr") %dopar%
  (cp(nsim = 100000,
      alpha = 0.025,
      beta = 0.2,
      p_le = 0.285,
      p_lc = 0.2,
      p_se = p_se[i],
      p_sc = 0.2,
      n = 200,
      fr_lo = 0.25,
      fr_sh = 0.5,
      phi_e = 0.5,
      phi_c = 0.5,
      c = seq(0, 1, 0.01)))
```

Simulate clinical trial under the null hypothesis

```
output_cp_null <- foreach(i = 1:length(p_se), .packages="binfutssr") %dopar%
  (cp(nsim = 100000,
      alpha = 0.025,
      beta = 0.2,
      p_le = 0.2,
      p_lc = 0.2,
      p_se = p_se[i],
      p_sc = 0.2,
      n = 200,
      fr_lo = 0.25,
```

```
      fr_sh = 0.5,
      phi_e = 0.5,
      phi_c = 0.5,
      c = seq(0, 1, 0.01)))
```

Figure 1 and Figure 2

Use the code below to produce the required plots for Figure 1 - Overall Power and Figure 2 - Probability to Stop for Futility. The last entry in the function specifies whether the function should save the figure into file or not. Options are `pdf`, `eps` and `none`. If `none` is selected, the plot is printed.

```
power_plots <- create_power_plots(output_cp_alt = output_cp_alt,
                                  output_cp_mod = output_cp_mod,
                                  output_cp_null = output_cp_null,
                                  phi_e = 0.5,
                                  phi_c = 0.5,
                                  filetype = "none")
```

```
grid.arrange(power_plots)
```

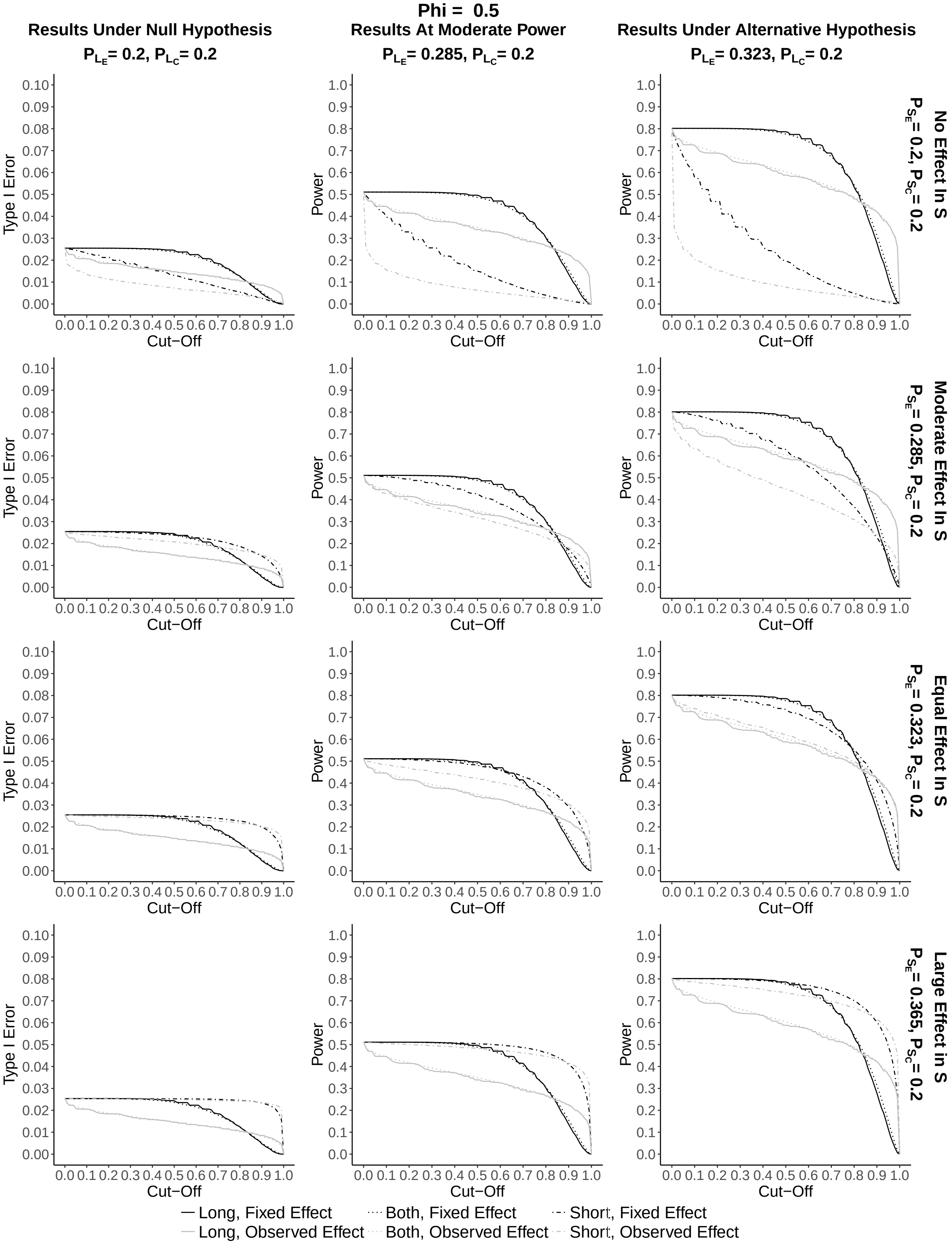

Phi = 0.5
Results Under Null Hypothesis
P_{L_E}= 0.2, P_{L_C}= 0.2
Results At Moderate Power
P_{L_E}= 0.285, P_{L_C}= 0.2
Results Under Alternative Hypothesis
P_{L_E}= 0.323, P_{L_C}= 0.2
No Effect In S
P_{S_E}= 0.2, P_{S_C}= 0.2
Moderate Effect In S
P_{S_E}= 0.285, P_{S_C}= 0.2
Equal Effect In S
P_{S_E}= 0.323, P_{S_C}= 0.2
Large Effect in S
P_{S_E}= 0.365, P_{S_C}= 0.2
Type I Error
Power
Cut−Off
Long, Fixed Effect
Both, Fixed Effect
Short, Fixed Effect
Long, Observed Effect
Both, Observed Effect
Short, Observed Effect

```r
fs_plots <- create_fs_plots(output_cp_alt = output_cp_alt,
                            output_cp_mod = output_cp_mod,
                            output_cp_null = output_cp_null,
                            phi_e = 0.5,
                            phi_c = 0.5,
                            filetype = "none")
```

```r
grid.arrange(fs_plots)
```

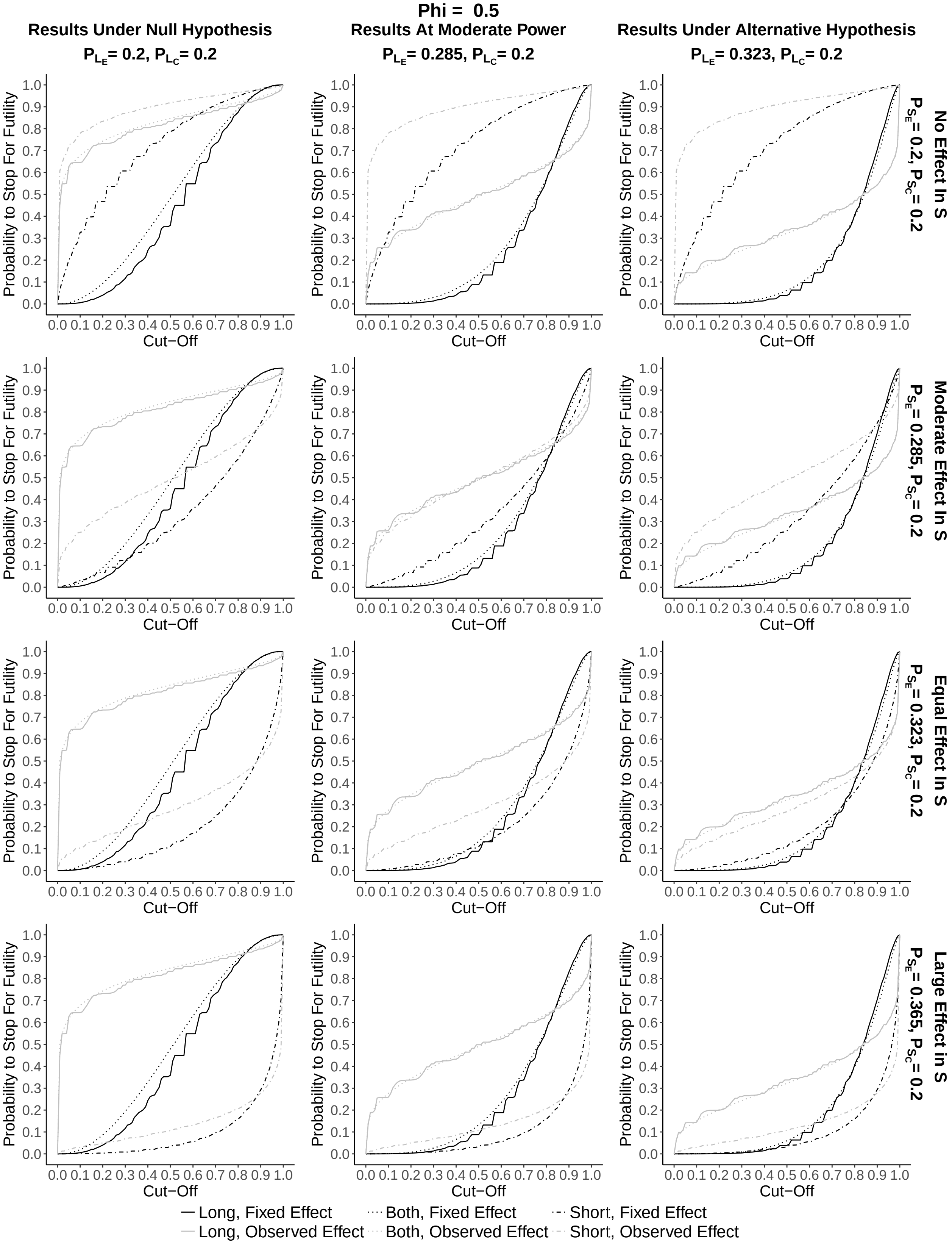

Phi = 0.5
Results Under Null Hypothesis
P_{L_E}= 0.2, P_{L_C}= 0.2
Results At Moderate Power
P_{L_E}= 0.285, P_{L_C}= 0.2
Results Under Alternative Hypothesis
P_{L_E}= 0.323, P_{L_C}= 0.2
No Effect In S
P_{S_E}= 0.2, P_{S_C}= 0.2
Moderate Effect In S
P_{S_E}= 0.285, P_{S_C}= 0.2
Equal Effect In S
P_{S_E}= 0.323, P_{S_C}= 0.2
Large Effect in S
P_{S_E}= 0.365, P_{S_C}= 0.2
Probability to Stop For Futility
Cut−Off
Long, Fixed Effect
Both, Fixed Effect
Short, Fixed Effect
Long, Observed Effect
Both, Observed Effect
Short, Observed Effect

Figure 3

In order to obtain Figure three which shows equivalence of cut-off points one has to run the `fixed_to_obs_plot` function.

```
fixed_to_obs <- fixed_to_obs_plot(
  alpha = 0.025,                       # type 1 error
  beta = 0.2,                          # type 2 error
  t = c(0.1, 0.25, 0.5, 0.75, 0.9), # vector of information fractions for equivalent c's
  c = seq(0, 1, 0.01),                 # sequence of cut-off points for which the data is obtained
  filetype = "none")                   # save the figure? options: c("pdf", "eps", "none")
```

```
grid.arrange(fixed_to_obs)
```

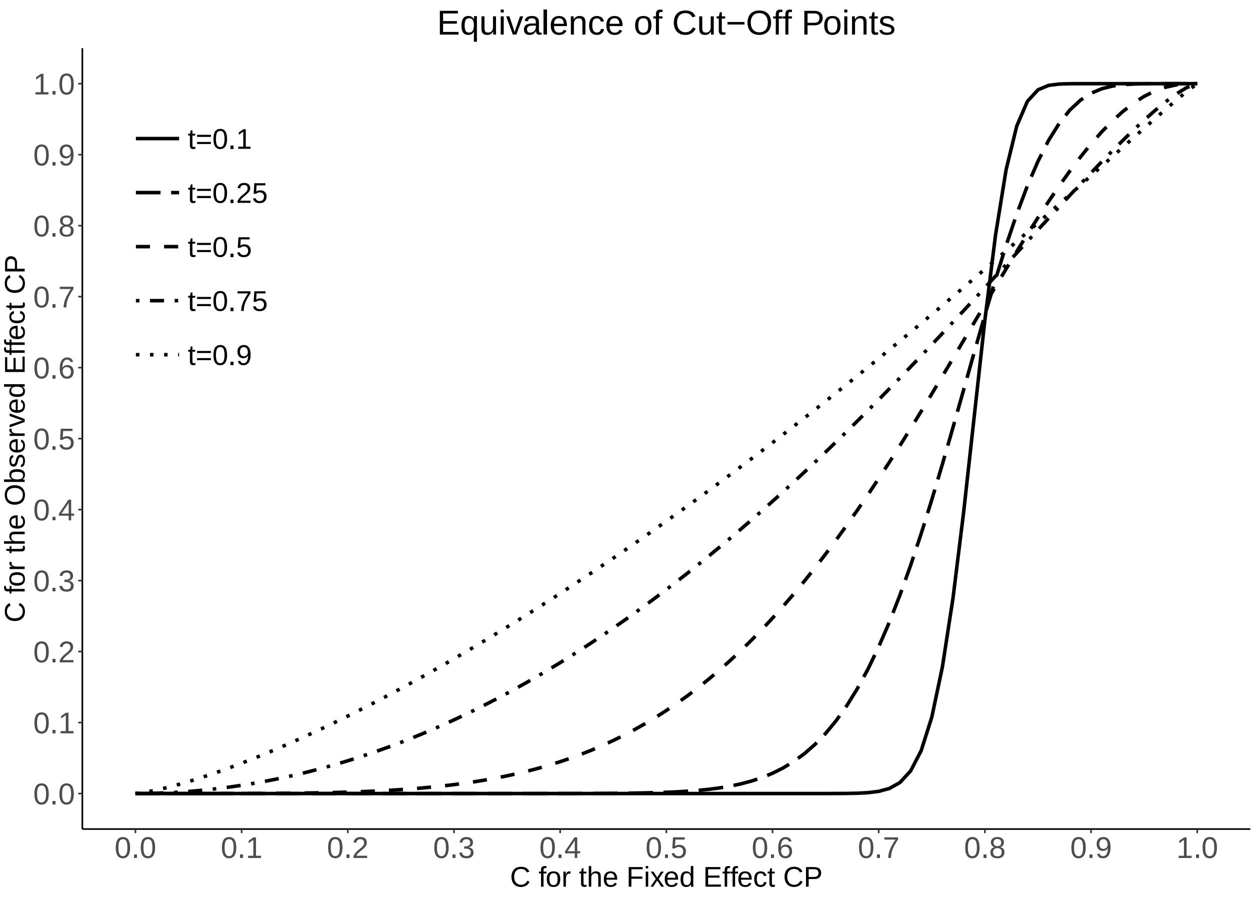

Varrying Correlation and Information Fraction

In this section, the code for producing the correlation plots. In this simulation scenario the cut-off points are chosen separately for each estimator so that for each approach the probability to stop for futility under the alternative hypothesis is 10%. Then, the overall power was reported for each cut-off point and approach and compared. This is done for cases when $t_l = 0.25$ and two values of t_s: 0.5 and 0.75.

At first, simulate clinical trial scenarios with the following set-up using `cp` function:

Figure 4

```
cl <- parallel::makeCluster(m)
doParallel::registerDoParallel(cl)

phi_e = c(0, 0.2, 0.5, 0.7, 0.9)
phi_c = c(0, 0.2, 0.5, 0.7, 0.9)


output_cp_alt_fr_sh_05 <- foreach(i = 1:length(phi_e),
                                           .packages="binfutssr") %dopar%
  (cp(nsim = 100000,
      alpha = 0.025,
      beta = 0.2,
      p_le = 0.3227348,
      p_lc = 0.2,
      p_se = 0.3227348,
      p_sc = 0.2,
      n = 200,
      fr_lo = 0.25,
      fr_sh = 0.5,
      phi_e = phi_e[i],
      phi_c = phi_c[i],
      c = seq(0, 1, 0.01)))


output_cp_alt_fr_sh_075 <- foreach(i = 1:length(phi_e),
                                            .packages="binfutssr") %dopar%
  (cp(nsim = 100000,
      alpha = 0.025,
      beta = 0.2,
      p_le = 0.3227348,
      p_lc = 0.2,
      p_se = 0.3227348,
      p_sc = 0.2,
      n = 200,
      fr_lo = 0.25,
      fr_sh = 0.75,
      phi_e = phi_e[i],
      phi_c = phi_c[i],
      c = seq(0, 1, 0.01)))
```

After the data has been simulated create a plot showing the overall power using `create_corr_plots` function.

```r
plots_corr <- create_corr_plots(output_cp_alt_fr_sh_05 = output_cp_alt_fr_sh_05,
                                output_cp_alt_fr_sh_075 = output_cp_alt_fr_sh_075,
                                phi_e = phi_e,
                                phi_c = phi_c,
                                fr_lo = 0.25,
                                filetype = "none")
```

```r
grid.arrange(plots_corr[[length(plots_corr)]])
```

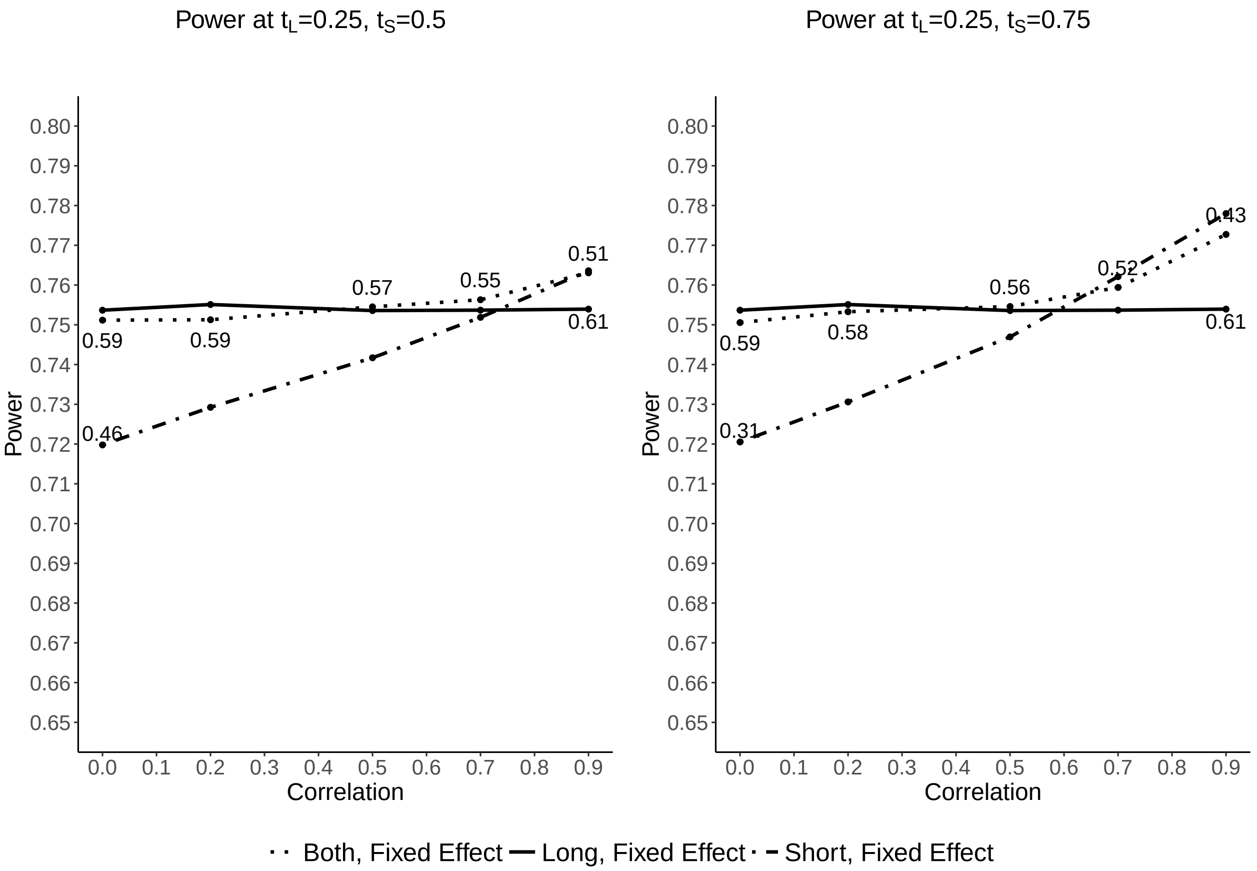

Now based on the results from the previous simulation another case is considered. The cut-off points for obtaining conditional power are fixed from previous simulation scenario. However, the true probability of P_{SE} is changed to no effect. At first, simulate a scenario in such a setting. A case with $\Phi = 0.9$ is excluded as such a correlation is not admissible.

Figure 5

```r
phi_e_v2 <- c(0, 0.2, 0.5, 0.7) # Fix new correlation
phi_c_v2 <- c(0, 0.2, 0.5, 0.7)

output_cp_alt_fr_sh_05_p_se_02 <- foreach(i = 1:length(phi_e_v2),
                                                  .packages="binfutssr") %dopar%
  (cp(nsim = 100000,
      alpha = 0.025,
      beta = 0.2,
      p_le = 0.3227348,
```

```r
      p_lc = 0.2,
      p_se = 0.2,
      p_sc = 0.2,
      n = 200,
      fr_lo = 0.25,
      fr_sh = 0.5,
      phi_e = phi_e_v2[i],
      phi_c = phi_c_v2[i],
      c = seq(0, 1, 0.01)))

output_cp_alt_fr_sh_075_p_se_02 <- foreach(i = 1:length(phi_e_v2),
                                                    .packages="binfutssr") %dopar%
  (cp(nsim = 100000,
      alpha = 0.025,
      beta = 0.2,
      p_le = 0.3227348,
      p_lc = 0.2,
      p_se = 0.2,
      p_sc = 0.2,
      n = 200,
      fr_lo = 0.25,
      fr_sh = 0.75,
      phi_e = phi_e_v2[i],
      phi_c = phi_c_v2[i],
      c = seq(0, 1, 0.01)))
```

Now, create a plot with overall power using function `create_corr_plots_p_se_low`

```r
plots_corr_p_se_low <- create_corr_plots_p_se_low(
  output_cp_alt_fr_sh_05_p_se_02 = output_cp_alt_fr_sh_05_p_se_02,
  output_cp_alt_fr_sh_075_p_se_02 = output_cp_alt_fr_sh_075_p_se_02,
  plots_corr = plots_corr,
  phi_e = phi_e_v2,
  phi_c = phi_c_v2,
  fr_lo = 0.25,
  filetype = "none")
```

```r
grid.arrange(plots_corr_p_se_low)
```

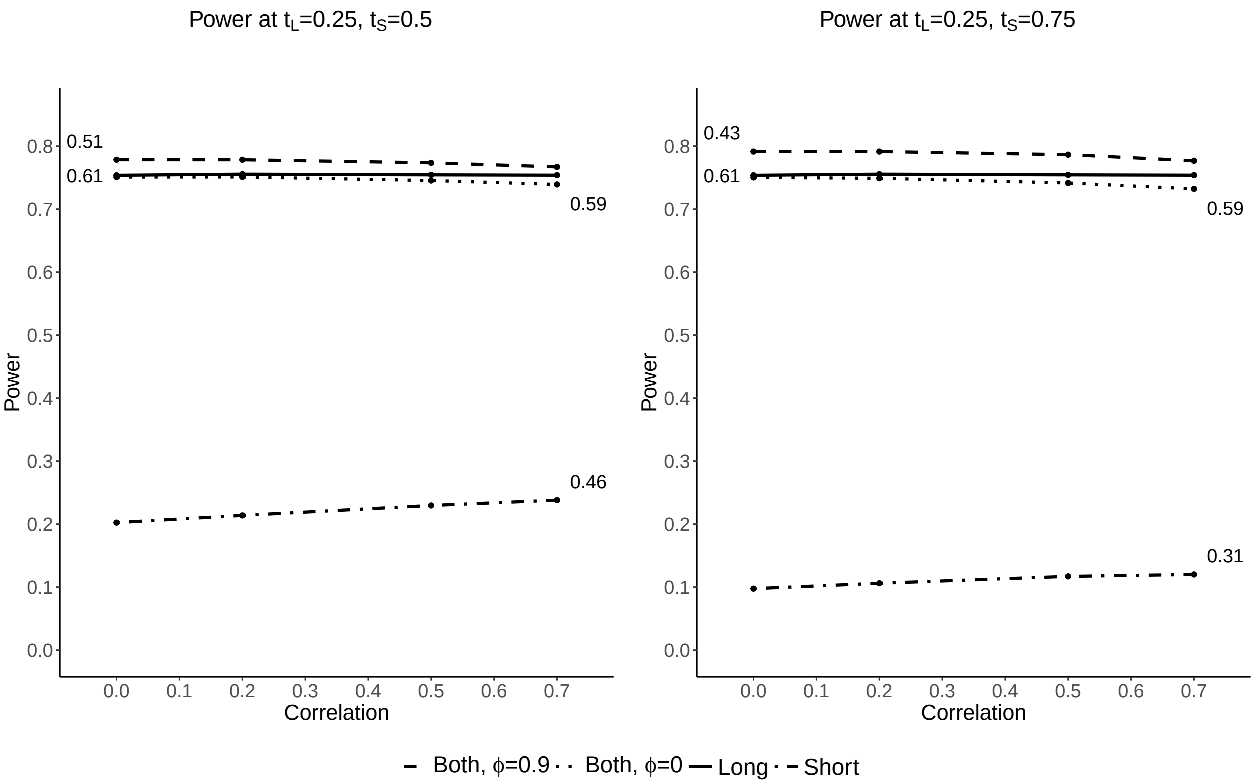
Power at t_L=0.25, t_S=0.5
Power at t_L=0.25, t_S=0.75
Power
Correlation
0.51
0.61
0.59
0.46
0.43
0.61
0.59
0.31
0.0
0.1
0.2
0.3
0.4
0.5
0.6
0.7
0.8
Both, ϕ=0.9
Both, ϕ=0
Long
Short

Sample Size Reassessments

In this section, simulation scenarios for sample size reassessments are created. Here, latex tables are printed. The operating characteristics compared are probability to stop for futility, overall power and average sample size with its standard deviation.

Futility Stopping based on cut-off point of conditional power, Table 1

At first, simulate a scenario where futility stopping is based on a pre-specified cut-off point for the conditional power. Then sample size reassessment is performed based on fixed effect conditional power for all three estimators. This is done for 4 effect sizes: no effect, moderate effect, alternative hypothesis effect and high effect. The effects in P_{LE} and P_{SE} are assumed to be equal. The table with results is printed using function `create_ssr_fixed_table`. Save option can be either true or false. If set `FALSE`then it is printed.

		**Probability of Success**			
	$\mathbf{P_{L_E}}$	0.2	0.285	0.323	0.365
	$\mathbf{P_{S_E}}$	0.2	0.285	0.323	0.365
	**Power Single Stage Trial**	0.0255	0.5112	0.8014	0.9594
**Long**	Probability to Stop for Futility	0.1163	0.0131	0.0041	9e-04
	Power SSR	0.0248	0.5506	0.822	0.9547
	ASN SSR	262 (±88)	222 (±66)	200 (±57)	181 (±45)
	Power NO SSR	0.0254	0.5101	0.8002	0.9588
	ASN NO SSR	183 (±49)	199 (±18)	200 (±10)	200 (±5)
**Both** t_B	Probability to Stop for Futility	0.1895	0.0251	0.008	0.0018
	Power SSR	0.0255	0.6088	0.8527	0.9598
	ASN SSR	285 (±110)	259 (±76)	234 (±68)	207 (±58)
	Power NO SSR	0.0253	0.5088	0.7994	0.9584
**Both** t_S	Probability to Stop for Futility	0.6017	0.2128	0.108	0.0428
	Power SSR	0.0217	0.5205	0.7846	0.9292
	ASN SSR	174 (±100)	210 (±85)	205 (±74)	190 (±61)
	Power NO SSR	0.0221	0.4654	0.7501	0.9268
**Short**	Probability to Stop for Futility	0.6071	0.122	0.0403	0.0082
	Power SSR	0.018	0.5042	0.7628	0.9199
	ASN SSR	174 (±100)	209 (±77)	191 (±61)	170 (±43)
	Power NO SSR	0.0183	0.4781	0.7797	0.9529

Table 1: Operating characteristics of a trial with sample size reassessment based on fixed effect conditional power with $c = 0.3$ as a futility stopping rule: overall power, probability to stop for futility, and average sample size per treatment arm over both stages (ASN) and its standard deviation (in brackets).

Futility Stopping based on p-value, Table 2

In this section the table for sample size reassessment based on fixed effect conditional power. The futility stopping is based on a p-value of one of the Z-statistics of the estimators. In such a case the trial is continued at the same time for all approaches, making the comparisons more straightforward.

		Probability of Success			
	$\mathbf{P_{L_E}}$	0.2	0.285	0.323	0.365
	$\mathbf{P_{S_E}}$	0.2	0.285	0.323	0.365
	**Power Single Stage Trial**	0.0255	0.5112	0.8014	0.9594
**Stopping Rule With The P-value Based On** $\hat{P}_L$					
	Probability to Stop for Futility	0.5484	0.1885	0.098	0.0395
	Power NO SSR	0.0225	0.4714	0.7548	0.9288
	ASN NO SSR	118 (±75)	172 (±59)	186 (±45)	195 (±30)
**Long**	Power SSR	0.0216	0.471	0.7456	0.9176
	ASN SSR	157 (±68)	181 (±54)	179 (±46)	172 (±37)
**Both** t_B	Power SSR	0.0208	0.5097	0.7721	0.9222
	ASN SSR	183 (±98)	215 (±75)	210 (±63)	197 (±52)
**Both** t_S	Power SSR	0.0225	0.537	0.7943	0.9324
	ASN SSR	195 (±120)	220 (±95)	210 (±80)	192 (±65)
**Short**	Power SSR	0.0227	0.517	0.7425	0.898
	ASN SSR	237 (±186)	216 (±108)	191 (±77)	169 (±49)
**Stopping Rule With The P-value Based On** $\hat{P}_B$					
	Probability to Stop for Futility	0.549	0.1747	0.0843	0.0311
	Power NO SSR	0.023	0.4781	0.765	0.9364
	ASN NO SSR	118 (±75)	174 (±57)	188 (±42)	196 (±27)
**Long**	Power SSR	0.0223	0.4824	0.7598	0.9263
	ASN SSR	160 (±73)	186 (±58)	183 (±49)	174 (±40)
**Both** t_B	Power SSR	0.021	0.5181	0.7833	0.9304
	ASN SSR	180 (±93)	216 (±72)	212 (±61)	199 (±52)
**Both** t_S	Power SSR	0.0231	0.5448	0.8058	0.9408
	ASN SSR	190 (±110)	221 (±90)	212 (±78)	194 (±64)
**Short**	Power SSR	0.023	0.5236	0.753	0.905
	ASN SSR	235 (±184)	218 (±108)	192 (±77)	170 (±49)
**Stopping Rule With The P-value Based On** $\hat{P}_S$					
	Probability to Stop for Futility	0.5358	0.0923	0.028	0.0054
	Power NO SSR	0.0198	0.489	0.7882	0.9554
	ASN NO SSR	120 (±75)	187 (±44)	196 (±25)	200 (±11)
**Long**	Power SSR	0.0191	0.5204	0.8066	0.9506
	ASN SSR	183 (±105)	211 (±73)	198 (±59)	181 (±46)
**Both** t_B	Power SSR	0.0183	0.5751	0.8375	0.9561
	ASN SSR	209 (±133)	248 (±88)	231 (±72)	207 (±59)
**Both** t_S	Power SSR	0.0195	0.6076	0.8618	0.9669
	ASN SSR	240 (±188)	268 (±131)	239 (±106)	205 (±81)
**Short**	Power SSR	0.0198	0.5241	0.7742	0.9231
	ASN SSR	195 (±114)	218 (±81)	195 (±65)	171 (±44)

Table 2: Operating characteristics of a trial with sample size reassessment based on fixed effect conditional power: overall power, probability to stop for futility, and average sample size per treatment arm over both stages (ASN) and its standard deviation (in brackets). Simulations with 3 different interim stopping approaches are shown: results with a p-value based on Z_L, second one with a p-value based on Z_B and the last one with p-value based on Z_S as a stopping rule.

Different weights, Table 3

In this section the last table from the main article is printed. Here the weight in the combination test as well as sample size reassessment formula is fixed to be equal for all approaches. It is varied from 0 to 1 in steps of 0.1. The futility stopping is based on the p-value of one of the estimators.

First Stage Weight		$\sqrt{0}$	$\sqrt{0.1}$	$\sqrt{0.2}$	$\sqrt{0.3}$	$\sqrt{0.4}$	$\sqrt{0.5}$	$\sqrt{0.6}$	$\sqrt{0.7}$	$\sqrt{0.8}$	$\sqrt{0.9}$	$\sqrt{1}$
**Power Single Stage Trial**		0.8014	0.8014	0.8014	0.8014	0.8014	0.8014	0.8014	0.8014	0.8014	0.8014	0.8014
**Stopping Rule with the P-value based on** $\hat{P}_L$												
	FS	0.098	0.098	0.098	0.098	0.098	0.098	0.098	0.098	0.098	0.098	0.098
	Power NO SSR	0.614	0.7268	0.7492	0.7579	0.7572	0.7498	0.7339	0.7032	0.6515	0.5602	0.2878
	ASN NO SSR	186 (45)	186 (45)	186 (45)	186 (45)	186 (45)	186 (45)	186 (45)	186 (45)	186 (45)	186 (45)	186 (45)
**Long**	Power SSR	0.7248	0.7282	0.7391	0.7524	0.7625	0.7711	0.7772	0.7824	0.785	0.7854	0.2725
	ASN SSR	237 (45)	188 (41)	180 (44)	179 (48)	181 (54)	186 (63)	194 (76)	209 (99)	240 (143)	335 (270)	1093 (328)
**Both**	Power SSR	0.725	0.7542	0.7638	0.7727	0.7845	0.7946	0.8018	0.8085	0.8096	0.8052	0.482
	ASN SSR	282 (60)	229 (54)	216 (58)	209 (64)	208 (71)	210 (80)	216 (94)	228 (117)	258 (163)	348 (279)	1093 (328)
**Short**	Power SSR	0.7249	0.7191	0.7186	0.7247	0.734	0.7417	0.749	0.7531	0.7508	0.743	0.483
	ASN SSR	282 (60)	217 (52)	201 (57)	193 (62)	190 (68)	191 (77)	194 (91)	204 (115)	229 (160)	310 (265)	1093 (328)
**Stopping Rule with the P-value based on** $\hat{P}_B$												
	FS	0.0843	0.0843	0.0843	0.0843	0.0843	0.0843	0.0843	0.0843	0.0843	0.0843	0.0843
	Power NO SSR	0.628	0.7392	0.7604	0.7672	0.7649	0.7552	0.7369	0.7039	0.6509	0.5602	0.2877
	ASN NO SSR	188 (42)	188 (42)	188 (42)	188 (42)	188 (42)	188 (42)	188 (42)	188 (42)	188 (42)	188 (42)	188 (42)
**Long**	Power SSR	0.7403	0.7416	0.7539	0.7672	0.776	0.7838	0.791	0.7951	0.798	0.7908	0.2729
	ASN SSR	239 (42)	191 (42)	184 (47)	183 (52)	186 (60)	191 (71)	201 (88)	218 (116)	254 (169)	355 (297)	1108 (306)
**Both**	Power SSR	0.7357	0.7637	0.7745	0.7852	0.7968	0.8063	0.8142	0.8212	0.8225	0.8182	0.4844
	ASN SSR	285 (56)	231 (51)	218 (57)	212 (62)	210 (69)	212 (78)	218 (91)	231 (112)	260 (155)	355 (277)	1108 (306)
**Short**	Power SSR	0.7358	0.7318	0.7294	0.7352	0.7436	0.7525	0.7598	0.763	0.7599	0.7524	0.4852
	ASN SSR	285 (56)	219 (51)	202 (56)	194 (61)	192 (68)	192 (77)	196 (92)	206 (116)	232 (161)	314 (266)	1108 (306)
**Stopping Rule with the P-value based on** $\hat{P}_S$												
	FS	0.028	0.028	0.028	0.028	0.028	0.028	0.028	0.028	0.028	0.028	0.028
	Power NO SSR	0.667	0.7708	0.7863	0.7872	0.7785	0.7623	0.7382	0.7	0.6445	0.5546	0.2855
	ASN NO SSR	196 (25)	196 (25)	196 (25)	196 (25)	196 (25)	196 (25)	196 (25)	196 (25)	196 (25)	196 (25)	196 (25)
**Long**	Power SSR	0.7852	0.7883	0.8001	0.8129	0.8214	0.8298	0.8368	0.8407	0.8392	0.8073	0.2703
	ASN SSR	247 (25)	202 (42)	197 (54)	199 (65)	205 (79)	215 (98)	230 (125)	257 (168)	308 (240)	425 (354)	1170 (182)
**Both**	Power SSR	0.7793	0.8167	0.8282	0.8383	0.8511	0.8629	0.8713	0.8784	0.8795	0.869	0.4836
	ASN SSR	296 (34)	246 (47)	235 (61)	231 (74)	232 (88)	239 (106)	251 (132)	274 (172)	319 (238)	430 (345)	1170 (182)
**Short**	Power SSR	0.7822	0.7678	0.7624	0.7621	0.7678	0.7743	0.7777	0.7775	0.7721	0.759	0.4849
	ASN SSR	296 (34)	226 (40)	208 (48)	199 (53)	195 (58)	195 (65)	198 (75)	207 (93)	230 (130)	316 (246)	1170 (182)

Table 3: Operating characteristics (probability to stop for futility (FS), overall power and average sample size (ASN) and its standard deviation) of a trial with sample size reassessment based on fixed effect conditional power with p-value futility stopping rule based on p-vales of Z_L, Z_S and Z_B for a different choice of weights for the combination test and sample size reassessment.
