# Supplementary material for: Interim analysis incorporating short‐ and long‐term binary endpoints
Source: Biom J. 2019 Jan 29;61(3):665–87. doi: 10.1002/bimj.201700281 (PMC6590444; doi:10.1002/bimj.201700281)
Supplement: Supplementary file 1 — Supporting Information [file BIMJ-61-665-s001.zip › Reproducibility_Julia_Niewczas/Suppl.pdf]

Supplementary Materials

Interim analysis incorporating short- and long-term binary endpoints

_Julia Niewczas and Franz König_*
Center for Medical Statistics, Informatics, and Intelligent Systems, Medical University of Vienna, Spitalgasse 23, 1090 Vienna, Austria
Cornelia Ursula Kunz
Department of Mathematics and Statistics, Lancaster University Lancaster LA1 4YF, UK

*franz.koenig@meduniwien.ac.at; Corresponding author

1 Mathematical Formulae

We compare three ways of estimating the response rate at the interim analysis:

- use of long-term data only, $\hat{P}_{\mathbf{L}}^{(1)}$,
- use of short-term data only, $\hat{P}_{\mathbf{S}}^{(1)}$,
- or combination of both, $\hat{P}_{\mathbf{B}}^{(1)}$.

There are n_{L_i} patients in each treatment group at interim for the primary endpoint, and $n_{S_i}(\geq n_{L_i})$ patients in each treatment group for the secondary endpoint. @WatsonCrick1953Adjusted significance levels for subgroup analyses in clinical trials

1.1 Information Fraction of $\hat{P}_{\mathbf{L}}^{(1)}$

The information fraction of $\hat{P}_{\mathbf{L}}^{(1)}$ is given by:

$$\begin{aligned} t_L &= \frac{\frac{\bar{P}_L^{(1)}(1-\bar{P}_L^{(1)})}{N_C} + \frac{\bar{P}_L^{(1)}(1-\bar{P}_L^{(1)})}{N_E}}{\frac{\bar{P}_L^{(1)}(1-\bar{P}_L^{(1)})}{n_{L_E}} + \frac{\bar{P}_L^{(1)}(1-\bar{P}_L^{(1)})}{n_{L_C}}} \\ &= \frac{\bar{P}_L^{(1)}(1-\bar{P}_L^{(1)})\left(\frac{1}{N_C}+\frac{1}{N_E}\right)}{\bar{P}_L^{(1)}(1-\bar{P}_L^{(1)})\left(\frac{1}{n_{L_C}}+\frac{1}{n_{L_E}}\right)} \\ &= \frac{\frac{1}{N_C}+\frac{1}{N_E}}{\frac{1}{n_{L_C}}+\frac{1}{n_{L_E}}} \end{aligned}$$

where $\bar{P}_L^{(1)} = \frac{\hat{P}_{L_E}^{(1)}+\hat{P}_{L_C}^{(1)}}{2}$ (under the assumption of null hypothesis). This simplifies to n_L/N when design is balanced, that is when $n_{L_E} = n_{L_C}$ and $N_E = N_C$.

1.2 Z-Statistic and t_S for $\hat{P}_{S_i}^{(1)}$

The Z-statistic and information fraction t_S for $\hat{P}_{S_i}^{(1)}$ can be obtained:

$$Z_S^{(1)} = \frac{\hat{P}_{S_E}^{(1)} - \hat{P}_{S_C}^{(1)}}{\sqrt{\bar{P}_S^{(1)}(1-\bar{P}_S^{(1)})\left(\frac{1}{n_{S_E}}+\frac{1}{n_{S_C}}\right)}},$$

where $\bar{P}_S^{(1)} = \frac{\hat{P}_{S_C}^{(1)}+\hat{P}_{S_E}^{(1)}}{2}$. The information fraction is equal to

$$t_S = \frac{1/N_E + 1/N_C}{1/n_{S_E} + 1/n_{S_C}}.$$

Again, if $n_{S_E} = n_{S_C}$ and $N_E = N_C$, the formula simplifies to n_S/N.

1.3 Estimator Incorporating Both S and L

Estimator that combines information from both S and L is derived from three-binomial distributions (Marschner and Becker, 2001) of $P_{S_i}^{(1)}$, $P_{L_i}^{(1)}$ and $P_{SL_i}^{(1)} = Pr(L_i = 1|S_i = 1)$. Consider patients for whom L has been observed and define n_{SL_i} to be the number of patients for whom $S_i = 1$ and similarly s_{SL_i} to be the number of patients for whom $S_i = 0$. Then, define m_{SL_i} and r_{SL_i} to be the number of subjects for whom $(L_i = 1, S_i = 1)$ and $(L_i = 1, S_i = 0)$ respectively. As

$Pr(L_i = 1|S_i = 0) = \frac{P_{L_i}^{(1)} - P_{SL_i}^{(1)} P_{S_i}^{(1)}}{1 - P_{S_i}^{(1)}}$, the likelihood function is given by Marschner and Becker (2001):

$$L(P_{L_i}^{(1)}, P_{S_i}^{(1)}, P_{SL_i}^{(1)} | \mathbf{L_i}, \mathbf{S_i}) = (P_{S_i}^{(1)})^{m_{S_i}} (1 - P_{S_i}^{(1)})^{n_{S_i} - m_{S_i}} \left(\frac{P_{L_i}^{(1)} - P_{S_i}^{(1)} P_{SL_i}^{(1)}}{1 - P_{S_i}^{(1)}} \right)^{r_{SL_i}}$$
$$\times \left(\frac{1 - P_{L_i}^{(1)} - (1 - P_{SL_i}^{(1)}) P_{S_i}^{(1)}}{1 - P_{S_i}^{(1)}} \right)^{s_{SL_i} - r_{SL_i}} (P_{SL_i}^{(1)})^{m_{SL_i}} (1 - P_{SL_i}^{(1)})^{n_{SL_i} - m_{SL_i}},$$

with corresponding observations of $(m_{S_i}, r_{SL_i}, m_{SL_i})$. In case of $\hat{P}_{\mathbf{B}}^{(1)}$, the variance is obtained from the asymptotic distribution of the likelihood function (see Marschner and Becker (2001)) and it can be simplified to the following form:

$$\text{Var}(\hat{P}_{B_i}^{(1)}) = \frac{1}{n_{L_i}} \hat{P}_{B_i}^{(1)} \left(1 - \hat{P}_{B_i}^{(1)}\right) \left(1 - \hat{\phi}_i^{\,2} \times \left(1 - \frac{n_{L_i}}{n_{S_i}}\right)\right),$$

where $\hat{\phi}_i$ is the estimate of the correlation (Phi coefficient) between S and L (defined by Cramér (1946)):

$$\hat{\phi}_i = \frac{\hat{P}_{SL_i}^{(1)} \hat{P}_{S_i}^{(1)} - \hat{P}_{B_i}^{(1)} \hat{P}_{S_i}^{(1)}}{\sqrt{\hat{P}_{B_i}^{(1)} (1 - \hat{P}_{B_i}^{(1)}) \hat{P}_{S_i}^{(1)} (1 - \hat{P}_{S_i}^{(1)})}}.$$

1.4 Information Fraction For $\hat{P}_{\mathbf{B}}^{(1)}$

Using methodology of Proschan et al. (2006) and Stallard (2010) for balanced design:

$$\begin{aligned}
t_B &= \frac{\frac{\bar{P}_B^{(1)}(1-\bar{P}_B^{(1)})}{N_E} + \frac{\bar{P}_B^{(1)}(1-\bar{P}_B^{(1)})}{N_C}}{\frac{\bar{P}_B^{(1)}(1-\bar{P}_B^{(1)})}{n_{L_E}} \left(1 - \hat{\phi}_E^2 \left(1 - \frac{n_{L_E}}{n_{S_E}}\right)\right) + \frac{\bar{P}_B^{(1)}(1-\bar{P}_B^{(1)})}{n_{L_C}} \left(1 - \hat{\phi}_C^2 \left(1 - \frac{n_{L_C}}{n_{S_C}}\right)\right)} \\
&= \frac{\bar{P}_B^{(1)}(1 - \bar{P}_B^{(1)}) \left(\frac{1}{N_C} + \frac{1}{N_E}\right)}{\bar{P}_B^{(1)}(1 - \bar{P}_B^{(1)}) \left(\frac{1 - \hat{\phi}_E^2 \left(1 - \frac{n_{L_E}}{n_{S_E}}\right)}{n_{L_E}} + \frac{1 - \hat{\phi}_C^2 \left(1 - \frac{n_{L_C}}{n_{S_C}}\right)}{n_{L_C}} \right)} \\
&= \frac{\frac{1}{N_C} + \frac{1}{N_E}}{\frac{1 - \hat{\phi}_E^2 \left(1 - \frac{n_{L_E}}{n_{S_E}}\right)}{n_{L_E}} + \frac{1 - \hat{\phi}_C^2 \left(1 - \frac{n_{L_C}}{n_{S_C}}\right)}{n_{L_C}}},
\end{aligned}$$

where $\bar{P}_B^{(1)} = \frac{\hat{P}_{B_E}^{(1)} + \hat{P}_{B_C}^{(1)}}{2}$. If we assume that $N_E = N_C = N$, $n_{L_E} = n_{L_C} = n_L$ and $n_{S_E} = n_{S_C} = n_S$, the above simplifies to

$$\begin{aligned}
t_B &= \frac{\frac{1}{N} + \frac{1}{N}}{\frac{1 - \hat{\phi}_E^2 \left(1 - \frac{n_L}{n_S}\right)}{n_L} + \frac{1 - \hat{\phi}_C^2 \left(1 - \frac{n_L}{n_S}\right)}{n_L}} \\
&= \frac{\frac{2}{N}}{\frac{1}{n_L} \left(1 - \hat{\phi}_E^2 \left(1 - \frac{n_L}{n_S}\right) + 1 - \hat{\phi}_C^2 \left(1 - \frac{n_L}{n_S}\right)\right)} \\
&= \frac{2n_L}{N \left(2 - \left(1 - \frac{n_L}{n_S}\right) (\hat{\phi}_E^2 + \hat{\phi}_C^2)\right)}.
\end{aligned}$$

1.5 Conditional Power

The conditional power for is obtained as follows. We know that the test statistic at the end of the trial can be written as:

$$Z_i = \sqrt{t_i} Z_i^{(1)} + \sqrt{1 - t_i} Z_i^{(2)},$$

where $i = \{L, S, B\}$ corresponds to a given estimator. We assume that sample sizes in experimental and control treatment groups are equal so that $N_E = N_C = N$. We want to calculate the probability:

$$
\begin{aligned}
Pr(Z_i \geq z_{1-\alpha}|Z_i^{(1)}) = Pr(\sqrt{t_i}Z_i^{(1)} + \sqrt{1-t_i}Z_i^{(2)}|Z_i^{(1)}) \\
=Pr\left(Z_i^{(2)} \geq \frac{z_{1-\alpha} - \sqrt{t_i}Z_i^{(1)}}{\sqrt{1-t_i}}\right).
\end{aligned}
$$

Now, if the effect for the second stage data is assumed to be equal to the one from the planning stage (fixed effect), we obtain conditional power:

$$
CP_{\theta_D} = 1 - \Phi\left(\frac{z_{1-\alpha} - \sqrt{t_i}Z_i^{(1)}}{\sqrt{1-t_i}} - \frac{P_{L_E} - P_{L_C}}{\sqrt{2\bar{P}_L(1-\bar{P}_L)\frac{2}{n_{i_2}}}}\right),
$$

where n_{i_2} corresponds to the second stage sample size for a given estimator such that $n_{i_2} = n_{i_{C_2}} = n_{i_{E_2}}$.

For the fixed effect we assume the effect, $P_{L_E} - P_{L_C} = \Delta$, from the planning stage, which is equal to

$$
\Delta = \frac{(z_{1-\alpha} + z_{1-\beta})\sqrt{2\bar{P}_L(1-\bar{P}_L)}}{\sqrt{N}}
$$

which comes from the sample size formula for 2 proportions. This can be then substituted into the conditional power equation:

$$
\begin{aligned}
CP_{\theta_D} =& 1 - \Phi\left(\frac{z_{1-\alpha} - \sqrt{t_i}Z_i^{(1)}}{\sqrt{1-t_i}} - \frac{P_{L_E} - P_{L_C}}{\sqrt{\bar{P}_L(1-\bar{P}_L))\frac{2}{n_{i_2}}}}\right) \\
=& 1 - \Phi\left(\frac{z_{1-\alpha} - \sqrt{t_i}Z_i^{(1)}}{\sqrt{1-t_i}} - \frac{(z_{1-\alpha} + z_{1-\beta})\sqrt{2\bar{P}_L(1-\bar{P}_L)}}{\sqrt{N}} \times \frac{1}{\sqrt{2\bar{P}_L(1-\bar{P}_L)\frac{2}{n_{i_2}}}}\right) \\
=& 1 - \Phi\left(\frac{z_{1-\alpha} - \sqrt{t_i}Z_i^{(1)}}{\sqrt{1-t_i}} - \frac{(z_{1-\alpha} + z_{1-\beta})\sqrt{n_{i_2}}}{\sqrt{N}}\right).
\end{aligned}
$$

We assume for all estimators that $n_{i_2}/N = 1 - t_i$. We therefore obtain:

$$
CP_{\theta_D} = 1 - \Phi\left(\frac{z_{1-\alpha} - \sqrt{t_i}Z_i^{(1)}}{\sqrt{1-t_i}} - (z_{1-\alpha} + z_{1-\beta})\sqrt{1-t_i}\right).
$$

For the observed effect instead of plugging in Δ from the planning stage, we plug in the effect from a Z-statistic obtained at interim. At first let us consider the case for $\hat{P}_{\mathbf{L}}^{(1)}$ and $\hat{P}_{\mathbf{S}}^{(1)}$ (assuming equal sample sizes in both treatment groups so that $n_{E_k} = n_{C_k} = n_k$:

$$
Z_k^{(1)} = \frac{\hat{P}_{k_E}^{(1)} - \hat{P}_{k_C}^{(1)}}{\sqrt{\bar{P}_k^{(1)}(1-\bar{P}_k^{(1)})\frac{2}{n_k}}},
$$

where $k = \{L, S\}$ so that

$$
\hat{P}_{k_E}^{(1)} - \hat{P}_{k_C}^{(1)} = Z_k^{(1)}\sqrt{\bar{P}_k^{(1)}(1-\bar{P}_k^{(1)})\frac{2}{n_k}},
$$

where n_k ($k = \{L, S\}$ is the first stage sample size and can be equal to n_L or n_S. This can be again substituted into the conditional power equation:

$$
\begin{aligned}
CP_{\hat{\theta}} =& 1 - \Phi\left(\frac{z_{1-\alpha} - \sqrt{t_k}Z_k^{(1)}}{\sqrt{1-t_k}} - \frac{\hat{P}_{k_E}^{(1)} - \hat{P}_{k_C}^{(1)}}{\sqrt{\bar{P}_k^{(1)}(1-\bar{P}_k^{(1)})\frac{2}{n_{k_2}}}}\right) \\
=& 1 - \Phi\left(\frac{z_{1-\alpha} - \sqrt{t_k}Z_k^{(1)}}{\sqrt{1-t_i}} - \frac{Z_k^{(1)}\sqrt{2\bar{P}_k^{(1)}(1-\bar{P}_k^{(1)})}}{\sqrt{n_k}} \times \frac{\sqrt{n_{k_2}}}{\sqrt{2\bar{P}_k^{(1)}(1-\bar{P}_k^{(1)})}}\right).
\end{aligned}
$$

As $n_{k_2} = (1-t_k)N$ and $n_k = t_k N$, we can substitute the values into the conditional power equation:

$$
\begin{aligned}
CP_{\hat{\theta}} =& 1 - \Phi\left(\frac{z_{1-\alpha} - \sqrt{t_k} Z_k^{(1)}}{\sqrt{1-t_k}} - \frac{Z_k^{(1)}\sqrt{n_{k_2}}}{\sqrt{n_k}}\right) \\
=& 1 - \Phi\left(\frac{z_{1-\alpha} - \sqrt{t_k} Z_k^{(1)}}{\sqrt{1-t_k}} - \frac{Z_k^{(1)}\sqrt{(1-t_k)}}{\sqrt{t_k}}\right) \\
=& 1 - \Phi\left(\frac{z_{1-\alpha} - Z_k^{(1)}/\sqrt{t_k}}{\sqrt{1-t_k}}\right).
\end{aligned}
$$

Similarly, we have for $\hat{P}_{\mathbf{B}}^{(1)}$. The Z-statistic for the estimator is equal to (again assuming equal sample sizes in experimental and treatment groups):

$$
\begin{aligned}
Z_B^{(1)} &= \frac{\hat{P}_{B_E}^{(1)} - \hat{P}_{B_C}^{(1)}}{\sqrt{\bar{P}_B^{(1)}(1-\bar{P}_B^{(1)})\left(\frac{1-\hat{\phi_E}^2 \times \left(1-\frac{n_L}{n_S}\right)}{n_L} + \frac{1-\hat{\phi_C}^2 \times \left(1-\frac{n_L}{n_S}\right)}{n_L}\right)}} \\
&= \frac{\hat{P}_{B_E}^{(1)} - \hat{P}_{B_C}^{(1)}}{\sqrt{\bar{P}_B^{(1)}(1-\bar{P}_B^{(1)})\left(\frac{2-(\hat{\phi_E}^2 + \hat{\phi_C}^2) \times \left(1-\frac{n_L}{n_S}\right)}{n_L}\right)}}.
\end{aligned}
$$

We know that the information fraction for $\hat{P}_{\mathbf{B}}^{(1)}$ is:

$$
t_B = \frac{2n_L}{N\left(2 - \left(1 - \frac{n_L}{n_S}\right)(\hat{\phi}_E^2 + \hat{\phi}_C^2)\right)},
$$

so we can rewrite the equation for the Z-statistic to be:

$$
Z_B^{(1)} = \frac{\hat{P}_{B_E}^{(1)} - \hat{P}_{B_C}^{(1)}}{\sqrt{\bar{P}_B^{(1)}(1-\bar{P}_B^{(1)})\frac{2}{t_B N}}}.
$$

This can be then plugged into the conditional power equation:

$$
\begin{aligned}
CP_{\hat{\theta}} =& 1 - \Phi\left(\frac{z_{1-\alpha} - \sqrt{t_B} Z_B^{(1)}}{\sqrt{1-t_B}} - \frac{Z_B^{(1)}\sqrt{2\bar{P}_B^{(1)}(1-\bar{P}_B^{(1)})}}{\sqrt{t_B N}} \times \frac{\sqrt{n_{B_2}}}{\sqrt{2\bar{P}_B^{(1)}(1-\bar{P}_B^{(1)})}}\right) \\
=& 1 - \Phi\left(\frac{z_{1-\alpha} - \sqrt{t_B} Z_B^{(1)}}{\sqrt{1-t_B}} - \frac{Z_B^{(1)}\sqrt{(1-t_B)N}}{\sqrt{t_B N}}\right) \\
=& 1 - \Phi\left(\frac{z_{1-\alpha} - \sqrt{t_B} Z_B^{(1)}}{\sqrt{1-t_B}} - \frac{Z_B^{(1)}\sqrt{(1-t_B)}}{\sqrt{t_B}}\right) \\
=& 1 - \Phi\left(\frac{z_{1-\alpha} - Z_B^{(1)}/\sqrt{t_B}}{\sqrt{1-t_B}}\right),
\end{aligned}
$$

as in the above example for $\hat{P}_{\mathbf{S}}^{(1)}$ and $\hat{P}_{\mathbf{L}}^{(1)}$.

1.6 Cut-off Point Equivalence

The equivalent cut off-points can be found by equalising the Z-statistics and solving for a chosen c. Let $Z^{(1)}$ be the Z-statistic calculated at interim, $\theta_D = (z_{1-\alpha} + z_{1-\beta})$ and $z_{1-\alpha}$ is the $(1-\alpha)$ quantile of the standard normal distribution.

We have the following for formula rearrangement for the fixed effect:

$$\begin{aligned}
c_{\theta_D} &= 1-\Phi\left(\frac{z_{1-\alpha}-Z^{(1)}\times\sqrt{t}-(z_{1-\alpha}+z_{1-\beta})(1-t)}{\sqrt{1-t}}\right)\\
\iff \quad \Phi^{-1}(1-c_{\theta_D}) &= \frac{z_{1-\alpha}-Z^{(1)}\times\sqrt{t}-(z_{1-\alpha}+z_{1-\beta})(1-t)}{\sqrt{1-t}}\\
\iff \quad \Phi^{-1}(1-c_{\theta_D})\sqrt{1-t} &= z_{1-\alpha}-Z^{(1)}\times\sqrt{t}-(z_{1-\alpha}+z_{1-\beta})(1-t)\\
\iff \quad Z^{(1)}\times\sqrt{t} &= z_{1-\alpha}-(z_{1-\alpha}+z_{1-\beta})(1-t)-\Phi^{-1}(1-c_{\theta_D})\sqrt{1-t}\\
\iff \quad Z^{(1)} &= \frac{z_{1-\alpha}-(z_{1-\alpha}+z_{1-\beta})(1-t)-\Phi^{-1}(1-c_{\theta_D})\sqrt{1-t}}{\sqrt{t}}.
\end{aligned}$$

Similarly, for the observed effect power approach, the Z-statistic can be obtained:

$$\begin{aligned}
c_{\hat{\theta}} &= 1-\Phi\left(\frac{z_{1-\alpha}-Z^{(1)}/\sqrt{t}}{\sqrt{1-t}}\right)\\
\iff \quad \Phi^{-1}(1-c_{\hat{\theta}}) &= \frac{z_{1-\alpha}-Z^{(1)}/\sqrt{t}}{\sqrt{1-t}}\\
\iff \quad \Phi^{-1}(1-c_{\hat{\theta}})\sqrt{1-t} &= z_{1-\alpha}-Z^{(1)}/\sqrt{t}\\
\iff \quad Z^{(1)}/\sqrt{t} &= z_{1-\alpha}-\Phi^{-1}(1-c_{\hat{\theta}})\sqrt{1-t}\\
\iff \quad Z^{(1)} &= \left(z_{1-\alpha}-\Phi^{-1}(1-c_{\hat{\theta}})\sqrt{1-t}\right)\sqrt{t}.
\end{aligned}$$

Now by equalising for $Z^{(1)}$, we can find an equivalent cut-off point for alternative hypothesis conditional power given the fixed effect:

$$\begin{aligned}
\left(z_{1-\alpha}-\Phi^{-1}(1-c_{\hat{\theta}})\sqrt{1-t}\right)\sqrt{t} &= \frac{z_{1-\alpha}-\Phi^{-1}(1-c_{\theta_D})\sqrt{1-t}-(z_{1-\alpha}+z_{1-\beta})(1-t)}{\sqrt{t}}\\
\iff \quad \left(z_{1-\alpha}-\Phi^{-1}(1-c_{\hat{\theta}})\sqrt{1-t}\right)t &= z_{1-\alpha}-\Phi^{-1}(1-c_{\theta_D})\sqrt{1-t}-(z_{1-\alpha}+z_{1-\beta})(1-t)\\
\iff \quad \Phi^{-1}(1-c_{\theta_D})\sqrt{1-t} &= z_{1-\alpha}-(z_{1-\alpha}+z_{1-\beta})(1-t)-\left(z_{1-\alpha}-\Phi^{-1}(1-c_{\hat{\theta}})\sqrt{1-t}\right)t\\
\iff \quad 1-c_{\theta_D} &= \Phi\left(\frac{z_{1-\alpha}-(z_{1-\alpha}+z_{1-\beta})(1-t)-\left(z_{1-\alpha}-\Phi^{-1}(1-c_{\hat{\theta}})\sqrt{1-t}\right)t}{\sqrt{1-t}}\right)\\
\iff \quad c_{\theta_D} &= 1-\Phi\left(\frac{z_{1-\alpha}-(z_{1-\alpha}+z_{1-\beta})(1-t)-\left(z_{1-\alpha}-\Phi^{-1}(1-c_{\hat{\theta}})\sqrt{1-t}\right)t}{\sqrt{1-t}}\right),
\end{aligned}$$

and similarly for observed effect given the fixed effect approach:

$$\begin{aligned}
\left(z_{1-\alpha}-\Phi^{-1}(1-c_{\hat{\theta}})\sqrt{1-t}\right)\sqrt{t} &= \frac{z_{1-\alpha}-\Phi^{-1}(1-c_{\theta_D})\sqrt{1-t}-(z_{1-\alpha}+z_{1-\beta})(1-t)}{\sqrt{t}}\\
\iff \quad \left(z_{1-\alpha}-\Phi^{-1}(1-c_{\hat{\theta}})\sqrt{1-t}\right) &= \frac{z_{1-\alpha}-\Phi^{-1}(1-c_{\theta_D})\sqrt{1-t}-(z_{1-\alpha}+z_{1-\beta})(1-t)}{t}\\
\iff \quad \Phi^{-1}(1-c_{\hat{\theta}})\sqrt{1-t} &= z_{1-\alpha}-\frac{z_{1-\alpha}-\Phi^{-1}(1-c_{\theta_D})\sqrt{1-t}-(z_{1-\alpha}+z_{1-\beta})(1-t)}{t}\\
\iff \quad \Phi^{-1}(1-c_{\hat{\theta}}) &= \frac{z_{1-\alpha}}{\sqrt{1-t}}-\frac{z_{1-\alpha}-\Phi^{-1}(1-c_{\theta_D})\sqrt{1-t}-(z_{1-\alpha}+z_{1-\beta})(1-t)}{t\sqrt{1-t}}\\
\iff \quad c_{\hat{\theta}} &= 1-\Phi\left(\frac{z_{1-\alpha}}{\sqrt{1-t}}-\frac{z_{1-\alpha}-\Phi^{-1}(1-c_{\theta_D})\sqrt{1-t}-(z_{1-\alpha}+z_{1-\beta})(1-t)}{t\sqrt{1-t}}\right).
\end{aligned}$$

1.7 Second Stage Sample Size Derivation

For sample size reassessment formula, we use similar methodology as for obtaining conditional power. Here, however, the assumption on the second stage data will be written in terms of the second stage sample size. As the combination test assumes independence of data from the two stages, the first stage sample size for $\hat{P}_{\mathbf{B}}^{(1)}$ will be assumed to be equal to n_S. What is more, the final Z-statistic is constructed using pre-specified weights of the combination test, so that:

$$Z = \sqrt{w}Z^{(1)} + \sqrt{1-w}Z^{(2)}.$$

In such a case the weights can be chosen arbitrarily as long as $0 \leq w \leq 1$. Let n_2 correspond to second-stage sample size. For the fixed effect we have:

$$CP_{\theta_D} = 1 - \Phi\left(\frac{z_{1-\alpha} - \sqrt{w}Z^{(1)}}{\sqrt{1-w}} - \frac{(z_{1-\alpha} + z_{1-\beta})\sqrt{n_2}}{\sqrt{N}}\right).$$

Now, rewrite the second stage sample size to be equal to $\tilde{N} - n_k$, which corresponds to the adapted total sample size and the first stage sample size and $k = \{S, L\}$. The conditional power equations will be set to be equal to $1 - \beta$, i.e. the power from the planning stage, and then rearranged in order to be solved for the second stage sample size, $\tilde{N} - n_k$:

$$\begin{aligned}
1 - \beta &= 1 - \Phi\left(\frac{z_{1-\alpha} - \sqrt{w}Z^{(1)}}{\sqrt{1-w}} - \frac{(z_{1-\alpha} + z_{1-\beta})\sqrt{\tilde{N} - n_k}}{\sqrt{N}}\right) \\
\iff \quad \Phi^{-1}(\beta) &= \frac{z_{1-\alpha} - \sqrt{w}Z^{(1)}}{\sqrt{1-w}} - \frac{(z_{1-\alpha} + z_{1-\beta})}{\sqrt{N}}\sqrt{\tilde{N} - n_k} \\
\iff \quad \sqrt{\tilde{N} - n_k} &= \frac{\frac{z_{1-\alpha} - \sqrt{w}Z^{(1)}}{\sqrt{1-w}} - \Phi^{-1}(\beta)}{(z_{1-\alpha} + z_{1-\beta})/\sqrt{N}} \\
\iff \quad \tilde{N} - n_k &= \left(\frac{\frac{z_{1-\alpha} - \sqrt{w}Z^{(1)}}{\sqrt{1-w}} - \Phi^{-1}(\beta)}{(z_{1-\alpha} + z_{1-\beta})/\sqrt{N}}\right)^2
\end{aligned}$$

For the observed effect we follow the same steps. Similarly, let $\tilde{N} - n_k$ denote the adapted second stage sample size:

$$CP_{\hat{\theta}} = 1 - \Phi\left(\frac{z_{1-\alpha} - \sqrt{w}Z^{(1)}}{\sqrt{1-w}} - \frac{Z^{(1)}\sqrt{\tilde{N} - n_k}}{\sqrt{tN}}\right),$$

Now, again rearranging for $\tilde{n}_2$ we obtain:

$$\begin{aligned}
1 - \beta &= 1 - \Phi\left(\frac{z_{1-\alpha} - \sqrt{w}Z^{(1)}}{\sqrt{1-w}} - \frac{Z^{(1)}\sqrt{\tilde{N} - n_k}}{\sqrt{tN}}\right) \\
\Phi^{-1}(\beta) &= \frac{z_{1-\alpha} - \sqrt{w}Z^{(1)}}{\sqrt{1-w}} - \frac{Z^{(1)}}{\sqrt{tN}}\sqrt{\tilde{N} - n_k} \\
\sqrt{\tilde{N} - n_k} &= \frac{\frac{z_{1-\alpha} - \sqrt{w}Z^{(1)}}{\sqrt{1-w}} - \Phi^{-1}(\beta)}{Z^{(1)}/\sqrt{tN}} \\
\tilde{N} - n_k &= \left(\frac{\frac{z_{1-\alpha} - \sqrt{w}Z^{(1)}}{\sqrt{1-w}} - \Phi^{-1}(\beta)}{Z^{(1)}/\sqrt{tN}}\right)^2.
\end{aligned}$$

Note that for each approach, t corresponds to the information fraction of a given estimator. $Z^{(1)}$ corresponds to the Z-statistic calculated at interim and can correspond to a Z-statistic for any estimator. t and $Z^{(1)}$ should however correspond to the information fraction and a Z-statistic from use of the same approach.

2 Conditional Probabilities For Interim Analyses with Futility Stopping Only

In this Section we report the conditional probabilities given the interim decision in the trial. These include the probability to reject the null hypothesis at the end of the trial given it was continued, the probability that we would have failed to reject the null hypothesis for the trials that were stopped for futility, and the probability of making the correct decision, i.e. rejecting the trial given it was continued and failed to reject the trial would have been continued (for the trials that were stopped for futility).

In the first subsection we present results from the case presented in the main body of the article with the following operating characteristics of the trial: one-sided $\alpha = 0.025$, $1 - \beta = 0.8$, $t_L = 0.25$, $t_S = 0.5$, $N = 200$, $P_{L_C} = P_{S_C} = 0.2$, $P_{L_E} \approx 0.323$, $\phi_E = \phi_C = 0.5$. We considered four types of outcomes for the short-term outcome in the experimental treatment group:

no effect, moderate effect, effect equal to long-term outcome, and larger effect than for the long-term outcome with the following probabilities: $(0.2, 0.285, 0.323, 0.365)$.

In the next subsection results for $\phi_E = \phi_C = 0.2$ and $\phi_E = \phi_C = 0.65$ are presented. The figures show the power, probability to stop for futility, the probability of rejecting the null hypothesis at the end of the trial given it was continued, the probability that we would have failed to reject the null hypothesis for the trials that were stopped for futility probability of making the correct decision.

For the remainder of the supplementary materials, the code for reproducing the output using package `binfutssr` will be also provided. The package consists of a set of functions which calculate the operating characteristics of simulated clinical trials.

In order to create the first plot, load the package `binfutssr` (with its dependencies) and type in the following code. One scenario of 100,000 simulations takes approximately 2 min for interim analysis with futility stopping and 2.5 min for sample size reassessments. If one has access to multiple cores, the packages `parallel` and `doParallel` can be loaded and the number of cores can be specified using function `cl <- makeCluster(m)` with m coresponding to the number of cores. The cores can be registered using `registerDoParallel(cl)`. The code for provision of graphics uses the `foreach` function which if more than 1 core is specified parallises the procedure.

At first, fix the setting for trial simulation

```
library(psych)
library(ggplot2)
```

```
##
## Attaching package: 'ggplot2'
## The following objects are masked from 'package:psych':
##
##     %+%, alpha
```

```
library(foreach)
library(mvtnorm)
library(stringr)
library(gridExtra)
library(parallel)
library(doParallel)
```

```
## Loading required package: iterators
```

```
library(xtable)
library(knitr)
library(kableExtra)
library(binfutssr) # need to install this locally from .tar.gz file
```

```
## Loading required package: grid
```

2.1 Conditional Probabilities for $\phi_E = \phi_C = 0.5$

```
p_se <- c(0.2, 0.285, 0.3227348, 0.365)

m <- length(p_se) # if multiple cores are available set this value to length of p_se
cl <- parallel::makeCluster(m)
doParallel::registerDoParallel(cl)

output_cp_alt <- foreach(i = 1:length(p_se), .packages="binfutssr") %dopar%
  (cp(nsim = 100000,        #number of simulations
      alpha = 0.025,         # one-sided alpha
      beta = 0.2,            # type 2 error such that 1-beta is the power
      p_le = 0.3227348,      # probability of success for long-term endpoint in E
      p_lc = 0.2,            # probability of success for long-term endpoint in C
      p_se = p_se[i],        # i-th probability of success for short-term endpoint in E
```

```
      p_sc = 0.2,            # probability of success for short-term endpoint in C
      n = 200,               # sample size per treatment arm
      fr_lo = 0.25,          # amounut of long-term information available at interim
      fr_sh = 0.5,           # amounut of short-term information available at interim
      phi_e = 0.5,           # correlation in E
      phi_c = 0.5,           # correlation in C
      c = seq(0, 1, 0.01)))  #sequence of cut-off points for stopping based on cp


#Simulate clinical trial for moderate power (~50%)



output_cp_mod <- foreach(i = 1:length(p_se), .packages="binfutssr") %dopar%
  (cp(nsim = 100000,
      alpha = 0.025,
      beta = 0.2,
      p_le = 0.285,
      p_lc = 0.2,
      p_se = p_se[i],
      p_sc = 0.2,
      n = 200,
      fr_lo = 0.25,
      fr_sh = 0.5,
      phi_e = 0.5,
      phi_c = 0.5,
      c = seq(0, 1, 0.01)))


#Simulate clinical trial under the null hypothesis


output_cp_null <- foreach(i = 1:length(p_se), .packages="binfutssr") %dopar%
  (cp(nsim = 100000,
      alpha = 0.025,
      beta = 0.2,
      p_le = 0.2,
      p_lc = 0.2,
      p_se = p_se[i],
      p_sc = 0.2,
      n = 200,
      fr_lo = 0.25,
      fr_sh = 0.5,
      phi_e = 0.5,
      phi_c = 0.5,
      c = seq(0, 1, 0.01)))
```

2.1.1 Probability to Reject the Null Hypothesis Given Trial Was Continued

```
prob_cont_rej_plots <- create_prob_cont_rej_plots(
  output_cp_alt = output_cp_alt,
  output_cp_mod = output_cp_mod,
  output_cp_null = output_cp_null,
  phi_e = 0.5,
  phi_c = 0.5,
  filetype = "none")
```

```r
grid.arrange(prob_cont_rej_plots)
```

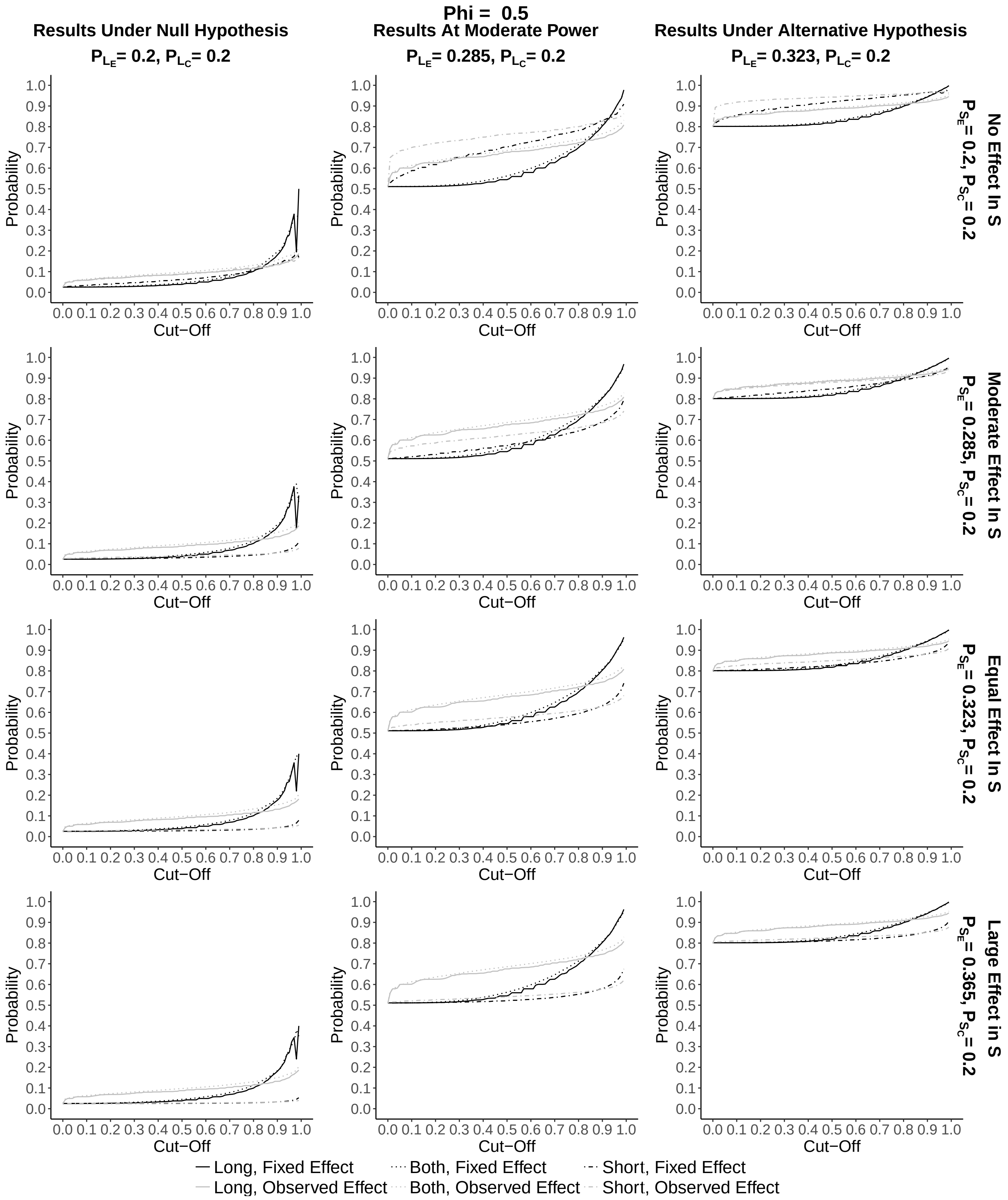

Figure 1: Plots showing the probability to reject the null hypothesis given the trial was continued plotted against cut-off points for different effect sizes in P_{S_E} for $\phi_E = \phi_C = 0.5$. First column corresponds to the results under the null hypothesis, middle column to the simulations at moderate power and right to the simulations under alternative hypothesis. The rows correspond to no effect, moderate effect, effect equal to the one P_{L_E} under the alternative hypothesis, and a higher effect than for P_{L_E} respectively. Grey lines correspond to observed effect conditional power, $CP_{\hat{\theta}}$, whereas black to fixed effect conditional power, CP_{θ_D}. $\hat{P}^{(1)}_{\mathbf{B}}$ is denoted by dotted lines, $\hat{P}^{(1)}_{\mathbf{L}}$ by solid and $\hat{P}^{(1)}_{\mathbf{S}}$ by dot-dashed.

2.1.2 Probability to Fail to Reject the Null Hypothesis Given The Trial Was Stopped Had it Been Continued

```
prob_stop_not_rej_plots <- create_prob_stop_not_rej_plots(
  output_cp_alt = output_cp_alt,
  output_cp_mod = output_cp_mod,
  output_cp_null = output_cp_null,
  phi_e = 0.5,
  phi_c = 0.5,
  filetype = "none")
```

```
grid.arrange(prob_stop_not_rej_plots)
```

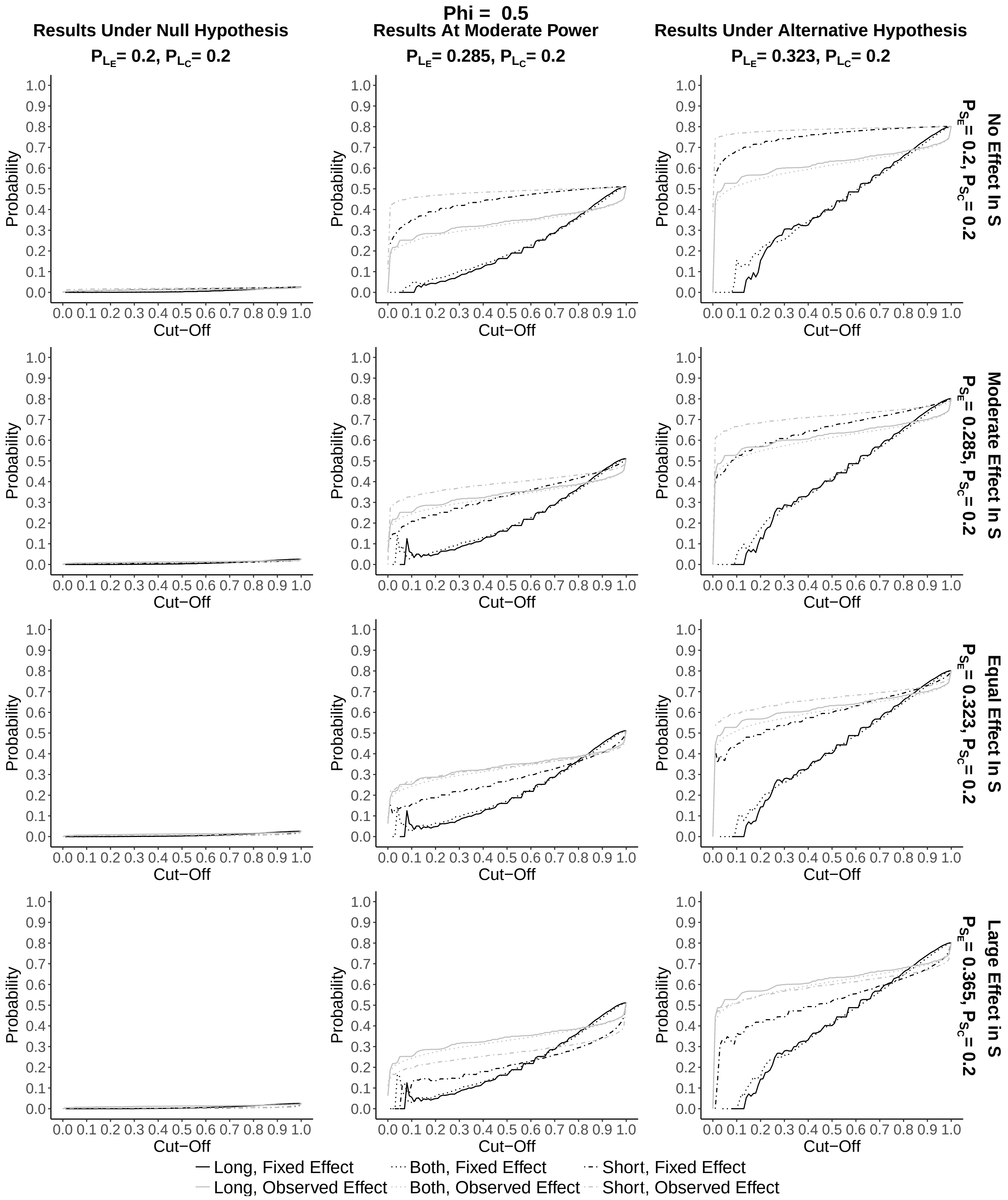

Figure 2: Plots showing the probability of not having rejected the null hypothesis, if the trial had been continued for the cases where the interim decision was to stop the trial for futility. The values were plotted against cut-off points for different effect sizes in P_{S_E} for $\phi_E = \phi_C = 0.5$. First column corresponds to the results under the null hypothesis, middle column to the simulations at moderate power and right to the simulations under alternative hypothesis. The rows correspond to no effect, moderate effect, effect equal to the one P_{L_E} under the alternative hypothesis, and a higher effect than for P_{L_E} respectively. Grey lines correspond to observed effect conditional power, $CP_{\hat{\theta}}$, whereas black to fixed effect conditional power, CP_{θ_D}. $\hat{P}^{(1)}_{\mathbf{B}}$ is denoted by dotted lines, $\hat{P}^{(1)}_{\mathbf{L}}$ by solid and $\hat{P}^{(1)}_{\mathbf{S}}$ by dot-dashed.

2.1.3 Probability to Make the Correct Decision

```
prob_corr_dec_plots <- create_prob_corr_dec_plots(
  output_cp_alt = output_cp_alt,
  output_cp_mod = output_cp_mod,
  output_cp_null = output_cp_null,
  phi_e = 0.5,
  phi_c = 0.5,
  filetype = "none")
```

```
grid.arrange(prob_corr_dec_plots)
```

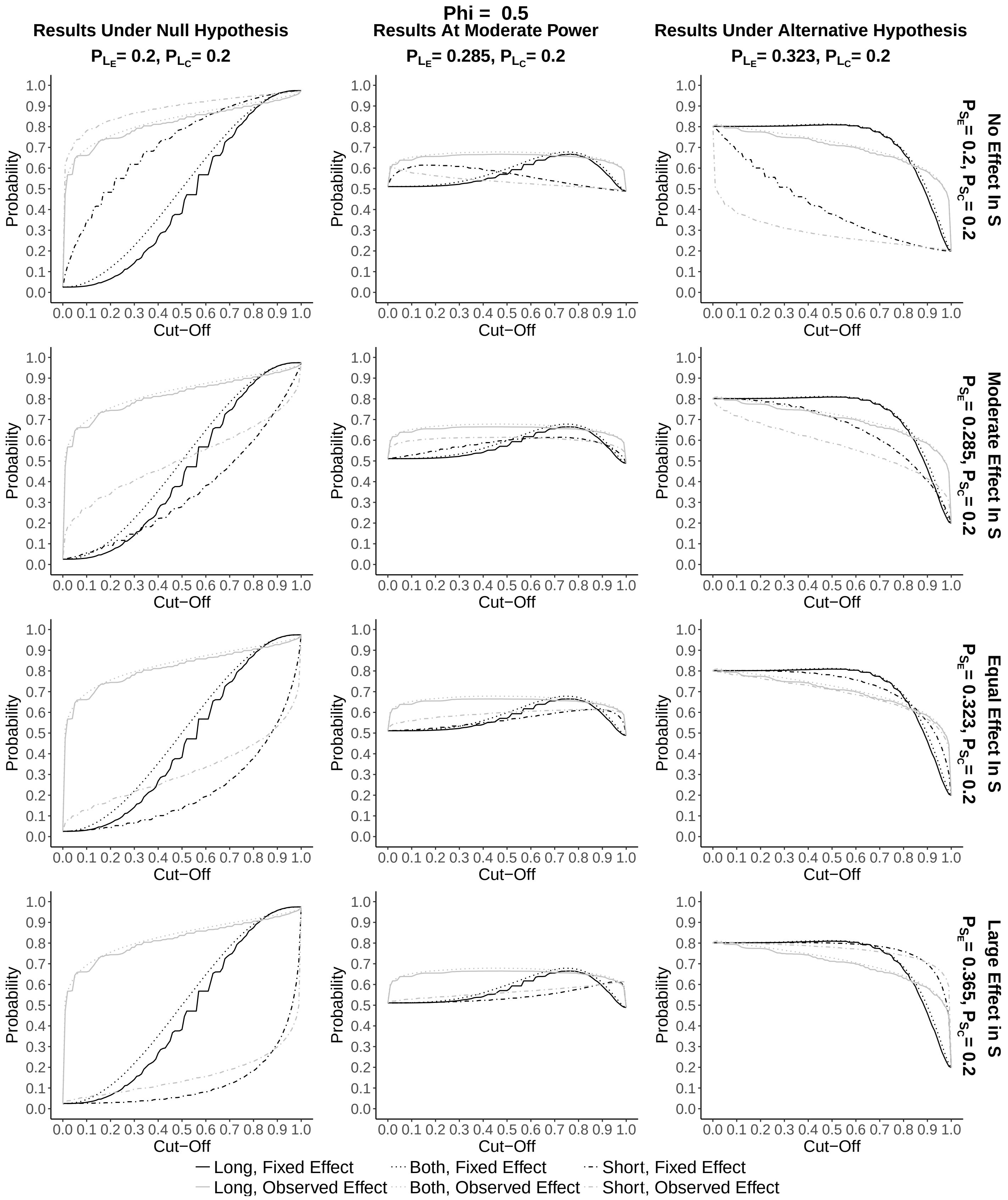

Figure 3: Plots showing the probability of making the correct decision at interim plotted against cut-off points for different effect sizes in P_{S_E} for $\phi_E = \phi_C = 0.5$. First column corresponds to the results under the null hypothesis, middle column to the simulations at moderate power and right to the simulations under alternative hypothesis. The rows correspond to no effect, moderate effect, effect equal to the one P_{L_E} under the alternative hypothesis, and a higher effect than for P_{L_E} respectively. Grey lines correspond to observed effect conditional power, $CP_{\hat{\theta}}$, whereas black to fixed effect conditional power, CP_{heta_D}. $\hat{P}^{(1)}_{\mathbf{B}}$ is denoted by dotted lines, $\hat{P}^{(1)}_{\mathbf{L}}$ by solid and $\hat{P}^{(1)}_{\mathbf{S}}$ by dot-dashed.

2.2 Varying Correlation between S and L

2.2.1 $\phi_E = \phi_C = 0.2$

```
p_se <- c(0.2, 0.285, 0.3227348, 0.365)

output_cp_alt <- foreach(i = 1:length(p_se), .packages="binfutssr") %dopar%
  (cp(nsim = 100000,        #number of simulations
      alpha = 0.025,         # one-sided alpha
      beta = 0.2,            # type 2 error such that 1-beta is the power
      p_le = 0.3227348,      # probability of success for long-term endpoint in E
      p_lc = 0.2,            # probability of success for long-term endpoint in C
      p_se = p_se[i],        # i-th probability of success for short-term endpoint in E
      p_sc = 0.2,            # probability of success for short-term endpoint in C
      n = 200,               # sample size per treatment arm
      fr_lo = 0.25,          # amounut of long-term information available at interim
      fr_sh = 0.5,           # amounut of short-term information available at interim
      phi_e = 0.2,           # correlation in E
      phi_c = 0.2,           # correlation in C
      c = seq(0, 1, 0.01)))  #sequence of cut-off points for stopping based on cp


#Simulate clinical trial for moderate power (~50%)



output_cp_mod <- foreach(i = 1:length(p_se), .packages="binfutssr") %dopar%
  (cp(nsim = 100000,
      alpha = 0.025,
      beta = 0.2,
      p_le = 0.285,
      p_lc = 0.2,
      p_se = p_se[i],
      p_sc = 0.2,
      n = 200,
      fr_lo = 0.25,
      fr_sh = 0.5,
      phi_e = 0.2,
      phi_c = 0.2,
      c = seq(0, 1, 0.01)))


#Simulate clinical trial under the null hypothesis


output_cp_null <- foreach(i = 1:length(p_se), .packages="binfutssr") %dopar%
  (cp(nsim = 100000,
      alpha = 0.025,
      beta = 0.2,
      p_le = 0.2,
      p_lc = 0.2,
      p_se = p_se[i],
      p_sc = 0.2,
      n = 200,
      fr_lo = 0.25,
      fr_sh = 0.5,
      phi_e = 0.2,
      phi_c = 0.2,
```

```
      c = seq(0, 1, 0.01)))
```

2.2.1.1 Overall Power

```
power_plots_phi_02 <- create_power_plots(output_cp_alt = output_cp_alt,
                                         output_cp_mod = output_cp_mod,
                                         output_cp_null = output_cp_null,
                                         phi_e = 0.2,
                                         phi_c = 0.2,
                                         filetype = "none")
```

```
grid.arrange(power_plots_phi_02)
```

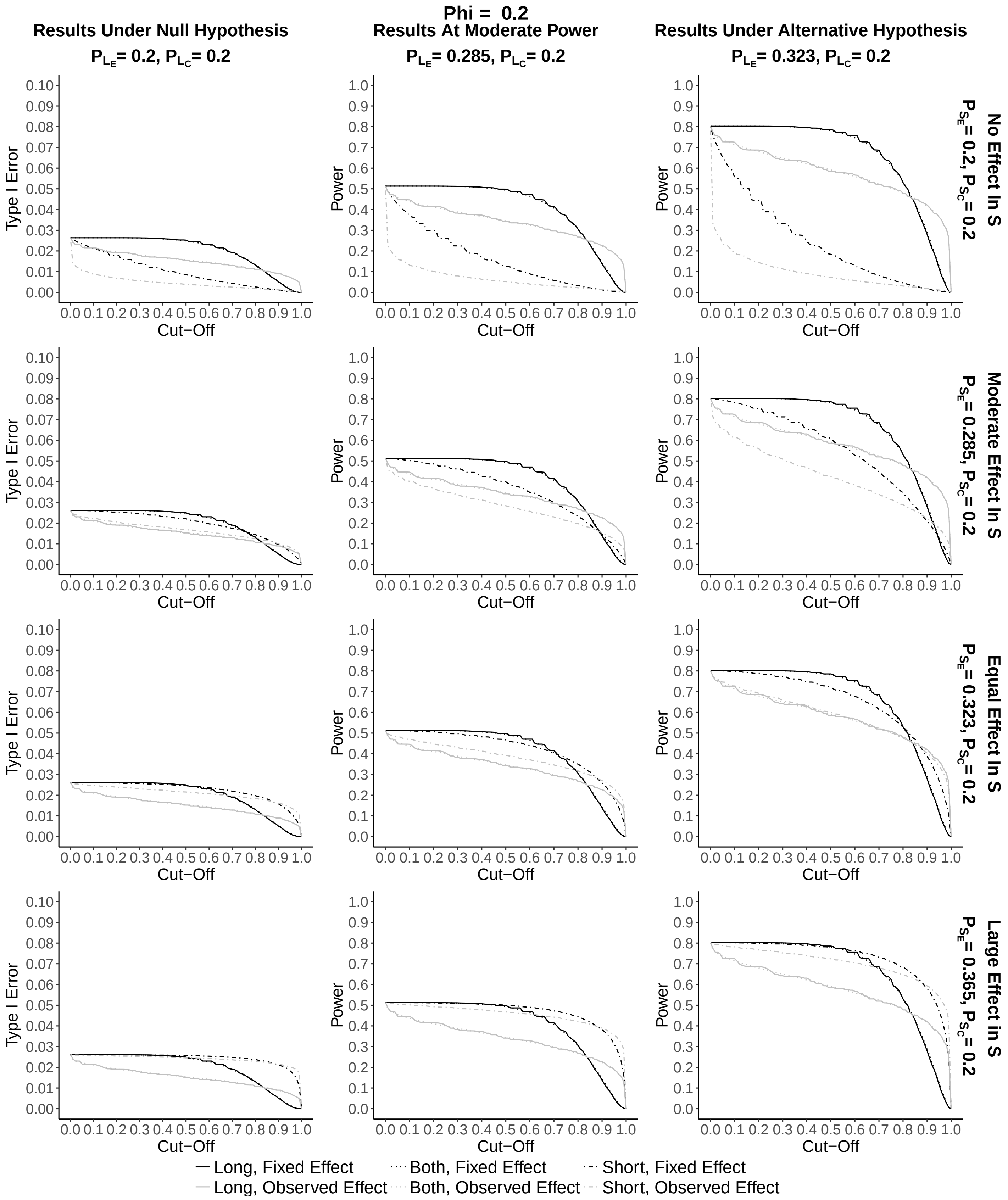

Figure 4: Plots showing the power plotted against cut-off points for different effect sizes in P_{S_E} for $\phi_E = \phi_C = 0.2$. First column corresponds to the results under the null hypothesis, middle column to the simulations at moderate power and right to the simulations under alternative hypothesis. The rows correspond to no effect, moderate effect, effect equal to the one P_{L_E} under the alternative hypothesis, and a higher effect than for P_{L_E} respectively. Grey lines correspond to observed effect conditional power, $CP_{\hat{\theta}}$, whereas black to fixed effect conditional power, CP_{θ_D}. $\hat{P}_{\mathbf{B}}^{(1)}$ is denoted by dotted lines, $\hat{P}_{\mathbf{L}}^{(1)}$ by solid and $\hat{P}_{\mathbf{S}}^{(1)}$ by dot-dashed. Note that the scale in the first column is from 0 to 0.1.

2.2.1.2 Probability to Stop for Futility

```
fs_plots_phi_02 <- create_fs_plots(output_cp_alt = output_cp_alt,
                                   output_cp_mod = output_cp_mod,
                                   output_cp_null = output_cp_null,
                                   phi_e = 0.2,
                                   phi_c = 0.2,
                                   filetype = "none")
```

```
grid.arrange(fs_plots_phi_02)
```

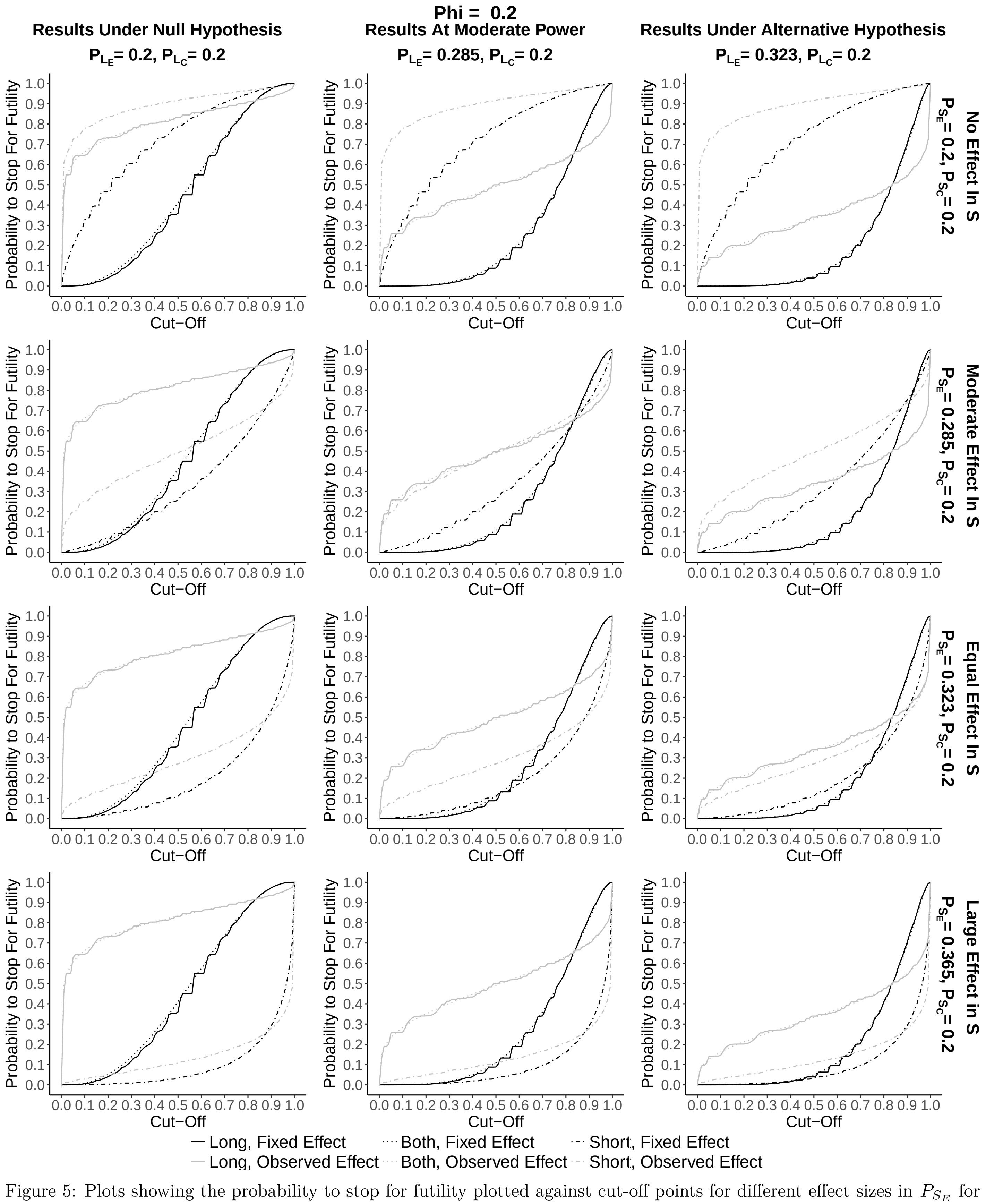

Figure 5: Plots showing the probability to stop for futility plotted against cut-off points for different effect sizes in P_{S_E} for $\phi_E = \phi_C = 0.2$. First column corresponds to the results under the null hypothesis, middle column to the simulations at moderate power and right to the simulations under alternative hypothesis. The rows correspond to no effect, moderate effect, effect equal to the one P_{L_E} under the alternative hypothesis, and a higher effect than for P_{L_E} respectively. Grey lines correspond to observed effect conditional power, $CP_{\hat{\theta}}$, whereas black to fixed effect conditional power, CP_{θ_D}. $\hat{P}^{(1)}_{\mathbf{B}}$ is denoted by dotted lines, $\hat{P}^{(1)}_{\mathbf{L}}$ by solid and $\hat{P}^{(1)}_{\mathbf{S}}$ by dot-dashed.

2.2.1.3 Probability to Reject the Null Hypothesis Given Trial Was Continued

```
prob_cont_rej_plots_phi_02 <- create_prob_cont_rej_plots(
  output_cp_alt = output_cp_alt,
  output_cp_mod = output_cp_mod,
  output_cp_null = output_cp_null,
  phi_e = 0.2,
  phi_c = 0.2,
  filetype = "none")
```

```
grid.arrange(prob_cont_rej_plots_phi_02)
```

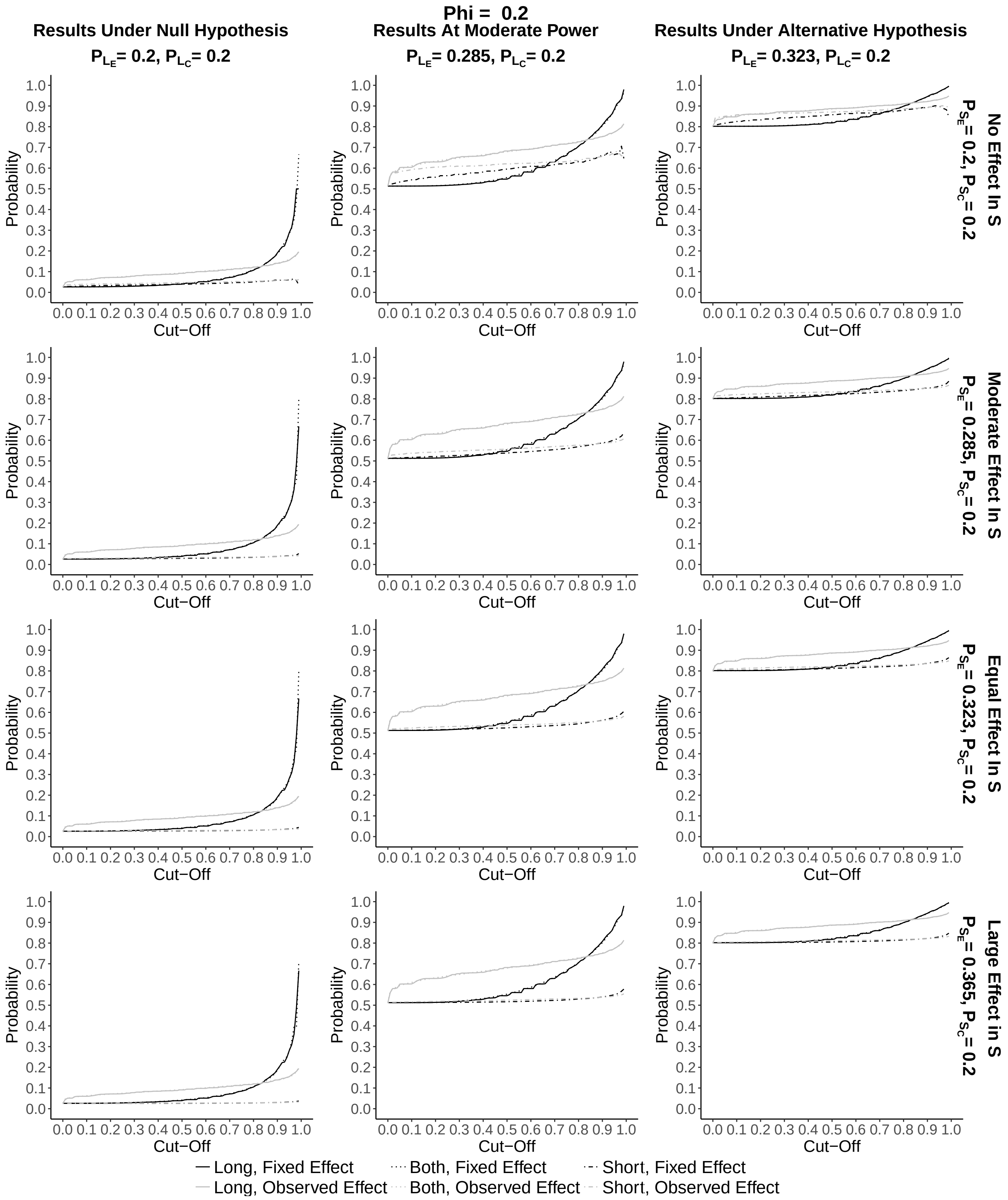

Figure 6: Plots showing the probability to reject the null hypothesis given the trial was continued plotted against cut-off points for different effect sizes in P_{S_E} for $\phi_E = \phi_C = 0.2$. First column corresponds to the results under the null hypothesis, middle column to the simulations at moderate power and right to the simulations under alternative hypothesis. The rows correspond to no effect, moderate effect, effect equal to the one P_{L_E} under the alternative hypothesis, and a higher effect than for P_{L_E} respectively. Grey lines correspond to observed effect conditional power, $CP_{\hat{\theta}}$, whereas black to fixed effect conditional power, CP_{θ_D}. $\hat{P}^{(1)}_{\mathbf{B}}$ is denoted by dotted lines, $\hat{P}^{(1)}_{\mathbf{L}}$ by solid and $\hat{P}^{(1)}_{\mathbf{S}}$ by dot-dashed.

2.2.1.4 Probability to Fail to Reject the Null Hypothesis Given The Trial Was Stopped Had it Been Continued

```
prob_stop_not_rej_plots_phi_02 <- create_prob_stop_not_rej_plots(
  output_cp_alt = output_cp_alt,
  output_cp_mod = output_cp_mod,
  output_cp_null = output_cp_null,
  phi_e = 0.2,
  phi_c = 0.2,
  filetype = "none")
```

```
grid.arrange(prob_stop_not_rej_plots_phi_02)
```

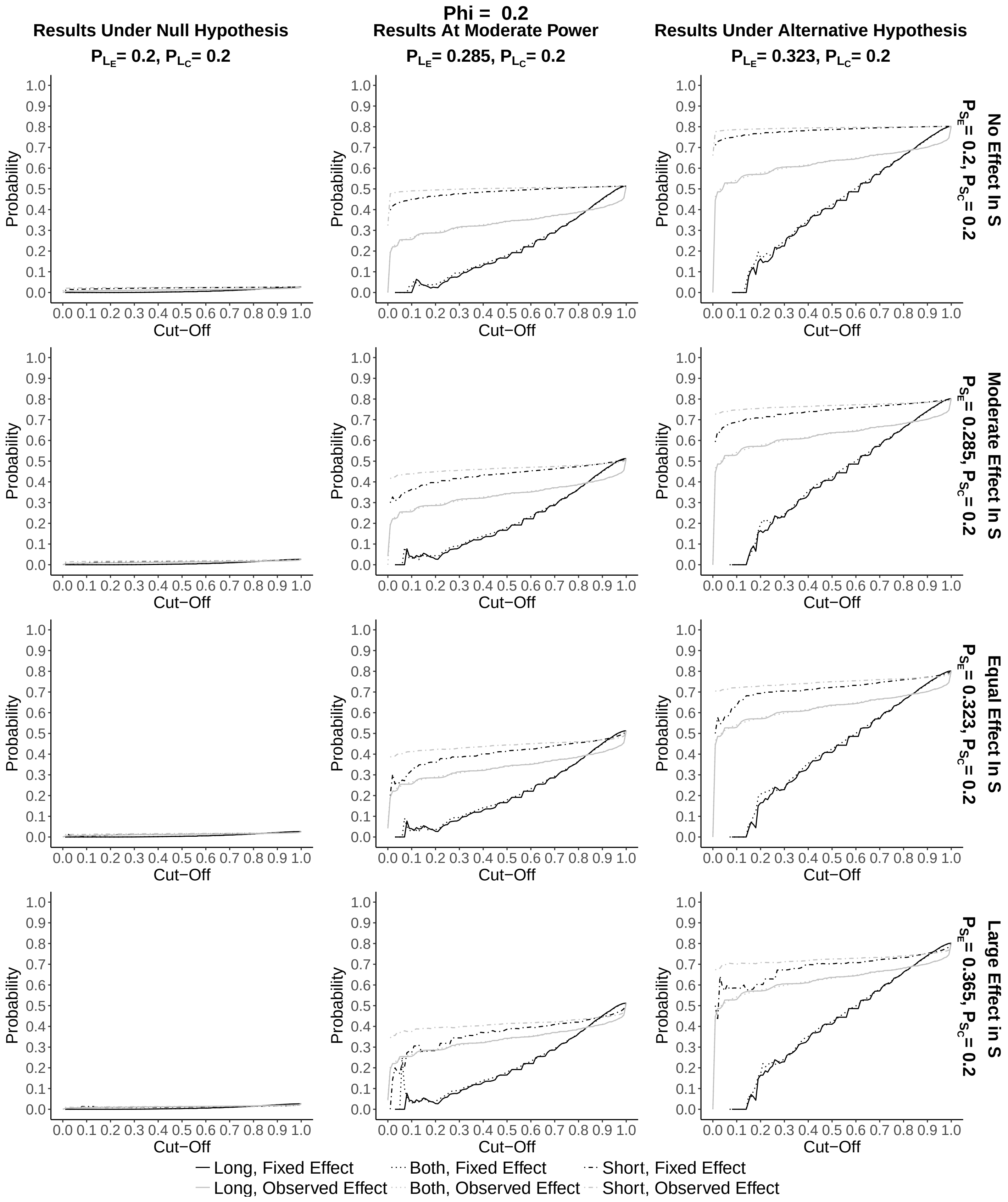

Figure 7: Plots showing the probability of not having rejected the null hypothesis, if the trial had been continued for the cases where the interim decision was to stop the trial for futility. The values were plotted against cut-off points for different effect sizes in P_{S_E} for $\phi_E = \phi_C = 0.2$. First column corresponds to the results under the null hypothesis, middle column to the simulations at moderate power and right to the simulations under alternative hypothesis. The rows correspond to no effect, moderate effect, effect equal to the one P_{L_E} under the alternative hypothesis, and a higher effect than for P_{L_E} respectively. Grey lines correspond to observed effect conditional power, $CP_{\hat{\theta}}$, whereas black to fixed effect conditional power, CP_{θ_D}. $\hat{P}^{(1)}_{\mathbf{B}}$ is denoted by dotted lines, $\hat{P}^{(1)}_{\mathbf{L}}$ by solid and $\hat{P}^{(1)}_{\mathbf{S}}$ by dot-dashed.

2.2.1.5 Probability to Make the Correct Decision

```
prob_corr_dec_plots_phi_02 <- create_prob_corr_dec_plots(
  output_cp_alt = output_cp_alt,
  output_cp_mod = output_cp_mod,
  output_cp_null = output_cp_null,
  phi_e = 0.2,
  phi_c = 0.2,
  filetype = "none")
```

```
grid.arrange(prob_corr_dec_plots_phi_02)
```

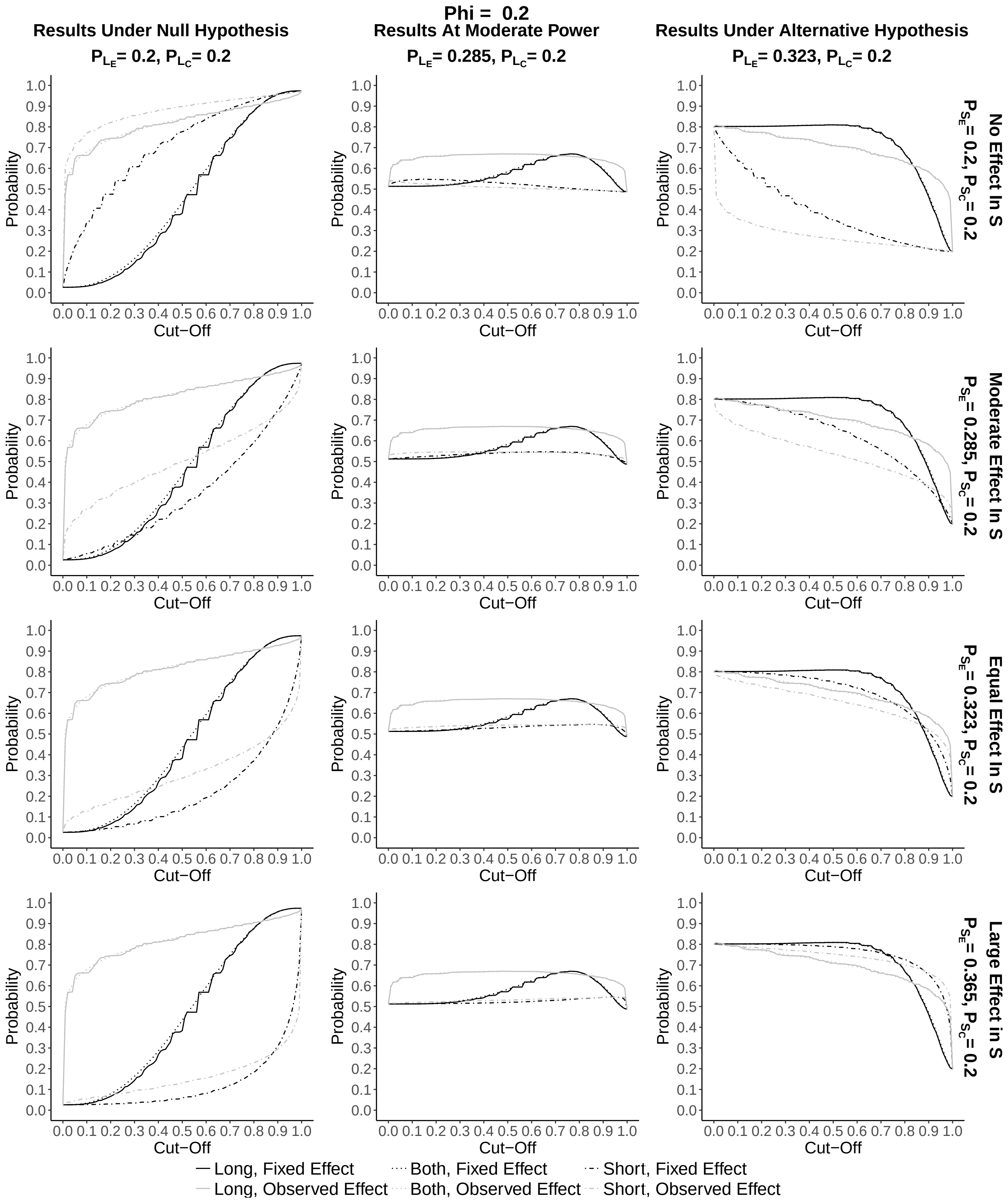

Figure 8: Plots showing the probability of making the correct decision at interim plotted against cut-off points for different effect sizes in P_{S_E} for $\phi_E = \phi_C = 0.2$. First column corresponds to the results under the null hypothesis, middle column to the simulations at moderate power and right to the simulations under alternative hypothesis. The rows correspond to no effect, moderate effect, effect equal to the one P_{L_E} under the alternative hypothesis, and a higher effect than for P_{L_E} respectively. Grey lines correspond to observed effect conditional power, $CP_{\hat{\theta}}$, whereas black to fixed effect conditional power, CP_{θ_D}. $\hat{P}^{(1)}_{\mathbf{B}}$ is denoted by dotted lines, $\hat{P}^{(1)}_{\mathbf{L}}$ by solid and $\hat{P}^{(1)}_{\mathbf{S}}$ by dot-dashed.

2.2.2 $\phi_E = \phi_C = 0.65$

```
p_se <- c(0.2, 0.285, 0.3227348, 0.365)

output_cp_alt <- foreach(i = 1:length(p_se), .packages="binfutssr") %dopar%
  (cp(nsim = 100000,          #number of simulations
      alpha = 0.025,          # one-sided alpha
      beta = 0.2,             # type 2 error such that 1-beta is the power
      p_le = 0.3227348,       # probability of success for long-term endpoint in E
      p_lc = 0.2,             # probability of success for long-term endpoint in C
      p_se = p_se[i],         # i-th probability of success for short-term endpoint in E
      p_sc = 0.2,             # probability of success for short-term endpoint in C
      n = 200,                # sample size per treatment arm
      fr_lo = 0.25,           # amounut of long-term information available at interim
      fr_sh = 0.5,            # amounut of short-term information available at interim
      phi_e = 0.65,            # correlation in E
      phi_c = 0.65,            # correlation in C
      c = seq(0, 1, 0.01)))   #sequence of cut-off points for stopping based on cp


#Simulate clinical trial for moderate power (~50%)



output_cp_mod <- foreach(i = 1:length(p_se), .packages="binfutssr") %dopar%
  (cp(nsim = 100000,
      alpha = 0.025,
      beta = 0.2,
      p_le = 0.285,
      p_lc = 0.2,
      p_se = p_se[i],
      p_sc = 0.2,
      n = 200,
      fr_lo = 0.25,
      fr_sh = 0.5,
      phi_e = 0.65,
      phi_c = 0.65,
      c = seq(0, 1, 0.01)))


#Simulate clinical trial under the null hypothesis


output_cp_null <- foreach(i = 1:length(p_se), .packages="binfutssr") %dopar%
  (cp(nsim = 100000,
      alpha = 0.025,
      beta = 0.2,
      p_le = 0.2,
      p_lc = 0.2,
      p_se = p_se[i],
      p_sc = 0.2,
      n = 200,
      fr_lo = 0.25,
      fr_sh = 0.5,
      phi_e = 0.65,
      phi_c = 0.65,
      c = seq(0, 1, 0.01)))
```

2.2.3 Overall Power

```
power_plot_phi_065 <- create_power_plots(output_cp_alt = output_cp_alt,
                                         output_cp_mod = output_cp_mod,
                                         output_cp_null = output_cp_null,
                                         phi_e = 0.65,
                                         phi_c = 0.65,
                                         filetype = "none")
```

```
grid.arrange(power_plot_phi_065)
```

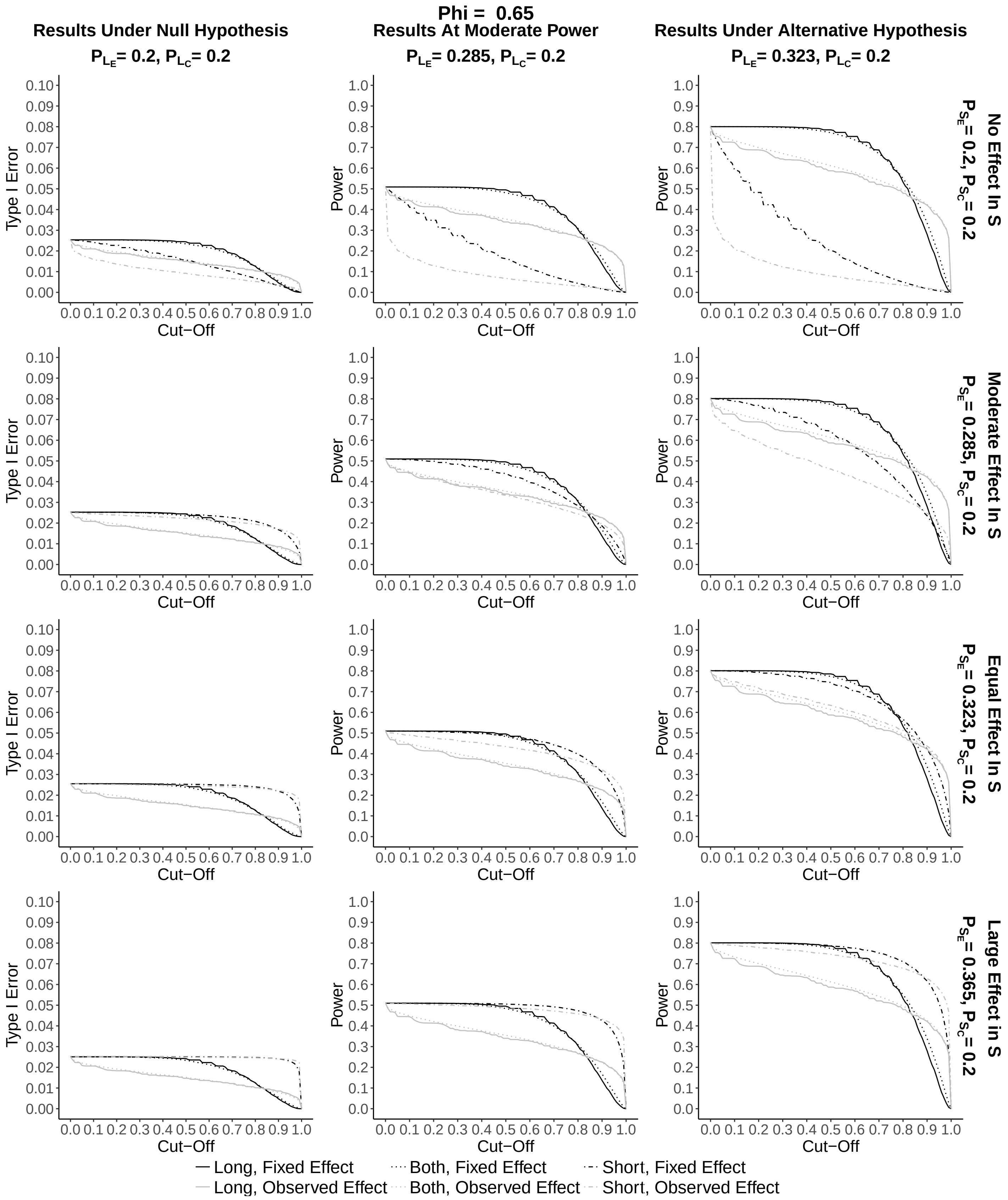

Figure 9: Plots showing the power plotted against cut-off points for different effect sizes in P_{S_E} for $\phi_E = \phi_C = 0.65$. First column corresponds to the results under the null hypothesis, middle column to the simulations at moderate power and right to the simulations under alternative hypothesis. The rows correspond to no effect, moderate effect, effect equal to the one P_{L_E} under the alternative hypothesis, and a higher effect than for P_{L_E} respectively. Grey lines correspond to observed effect conditional power, $CP_{\hat{\theta}}$, whereas black to fixed effect conditional power, CP_{θ_D}. $\hat{P}_{\mathbf{B}}^{(1)}$ is denoted by dotted lines, $\hat{P}_{\mathbf{L}}^{(1)}$ by solid and $\hat{P}_{\mathbf{S}}^{(1)}$ by dot-dashed. Note that the scale in the first column is from 0 to 0.1.

2.2.3.1 Probability to Stop for Futility

```
fs_plots_phi_065 <- create_fs_plots(output_cp_alt = output_cp_alt,
                                    output_cp_mod = output_cp_mod,
                                    output_cp_null = output_cp_null,
                                    phi_e = 0.65,
                                    phi_c = 0.65,
                                    filetype = "none")
```

```
grid.arrange(fs_plots_phi_065)
```

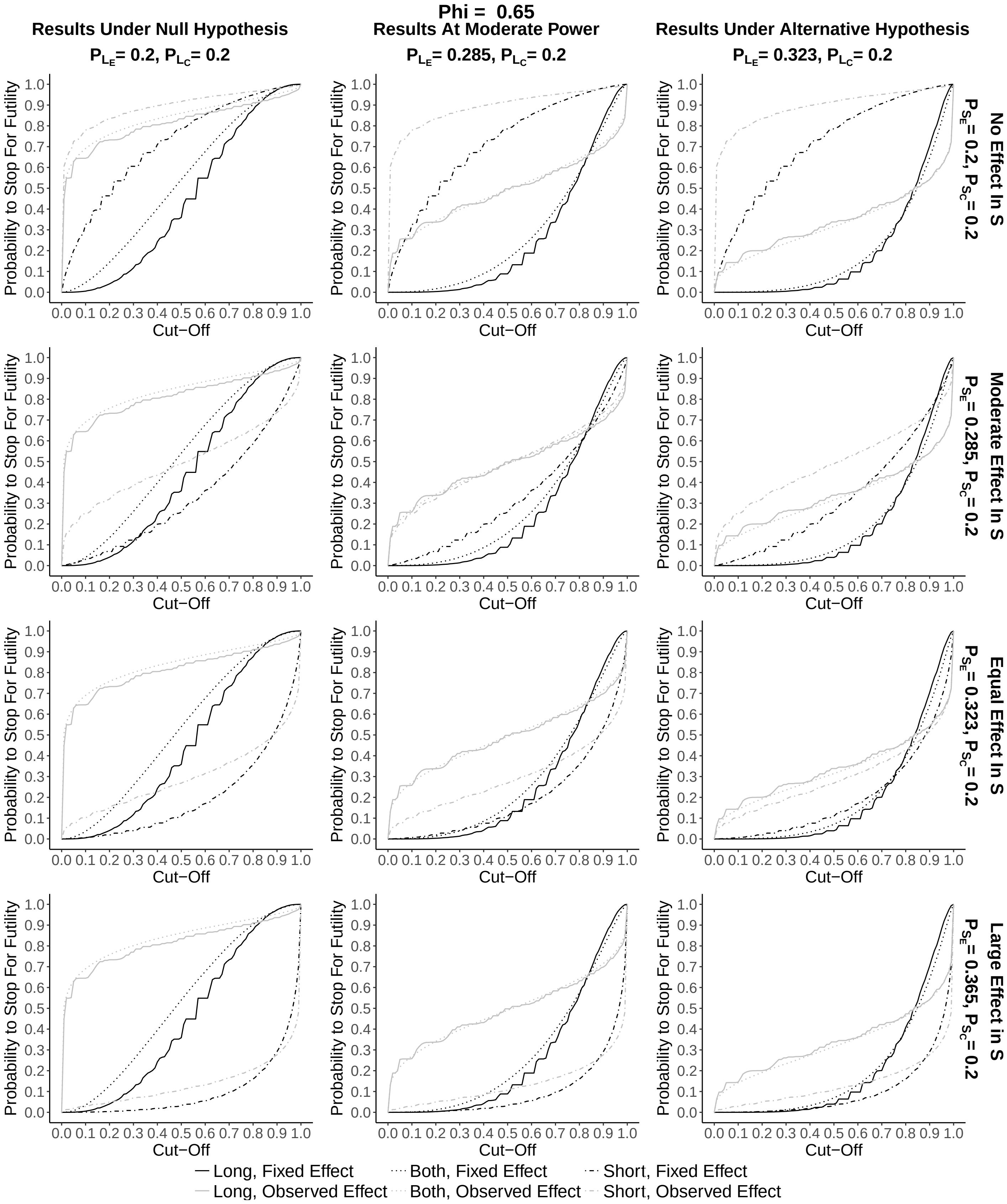

Figure 10: Plots showing the probability to stop for futility plotted against cut-off points for different effect sizes in P_{S_E} for $\phi_E = \phi_C = 0.65$. First column corresponds to the results under the null hypothesis, middle column to the simulations at moderate power and right to the simulations under alternative hypothesis. The rows correspond to no effect, moderate effect, effect equal to the one P_{L_E} under the alternative hypothesis, and a higher effect than for P_{L_E} respectively. Grey lines correspond to observed effect conditional power, $CP_{\hat{\theta}}$, whereas black to fixed effect conditional power, CP_{θ_D}. $\hat{P}_{\mathbf{B}}^{(1)}$ is denoted by dotted lines, $\hat{P}_{\mathbf{L}}^{(1)}$ by solid and $\hat{P}_{\mathbf{S}}^{(1)}$ by dot-dashed.

2.2.3.2 Probability to Reject the Null Hypothesis Given Trial Was Continued

```
prob_cont_rej_plots_phi_065 <- create_prob_cont_rej_plots(
  output_cp_alt = output_cp_alt,
  output_cp_mod = output_cp_mod,
  output_cp_null = output_cp_null,
  phi_e = 0.65,
  phi_c = 0.65,
  filetype = "none")
```

```
grid.arrange(prob_cont_rej_plots_phi_065)
```

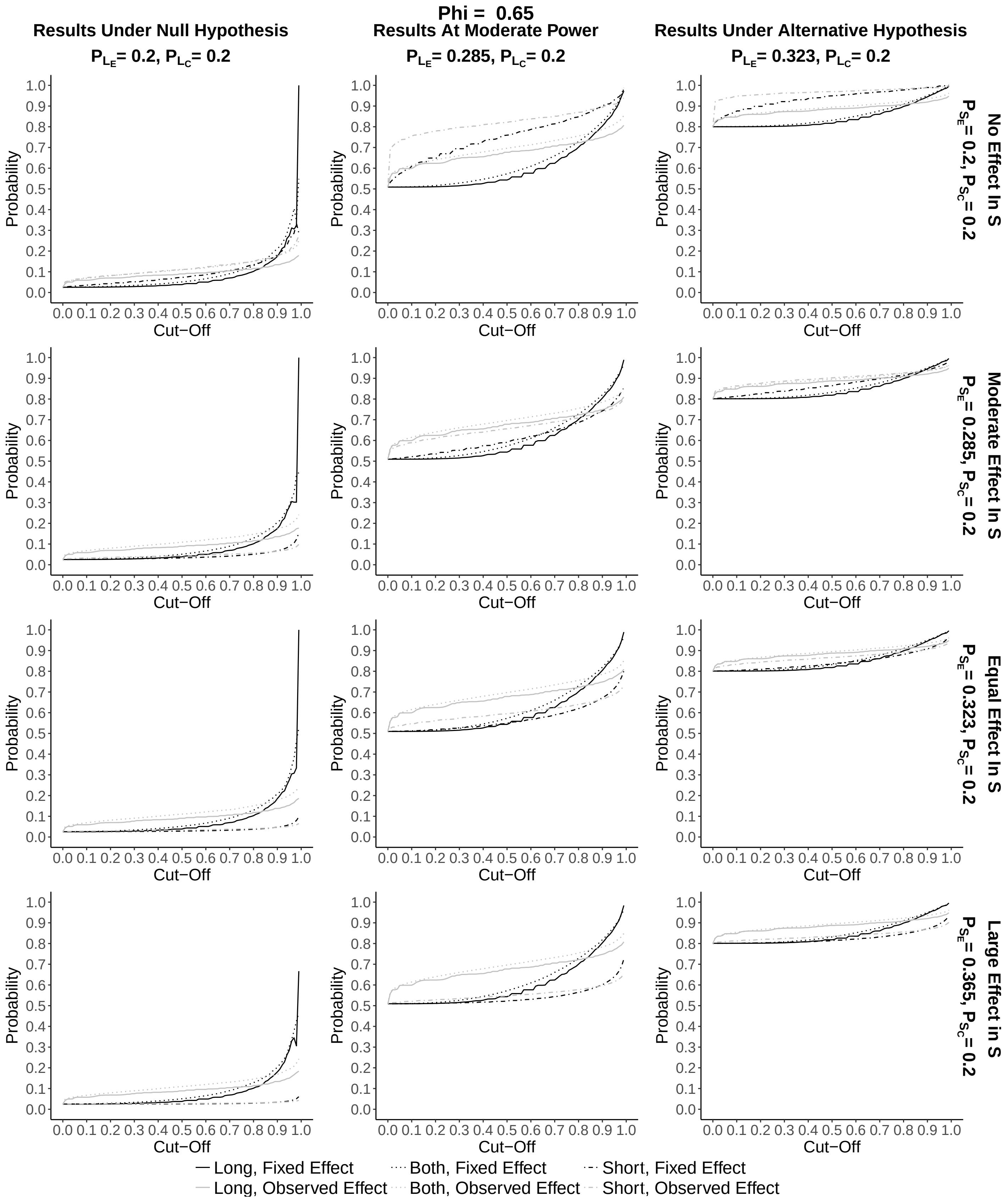

Figure 11: Plots showing the probability to reject the null hypothesis given the trial was continued plotted against cut-off points for different effect sizes in P_{S_E} for $\phi_E = \phi_C = 0.65$. First column corresponds to the results under the null hypothesis, middle column to the simulations at moderate power and right to the simulations under alternative hypothesis. The rows correspond to no effect, moderate effect, effect equal to the one P_{L_E} under the alternative hypothesis, and a higher effect than for P_{L_E} respectively. Grey lines correspond to observed effect conditional power, $CP_{\hat{\theta}}$, whereas black to fixed effect conditional power, CP_{θ_D}. $\hat{P}_{\mathbf{B}}^{(1)}$ is denoted by dotted lines, $\hat{P}_{\mathbf{L}}^{(1)}$ by solid and $\hat{P}_{\mathbf{S}}^{(1)}$ by dot-dashed.

2.2.3.3 Probability to Fail to Reject the Null Hypothesis Given The Trial Was Stopped Had it Been Continued

```
prob_stop_not_rej_plots_phi_065 <- create_prob_stop_not_rej_plots(
  output_cp_alt = output_cp_alt,
  output_cp_mod = output_cp_mod,
  output_cp_null = output_cp_null,
  phi_e = 0.65,
  phi_c = 0.65,
  filetype = "none")
```

```
grid.arrange(prob_stop_not_rej_plots_phi_065)
```

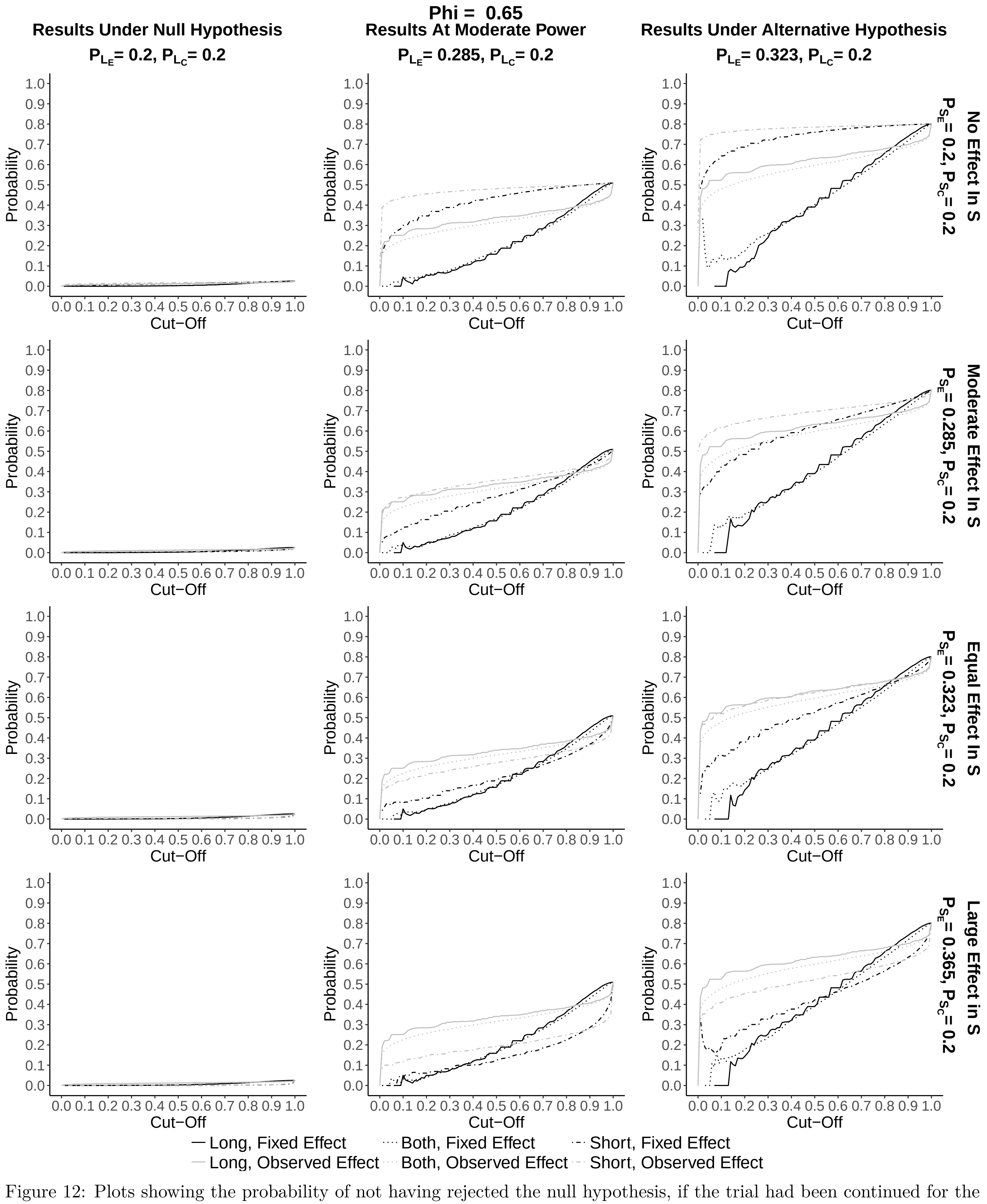

Figure 12: Plots showing the probability of not having rejected the null hypothesis, if the trial had been continued for the cases where the interim decision was to stop the trial for futility. The values were plotted against cut-off points for different effect sizes in P_{S_E} for $\phi_E = \phi_C = 0.65$. First column corresponds to the results under the null hypothesis, middle column to the simulations at moderate power and right to the simulations under alternative hypothesis. The rows correspond to no effect, moderate effect, effect equal to the one P_{L_E} under the alternative hypothesis, and a higher effect than for P_{L_E} respectively. Grey lines correspond to observed effect conditional power, $CP_{\hat{\theta}}$, whereas black to fixed effect conditional power, CP_{θ_D}. $\hat{P}_{\mathbf{B}}^{(1)}$ is denoted by dotted lines, $\hat{P}_{\mathbf{L}}^{(1)}$ by solid and $\hat{P}_{\mathbf{S}}^{(1)}$ by dot-dashed.

2.2.3.4 Probability to Make the Correct Decision

```
prob_corr_dec_plots_phi_065 <- create_prob_corr_dec_plots(
  output_cp_alt = output_cp_alt,
  output_cp_mod = output_cp_mod,
  output_cp_null = output_cp_null,
  phi_e = 0.65,
  phi_c = 0.65,
  filetype = "none")
```

```
grid.arrange(prob_corr_dec_plots_phi_065)
```

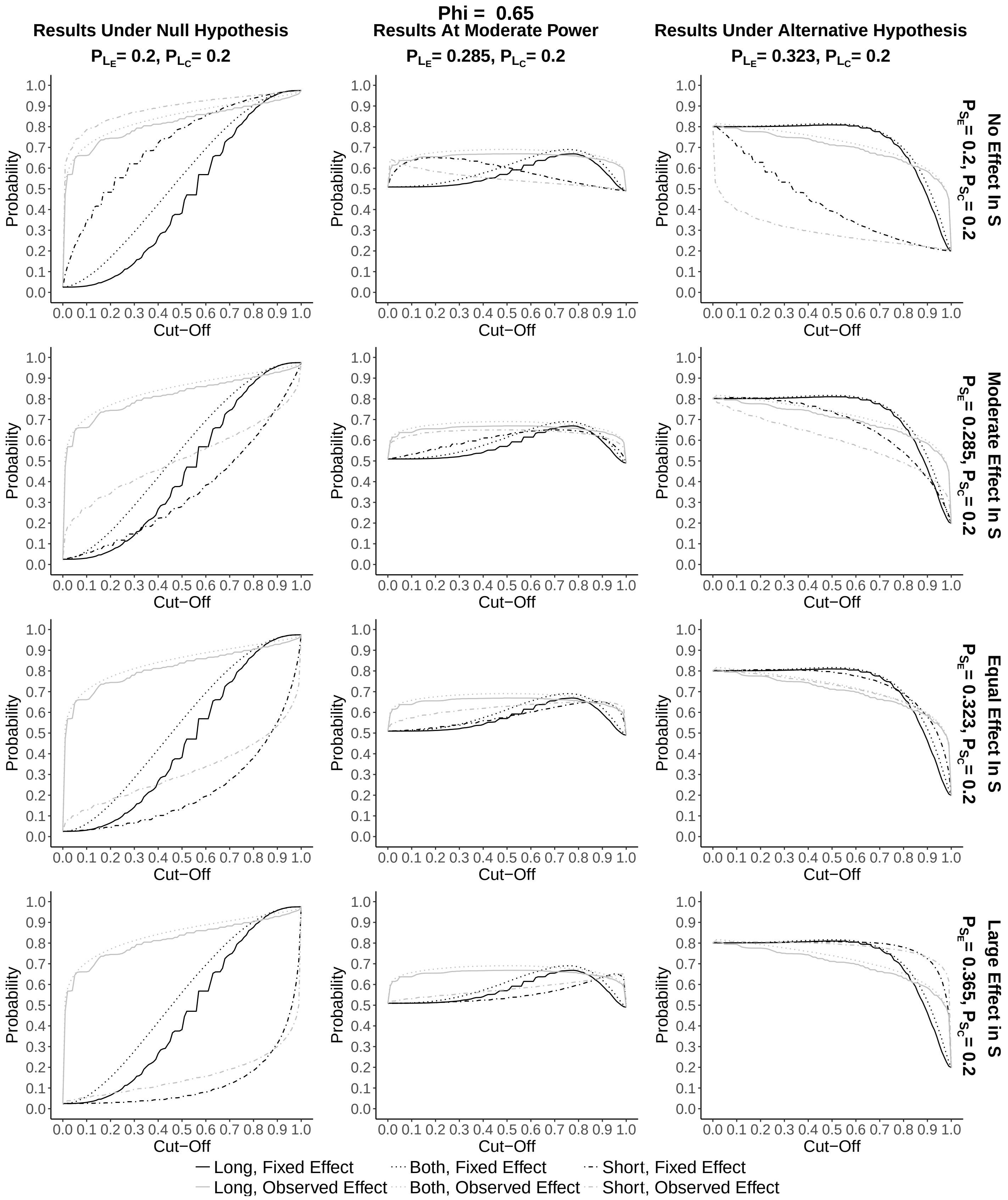

Figure 13: Plots showing the probability of making the correct decision at interim plotted against cut-off points for different effect sizes in P_{S_E} for $\phi_E = \phi_C = 0.65$. First column corresponds to the results under the null hypothesis, middle column to the simulations at moderate power and right to the simulations under alternative hypothesis. The rows correspond to no effect, moderate effect, effect equal to the one P_{L_E} under the alternative hypothesis, and a higher effect than for P_{L_E} respectively. Grey lines correspond to observed effect conditional power, $CP_{\hat{\theta}}$, whereas black to fixed effect conditional power, CP_{θ_D}. $\hat{P}_{\mathbf{B}}^{(1)}$ is denoted by dotted lines, $\hat{P}_{\mathbf{L}}^{(1)}$ by solid and $\hat{P}_{\mathbf{S}}^{(1)}$ by dot-dashed.

2.3 'Nested' Endpoints Example

We also looked at a scenario with 'nested' outcomes in which it was assumed $Pr(L_i = 1|S_i = 0) = 0$, so that if there is no successful outcome for the short-term endpoint, there would be no successful outcome for the long-term one resulting in a fixed correlation between S and L. The effect sizes in the short-term outcomes are hence always either equal to or larger than for long-term. For this reason, the probabilities of success in treatment arms for the short-outcomes were changed for both the control and experimental groups. We again considered results under the null hypothesis, at moderate power and design power, and changed effects in S accordingly for no effect, moderate effect, effects at 80% of power and very high effects when comparing experimental and control groups. Under such scenarios the correlation between S_C and L_C, as well as S_E and L_E changes for each setting. The table below summarises the correlations and probabilities of success for each scenario presented below.

```
p_lc <- 0.2
p_sc <- 0.35
n <- 200
alpha <- 0.025
beta <- 0.2

p_le <- 0.3227348
p_se <- c(p_sc,power.prop.test(n = n, p1 = p_sc, sig.level = alpha,
                               power=0.5,
                               alternative = "one.sided")$p2,
          power.prop.test(n = n, p1 = p_sc, sig.level = alpha,
                          power=0.8,
                          alternative = "one.sided")$p2,
          power.prop.test(n = n, p1 = p_sc, sig.level = alpha,
                          power=0.9,
                          alternative = "one.sided")$p2)




phi_e <- floor(((p_le - p_le * p_se) /
                  sqrt(p_le * (1 - p_le) * (1 - p_se) * p_se)) * 1000000) / 1000000
phi_c <- floor(((p_lc - p_lc * p_sc) /
                  sqrt(p_lc * (1 - p_lc) * (1 - p_sc) * p_sc)) * 1000000) / 1000000

# m <- length(p_se) # if multiple cores are available set this value to length of p_se
# cl <- parallel::makeCluster(m)
# doParallel::registerDoParallel(cl)

output_cp_alt <- foreach(i = 1:length(p_se), .packages="binfutssr") %dopar%
  (cp(nsim = 100000,
      alpha = 0.025,
      beta = 0.2,
      p_le = 0.3227348,
      p_lc = 0.2,
      p_se = p_se[i],
      p_sc = 0.35,
      n = 200,
      fr_lo = 0.25,
      fr_sh = 0.5,
      phi_e = phi_e[i],
      phi_c = phi_c,
      c = seq(0, 1, 0.01)))

p_le <- power.prop.test(n = n, p1 = p_lc, sig.level = alpha,
                               power=0.5,
                               alternative = "one.sided")$p2
```

```r
phi_e <- floor(((p_le - p_le * p_se) /
                  sqrt(p_le * (1 - p_le) * (1 - p_se) * p_se)) * 1000000) / 1000000

output_cp_mod <- foreach(i = 1:length(p_se), .packages="binfutssr") %dopar%
  (cp(nsim = 100000,
      alpha = 0.025,
      beta = 0.2,
      p_le = power.prop.test(n = n, p1 = p_lc, sig.level = alpha,
                             power=0.5,
                             alternative = "one.sided")$p2,
      p_lc = 0.2,
      p_se = p_se[i],
      p_sc = 0.35,
      n = 200,
      fr_lo = 0.25,
      fr_sh = 0.5,
      phi_e = phi_e[i],
      phi_c = phi_c,
      c = seq(0, 1, 0.01)))

p_le <- 0.2
phi_e <- floor(((p_le - p_le * p_se) /
                  sqrt(p_le * (1 - p_le) * (1 - p_se) * p_se)) * 1000000) / 1000000

output_cp_null <- foreach(i = 1:length(p_se), .packages="binfutssr") %dopar%
  (cp(nsim = 100000,
      alpha = 0.025,
      beta = 0.2,
      p_le = 0.2,
      p_lc = 0.2,
      p_se = p_se[i],
      p_sc = 0.35,
      n = 200,
      fr_lo = 0.25,
      fr_sh = 0.5,
      phi_e = phi_e[i],
      phi_c = phi_c,
      c = seq(0, 1, 0.01)))
```

	P_{L_E} =0.2, P_{L_C} =0.2	P_{L_E} =0.284, P_{L_C} =0.2	P_{L_E} =0.323, P_{L_C} =0.2
P_{S_E} =0.35, P_{S_C} =0.35	Φ_E =0.681, Φ_C =0.681	Φ_E =0.858, Φ_C =0.681	Φ_E =0.941, Φ_C =0.681
P_{S_E} =0.446, P_{S_C} =0.35	Φ_E =0.557, Φ_C =0.681	Φ_E =0.702, Φ_C =0.681	Φ_E =0.769, Φ_C =0.681
P_{S_E} =0.488, P_{S_C} =0.35	Φ_E =0.512, Φ_C =0.681	Φ_E =0.645, Φ_C =0.681	Φ_E =0.707, Φ_C =0.681
P_{S_E} =0.51, P_{S_C} =0.35	Φ_E =0.49, Φ_C =0.681	Φ_E =0.618, Φ_C =0.681	Φ_E =0.677, Φ_C =0.681

Table 1: Correlations between S and L for all scenarios considered for the 'nested' endpoints.

2.3.1 Overall Power

```
power_plots_nested <- create_power_plots_nested(output_cp_alt = output_cp_alt,
                                                output_cp_mod = output_cp_mod,
                                                output_cp_null = output_cp_null,
                                                phi_e = phi_e,
                                                phi_c = phi_c,
                                                filetype = "none")
```

```
grid.arrange(power_plots_nested)
```

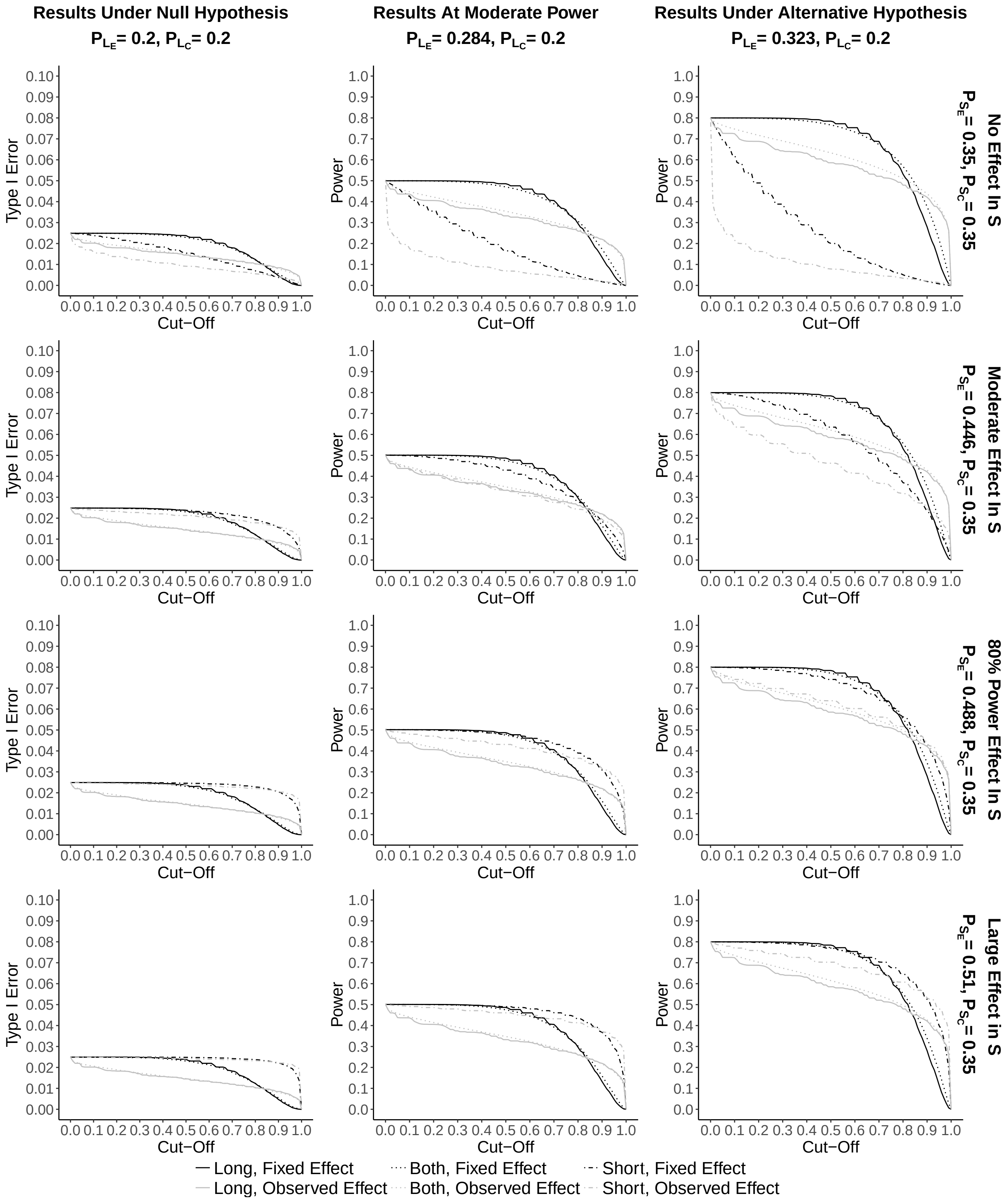

Figure 14: Plots showing the power plotted against cut-off points for different effect sizes in P_{S_E} for nested endpoints. First column corresponds to the results under the null hypothesis, middle column to the simulations at moderate power and right to the simulations under alternative hypothesis. The rows correspond to no effect, moderate effect, effect equal to 80% power compared to P_{S_C}, and a high effect in P_{S_E} respectively. Grey lines correspond to observed effect conditional power, $CP_{\hat{\theta}}$, whereas black to fixed effect conditional power, CP_{θ_D}. $\hat{P}_{\mathbf{B}}^{(1)}$ is denoted by dotted lines, $\hat{P}_{\mathbf{L}}^{(1)}$ by solid and $\hat{P}_{\mathbf{S}}^{(1)}$ by dot-dashed. Note that the scale in the first column is from 0 to 0.1.

2.3.2 Probability to Stop for Futility

```
fs_plots_nested <- create_fs_plots_nested(output_cp_alt = output_cp_alt,
                                          output_cp_mod = output_cp_mod,
                                          output_cp_null = output_cp_null,
                                          phi_e = phi_e,
                                          phi_c = phi_c,
                                          filetype = "none")
```

```
grid.arrange(fs_plots_nested)
```

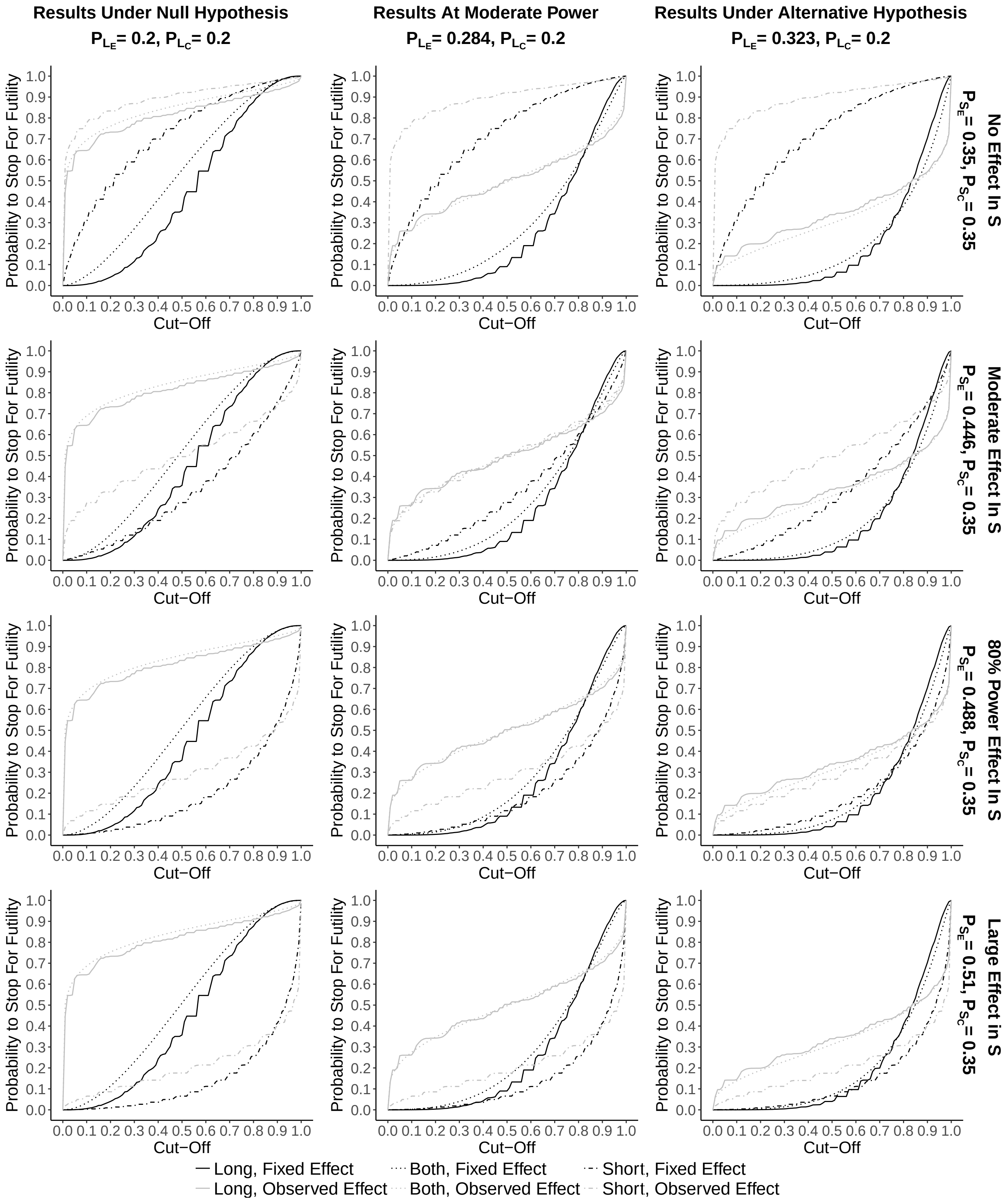

Figure 15: Plots showing the probability to stop for futility plotted against cut-off points for different effect sizes in P_{S_E} for nested endpoints. First column corresponds to the results under the null hypothesis, middle column to the simulations at moderate power and right to the simulations under alternative hypothesis. The rows correspond to no effect, moderate effect, effect equal to 80% power compared to P_{S_C}, and a high effect in P_{S_E} respectively. Grey lines correspond to observed effect conditional power, $CP_{\hat{\theta}}$, whereas black to fixed effect conditional power, CP_{θ_D}. $\hat{P}^{(1)}_{\mathbf{B}}$ is denoted by dotted lines, $\hat{P}^{(1)}_{\mathbf{L}}$ by solid and $\hat{P}^{(1)}_{\mathbf{S}}$ by dot-dashed.

2.3.3 Probability to Reject the Null Hypothesis Given Trial Was Continued

```
prob_cont_rej_plots_nested <- create_prob_cont_rej_plots_nested(
  output_cp_alt = output_cp_alt,
  output_cp_mod = output_cp_mod,
  output_cp_null = output_cp_null,
  phi_e = phi_e,
  phi_c = phi_c,
  filetype = "none")
```

```
grid.arrange(prob_cont_rej_plots_nested)
```

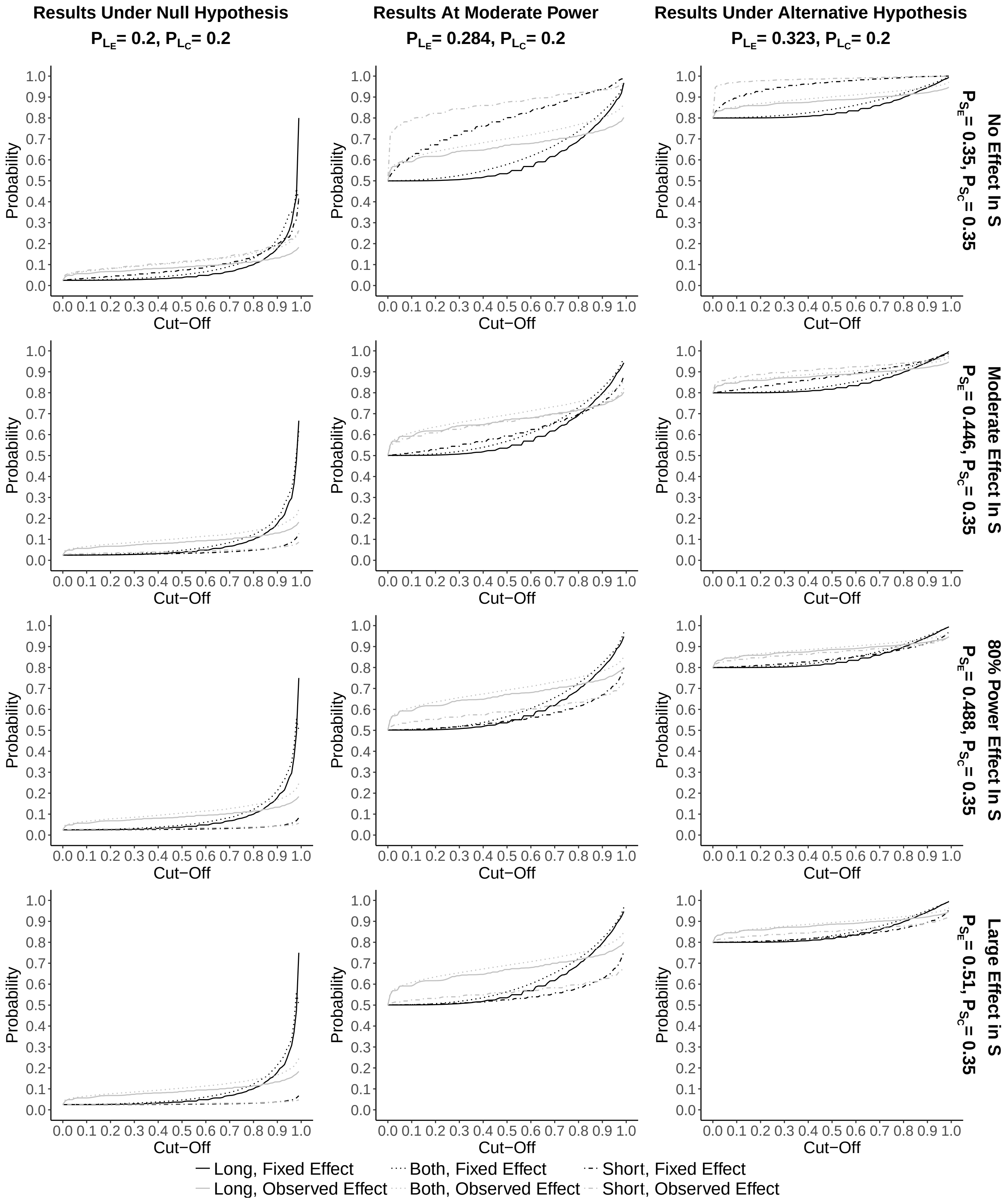

Figure 16: Plots showing the probability to reject the null hypothesis given the trial was continued plotted against cut-off points for different effect sizes in P_{S_E} for nested endpoints. First column corresponds to the results under the null hypothesis, middle column to the simulations at moderate power and right to the simulations under alternative hypothesis. The rows correspond to no effect, moderate effect, effect equal to 80% power compared to P_{S_C}, and a high effect in P_{S_E} respectively. Grey lines correspond to observed effect conditional power, $CP_{\hat{\theta}}$, whereas black to fixed effect conditional power, CP_{θ_D}. $\hat{P}^{(1)}_{\mathbf{B}}$ is denoted by dotted lines, $\hat{P}^{(1)}_{\mathbf{L}}$ by solid and $\hat{P}^{(1)}_{\mathbf{S}}$ by dot-dashed.

2.3.4 Probability to Fail to Reject the Null Hypothesis Given The Trial Was Stopped Had it Been Continued

```
prob_stop_not_rej_plots_nested <- create_prob_stop_not_rej_plots_nested(
  output_cp_alt = output_cp_alt,
  output_cp_mod = output_cp_mod,
  output_cp_null = output_cp_null,
  phi_e = phi_e,
  phi_c = phi_c,
  filetype = "none")
```

```
grid.arrange(prob_stop_not_rej_plots_nested)
```

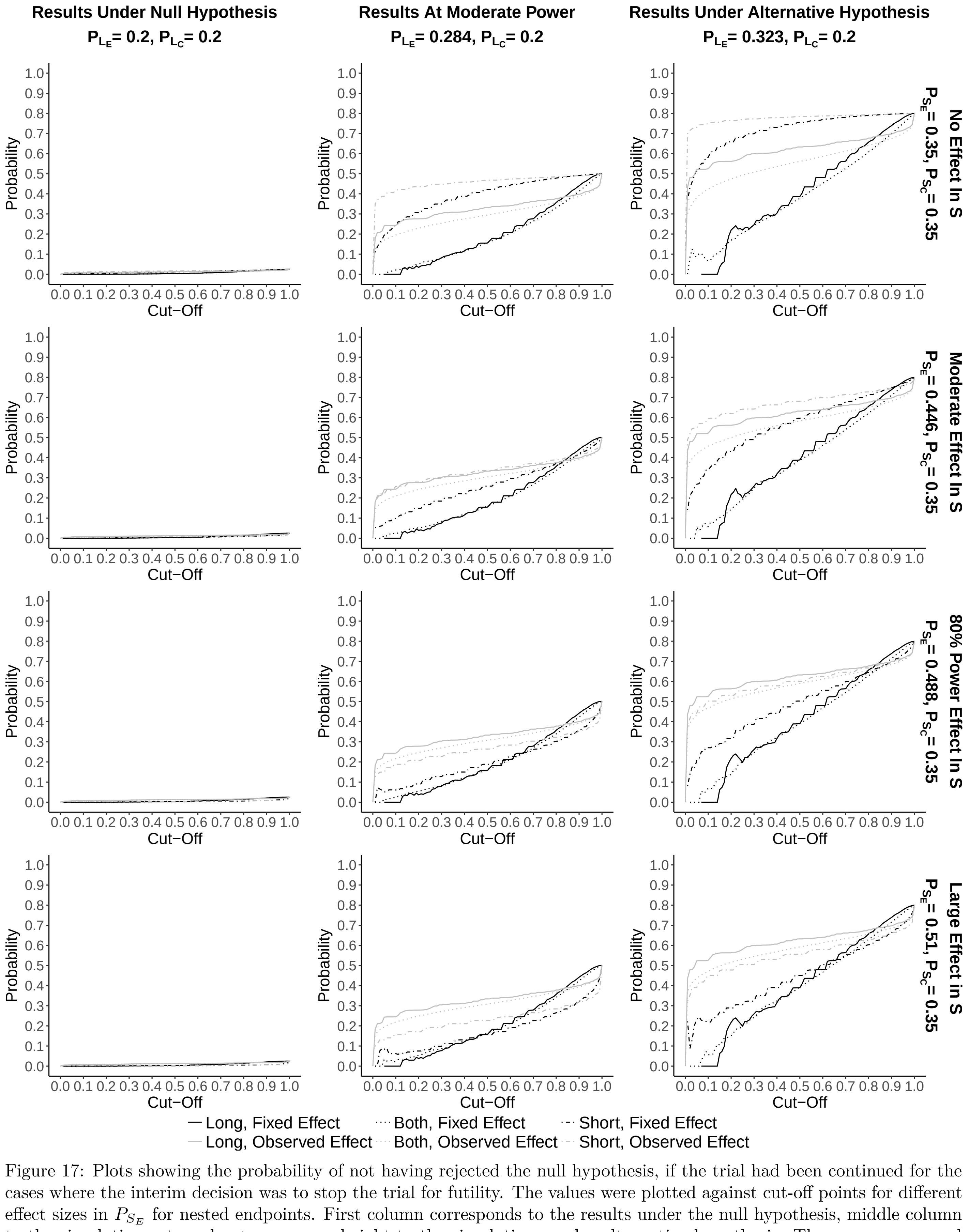

Figure 17: Plots showing the probability of not having rejected the null hypothesis, if the trial had been continued for the cases where the interim decision was to stop the trial for futility. The values were plotted against cut-off points for different effect sizes in P_{S_E} for nested endpoints. First column corresponds to the results under the null hypothesis, middle column to the simulations at moderate power and right to the simulations under alternative hypothesis. The rows correspond to no effect, moderate effect, effect equal to 80% power compared to P_{S_C}, and a high effect in P_{S_E} respectively. Grey lines correspond to observed effect conditional power, $CP_{\hat{\theta}}$, whereas black to fixed effect conditional power, CP_{θ_D}. $\hat{P}_{\mathbf{B}}^{(1)}$ is denoted by dotted lines, $\hat{P}_{\mathbf{L}}^{(1)}$ by solid and $\hat{P}_{\mathbf{S}}^{(1)}$ by dot-dashed.

2.3.5 Probability to Make the Correct Decision

```
prob_corr_dec_plots_nested <- create_prob_corr_dec_plots_nested(
  output_cp_alt = output_cp_alt,
  output_cp_mod = output_cp_mod,
  output_cp_null = output_cp_null,
  phi_e = phi_e,
  phi_c = phi_c,
  filetype = "none")
```

```
grid.arrange(prob_corr_dec_plots_nested)
```

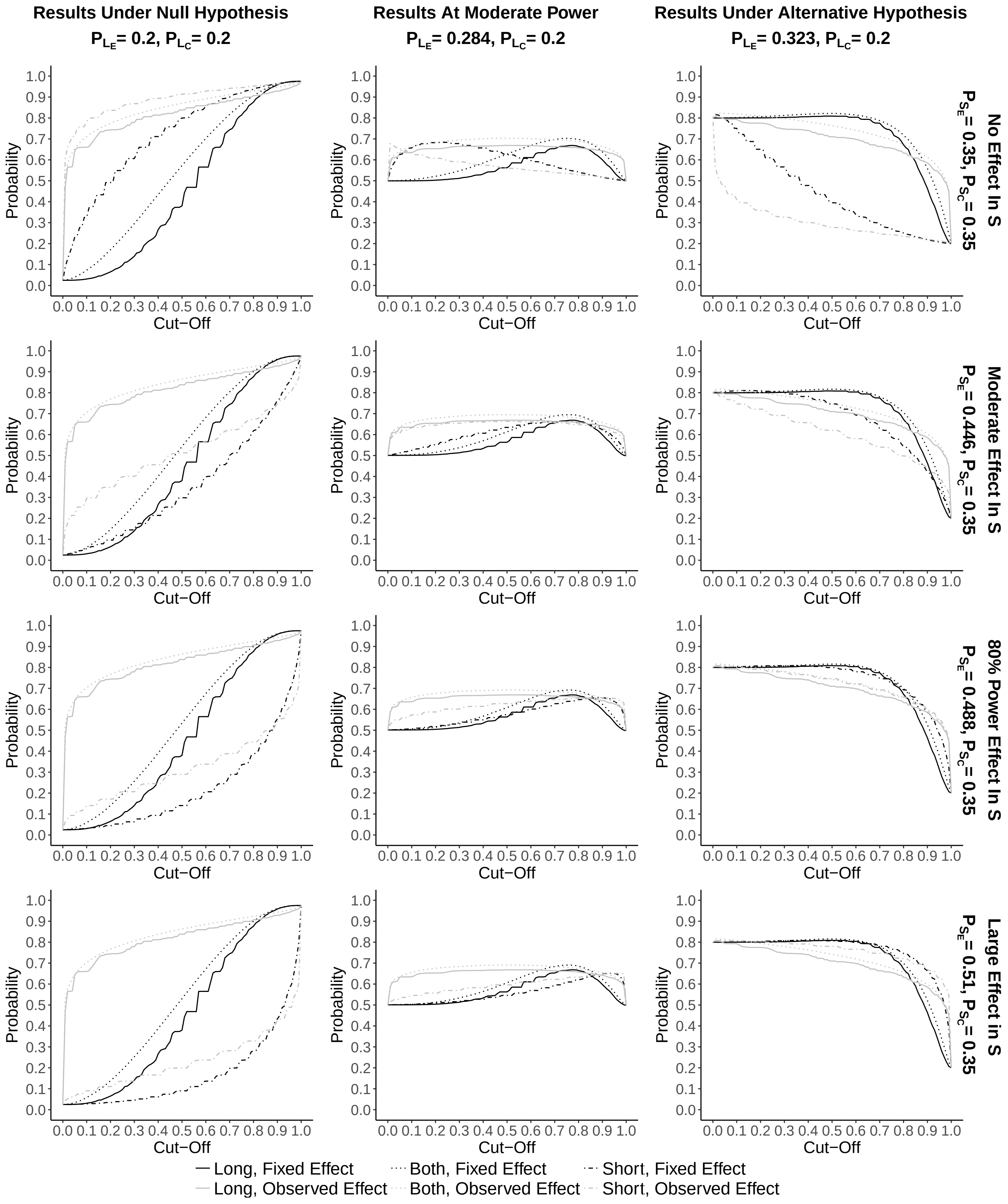

Figure 18: Plots showing the probability of making the correct decision at interim plotted against cut-off points for different effect sizes in P_{S_E} for nested endpoints. First column corresponds to the results under the null hypothesis, middle column to the simulations at moderate power and right to the simulations under alternative hypothesis. The rows correspond to no effect, moderate effect, effect equal to 80% power compared to P_{S_C}, and a high effect in P_{S_E} respectively. Grey lines correspond to observed effect conditional power, $CP_{\hat{\theta}}$, whereas black to fixed effect conditional power, CP_{θ_D}. $\hat{P}_{\mathbf{B}}^{(1)}$ is denoted by dotted lines, $\hat{P}_{\mathbf{L}}^{(1)}$ by solid and $\hat{P}_{\mathbf{S}}^{(1)}$ by dot-dashed.

2.4 Varying Correlation and Information Fraction and Cut-Off Point Thresholds

In Figure 19 below, we can see how changing the probability of success for the short-term endpoint S influences the overall power of the trial. In this scenario, we decided to have a look at the overall power, when our cut-off point values for $\hat{P}_{\mathbf{S}}^{(1)}$ and $\hat{P}_{\mathbf{B}}^{(1)}$ were chosen assuming equal probabilities of success for both, short- and long-term endpoints, either assuming no correlation between the endpoints or a high one. So P_{SE} was set to $(0.2, 0.28, 0.323, 0.365)$ which corresponds to no effect, moderate effect, alternative hypothesis effect and a very large effect. In the case of no effect the maximum correlation between P_{SE} and P_{LE} was just over 0.7, so the maximum plotted value for the correlation was set to 0.7. For $\hat{P}_{\mathbf{L}}^{(1)}$ the corresponding cut-off point for futility stopping was set to 0.61. For $\hat{P}_{\mathbf{S}}^{(1)}$ it was equal to 0.46 for $t_S = 0.5$ and to 0.31 for $t_S = 0.75$. For $\hat{P}_{\mathbf{B}}^{(1)}$ we considered two cut-off points: assuming no correlation, and assuming that the endpoints are highly correlated. So the resulting values were: 0.59 assuming no correlation and 0.47 assuming high correlation for $t_S = 0.5$, and 0.59 assuming no correlation and 0.34 assuming high for $t_S = 0.75$.

```
phi_e <- c(0,0.2,0.5,0.7,0.9)
phi_c <- c(0,0.2,0.5,0.7,0.9)

m <- length(phi_e) # if multiple cores are available set this value to length of phi_e

cl <- parallel::makeCluster(m)
doParallel::registerDoParallel(cl)


output_cp_alt_fr_sh_05 <- foreach(i = 1:length(phi_e), .packages="binfutssr") %dopar%
  (cp(nsim = 100000,
      alpha = 0.025,
      beta = 0.2,
      p_le = 0.3227348,
      p_lc = 0.2,
      p_se = 0.3227348,
      p_sc = 0.2,
      n = 200,
      fr_lo = 0.25,
      fr_sh = 0.5,
      phi_e = phi_e[i],
      phi_c = phi_c[i],
      c = seq(0, 1, 0.01)))

output_cp_alt_fr_sh_075 <- foreach(i = 1:length(phi_e), .packages="binfutssr") %dopar%
  (cp(nsim = 100000,
      alpha = 0.025,
      beta = 0.2,
      p_le = 0.3227348,
      p_lc = 0.2,
      p_se = 0.3227348,
      p_sc = 0.2,
      n = 200,
      fr_lo = 0.25,
      fr_sh = 0.75,
      phi_e = phi_e[i],
      phi_c = phi_c[i],
      c = seq(0, 1, 0.01)))

plots_corr <- create_corr_plots(output_cp_alt_fr_sh_05 = output_cp_alt_fr_sh_05,
                                output_cp_alt_fr_sh_075 = output_cp_alt_fr_sh_075,
                                phi_e = phi_e,
                                phi_c = phi_c,
                                fr_lo = 0.25,
                                filetype = "pdf")
```

```
phi_e_v2 <- c(0, 0.2, 0.5, 0.7,
              0, 0.2, 0.5, 0.7, 0.9,
              0, 0.2, 0.5, 0.7, 0.9,
              0, 0.2, 0.5, 0.7, 0.9)
phi_c_v2 <- c(0, 0.2, 0.5, 0.7,
              0, 0.2, 0.5, 0.7, 0.9,
              0, 0.2, 0.5, 0.7, 0.9,
              0, 0.2, 0.5, 0.7, 0.9)
p_se <- c(rep(0.2, 4), rep(0.285, 5),
          rep(0.3227348 ,5), rep(0.365, 5))

output_cp_alt_fr_sh_05_p_se <- foreach(i = 1:length(phi_e_v2),
                                       .packages= c("binfutssr","psych","mvtnorm")) %dopar%
  (cp(nsim = 100000,
      alpha = 0.025,
      beta = 0.2,
      p_le = 0.3227348,
      p_lc = 0.2,
      p_se = p_se[i],
      p_sc = 0.2,
      n = 200,
      fr_lo = 0.25,
      fr_sh = 0.5,
      phi_e = phi_e_v2[i],
      phi_c = phi_c_v2[i],
      c = seq(0, 1, 0.01)))

output_cp_alt_fr_sh_075_p_se <- foreach(i = 1:length(phi_e_v2),
                                        .packages= c("binfutssr","psych","mvtnorm")) %dopar%
  (cp(nsim = 100000,
      alpha = 0.025,
```

```r
      beta = 0.2,
      p_le = 0.3227348,
      p_lc = 0.2,
      p_se = p_se[i],
      p_sc = 0.2,
      n = 200,
      fr_lo = 0.25,
      fr_sh = 0.75,
      phi_e = phi_e_v2[i],
      phi_c = phi_c_v2[i],
      c = seq(0, 1, 0.01)))
```

2.4.1 Overall Power

```r
corr_plots_p_se <- create_corr_plots_p_se(
  output_cp_alt_fr_sh_05_p_se = output_cp_alt_fr_sh_05_p_se,
  output_cp_alt_fr_sh_075_p_se = output_cp_alt_fr_sh_075_p_se,
  plots_corr = plots_corr,
  phi_e = phi_e_v2,
  phi_c = phi_c_v2,
  fr_lo = 0.25,
  filetype = "none")
```

```r
grid.arrange(corr_plots_p_se)
```

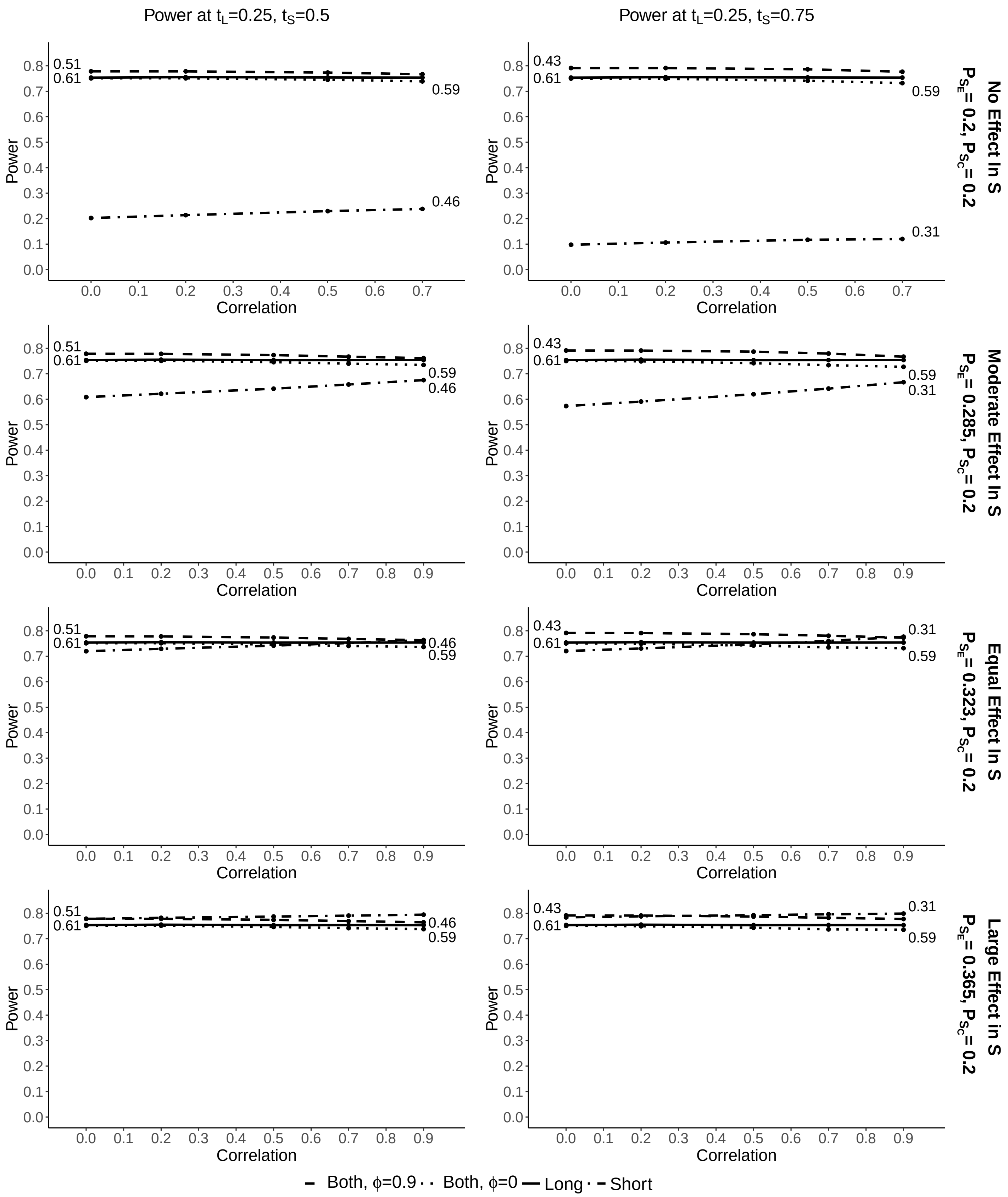

Figure 19: Overall power plotted against correlations for fixed effect conditional power for different correlations for different effects in the short-term outcome, $P_{S_E} = (0.2, 0.285, 0.323, 0.365)$. The cut-off points at which the probability occurs are denoted by the numbers above the lines. Left column shows results at $t_L = 0.25$ and $t_S = 0.5$ and right column at $t_L = 0.25$ and $t_S = 0.75$. $\hat{P}_{\mathbf{B}}^{(1)}$ is denoted by dotted lines, $\hat{P}_{\mathbf{L}}^{(1)}$ by solid and $\hat{P}_{\mathbf{S}}^{(1)}$ by dot-dashed.

2.4.2 Probability to Reject the Null Hypothesis Given Trial Was Continued

```
corr_plots_prob_cont_rej_p_se <- create_corr_plots_prob_cont_rej_p_se(
  output_cp_alt_fr_sh_05_p_se = output_cp_alt_fr_sh_05_p_se,
  output_cp_alt_fr_sh_075_p_se = output_cp_alt_fr_sh_075_p_se,
  plots_corr = plots_corr,
  phi_e = phi_e_v2,
  phi_c = phi_c_v2,
  fr_lo = 0.25,
  filetype = "none")
```

```
grid.arrange(corr_plots_prob_cont_rej_p_se)
```

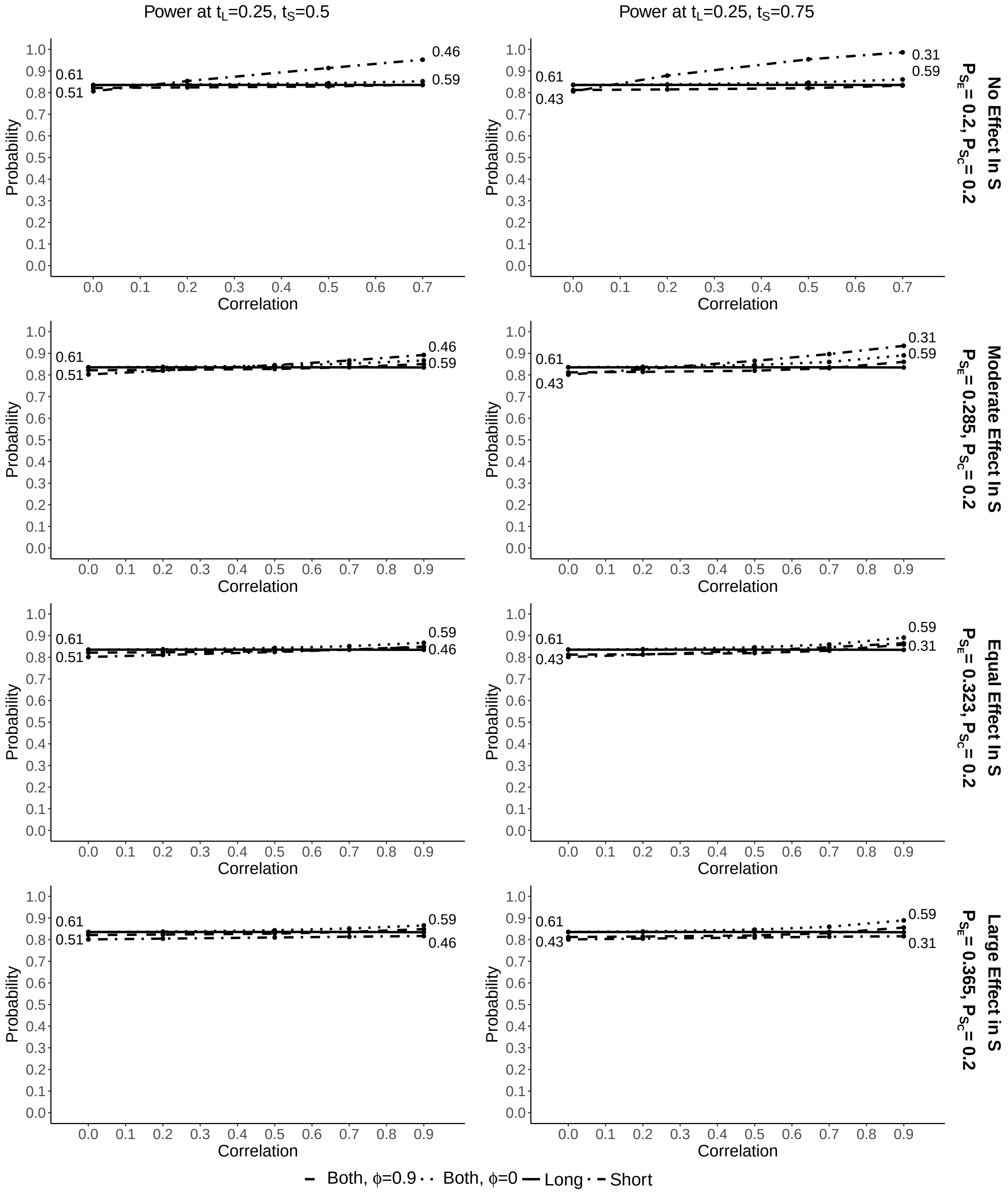

Figure 20: Probability to reject the null hypothesis at the end of the trial given the trial was continued plotted against correlations for fixed effect conditional power for different correlations for different effects in the short-term outcome, $P_{S_E} = (0.2, 0.285, 0.323, 0.365)$. The cut-off points at which the probability occurs are denoted by the numbers above the lines. Left column shows results at $t_L = 0.25$ and $t_S = 0.5$ and right column at $t_L = 0.25$ and $t_S = 0.75$. $\hat{P}_{\mathbf{B}}^{(1)}$ is denoted by dotted lines, $\hat{P}_{\mathbf{L}}^{(1)}$ by solid and $\hat{P}_{\mathbf{S}}^{(1)}$ by dot-dashed.

2.4.3 Probability to Fail to Reject the Null Hypothesis Given The Trial Was Stopped Had it Been Continued

```
corr_plots_prob_stop_not_p_se <- create_corr_plots_prob_stop_not_p_se(
  output_cp_alt_fr_sh_05_p_se = output_cp_alt_fr_sh_05_p_se,
  output_cp_alt_fr_sh_075_p_se = output_cp_alt_fr_sh_075_p_se,
  plots_corr = plots_corr,
  phi_e = phi_e_v2,
  phi_c = phi_c_v2,
  fr_lo = 0.25,
  filetype = "none")
```

```
grid.arrange(corr_plots_prob_stop_not_p_se)
```

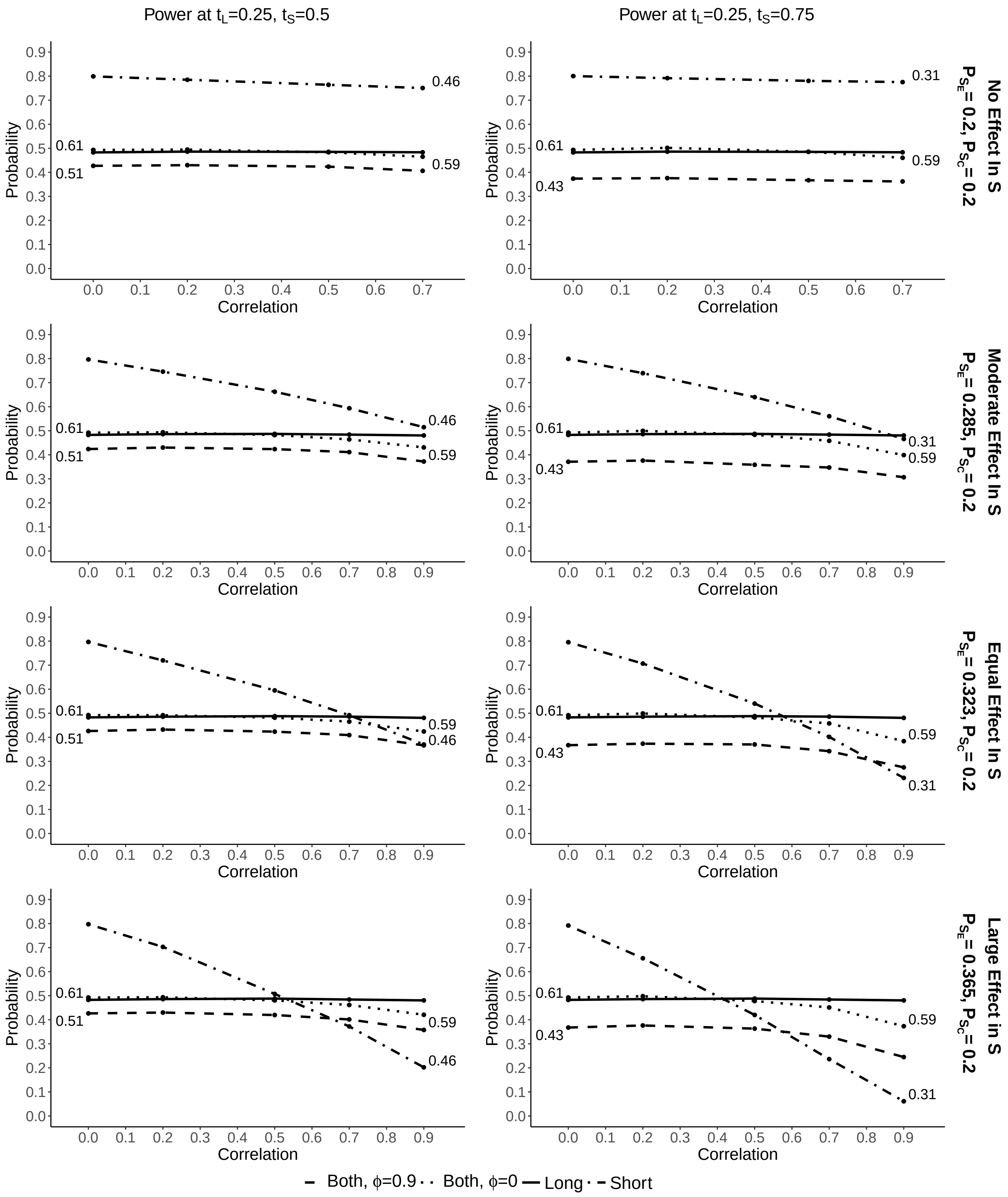

Figure 21: Probability that we would have failed to reject the null hypothesis given the trial was stopped for futility, had it been continues plotted against correlations for fixed effect conditional power for different correlations for different effects in the short-term outcome, $P_{S_E} = (0.2, 0.285, 0.323, 0.365)$. The cut-off points at which the probability occurs are denoted by the numbers above the lines. Left column shows results at $t_L = 0.25$ and $t_S = 0.5$ and right column at $t_L = 0.25$ and $t_S = 0.75$. $\hat{P}^{(1)}_{\mathbf{B}}$ is denoted by dotted lines, $\hat{P}^{(1)}_{\mathbf{L}}$ by solid and $\hat{P}^{(1)}_{\mathbf{S}}$ by dot-dashed.

2.4.4 Probability to Make the Correct Decision

```
corr_plots_prob_corr_dec_p_se <- create_corr_plots_prob_corr_dec_p_se(
  output_cp_alt_fr_sh_05_p_se = output_cp_alt_fr_sh_05_p_se,
  output_cp_alt_fr_sh_075_p_se = output_cp_alt_fr_sh_075_p_se,
  plots_corr = plots_corr,
  phi_e = phi_e_v2,
  phi_c = phi_c_v2,
  fr_lo = 0.25,
  filetype = "none")
```

```
grid.arrange(corr_plots_prob_corr_dec_p_se)
```

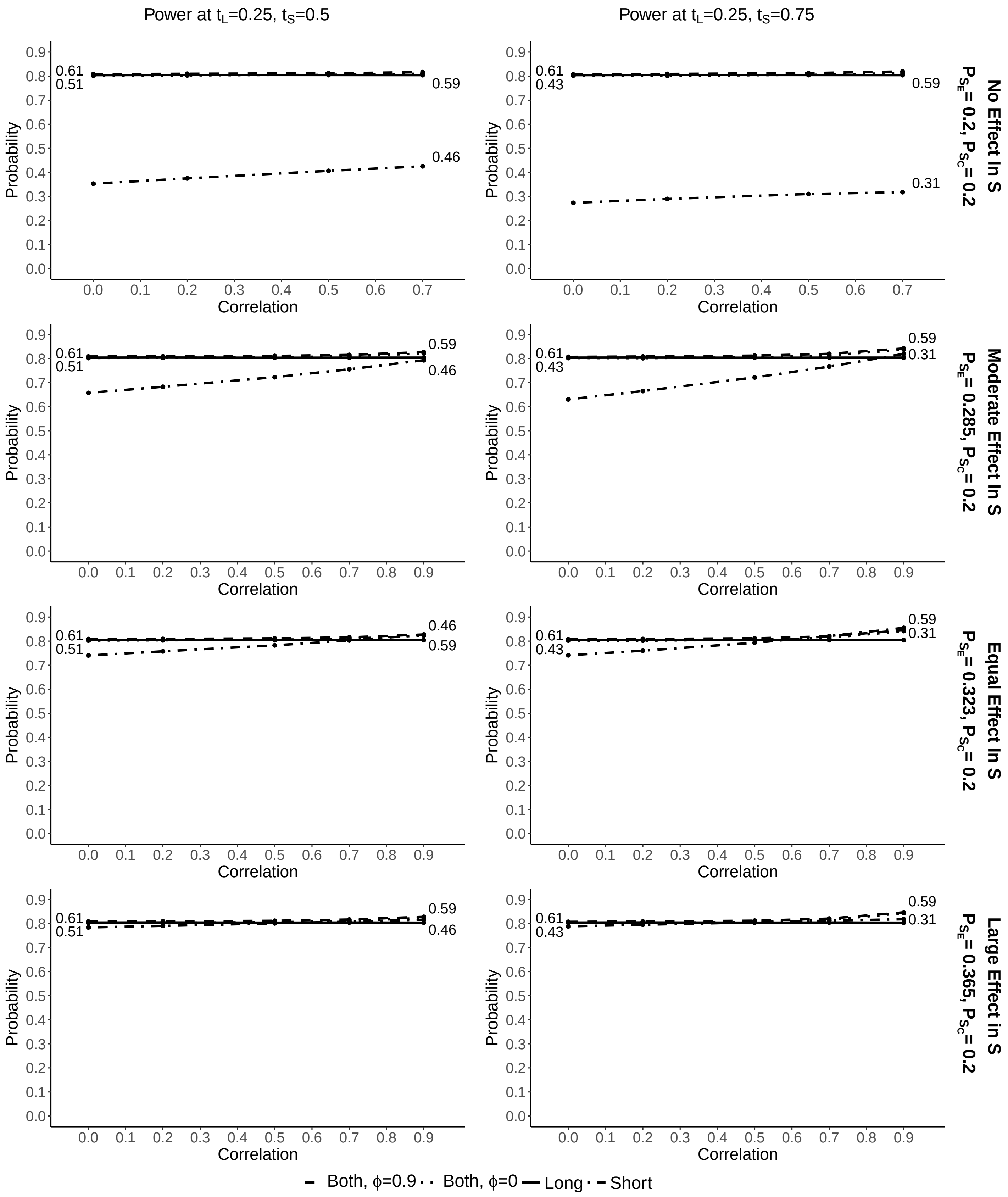

Figure 22: Probability of making the correct decision plotted against correlations for fixed effect conditional power for different correlations for different effects in the short-term outcome, $P_{S_E} = (0.2, 0.285, 0.323, 0.365)$. The cut-off points at which the probability occurs are denoted by the numbers above the lines. Left column shows results at $t_L = 0.25$ and $t_S = 0.5$ and right column at $t_L = 0.25$ and $t_S = 0.75$. $\hat{P}^{(1)}_{\mathbf{B}}$ is denoted by dotted lines, $\hat{P}^{(1)}_{\mathbf{L}}$ by solid and $\hat{P}^{(1)}_{\mathbf{S}}$ by dot-dashed.

3 Sample Size Reassessment Further Simulation Results

Here, we also considered a scenario when the effect in P_{S_E} was constant with changing effect in P_{L_E}. We considered 4 scenarios: P_{S_E}=(0.2,0.285,0.323,0.365) under four scenarios for the long-term outcome: P_{L_E}=(0.2,0.285,0.323,0.365). Tables below summarise the operating characteristics of such trials. Sample size reassessment for both fixed and observed effects were considered.

3.1 Fixed Effect

3.1.1 $P_{S_E} = 0.2$

		Probability of Success			
	$\mathbf{P_{L_E}}$	0.2	0.285	0.323	0.365
	$\mathbf{P_{S_E}}$	0.2	0.2	0.2	0.2
	**Power Single Stage Trial**	0.0255	0.5108	0.8016	0.9594
**Long**	Probability to Stop for Futility	0.1163	0.0132	0.0041	0.001
	Power SSR	0.0248	0.5506	0.8214	0.9549
	ASN SSR	262 (±88)	222 (±66)	200 (±57)	181 (±45)
	Power NO SSR	0.0254	0.5098	0.8003	0.9589
	ASN NO SSR	183 (±49)	199 (±18)	200 (±10)	200 (±5)
**Both** t_B	Probability to Stop for Futility	0.1895	0.0253	0.008	0.0017
	Power SSR	0.0255	0.6094	0.8542	0.9594
	ASN SSR	285 (±110)	259 (±76)	234 (±68)	207 (±58)
	Power NO SSR	0.0253	0.5086	0.7997	0.9585
**Both** t_S	Probability to Stop for Futility	0.6017	0.2137	0.1078	0.043
	Power SSR	0.0217	0.521	0.7857	0.9287
	ASN SSR	174 (±100)	210 (±85)	205 (±74)	190 (±61)
	Power NO SSR	0.0221	0.4646	0.7505	0.9265
**Short**	Probability to Stop for Futility	0.6071	0.6067	0.6068	0.6066
	Power SSR	0.018	0.3052	0.3774	0.3924
	ASN SSR	174 (±100)	174 (±100)	174 (±100)	174 (±100)
	Power NO SSR	0.0183	0.2561	0.3513	0.3876

Table 2: Operating characteristics of a trial with sample size reassessment based on fixed effect conditional power with $c = 0.3$ as a futility stopping rule: overall power, probability to stop for futility, and average sample size per treatment arm over both stages (ASN) and its standard deviation (in brackets).

	Probability of Success			
$\mathbf{P_{L_E}}$	0.2	0.285	0.323	0.365
$\mathbf{P_{S_E}}$	0.2	0.2	0.2	0.2
**Power Single Stage Trial**	0.0255	0.5108	0.8016	0.9594
**Stopping Rule With The P-value Based On** $\hat{P}_L$				
Probability to Stop for Futility	0.5484	0.1885	0.0974	0.0396
Power NO SSR	0.0225	0.4715	0.7556	0.9286
ASN NO SSR	118 (±75)	172 (±59)	186 (±45)	195 (±30)
**Long** Power SSR	0.0216	0.4703	0.7475	0.9176
ASN SSR	157 (±68)	181 (±54)	179 (±46)	172 (±37)
**Both** t_B Power SSR	0.0208	0.5102	0.7721	0.9219
ASN SSR	183 (±98)	215 (±75)	210 (±63)	197 (±53)
**Both** t_S Power SSR	0.0225	0.5379	0.7938	0.9322
ASN SSR	195 (±120)	220 (±95)	210 (±81)	192 (±65)
**Short** Power SSR	0.0227	0.6908	0.8867	0.9596
ASN SSR	237 (±186)	374 (±206)	413 (±199)	439 (±192)
**Stopping Rule With The P-value Based On** $\hat{P}_B$				
Probability to Stop for Futility	0.549	0.175	0.0839	0.0319
Power NO SSR	0.023	0.4778	0.7654	0.9356
ASN NO SSR	118 (±75)	174 (±57)	188 (±42)	196 (±27)
**Long** Power SSR	0.0223	0.4828	0.7611	0.9255
ASN SSR	160 (±73)	186 (±58)	183 (±49)	174 (±40)
**Both** t_B Power SSR	0.021	0.5172	0.7844	0.9293
ASN SSR	180 (±93)	216 (±72)	212 (±61)	199 (±52)
**Both** t_S Power SSR	0.0231	0.5456	0.8072	0.9401
ASN SSR	190 (±110)	221 (±90)	212 (±78)	194 (±64)
**Short** Power SSR	0.023	0.7031	0.8996	0.9672
ASN SSR	235 (±184)	378 (±204)	418 (±197)	443 (±191)
**Stopping Rule With The P-value Based On** $\hat{P}_S$				
Probability to Stop for Futility	0.5358	0.5359	0.5359	0.5357
Power NO SSR	0.0198	0.2943	0.4109	0.4564
ASN NO SSR	120 (±75)	120 (±75)	120 (±75)	120 (±75)
**Long** Power SSR	0.0191	0.2998	0.4093	0.4535
ASN SSR	183 (±105)	150 (±67)	140 (±54)	133 (±42)
**Both** t_B Power SSR	0.0183	0.3108	0.408	0.4484
ASN SSR	209 (±133)	167 (±86)	154 (±70)	142 (±55)
**Both** t_S Power SSR	0.0195	0.3349	0.4238	0.4552
ASN SSR	240 (±188)	172 (±107)	153 (±81)	139 (±58)
**Short** Power SSR	0.0198	0.3617	0.447	0.4633
ASN SSR	195 (±114)	195 (±114)	195 (±114)	195 (±114)

Table 3: Operating characteristics of a trial with sample size reassessment based on fixed effect conditional power: overall power, probability to stop for futility, and average sample size per treatment arm over both stages (ASN) and its standard deviation (in brackets). Simulations with 3 different interim stopping approaches are shown: results with a p-value based on Z_L, second one with a p-value based on Z_B and the last one with p-value based on Z_S as a stopping rule.

3.1.2 $P_{S_E} = 0.285$

		Probability of Success			
	$\mathbf{P_{L_E}}$	0.2	0.285	0.323	0.365
	$\mathbf{P_{S_E}}$	0.285	0.285	0.285	0.285
	**Power Single Stage Trial**	0.0255	0.5112	0.8011	0.9591
**Long**	Probability to Stop for Futility	0.1161	0.0131	0.0041	9e-04
	Power SSR	0.0248	0.5506	0.8224	0.9544
	ASN SSR	262 (±88)	222 (±66)	200 (±57)	181 (±45)
	Power NO SSR	0.0254	0.5101	0.8	0.9586
	ASN NO SSR	183 (±49)	199 (±18)	200 (±10)	200 (±5)
**Both** t_B	Probability to Stop for Futility	0.1888	0.0251	0.0079	0.0019
	Power SSR	0.0248	0.6088	0.8538	0.9593
	ASN SSR	285 (±110)	259 (±76)	234 (±68)	207 (±57)
	Power NO SSR	0.0253	0.5088	0.7992	0.9581
**Both** t_S	Probability to Stop for Futility	0.6017	0.2128	0.1078	0.0428
	Power SSR	0.0216	0.5205	0.7852	0.9295
	ASN SSR	174 (±100)	210 (±85)	205 (±74)	190 (±61)
	Power NO SSR	0.0222	0.4654	0.7502	0.9265
**Short**	Probability to Stop for Futility	0.1219	0.122	0.1219	0.1219
	Power SSR	0.0251	0.5042	0.7542	0.8575
	ASN SSR	209 (±77)	209 (±77)	209 (±77)	209 (±77)
	Power NO SSR	0.0249	0.4781	0.727	0.8505

Table 4: Operating characteristics of a trial with sample size reassessment based on fixed effect conditional power with $c = 0.3$ as a futility stopping rule: overall power, probability to stop for futility, and average sample size per treatment arm over both stages (ASN) and its standard deviation (in brackets).

		Probability of Success			
	$\mathbf{P_{L_E}}$	0.2	0.285	0.323	0.365
	$\mathbf{P_{S_E}}$	0.285	0.285	0.285	0.285
	**Power Single Stage Trial**	0.0255	0.5112	0.8011	0.9591
**Stopping Rule With The P-value Based On** $\hat{P}_L$					
	Probability to Stop for Futility	0.5482	0.1885	0.0978	0.0394
	Power NO SSR	0.0224	0.4714	0.7548	0.9286
	ASN NO SSR	118 (±75)	172 (±59)	186 (±45)	195 (±30)
**Long**	Power SSR	0.0215	0.471	0.747	0.9167
	ASN SSR	157 (±68)	181 (±54)	179 (±46)	172 (±37)
**Both** t_B	Power SSR	0.0202	0.5097	0.772	0.9225
	ASN SSR	183 (±98)	215 (±75)	210 (±63)	197 (±52)
**Both** t_S	Power SSR	0.0225	0.537	0.7952	0.9328
	ASN SSR	195 (±120)	220 (±95)	210 (±80)	192 (±65)
**Short**	Power SSR	0.0227	0.517	0.7988	0.9419
	ASN SSR	156 (±86)	216 (±108)	234 (±110)	246 (±111)
**Stopping Rule With The P-value Based On** $\hat{P}_B$					
	Probability to Stop for Futility	0.549	0.1747	0.0841	0.0312
	Power NO SSR	0.0229	0.4781	0.7645	0.936
	ASN NO SSR	118 (±75)	174 (±57)	188 (±42)	196 (±27)
**Long**	Power SSR	0.0216	0.4824	0.7603	0.9256
	ASN SSR	160 (±73)	186 (±58)	183 (±49)	175 (±40)
**Both** t_B	Power SSR	0.021	0.5181	0.7846	0.93
	ASN SSR	180 (±93)	216 (±72)	212 (±61)	199 (±52)
**Both** t_S	Power SSR	0.023	0.5448	0.807	0.9412
	ASN SSR	190 (±110)	221 (±90)	212 (±78)	194 (±64)
**Short**	Power SSR	0.0231	0.5236	0.8112	0.9499
	ASN SSR	155 (±85)	218 (±108)	236 (±110)	248 (±111)
**Stopping Rule With The P-value Based On** $\hat{P}_S$					
	Probability to Stop for Futility	0.0915	0.0923	0.0922	0.0923
	Power NO SSR	0.0248	0.489	0.7482	0.8781
	ASN NO SSR	187 (±44)	187 (±44)	187 (±44)	187 (±44)
**Long**	Power SSR	0.0241	0.5204	0.7602	0.872
	ASN SSR	281 (±103)	211 (±73)	189 (±60)	171 (±47)
**Both** t_B	Power SSR	0.0251	0.5751	0.785	0.8732
	ASN SSR	335 (±122)	248 (±88)	220 (±75)	194 (±61)
**Both** t_S	Power SSR	0.0245	0.6076	0.8095	0.8843
	ASN SSR	415 (±201)	268 (±131)	225 (±104)	191 (±77)
**Short**	Power SSR	0.0255	0.5241	0.7827	0.8863
	ASN SSR	218 (±81)	218 (±81)	218 (±81)	218 (±81)

Table 5: Operating characteristics of a trial with sample size reassessment based on fixed effect conditional power: overall power, probability to stop for futility, and average sample size per treatment arm over both stages (ASN) and its standard deviation (in brackets). Simulations with 3 different interim stopping approaches are shown: results with a p-value based on Z_L, second one with a p-value based on Z_B and the last one with p-value based on Z_S as a stopping rule.

3.1.3 $P_{S_E} = 0.323$

		Probability of Success			
	$\mathbf{P_{L_E}}$	0.2	0.285	0.323	0.365
	$\mathbf{P_{S_E}}$	0.323	0.323	0.323	0.323
	**Power Single Stage Trial**	0.0255	0.5113	0.8014	0.9593
**Long**	Probability to Stop for Futility	0.1165	0.0131	0.0041	9e-04
	Power SSR	0.0249	0.5506	0.822	0.9547
	ASN SSR	262 (±88)	222 (±66)	200 (±57)	181 (±45)
	Power NO SSR	0.0254	0.5102	0.8002	0.9588
	ASN NO SSR	183 (±49)	199 (±18)	200 (±10)	200 (±5)
**Both** t_B	Probability to Stop for Futility	0.1879	0.0249	0.008	0.0019
	Power SSR	0.0248	0.609	0.8527	0.9598
	ASN SSR	286 (±110)	259 (±76)	234 (±68)	207 (±57)
	Power NO SSR	0.0253	0.509	0.7994	0.9583
**Both** t_S	Probability to Stop for Futility	0.6014	0.2134	0.108	0.0425
	Power SSR	0.0221	0.5203	0.7846	0.9294
	ASN SSR	174 (±100)	209 (±85)	205 (±74)	190 (±61)
	Power NO SSR	0.0223	0.4653	0.7501	0.9268
**Short**	Probability to Stop for Futility	0.0399	0.0402	0.0403	0.0403
	Power SSR	0.0257	0.477	0.7628	0.9165
	ASN SSR	191 (±61)	191 (±61)	191 (±61)	191 (±61)
	Power NO SSR	0.0254	0.5025	0.7797	0.9249

Table 6: Operating characteristics of a trial with sample size reassessment based on fixed effect conditional power with $c = 0.3$ as a futility stopping rule: overall power, probability to stop for futility, and average sample size per treatment arm over both stages (ASN) and its standard deviation (in brackets).

		Probability of Success			
	$\mathbf{P_{L_E}}$	0.2	0.285	0.323	0.365
	$\mathbf{P_{S_E}}$	0.323	0.323	0.323	0.323
	**Power Single Stage Trial**	0.0255	0.5113	0.8014	0.9593
**Stopping Rule With The P-value Based On** $\hat{P}_L$					
	Probability to Stop for Futility	0.5481	0.1885	0.098	0.0396
	Power NO SSR	0.0226	0.4713	0.7548	0.9285
	ASN NO SSR	118 (±75)	172 (±59)	186 (±45)	195 (±30)
**Long**	Power SSR	0.0216	0.4711	0.7456	0.9175
	ASN SSR	157 (±68)	181 (±54)	179 (±46)	172 (±37)
**Both** t_B	Power SSR	0.0198	0.5106	0.7721	0.9223
	ASN SSR	183 (±98)	215 (±75)	210 (±63)	197 (±52)
**Both** t_S	Power SSR	0.0223	0.5382	0.7943	0.9324
	ASN SSR	195 (±120)	220 (±95)	210 (±80)	192 (±65)
**Short**	Power SSR	0.0227	0.4501	0.7425	0.9226
	ASN SSR	139 (±58)	179 (±75)	191 (±77)	199 (±79)
**Stopping Rule With The P-value Based On** $\hat{P}_B$					
	Probability to Stop for Futility	0.548	0.1748	0.0843	0.0312
	Power NO SSR	0.0229	0.4778	0.765	0.9362
	ASN NO SSR	118 (±75)	174 (±57)	188 (±42)	196 (±27)
**Long**	Power SSR	0.0223	0.4823	0.7598	0.9257
	ASN SSR	160 (±73)	186 (±58)	183 (±49)	174 (±40)
**Both** t_B	Power SSR	0.021	0.5171	0.7833	0.9305
	ASN SSR	180 (±93)	216 (±72)	212 (±61)	199 (±52)
**Both** t_S	Power SSR	0.023	0.5463	0.8058	0.9408
	ASN SSR	190 (±110)	221 (±90)	212 (±78)	194 (±64)
**Short**	Power SSR	0.0231	0.4557	0.753	0.9303
	ASN SSR	138 (±57)	180 (±75)	192 (±77)	200 (±79)
**Stopping Rule With The P-value Based On** $\hat{P}_S$					
	Probability to Stop for Futility	0.0278	0.0279	0.028	0.028
	Power NO SSR	0.0252	0.5066	0.7882	0.9358
	ASN NO SSR	196 (±25)	196 (±25)	196 (±25)	196 (±25)
**Long**	Power SSR	0.0248	0.5461	0.8066	0.9306
	ASN SSR	297 (±96)	221 (±71)	198 (±59)	178 (±46)
**Both** t_B	Power SSR	0.0259	0.6099	0.8375	0.9345
	ASN SSR	356 (±111)	262 (±84)	231 (±72)	203 (±59)
**Both** t_S	Power SSR	0.0254	0.6422	0.8618	0.9454
	ASN SSR	445 (±193)	285 (±132)	239 (±106)	201 (±79)
**Short**	Power SSR	0.0256	0.4847	0.7742	0.9289
	ASN SSR	195 (±65)	195 (±65)	195 (±65)	195 (±65)

Table 7: Operating characteristics of a trial with sample size reassessment based on fixed effect conditional power: overall power, probability to stop for futility, and average sample size per treatment arm over both stages (ASN) and its standard deviation (in brackets). Simulations with 3 different interim stopping approaches are shown: results with a p-value based on Z_L, second one with a p-value based on Z_B and the last one with p-value based on Z_S as a stopping rule.

3.1.4 $P_{S_E} = 0.365$

		Probability of Success			
	$\mathbf{P_{L_E}}$	0.2	0.285	0.323	0.365
	$\mathbf{P_{S_E}}$	0.365	0.365	0.365	0.365
	**Power Single Stage Trial**	0.0254	0.5111	0.8014	0.9594
**Long**	Probability to Stop for Futility	0.1163	0.0132	0.0042	9e-04
	Power SSR	0.0249	0.5509	0.8216	0.9547
	ASN SSR	262 (±88)	222 (±66)	200 (±57)	181 (±45)
	Power NO SSR	0.0253	0.51	0.8002	0.9588
	ASN NO SSR	183 (±49)	199 (±18)	200 (±10)	200 (±5)
**Both** t_B	Probability to Stop for Futility	0.1881	0.025	0.0079	0.0018
	Power SSR	0.0254	0.609	0.8523	0.9598
	ASN SSR	286 (±110)	259 (±76)	234 (±68)	207 (±58)
	Power NO SSR	0.0252	0.5088	0.7994	0.9584
**Both** t_S	Probability to Stop for Futility	0.602	0.2126	0.108	0.0428
	Power SSR	0.0217	0.5207	0.7843	0.9292
	ASN SSR	174 (±100)	210 (±85)	204 (±74)	190 (±61)
	Power NO SSR	0.0219	0.4652	0.7502	0.9268
**Short**	Probability to Stop for Futility	0.0084	0.0082	0.0083	0.0082
	Power SSR	0.0256	0.4331	0.7282	0.9199
	ASN SSR	170 (±43)	170 (±43)	170 (±43)	170 (±43)
	Power NO SSR	0.0254	0.5099	0.7977	0.9529

Table 8: Operating characteristics of a trial with sample size reassessment based on fixed effect conditional power with $c = 0.3$ as a futility stopping rule: overall power, probability to stop for futility, and average sample size per treatment arm over both stages (ASN) and its standard deviation (in brackets).

		Probability of Success			
	$\mathbf{P_{L_E}}$	0.2	0.285	0.323	0.365
	$\mathbf{P_{S_E}}$	0.365	0.365	0.365	0.365
	**Power Single Stage Trial**	0.0254	0.5111	0.8014	0.9594
**Stopping Rule With The P-value Based On** $\hat{P}_L$					
	Probability to Stop for Futility	0.5485	0.1885	0.0979	0.0395
	Power NO SSR	0.0224	0.4714	0.7548	0.9288
	ASN NO SSR	118 (±75)	172 (±59)	186 (±45)	195 (±30)
**Long**	Power SSR	0.022	0.4702	0.7467	0.9176
	ASN SSR	157 (±68)	181 (±54)	179 (±46)	172 (±37)
**Both** t_B	Power SSR	0.02	0.5085	0.7724	0.9222
	ASN SSR	182 (±98)	215 (±75)	210 (±63)	197 (±52)
**Both** t_S	Power SSR	0.0223	0.5382	0.7937	0.9324
	ASN SSR	195 (±120)	220 (±95)	209 (±80)	192 (±65)
**Short**	Power SSR	0.0229	0.4016	0.6902	0.898
	ASN SSR	129 (±39)	156 (±47)	163 (±48)	169 (±49)
**Stopping Rule With The P-value Based On** $\hat{P}_B$					
	Probability to Stop for Futility	0.5476	0.1747	0.0843	0.0311
	Power NO SSR	0.0228	0.4779	0.7652	0.9364
	ASN NO SSR	118 (±75)	174 (±57)	188 (±42)	196 (±27)
**Long**	Power SSR	0.0222	0.4819	0.7602	0.9263
	ASN SSR	160 (±73)	186 (±58)	183 (±49)	174 (±40)
**Both** t_B	Power SSR	0.0207	0.5175	0.7833	0.9304
	ASN SSR	180 (±93)	216 (±72)	212 (±61)	199 (±52)
**Both** t_S	Power SSR	0.0232	0.5448	0.8054	0.9408
	ASN SSR	190 (±110)	221 (±90)	212 (±78)	194 (±64)
**Short**	Power SSR	0.0232	0.4071	0.6994	0.905
	ASN SSR	128 (±38)	157 (±47)	165 (±48)	170 (±49)
**Stopping Rule With The P-value Based On** $\hat{P}_S$					
	Probability to Stop for Futility	0.0053	0.0055	0.0054	0.0054
	Power NO SSR	0.0253	0.5115	0.8003	0.9554
	ASN NO SSR	200 (±11)	200 (±12)	200 (±12)	200 (±11)
**Long**	Power SSR	0.025	0.5526	0.8216	0.9506
	ASN SSR	304 (±93)	225 (±70)	201 (±59)	181 (±46)
**Both** t_B	Power SSR	0.0251	0.6226	0.8547	0.9561
	ASN SSR	364 (±106)	267 (±82)	235 (±71)	207 (±59)
**Both** t_S	Power SSR	0.0251	0.6541	0.8799	0.9669
	ASN SSR	456 (±190)	292 (±132)	244 (±107)	205 (±81)
**Short**	Power SSR	0.0255	0.4348	0.7304	0.9231
	ASN SSR	171 (±45)	171 (±44)	171 (±44)	171 (±44)

Table 9: Operating characteristics of a trial with sample size reassessment based on fixed effect conditional power: overall power, probability to stop for futility, and average sample size per treatment arm over both stages (ASN) and its standard deviation (in brackets). Simulations with 3 different interim stopping approaches are shown: results with a p-value based on Z_L, second one with a p-value based on Z_B and the last one with p-value based on Z_S as a stopping rule.

3.2 Sample Size Reassessment Based on Observed Effect Conditional Power

3.2.1 Equal Effects in P_{S_E} and P_{L_E}

		Probability of Success			
	$\mathbf{P_{L_E}}$	0.2	0.285	0.323	0.365
	$\mathbf{P_{S_E}}$	0.2	0.285	0.323	0.365
	**Power Single Stage Trial**	0.0255	0.5112	0.8014	0.9594
**Long**	Probability to Stop for Futility	0.7704	0.4109	0.2613	0.1388
	Power SSR	0.0167	0.3981	0.6359	0.8244
	ASN SSR	147 (±115)	177 (±113)	179 (±101)	175 (±86)
	Power NO SSR	0.0172	0.3816	0.6447	0.8407
	ASN NO SSR	85 (±64)	139 (±74)	161 (±66)	180 (±52)
**Both** t_B	Probability to Stop for Futility	0.7845	0.3961	0.2408	0.1192
	Power SSR	0.0143	0.4066	0.6368	0.8248
	ASN SSR	152 (±125)	201 (±140)	204 (±128)	193 (±107)
	Power NO SSR	0.0175	0.3944	0.6637	0.86
**Both** t_S	Probability to Stop for Futility	0.8629	0.5172	0.3463	0.1927
	Power SSR	0.0138	0.3285	0.568	0.776
	ASN SSR	118 (±52)	148 (±68)	157 (±64)	160 (±54)
	Power NO SSR	0.014	0.3351	0.585	0.792
**Short**	Probability to Stop for Futility	0.8696	0.3851	0.1924	0.0693
	Power SSR	0.0094	0.3891	0.6574	0.8653
	ASN SSR	129 (±88)	186 (±115)	186 (±97)	172 (±69)
	Power NO SSR	0.0098	0.369	0.6775	0.8988

Table 10: Operating characteristics of a trial with sample size reassessment based on observed effect conditional power with $c = 0.3$ as a futility stopping rule: overall power, probability to stop for futility, and average sample size per treatment arm over both stages (ASN) and its standard deviation (in brackets).

		Probability of Success		
	$\mathbf{P_{L_E}}$	0.2	0.285	0.323	0.365
	$\mathbf{P_{S_E}}$	0.2	0.285	0.323	0.365
	**Power Single Stage Trial**	0.0255	0.5112	0.8014	0.9594
**Stopping Rule With The P-value Based On $\hat{P}_L$**					
	Probability to Stop for Futility	0.5484	0.1885	0.098	0.0395
	Power NO SSR	0.0225	0.4714	0.7548	0.9288
	ASN NO SSR	118 (±75)	172 (±59)	186 (±45)	195 (±30)
**Long**	Power SSR	0.0216	0.6187	0.799	0.9237
	ASN SSR	374 (±424)	393 (±389)	337 (±343)	271 (±281)
**Both t_B**	Power SSR	0.0202	0.6124	0.7797	0.9048
	ASN SSR	400 (±438)	415 (±393)	350 (±341)	274 (±270)
**Both t_S**	Power SSR	0.0225	0.6417	0.8156	0.9289
	ASN SSR	407 (±446)	423 (±404)	355 (±354)	276 (±281)
**Short**	Power SSR	0.0214	0.6286	0.7765	0.9029
	ASN SSR	501 (±504)	446 (±424)	325 (±336)	223 (±218)
**Stopping Rule With The P-value Based On $\hat{P}_B$**					
	Probability to Stop for Futility	0.549	0.1747	0.0843	0.0311
	Power NO SSR	0.023	0.4781	0.765	0.9364
	ASN NO SSR	118 (±75)	174 (±57)	188 (±42)	196 (±27)
**Long**	Power SSR	0.0211	0.633	0.8126	0.9322
	ASN SSR	377 (±428)	410 (±401)	353 (±358)	281 (±294)
**Both t_B**	Power SSR	0.0211	0.6266	0.7935	0.9132
	ASN SSR	397 (±435)	427 (±398)	362 (±350)	281 (±279)
**Both t_S**	Power SSR	0.0226	0.6554	0.8295	0.9374
	ASN SSR	404 (±443)	435 (±410)	368 (±363)	284 (±290)
**Short**	Power SSR	0.0218	0.6388	0.7873	0.9098
	ASN SSR	499 (±503)	452 (±425)	329 (±338)	225 (±220)
**Stopping Rule With The P-value Based On $\hat{P}_S$**					
	Probability to Stop for Futility	0.5358	0.0923	0.028	0.0054
	Power NO SSR	0.0198	0.489	0.7882	0.9554
	ASN NO SSR	120 (±75)	187 (±44)	196 (±25)	200 (±11)
**Long**	Power SSR	0.0182	0.7194	0.8714	0.9582
	ASN SSR	429 (±459)	515 (±446)	420 (±403)	309 (±326)
**Both t_B**	Power SSR	0.0174	0.7159	0.8514	0.9388
	ASN SSR	452 (±469)	538 (±444)	432 (±399)	312 (±315)
**Both t_S**	Power SSR	0.0196	0.7438	0.8871	0.9633
	ASN SSR	469 (±483)	550 (±456)	440 (±410)	315 (±326)
**Short**	Power SSR	0.0201	0.6749	0.8217	0.9293
	ASN SSR	472 (±482)	472 (±416)	341 (±336)	229 (±221)

Table 11: Operating characteristics of a trial with sample size reassessment based on observed effect conditional power: overall power, probability to stop for futility, and average sample size per treatment arm over both stages (ASN) and its standard deviation (in brackets). Simulations with 3 different interim stopping approaches are shown: results with a p-value based on Z_L, second one with a p-value based on Z_B and the last one with p-value based on Z_S as a stopping rule.

3.2.2 $P_{S_E} = 0.2$

		Probability of Success			
	$\mathbf{P_{L_E}}$	0.2	0.285	0.323	0.365
	$\mathbf{P_{S_E}}$	0.2	0.2	0.2	0.2
	**Power Single Stage Trial**	0.0255	0.5108	0.8016	0.9594
**Long**	Probability to Stop for Futility	0.7704	0.4106	0.2612	0.1397
	Power SSR	0.0167	0.3993	0.6359	0.8233
	ASN SSR	147 (±115)	177 (±113)	180 (±101)	175 (±85)
	Power NO SSR	0.0172	0.3818	0.6448	0.8397
	ASN NO SSR	85 (±64)	139 (±74)	161 (±66)	180 (±52)
**Both** t_B	Probability to Stop for Futility	0.7845	0.3959	0.2415	0.12
	Power SSR	0.0143	0.405	0.6359	0.8234
	ASN SSR	152 (±125)	201 (±140)	203 (±127)	193 (±107)
	Power NO SSR	0.0175	0.3945	0.6633	0.859
**Both** t_S	Probability to Stop for Futility	0.8629	0.5176	0.3465	0.1924
	Power SSR	0.0138	0.3297	0.5673	0.7759
	ASN SSR	118 (±52)	148 (±68)	157 (±64)	160 (±54)
	Power NO SSR	0.014	0.335	0.585	0.7922
**Short**	Probability to Stop for Futility	0.8696	0.8696	0.87	0.8697
	Power SSR	0.0094	0.1101	0.1258	0.1298
	ASN SSR	129 (±88)	129 (±88)	129 (±88)	129 (±88)
	Power NO SSR	0.0098	0.0957	0.1214	0.1294

Table 12: Operating characteristics of a trial with sample size reassessment based on observed effect conditional power with $c = 0.3$ as a futility stopping rule: overall power, probability to stop for futility, and average sample size per treatment arm over both stages (ASN) and its standard deviation (in brackets).

		Probability of Success			
	$\mathbf{P_{L_E}}$	0.2	0.285	0.323	0.365
	$\mathbf{P_{S_E}}$	0.2	0.2	0.2	0.2
	**Power Single Stage Trial**	0.0255	0.5108	0.8016	0.9594
**Stopping Rule With The P-value Based On** $\hat{P}_L$					
	Probability to Stop for Futility	0.5484	0.1885	0.0974	0.0396
	Power NO SSR	0.0225	0.4715	0.7556	0.9286
	ASN NO SSR	118 (±75)	172 (±59)	186 (±45)	195 (±30)
**Long**	Power SSR	0.0216	0.6176	0.8	0.9233
	ASN SSR	374 (±424)	393 (±389)	337 (±343)	271 (±281)
**Both** t_B	Power SSR	0.0202	0.6127	0.7806	0.9036
	ASN SSR	400 (±438)	415 (±393)	350 (±342)	274 (±271)
**Both** t_S	Power SSR	0.0225	0.6424	0.8154	0.9286
	ASN SSR	407 (±446)	422 (±405)	356 (±354)	277 (±282)
**Short**	Power SSR	0.0214	0.7896	0.8987	0.96
	ASN SSR	501 (±504)	855 (±468)	947 (±411)	1006 (±358)
**Stopping Rule With The P-value Based On** $\hat{P}_B$					
	Probability to Stop for Futility	0.549	0.175	0.0839	0.0319
	Power NO SSR	0.023	0.4778	0.7654	0.9356
	ASN NO SSR	118 (±75)	174 (±57)	188 (±42)	196 (±27)
**Long**	Power SSR	0.0211	0.633	0.8132	0.931
	ASN SSR	377 (±428)	410 (±401)	354 (±359)	281 (±294)
**Both** t_B	Power SSR	0.0211	0.6246	0.7936	0.9113
	ASN SSR	397 (±435)	427 (±398)	363 (±350)	281 (±279)
**Both** t_S	Power SSR	0.0226	0.6546	0.8291	0.9363
	ASN SSR	404 (±443)	435 (±410)	368 (±363)	284 (±290)
**Short**	Power SSR	0.0218	0.8031	0.9119	0.9677
	ASN SSR	499 (±503)	868 (±462)	960 (±401)	1014 (±350)
**Stopping Rule With The P-value Based On** $\hat{P}_S$					
	Probability to Stop for Futility	0.5358	0.5359	0.5359	0.5357
	Power NO SSR	0.0198	0.2943	0.4109	0.4564
	ASN NO SSR	120 (±75)	120 (±75)	120 (±75)	120 (±75)
**Long**	Power SSR	0.0182	0.3722	0.425	0.4543
	ASN SSR	429 (±459)	272 (±341)	211 (±264)	166 (±185)
**Both** t_B	Power SSR	0.0174	0.3571	0.4046	0.4408
	ASN SSR	452 (±469)	280 (±343)	213 (±259)	165 (±173)
**Both** t_S	Power SSR	0.0196	0.3812	0.4271	0.4537
	ASN SSR	469 (±483)	284 (±352)	214 (±266)	165 (±179)
**Short**	Power SSR	0.0201	0.4417	0.4598	0.4638
	ASN SSR	472 (±482)	472 (±482)	472 (±482)	472 (±482)

Table 13: Operating characteristics of a trial with sample size reassessment based on observed effect conditional power: overall power, probability to stop for futility, and average sample size per treatment arm over both stages (ASN) and its standard deviation (in brackets). Simulations with 3 different interim stopping approaches are shown: results with a p-value based on Z_L, second one with a p-value based on Z_B and the last one with p-value based on Z_S as a stopping rule.

3.2.3 $P_{S_E} = 0.285$

		Probability of Success			
	$\mathbf{P_{L_E}}$	0.2	0.285	0.323	0.365
	$\mathbf{P_{S_E}}$	0.285	0.285	0.285	0.285
	**Power Single Stage Trial**	0.0255	0.5112	0.8011	0.9591
**Long**	Probability to Stop for Futility	0.7704	0.4109	0.2611	0.1394
	Power SSR	0.017	0.3981	0.6357	0.8237
	ASN SSR	146 (±115)	177 (±113)	180 (±101)	175 (±86)
	Power NO SSR	0.0173	0.3816	0.6446	0.84
	ASN NO SSR	85 (±64)	139 (±74)	161 (±66)	180 (±52)
**Both** t_B	Probability to Stop for Futility	0.7843	0.3961	0.24	0.1194
	Power SSR	0.014	0.4066	0.6381	0.8252
	ASN SSR	152 (±126)	201 (±140)	204 (±128)	193 (±107)
	Power NO SSR	0.0174	0.3944	0.664	0.8596
**Both** t_S	Probability to Stop for Futility	0.8632	0.5172	0.3462	0.1921
	Power SSR	0.0138	0.3285	0.5672	0.7768
	ASN SSR	118 (±52)	148 (±68)	157 (±64)	161 (±54)
	Power NO SSR	0.014	0.3351	0.5847	0.7928
**Short**	Probability to Stop for Futility	0.385	0.3851	0.3853	0.3853
	Power SSR	0.0219	0.3891	0.5382	0.5999
	ASN SSR	186 (±115)	186 (±115)	186 (±115)	186 (±115)
	Power NO SSR	0.0224	0.369	0.5318	0.6019

Table 14: Operating characteristics of a trial with sample size reassessment based on observed effect conditional power with $c = 0.3$ as a futility stopping rule: overall power, probability to stop for futility, and average sample size per treatment arm over both stages (ASN) and its standard deviation (in brackets).

		Probability of Success			
	$\mathbf{P_{L_E}}$	0.2	0.285	0.323	0.365
	$\mathbf{P_{S_E}}$	0.285	0.285	0.285	0.285
	**Power Single Stage Trial**	0.0255	0.5112	0.8011	0.9591
**Stopping Rule With The P-value Based On $\hat{P}_L$**					
	Probability to Stop for Futility	0.5482	0.1885	0.0978	0.0394
	Power NO SSR	0.0224	0.4714	0.7548	0.9286
	ASN NO SSR	118 (±75)	172 (±59)	186 (±45)	195 (±30)
**Long**	Power SSR	0.0223	0.6187	0.7987	0.9235
	ASN SSR	374 (±424)	393 (±389)	337 (±343)	271 (±281)
**Both t_B**	Power SSR	0.0196	0.6124	0.7804	0.9052
	ASN SSR	400 (±438)	415 (±393)	349 (±341)	274 (±270)
**Both t_S**	Power SSR	0.0218	0.6417	0.8151	0.9296
	ASN SSR	408 (±447)	423 (±404)	354 (±353)	276 (±281)
**Short**	Power SSR	0.0225	0.6286	0.8375	0.9469
	ASN SSR	259 (±323)	446 (±424)	503 (±437)	542 (±443)
**Stopping Rule With The P-value Based On $\hat{P}_B$**					
	Probability to Stop for Futility	0.549	0.1747	0.0841	0.0312
	Power NO SSR	0.0229	0.4781	0.7645	0.936
	ASN NO SSR	118 (±75)	174 (±57)	188 (±42)	196 (±27)
**Long**	Power SSR	0.0215	0.633	0.8123	0.9319
	ASN SSR	377 (±428)	410 (±401)	353 (±358)	281 (±294)
**Both t_B**	Power SSR	0.02	0.6266	0.7938	0.9134
	ASN SSR	397 (±435)	427 (±398)	362 (±350)	281 (±279)
**Both t_S**	Power SSR	0.0223	0.6554	0.8292	0.9377
	ASN SSR	405 (±444)	435 (±410)	367 (±362)	284 (±290)
**Short**	Power SSR	0.0234	0.6388	0.8498	0.9552
	ASN SSR	256 (±319)	452 (±425)	511 (±438)	548 (±444)
**Stopping Rule With The P-value Based On $\hat{P}_S$**					
	Probability to Stop for Futility	0.0915	0.0923	0.0922	0.0923
	Power NO SSR	0.0248	0.489	0.7482	0.8781
	ASN NO SSR	187 (±44)	187 (±44)	187 (±44)	187 (±44)
**Long**	Power SSR	0.0238	0.7194	0.8151	0.8774
	ASN SSR	772 (±447)	515 (±446)	385 (±389)	276 (±300)
**Both t_B**	Power SSR	0.0242	0.7159	0.7943	0.8579
	ASN SSR	822 (±432)	538 (±444)	395 (±384)	277 (±290)
**Both t_S**	Power SSR	0.0248	0.7438	0.829	0.881
	ASN SSR	867 (±430)	550 (±456)	401 (±396)	279 (±299)
**Short**	Power SSR	0.0251	0.6749	0.8316	0.893
	ASN SSR	472 (±417)	472 (±416)	472 (±416)	472 (±416)

Table 15: Operating characteristics of a trial with sample size reassessment based on observed effect conditional power: overall power, probability to stop for futility, and average sample size per treatment arm over both stages (ASN) and its standard deviation (in brackets). Simulations with 3 different interim stopping approaches are shown: results with a p-value based on Z_L, second one with a p-value based on Z_B and the last one with p-value based on Z_S as a stopping rule.

3.2.4 $P_{S_E} = 0.323$

		Probability of Success			
	$\mathbf{P_{L_E}}$	0.2	0.285	0.323	0.365
	$\mathbf{P_{S_E}}$	0.323	0.323	0.323	0.323
	**Power Single Stage Trial**	0.0255	0.5113	0.8014	0.9593
**Long**	Probability to Stop for Futility	0.7705	0.411	0.2613	0.1389
	Power SSR	0.0171	0.3991	0.6359	0.8242
	ASN SSR	146 (±115)	177 (±113)	179 (±101)	175 (±86)
	Power NO SSR	0.0173	0.3818	0.6447	0.8406
	ASN NO SSR	85 (±64)	139 (±74)	161 (±66)	180 (±52)
**Both** t_B	Probability to Stop for Futility	0.7844	0.3969	0.2408	0.1194
	Power SSR	0.0138	0.4048	0.6368	0.825
	ASN SSR	152 (±125)	201 (±140)	204 (±128)	193 (±107)
	Power NO SSR	0.0174	0.3944	0.6637	0.8597
**Both** t_S	Probability to Stop for Futility	0.862	0.5174	0.3463	0.1929
	Power SSR	0.0137	0.329	0.568	0.776
	ASN SSR	118 (±53)	148 (±68)	157 (±64)	160 (±54)
	Power NO SSR	0.0141	0.3352	0.585	0.792
**Short**	Probability to Stop for Futility	0.1925	0.1924	0.1924	0.1923
	Power SSR	0.0249	0.4354	0.6574	0.772
	ASN SSR	186 (±97)	186 (±97)	186 (±97)	186 (±97)
	Power NO SSR	0.0244	0.4519	0.6775	0.7849

Table 16: Operating characteristics of a trial with sample size reassessment based on observed effect conditional power with $c = 0.3$ as a futility stopping rule: overall power, probability to stop for futility, and average sample size per treatment arm over both stages (ASN) and its standard deviation (in brackets).

		Probability of Success			
	$\mathbf{P_{L_E}}$	0.2	0.285	0.323	0.365
	$\mathbf{P_{S_E}}$	0.323	0.323	0.323	0.323
	**Power Single Stage Trial**	0.0255	0.5113	0.8014	0.9593
**Stopping Rule With The P-value Based On $\hat{P}_L$**					
	Probability to Stop for Futility	0.5481	0.1885	0.098	0.0396
	Power NO SSR	0.0226	0.4713	0.7548	0.9285
	ASN NO SSR	118 (±75)	172 (±59)	186 (±45)	195 (±30)
**Long**	Power SSR	0.0219	0.6181	0.799	0.9234
	ASN SSR	375 (±425)	393 (±389)	337 (±343)	271 (±281)
**Both t_B**	Power SSR	0.0205	0.6122	0.7797	0.905
	ASN SSR	401 (±439)	415 (±392)	350 (±341)	274 (±270)
**Both t_S**	Power SSR	0.022	0.6429	0.8156	0.9291
	ASN SSR	408 (±447)	422 (±404)	355 (±354)	276 (±281)
**Short**	Power SSR	0.0228	0.5231	0.7765	0.9284
	ASN SSR	183 (±212)	290 (±313)	325 (±336)	350 (±351)
**Stopping Rule With The P-value Based On $\hat{P}_B$**					
	Probability to Stop for Futility	0.548	0.1748	0.0843	0.0312
	Power NO SSR	0.0229	0.4778	0.765	0.9362
	ASN NO SSR	118 (±75)	174 (±57)	188 (±42)	196 (±27)
**Long**	Power SSR	0.0218	0.6331	0.8126	0.9318
	ASN SSR	378 (±429)	410 (±401)	353 (±358)	281 (±294)
**Both t_B**	Power SSR	0.0209	0.6259	0.7935	0.9132
	ASN SSR	398 (±436)	427 (±398)	362 (±350)	282 (±280)
**Both t_S**	Power SSR	0.0227	0.6552	0.8295	0.9375
	ASN SSR	406 (±444)	435 (±410)	368 (±363)	284 (±291)
**Short**	Power SSR	0.023	0.5306	0.7873	0.9362
	ASN SSR	180 (±207)	293 (±315)	329 (±338)	354 (±352)
**Stopping Rule With The P-value Based On $\hat{P}_S$**					
	Probability to Stop for Futility	0.0278	0.0279	0.028	0.028
	Power NO SSR	0.0252	0.5066	0.7882	0.9358
	ASN NO SSR	196 (±25)	196 (±25)	196 (±25)	196 (±25)
**Long**	Power SSR	0.0244	0.7702	0.8714	0.9371
	ASN SSR	819 (±423)	559 (±450)	420 (±403)	300 (±318)
**Both t_B**	Power SSR	0.0252	0.7699	0.8514	0.9179
	ASN SSR	873 (±398)	584 (±447)	432 (±399)	302 (±308)
**Both t_S**	Power SSR	0.0243	0.7964	0.8871	0.9421
	ASN SSR	924 (±389)	598 (±458)	440 (±410)	304 (±318)
**Short**	Power SSR	0.0258	0.5928	0.8217	0.9364
	ASN SSR	341 (±337)	341 (±337)	341 (±336)	341 (±336)

Table 17: Operating characteristics of a trial with sample size reassessment based on observed effect conditional power: overall power, probability to stop for futility, and average sample size per treatment arm over both stages (ASN) and its standard deviation (in brackets). Simulations with 3 different interim stopping approaches are shown: results with a p-value based on Z_L, second one with a p-value based on Z_B and the last one with p-value based on Z_S as a stopping rule.

3.2.5 $P_{S_E} = 0.365$

		Probability of Success			
	$\mathbf{P_{L_E}}$	0.2	0.285	0.323	0.365
	$\mathbf{P_{S_E}}$	0.365	0.365	0.365	0.365
	**Power Single Stage Trial**	0.0254	0.5111	0.8014	0.9594
**Long**	Probability to Stop for Futility	0.7705	0.4108	0.2614	0.1388
	Power SSR	0.017	0.3985	0.6356	0.8244
	ASN SSR	146 (±115)	177 (±113)	180 (±101)	175 (±86)
	Power NO SSR	0.0171	0.3816	0.6447	0.8407
	ASN NO SSR	85 (±64)	139 (±74)	161 (±66)	180 (±52)
**Both** t_B	Probability to Stop for Futility	0.7849	0.3968	0.2411	0.1192
	Power SSR	0.0142	0.4049	0.6355	0.8248
	ASN SSR	152 (±125)	201 (±140)	203 (±127)	193 (±107)
	Power NO SSR	0.0173	0.3939	0.6633	0.86
**Both** t_S	Probability to Stop for Futility	0.8629	0.5165	0.3466	0.1927
	Power SSR	0.0138	0.3291	0.567	0.776
	ASN SSR	118 (±52)	148 (±68)	157 (±64)	160 (±54)
	Power NO SSR	0.014	0.3359	0.585	0.792
**Short**	Probability to Stop for Futility	0.0691	0.0691	0.0692	0.0693
	Power SSR	0.0253	0.4294	0.697	0.8653
	ASN SSR	172 (±70)	172 (±70)	172 (±69)	172 (±69)
	Power NO SSR	0.0251	0.4946	0.762	0.8988

Table 18: Operating characteristics of a trial with sample size reassessment based on observed effect conditional power with $c = 0.3$ as a futility stopping rule: overall power, probability to stop for futility, and average sample size per treatment arm over both stages (ASN) and its standard deviation (in brackets).

		Probability of Success			
	$\mathbf{P_{L_E}}$	0.2	0.285	0.323	0.365
	$\mathbf{P_{S_E}}$	0.365	0.365	0.365	0.365
	**Power Single Stage Trial**	0.0254	0.5111	0.8014	0.9594
**Stopping Rule With The P-value Based On $\hat{P}_L$**					
	Probability to Stop for Futility	0.5485	0.1885	0.0979	0.0395
	Power NO SSR	0.0224	0.4714	0.7548	0.9288
	ASN NO SSR	118 (±75)	172 (±59)	186 (±45)	195 (±30)
**Long**	Power SSR	0.0224	0.6188	0.7988	0.9237
	ASN SSR	374 (±424)	393 (±389)	337 (±343)	271 (±281)
**Both t_B**	Power SSR	0.0202	0.6124	0.7787	0.9048
	ASN SSR	400 (±438)	414 (±392)	349 (±341)	274 (±270)
**Both t_S**	Power SSR	0.0223	0.6423	0.8152	0.9289
	ASN SSR	407 (±446)	422 (±404)	355 (±353)	276 (±281)
**Short**	Power SSR	0.0227	0.4339	0.7097	0.9029
	ASN SSR	141 (±113)	193 (±185)	210 (±204)	223 (±218)
**Stopping Rule With The P-value Based On $\hat{P}_B$**					
	Probability to Stop for Futility	0.5476	0.1747	0.0843	0.0311
	Power NO SSR	0.0228	0.4779	0.7652	0.9364
	ASN NO SSR	118 (±75)	174 (±57)	188 (±42)	196 (±27)
**Long**	Power SSR	0.0219	0.6335	0.8127	0.9322
	ASN SSR	378 (±429)	410 (±401)	353 (±358)	281 (±294)
**Both t_B**	Power SSR	0.0207	0.6265	0.7921	0.9132
	ASN SSR	399 (±436)	427 (±397)	362 (±350)	281 (±279)
**Both t_S**	Power SSR	0.0226	0.6554	0.8283	0.9374
	ASN SSR	406 (±445)	435 (±409)	367 (±362)	284 (±290)
**Short**	Power SSR	0.0231	0.4396	0.7197	0.9098
	ASN SSR	140 (±109)	194 (±186)	212 (±206)	225 (±220)
**Stopping Rule With The P-value Based On $\hat{P}_S$**					
	Probability to Stop for Futility	0.0053	0.0055	0.0054	0.0054
	Power NO SSR	0.0253	0.5115	0.8003	0.9554
	ASN NO SSR	200 (±11)	200 (±12)	200 (±12)	200 (±11)
**Long**	Power SSR	0.0255	0.7883	0.8904	0.9582
	ASN SSR	835 (±413)	575 (±451)	434 (±408)	309 (±326)
**Both t_B**	Power SSR	0.0253	0.7882	0.8714	0.9388
	ASN SSR	891 (±384)	602 (±447)	447 (±403)	312 (±315)
**Both t_S**	Power SSR	0.0254	0.8151	0.9071	0.9633
	ASN SSR	944 (±371)	617 (±458)	455 (±415)	315 (±326)
**Short**	Power SSR	0.0254	0.4878	0.7605	0.9293
	ASN SSR	229 (±221)	229 (±221)	229 (±221)	229 (±221)

Table 19: Operating characteristics of a trial with sample size reassessment based on observed effect conditional power: overall power, probability to stop for futility, and average sample size per treatment arm over both stages (ASN) and its standard deviation (in brackets). Simulations with 3 different interim stopping approaches are shown: results with a p-value based on Z_L, second one with a p-value based on Z_B and the last one with p-value based on Z_S as a stopping rule.

3.2.6 Weight fixed and varied

First Stage Weight		$\sqrt{0}$	$\sqrt{0.1}$	$\sqrt{0.2}$	$\sqrt{0.3}$	$\sqrt{0.4}$	$\sqrt{0.5}$	$\sqrt{0.6}$	$\sqrt{0.7}$	$\sqrt{0.8}$	$\sqrt{0.9}$	$\sqrt{1}$
**Power Single Stage Trial**		0.8014	0.8014	0.8014	0.8014	0.8014	0.8014	0.8014	0.8014	0.8014	0.8014	0.8014
**Stopping Rule with the P-value based on $\hat{P}_L$**												
	FS	0.098	0.098	0.098	0.098	0.098	0.098	0.098	0.098	0.098	0.098	0.098
	Power NO SSR	0.614	0.7268	0.7492	0.7579	0.7572	0.7498	0.7339	0.7032	0.6515	0.5602	0.2878
	ASN NO SSR	186 (45)	186 (45)	186 (45)	186 (45)	186 (45)	186 (45)	186 (45)	186 (45)	186 (45)	186 (45)	186 (45)
**Long**	Power SSR	0.6688	0.7592	0.7885	0.8068	0.8193	0.8285	0.8361	0.8414	0.8448	0.8395	0.2725
	ASN SSR	365 (344)	340 (340)	337 (342)	337 (345)	341 (350)	348 (359)	359 (372)	375 (387)	397 (400)	447 (434)	1093 (328)
**Both**	Power SSR	0.637	0.7219	0.7577	0.7827	0.8015	0.8152	0.8247	0.8318	0.8334	0.8235	0.482
	ASN SSR	394 (338)	358 (336)	351 (338)	350 (342)	351 (347)	355 (354)	362 (362)	375 (375)	396 (393)	441 (424)	1093 (328)
**Short**	Power SSR	0.6691	0.7117	0.7351	0.7536	0.7657	0.7765	0.7827	0.7846	0.7807	0.7648	0.483
	ASN SSR	400 (327)	347 (325)	334 (326)	328 (328)	325 (331)	325 (336)	327 (341)	333 (351)	347 (367)	381 (391)	1093 (328)
**Stopping Rule with the P-value based on $\hat{P}_B$**												
	FS	0.0843	0.0843	0.0843	0.0843	0.0843	0.0843	0.0843	0.0843	0.0843	0.0843	0.0843
	Power NO SSR	0.628	0.7392	0.7604	0.7672	0.7649	0.7552	0.7369	0.7039	0.6509	0.5602	0.2877
	ASN NO SSR	188 (42)	188 (42)	188 (42)	188 (42)	188 (42)	188 (42)	188 (42)	188 (42)	188 (42)	188 (42)	188 (42)
**Long**	Power SSR	0.6822	0.7736	0.8027	0.8208	0.8333	0.8421	0.8502	0.8552	0.8581	0.8409	0.2729
	ASN SSR	381 (358)	356 (355)	353 (357)	354 (360)	357 (365)	364 (373)	375 (385)	391 (398)	412 (410)	463 (441)	1108 (306)
**Both**	Power SSR	0.6502	0.736	0.7714	0.7959	0.8149	0.8291	0.8389	0.8446	0.8473	0.8377	0.4844
	ASN SSR	407 (346)	371 (345)	364 (347)	362 (351)	364 (356)	368 (363)	376 (371)	388 (383)	410 (401)	455 (430)	1108 (306)
**Short**	Power SSR	0.6808	0.7231	0.7458	0.7644	0.7772	0.7871	0.7934	0.7956	0.7908	0.7746	0.4852
	ASN SSR	405 (328)	352 (327)	339 (328)	332 (330)	330 (334)	329 (338)	332 (344)	338 (353)	352 (370)	386 (393)	1108 (306)
**Stopping Rule with the P-value based on $\hat{P}_S$**												
	FS	0.028	0.028	0.028	0.028	0.028	0.028	0.028	0.028	0.028	0.028	0.028
	Power NO SSR	0.667	0.7708	0.7863	0.7872	0.7785	0.7623	0.7382	0.7	0.6445	0.5546	0.2855
	ASN NO SSR	196 (25)	196 (25)	196 (25)	196 (25)	196 (25)	196 (25)	196 (25)	196 (25)	196 (25)	196 (25)	196 (25)
**Long**	Power SSR	0.741	0.8319	0.8612	0.8792	0.8911	0.8999	0.9078	0.9121	0.9085	0.858	0.2703
	ASN SSR	442 (393)	421 (397)	419 (401)	421 (405)	426 (410)	434 (417)	446 (427)	462 (438)	484 (446)	533 (466)	1170 (182)
**Both**	Power SSR	0.7092	0.794	0.8295	0.8548	0.8735	0.8877	0.8975	0.9034	0.9045	0.8866	0.4836
	ASN SSR	472 (383)	439 (390)	433 (395)	433 (399)	435 (404)	440 (410)	448 (417)	461 (426)	482 (438)	526 (457)	1170 (182)
**Short**	Power SSR	0.7269	0.7649	0.7869	0.8031	0.8133	0.822	0.8252	0.8251	0.8164	0.7924	0.4849
	ASN SSR	424 (324)	365 (324)	351 (325)	344 (327)	341 (331)	341 (336)	343 (343)	351 (354)	366 (372)	405 (397)	1170 (182)

Table 20: Operating characteristics (probability to stop for futility (FS), overall power and average sample size (ASN) and its standard deviation) of a trial with sample size reassessment based on observed effect conditional power with p-value futility stopping rule based on p-vales of Z_L, Z_S and Z_B for a different choice of weights for the combination test and sample size reassessment.

4 References

Cramér, H. (1946). Mathematical Methods of Statistics, Princeton University Press, Princeton, 282.

Marschner, I. C. and Becker, S. L. (2001). Interim monitoring of clinical trials based on long-term binary endpoints. *Statistics in Medicine* **20**, 177–192.

Proschan, M. A. and Lan, K. K. G. and Wittes, J. T. (2006). Statistical Monitoring of Clinical Trials: A Unified Approach, Springer-Verlag, New York, 43–66.

Stallard, N. (2010). A confirmatory seamless phase II/III clinical trial design incorporating short-term endpoint information. *Statistics in Medicine* **29**, 959–971
