# Supplementary material for: Interim analysis incorporating short‐ and long‐term binary endpoints
Source: Biom J. 2019 Jan 29;61(3):665–87. doi: 10.1002/bimj.201700281 (PMC6590444; doi:10.1002/bimj.201700281)
Supplement: Supplementary file 1 — Supporting Information [file BIMJ-61-665-s001.zip › Reproducibility_Julia_Niewczas/binfutssr-manual.pdf]

Package ‘binfutssr’

December 20, 2018

Type Package

Title Incorporation of Short-Term Information into Interim Analyses with Futility Stopping or Sample Size Reassessment for Binary Endoints

Version 0.1.0

Author Julia Niewczas

Maintainer Julia Niewczas <julia.niewczas@gmail.com>

Description This package simulates clinical trial scenarios for designs incoroporating short-term information into the analysis. Here we consider only binary endpoints with one interim analysis stopping for futility only. Two approaches for stopping the trial at interim are considered: conditional power using fixed effect and conditional power using the observed effect. Further sample size reassessments are considered using combination test as the final analysis.

License GPL-2

Depends foreach, mvtnorm, psych, parallel, doParallel, ggplot2, stringr, grid, gridExtra, xtable

RoxygenNote 5.0.1

Suggests knitr, rmarkdown

VignetteBuilder knitr

NeedsCompilation no

R topics documented:

cp 2
create_corr_plots 3
create_corr_plots_prob_cont_rej_p_se 4
create_corr_plots_prob_corr_dec_p_se 7
create_corr_plots_prob_stop_not_p_se 9
create_corr_plots_p_se 12
create_corr_plots_p_se_low 14
create_fs_plots 17
create_fs_plots_nested 18
create_power_plots 20
create_power_plots_nested 22
create_prob_cont_rej_plots 24

create_prob_cont_rej_plots_nested 26
create_prob_corr_dec_plots 28
create_prob_corr_dec_plots_nested 29
create_prob_stop_not_rej_plots 31
create_prob_stop_not_rej_plots_nested 33
create_ssr_fix_cp_table 35
create_ssr_fix_pval_table 36
create_ssr_fix_pval_weight_table 38
create_ssr_obs_cp_table 40
create_ssr_obs_pval_table 41
create_ssr_obs_pval_weight_table 43
fixed_to_obs_plot 45
ssr_fixed_cp 46
ssr_fixed_z 47
ssr_fixed_z_weight 48
ssr_obs_cp 49
ssr_obs_z 50
ssr_obs_z_weight 52

Index **54**

`cp` *Conditional power Stopping For Futility Approaches*

Description

Calculate operating characteristics of a trial simulated under the alternative hypothesis with binary endpoints with one interim analysis at time t allowing for futility stopping only using two conditional power approaches: conditional power basen on the fixed effect and obs effect_ Three estimators are compared: using long-term data only, short-term data only, and combination of both_

Usage

```
cp(nsim, alpha, beta, p_le, p_lc, p_se, p_sc, n, fr_lo, fr_sh, phi_e, phi_c, c)
```

Arguments

`nsim`	Number of simulated trials to be run
`alpha`	Type 1 error level
`beta`	Type 2 error level
`p_le`	Probability of success in the treatment group for the long-term endpoint L
`p_lc`	Probability of success in the control group for the long-term endpoint L
`p_se`	Probability of success in the trearment group for the short-term endpoint S
`p_sc`	Probability of success in the control group for the short-term endpoint S
`n`	Sample size per treatment group
`fr_lo`	Amount of information available at interim for the long-term endpoint, value between 0 and 1
`fr_sh`	Amount of information available at interim for the short-term endpoint, value between 0 and 1

`phi_e` Correlation between S and L in the treatment group

`phi_c` Correlation between S and L in the control group

`c` The cut-off point to be considered for the conditional power. Can be a single value or a vector of values between 0 and 1

Value

The function returns a data frame with operating characteristics of a simulated trial for each cut-off point Including power for a one-stage trial, overall power for all estimators under both fixed and obs effects, probability to stop for futility, the amount of times then P_B was undefined, mean conditional power, power if the trial was continued, probability not to reject given the trial was continued, probability to having rejected the null if it the trial had been continued (when it was stopped), probability to having failed to reject the null for those that were stopped for futility had they been continued, probability of making the correct decision

Examples

```
cp(nsim = 1000,
alpha = 0.025,
beta = 0.2,
p_le = 0.3227348,
p_lc = 0.2,
p_se = 0.3227348,
p_sc = 0.2,
n = 200,
fr_lo = 0.25,
fr_sh = 0.5,
phi_e = 0.5,
phi_c = 0.5,
c = seq(0, 1, 0.1))
```

`create_corr_plots` *Plot for Overall Power Different t and Correlation*

Description

Obtain a plot with overall power for a range of correlations between S and L. The power is obtained for cut-off points for which probability to stop for futility under alternative hypothesis is equal to 10 Two cases are considered t_s = 0.5 and t_s = 0.75.

Usage

```
create_corr_plots(output_cp_alt_fr_sh_05, output_cp_alt_fr_sh_075, phi_e, phi_c,
  fr_lo, filetype)
```

Arguments

`output_cp_alt_fr_sh_05`
Output from simulation of a clinical trial under alternative hypothesis with fr_sh = 0.5

`output_cp_alt_fr_sh_075`
Output from simulation of a clinical trial under alternative hypothesis with fr_sh = 0.75

phi_e	Correlation between S and L in the treatment group
phi_c	Correlation between S and L in the control group
fr_lo	Amount of information available at interim for the long-term endpoint, value between 0 and 1
filetype	Filetype which the plot should be saved to. Options are: c("pdf", "eps", "none"). If none is chosen the plot is printed

Value

The function creates a plot with results of simulations based on the output from function cp(). It consists of 2 plots showing overall power for a case when the cut-off point is chosen based on probability to stop for futility under alternative hypothesis of 10"%"

Examples

```
cl <- makeCluster(1)
registerDoParallel(cl)

phi_e = c(0, 0.2, 0.5, 0.7, 0.9)
phi_c = c(0, 0.2, 0.5, 0.7, 0.9)

output_cp_alt_fr_sh_05 <- foreach(i = 1:length(phi_e), .packages="binfutssr") %dopar%
(cp(nsim = 1000,
alpha = 0.025,
beta = 0.2,
p_le = 0.3227348,
p_lc = 0.2,
p_se = 0.3227348,
p_sc = 0.2,
n = 200,
fr_lo = 0.25,
fr_sh = 0.5,
phi_e = phi_e[i],
phi_c = phi_c[i],
c = seq(0, 1, 0.01)))

output_cp_alt_fr_sh_075 <- foreach(i = 1:length(phi_e), .packages="binfutssr") %dopar%
 (cp(nsim = 1000,
 alpha = 0.025,
 beta = 0.2,
 p_le = 0.3227348,
 p_lc = 0.2,
 p_se = 0.3227348,
 p_sc = 0.2,
 n = 200,
 fr_lo = 0.25,
 fr_sh = 0.75,
 phi_e = phi_e[i],
 phi_c = phi_c[i],
 c = seq(0, 1, 0.01)))

create_corr_plots(output_cp_alt_fr_sh_05 = output_cp_alt_fr_sh_05,
output_cp_alt_fr_sh_075 = output_cp_alt_fr_sh_075,
phi_e = phi_e,
phi_c = phi_c,
```

```
fr_lo = 0.25,
filetype = "pdf")
```

---

create_corr_plots_prob_cont_rej_p_se
Correlation Plots Prob Cont And Reject

Description

Obtain a plot with probability to reject the null hypothesis given the trial was continued for a range of correlations between S and L. The probability is obtained for cut-off points for which probability to stop for futility under alternative hypothesis is equal to 10 true p_se is different than it was expected. This is done for three estimators. Two cases are considered t_s = 0.5 and t_s = 0.75.

Usage

```
create_corr_plots_prob_cont_rej_p_se(output_cp_alt_fr_sh_05_p_se,
  output_cp_alt_fr_sh_075_p_se, plots_corr, phi_e, phi_c, fr_lo, filetype)
```

Arguments

`output_cp_alt_fr_sh_05_p_se`	Output from simulation of a clinical trial under alternative hypothesis with fr_sh = 0.5 for different p_se
`output_cp_alt_fr_sh_075_p_se`	Output from simulation of a clinical trial under alternative hypothesis with fr_sh = 0.75 for different p_se
`plots_corr`	Output from 'create_corr_plots' function for p_se equal to p_le
`phi_e`	Correlation between S and L in the treatment group
`phi_c`	Correlation between S and L in the control group
`fr_lo`	Amount of information available at interim for the long-term endpoint, value between 0 and 1
`filetype`	- Filetype which the plot should be saved to. Options are: c("pdf", "eps", "none"). If none is chosen the plot is printed

Value

The function creates a plot with results of simulations based on the output from function cp(). It consists of 8 plots showing probability to reject the null hypothesis given the trial was continued for a case when the cut-off point is chosen based on probability to stop for futility under alternative hypothesis of 10"%" for different values of p_se assuming it could have been mis-specified.

Examples

```
cl <- makeCluster(1)
registerDoParallel(cl)

phi_e = c(0,0.2,0.5,0.7,0.9)
phi_c = c(0,0.2,0.5,0.7,0.9)
```

```
output_cp_alt_fr_sh_05 <- foreach(i = 1:length(phi_e), .packages="binfutssr") %dopar%
(cp(nsim = 1000,
alpha = 0.025,
beta = 0.2,
p_le = 0.3227348,
p_lc = 0.2,
p_se = 0.3227348,
p_sc = 0.2,
n = 200,
fr_lo = 0.25,
fr_sh = 0.5,
phi_e = phi_e[i],
phi_c = phi_c[i],
c = seq(0, 1, 0.01)))

output_cp_alt_fr_sh_075 <- foreach(i = 1:length(phi_e), .packages="binfutssr") %dopar%
 (cp(nsim = 1000,
 alpha = 0.025,
 beta = 0.2,
 p_le = 0.3227348,
 p_lc = 0.2,
 p_se = 0.3227348,
 p_sc = 0.2,
 n = 200,
 fr_lo = 0.25,
 fr_sh = 0.75,
 phi_e = phi_e[i],
 phi_c = phi_c[i],
 c = seq(0, 1, 0.01)))

plots_corr <- create_corr_plots(output_cp_alt_fr_sh_05 = output_cp_alt_fr_sh_05,
output_cp_alt_fr_sh_075 = output_cp_alt_fr_sh_075,
phi_e = phi_e,
phi_c = phi_c,
fr_lo = 0.25,
filetype = "none")

phi_e_v2 <- c(0, 0.2, 0.5, 0.7,
             0, 0.2, 0.5, 0.7, 0.9,
             0, 0.2, 0.5, 0.7, 0.9,
             0, 0.2, 0.5, 0.7, 0.9)

phi_c_v2 <- c(0, 0.2, 0.5, 0.7,
             0, 0.2, 0.5, 0.7, 0.9,
             0, 0.2, 0.5, 0.7, 0.9,
             0, 0.2, 0.5, 0.7, 0.9)

p_se <- c(rep(0.2, 4), rep(0.285, 5),
rep(0.3227348 ,5), rep(0.365, 5))

output_cp_alt_fr_sh_05_p_se <- foreach(i = 1:length(phi_e_v2),
.packages= c("binfutssr","psych","mvtnorm")) %dopar%
(cp(nsim = 1000,
alpha = 0.025,
beta = 0.2,
p_le = 0.3227348,
p_lc = 0.2,
```

```
p_se = p_se[i],
p_sc = 0.2,
n = 200,
fr_lo = 0.25,
fr_sh = 0.5,
phi_e = phi_e_v2[i],
phi_c = phi_c_v2[i],
c = seq(0, 1, 0.01)))

output_cp_alt_fr_sh_075_p_se <- foreach(i = 1:length(phi_e_v2),
.packages= c("binfutssr","psych","mvtnorm")) %dopar%
(cp(nsim = 1000,
alpha = 0.025,
beta = 0.2,
p_le = 0.3227348,
p_lc = 0.2,
p_se = p_se[i],
p_sc = 0.2,
n = 200,
fr_lo = 0.25,
fr_sh = 0.75,
phi_e = phi_e_v2[i],
phi_c = phi_c_v2[i],
c = seq(0, 1, 0.01)))

create_corr_plots_prob_cont_rej_p_se(output_cp_alt_fr_sh_05_p_se = output_cp_alt_fr_sh_05_p_se,
output_cp_alt_fr_sh_075_p_se = output_cp_alt_fr_sh_075_p_se,
plots_corr = plots_corr,
phi_e = phi_e_v2,
phi_c = phi_c_v2,
fr_lo = 0.25,
filetype = "pdf")
```

`create_corr_plots_prob_corr_dec_p_se`
Correlation Plots Correct Decision

Description

Obtain a plot with probability to make the correct decision the for a range of correlations between S and L. The probability is obtained for cut-off points for which probability to stop for futility under alternative hypothesis is equal to 10 what happens if true p_se is different than it was expected.v This is done for three estimators. Two cases are considered t_s = 0.5 and t_s = 0.75.

Usage

```
create_corr_plots_prob_corr_dec_p_se(output_cp_alt_fr_sh_05_p_se,
  output_cp_alt_fr_sh_075_p_se, plots_corr, phi_e, phi_c, fr_lo, filetype)
```

Arguments

`output_cp_alt_fr_sh_05_p_se`
: Output from simulation of a clinical trial under alternative hypothesis with fr_sh = 0.5 for different p_se

`output_cp_alt_fr_sh_075_p_se`
: Output from simulation of a clinical trial under alternative hypothesis with fr_sh = 0.75 for different p_se

`plots_corr`
: Output from 'create_corr_plots' function for p_se equal to p_le

`phi_e`
: Correlation between S and L in the treatment group

`phi_c`
: Correlation between S and L in the control group

`fr_lo`
: Amount of information available at interim for the long-term endpoint, value between 0 and 1

`filetype`
: - Filetype which the plot should be saved to. Options are: c("pdf", "eps", "none"). If none is chosen the plot is printed

Value

The function creates a plot with results of simulations based on the output from function cp(). It consists of 8 plots showing probability to make the correct decision for a case when the cut-off point is chosen based on probabilityto stop for futility under alternative hypothesis of 10"%" for different values of p_se assuming it could have been mis-specified.

Examples

```
cl <- makeCluster(1)
registerDoParallel(cl)

phi_e = c(0,0.2,0.5,0.7,0.9)
phi_c = c(0,0.2,0.5,0.7,0.9)

output_cp_alt_fr_sh_05 <- foreach(i = 1:length(phi_e), .packages="binfutssr") %dopar%
(cp(nsim = 1000,
alpha = 0.025,
beta = 0.2,
p_le = 0.3227348,
p_lc = 0.2,
p_se = 0.3227348,
p_sc = 0.2,
n = 200,
fr_lo = 0.25,
fr_sh = 0.5,
phi_e = phi_e[i],
phi_c = phi_c[i],
c = seq(0, 1, 0.01)))

output_cp_alt_fr_sh_075 <- foreach(i = 1:length(phi_e), .packages="binfutssr") %dopar%
 (cp(nsim = 1000,
 alpha = 0.025,
 beta = 0.2,
 p_le = 0.3227348,
 p_lc = 0.2,
 p_se = 0.3227348,
 p_sc = 0.2,
 n = 200,
 fr_lo = 0.25,
 fr_sh = 0.75,
 phi_e = phi_e[i],
 phi_c = phi_c[i],
 c = seq(0, 1, 0.01)))
```

```
plots_corr <- create_corr_plots(output_cp_alt_fr_sh_05 = output_cp_alt_fr_sh_05,
output_cp_alt_fr_sh_075 = output_cp_alt_fr_sh_075,
phi_e = phi_e,
phi_c = phi_c,
fr_lo = 0.25,
filetype = "none")

phi_e_v2 <- c(0, 0.2, 0.5, 0.7,
             0, 0.2, 0.5, 0.7, 0.9,
             0, 0.2, 0.5, 0.7, 0.9,
             0, 0.2, 0.5, 0.7, 0.9)

phi_c_v2 <- c(0, 0.2, 0.5, 0.7,
             0, 0.2, 0.5, 0.7, 0.9,
             0, 0.2, 0.5, 0.7, 0.9,
             0, 0.2, 0.5, 0.7, 0.9)

p_se <- c(rep(0.2, 4), rep(0.285, 5),
rep(0.3227348 ,5), rep(0.365, 5))

output_cp_alt_fr_sh_05_p_se <- foreach(i = 1:length(phi_e_v2),
.packages= c("binfutssr","psych","mvtnorm")) %dopar%
(cp(nsim = 1000,
alpha = 0.025,
beta = 0.2,
p_le = 0.3227348,
p_lc = 0.2,
p_se = p_se[i],
p_sc = 0.2,
n = 200,
fr_lo = 0.25,
fr_sh = 0.5,
phi_e = phi_e_v2[i],
phi_c = phi_c_v2[i],
c = seq(0, 1, 0.01)))

output_cp_alt_fr_sh_075_p_se <- foreach(i = 1:length(phi_e_v2),
.packages= c("binfutssr","psych","mvtnorm")) %dopar%
(cp(nsim = 1000,
alpha = 0.025,
beta = 0.2,
p_le = 0.3227348,
p_lc = 0.2,
p_se = p_se[i],
p_sc = 0.2,
n = 200,
fr_lo = 0.25,
fr_sh = 0.75,
phi_e = phi_e_v2[i],
phi_c = phi_c_v2[i],
c = seq(0, 1, 0.01)))

create_corr_plots_prob_corr_dec_p_se(output_cp_alt_fr_sh_05_p_se = output_cp_alt_fr_sh_05_p_se,
output_cp_alt_fr_sh_075_p_se = output_cp_alt_fr_sh_075_p_se,
plots_corr = plots_corr,
phi_e = phi_e_v2,
```

```
phi_c = phi_c_v2,
fr_lo = 0.25,
filetype = "pdf")
```

---

create_corr_plots_prob_stop_not_p_se
Correlation Plots Prob Not Reject If Stopped for Futility

Description

Obtain a plot with Obtain a plot with probability of having rejected the null hypothesis given the trial was stopped had it been continued for a range of correlations between S and L. The probability is obtained for cut-off points for which probability to stop for futility under alternative hypothesis is equal to 10 true p_se is different than it was expected. This is done for three estimators. Two cases are considered t_s = 0.5 and t_s = 0.75.

Usage

```
create_corr_plots_prob_stop_not_p_se(output_cp_alt_fr_sh_05_p_se,
  output_cp_alt_fr_sh_075_p_se, plots_corr, phi_e, phi_c, fr_lo, filetype)
```

Arguments

`output_cp_alt_fr_sh_05_p_se`
: Output from simulation of a clinical trial under alternative hypothesis with fr_sh = 0.5 for different p_se

`output_cp_alt_fr_sh_075_p_se`
: Output from simulation of a clinical trial under alternative hypothesis with fr_sh = 0.75 for different p_se

`plots_corr`
: Output from 'create_corr_plots' function for p_se equal to p_le

`phi_e`
: Correlation between S and L in the treatment group

`phi_c`
: Correlation between S and L in the control group

`fr_lo`
: Amount of information available at interim for the long-term endpoint, value between 0 and 1

`filetype`
: - Filetype which the plot should be saved to. Options are: c("pdf", "eps", "none"). If none is chosen the plot is printed

Value

The function creates a plot with results of simulations based on the output from function cp(). It consists of 8 plots showing probability of having rejected the null hypothesis given the trial was stopped had it been continued for a case when the cut-off point is chosen based on probability to stop for futility under alternative hypothesis of 10"%" for different values of p_se assuming it could have been mis-specified.

Examples

```
cl <- makeCluster(1)
registerDoParallel(cl)

phi_e = c(0,0.2,0.5,0.7,0.9)
phi_c = c(0,0.2,0.5,0.7,0.9)

output_cp_alt_fr_sh_05 <- foreach(i = 1:length(phi_e), .packages="binfutssr") %dopar%
(cp(nsim = 1000,
alpha = 0.025,
beta = 0.2,
p_le = 0.3227348,
p_lc = 0.2,
p_se = 0.3227348,
p_sc = 0.2,
n = 200,
fr_lo = 0.25,
fr_sh = 0.5,
phi_e = phi_e[i],
phi_c = phi_c[i],
c = seq(0, 1, 0.01)))

output_cp_alt_fr_sh_075 <- foreach(i = 1:length(phi_e), .packages="binfutssr") %dopar%
 (cp(nsim = 1000,
 alpha = 0.025,
 beta = 0.2,
 p_le = 0.3227348,
 p_lc = 0.2,
 p_se = 0.3227348,
 p_sc = 0.2,
 n = 200,
 fr_lo = 0.25,
 fr_sh = 0.75,
 phi_e = phi_e[i],
 phi_c = phi_c[i],
 c = seq(0, 1, 0.01)))

plots_corr <- create_corr_plots(output_cp_alt_fr_sh_05 = output_cp_alt_fr_sh_05,
output_cp_alt_fr_sh_075 = output_cp_alt_fr_sh_075,
phi_e = phi_e,
phi_c = phi_c,
fr_lo = 0.25,
filetype = "none")

phi_e_v2 <- c(0, 0.2, 0.5, 0.7,
             0, 0.2, 0.5, 0.7, 0.9,
             0, 0.2, 0.5, 0.7, 0.9,
             0, 0.2, 0.5, 0.7, 0.9)

phi_c_v2 <- c(0, 0.2, 0.5, 0.7,
             0, 0.2, 0.5, 0.7, 0.9,
             0, 0.2, 0.5, 0.7, 0.9,
             0, 0.2, 0.5, 0.7, 0.9)

p_se <- c(rep(0.2, 4), rep(0.285, 5),
rep(0.3227348 ,5), rep(0.365, 5))
```

```
output_cp_alt_fr_sh_05_p_se <- foreach(i = 1:length(phi_e_v2),
.packages= c("binfutssr","psych","mvtnorm")) %dopar%
(cp(nsim = 1000,
alpha = 0.025,
beta = 0.2,
p_le = 0.3227348,
p_lc = 0.2,
p_se = p_se[i],
p_sc = 0.2,
n = 200,
fr_lo = 0.25,
fr_sh = 0.5,
phi_e = phi_e_v2[i],
phi_c = phi_c_v2[i],
c = seq(0, 1, 0.01)))

output_cp_alt_fr_sh_075_p_se <- foreach(i = 1:length(phi_e_v2),
.packages= c("binfutssr","psych","mvtnorm")) %dopar%
(cp(nsim = 1000,
alpha = 0.025,
beta = 0.2,
p_le = 0.3227348,
p_lc = 0.2,
p_se = p_se[i],
p_sc = 0.2,
n = 200,
fr_lo = 0.25,
fr_sh = 0.75,
phi_e = phi_e_v2[i],
phi_c = phi_c_v2[i],
c = seq(0, 1, 0.01)))

create_corr_plots_prob_stop_not_p_se(output_cp_alt_fr_sh_05_p_se = output_cp_alt_fr_sh_05_p_se,
output_cp_alt_fr_sh_075_p_se = output_cp_alt_fr_sh_075_p_se,
plots_corr = plots_corr,
phi_e = phi_e_v2,
phi_c = phi_c_v2,
fr_lo = 0.25,
filetype = "pdf")
```

`create_corr_plots_p_se` *Correlation Plots Overall Power*

Description

Obtain a plot with overall power for a range of correlations between S and L. The power is obtained for cut-off points for which probability to stop for futility under alternative hypothesis is equal to 10 true p_se is different than it was expected.v This is done for three estimators. Two cases are considered t_s = 0.5 and t_s = 0.75. The cut-off

Usage

```
create_corr_plots_p_se(output_cp_alt_fr_sh_05_p_se,
  output_cp_alt_fr_sh_075_p_se, plots_corr, phi_e, phi_c, fr_lo, filetype)
```

Arguments

`output_cp_alt_fr_sh_05_p_se`
: Output from simulation of a clinical trial under alternative hypothesis with fr_sh = 0.5 for different p_se

`output_cp_alt_fr_sh_075_p_se`
: Output from simulation of a clinical trial under alternative hypothesis with fr_sh = 0.75 for different p_se

`plots_corr`
: Output from 'create_corr_plots' function for p_se equal to p_le

`phi_e`
: Correlation between S and L in the treatment group

`phi_c`
: Correlation between S and L in the control group

`fr_lo`
: Amount of information available at interim for the long-term endpoint, value between 0 and 1

`filetype`
: - Filetype which the plot should be saved to. Options are: c("pdf", "eps", "none"). If none is chosen the plot is printed

Value

The function creates a plot with results of simulations based on the output from function cp(). It consists of 8 plots showing overall power for a case when the cut-off point is chosen based on probability to stop for futility under alternative hypothesis of 10"%" for different values of p_se assuming it could have been mis-specified.

Examples

```
cl <- makeCluster(1)
registerDoParallel(cl)

phi_e = c(0,0.2,0.5,0.7,0.9)
phi_c = c(0,0.2,0.5,0.7,0.9)

output_cp_alt_fr_sh_05 <- foreach(i = 1:length(phi_e), .packages="binfutssr") %dopar%
(cp(nsim = 1000,
alpha = 0.025,
beta = 0.2,
p_le = 0.3227348,
p_lc = 0.2,
p_se = 0.3227348,
p_sc = 0.2,
n = 200,
fr_lo = 0.25,
fr_sh = 0.5,
phi_e = phi_e[i],
phi_c = phi_c[i],
c = seq(0, 1, 0.01)))

output_cp_alt_fr_sh_075 <- foreach(i = 1:length(phi_e), .packages="binfutssr") %dopar%
 (cp(nsim = 1000,
 alpha = 0.025,
 beta = 0.2,
 p_le = 0.3227348,
 p_lc = 0.2,
 p_se = 0.3227348,
 p_sc = 0.2,
```

```
 n = 200,
 fr_lo = 0.25,
 fr_sh = 0.75,
 phi_e = phi_e[i],
 phi_c = phi_c[i],
 c = seq(0, 1, 0.01)))

plots_corr <- create_corr_plots(output_cp_alt_fr_sh_05 = output_cp_alt_fr_sh_05,
output_cp_alt_fr_sh_075 = output_cp_alt_fr_sh_075,
phi_e = phi_e,
phi_c = phi_c,
fr_lo = 0.25,
filetype = "none")

phi_e_v2 <- c(0, 0.2, 0.5, 0.7,
             0, 0.2, 0.5, 0.7, 0.9,
             0, 0.2, 0.5, 0.7, 0.9,
             0, 0.2, 0.5, 0.7, 0.9)

phi_c_v2 <- c(0, 0.2, 0.5, 0.7,
             0, 0.2, 0.5, 0.7, 0.9,
             0, 0.2, 0.5, 0.7, 0.9,
             0, 0.2, 0.5, 0.7, 0.9)

p_se <- c(rep(0.2, 4), rep(0.285, 5),
rep(0.3227348 ,5), rep(0.365, 5))

output_cp_alt_fr_sh_05_p_se <- foreach(i = 1:length(phi_e_v2),
.packages= c("binfutssr","psych","mvtnorm")) %dopar%
(cp(nsim = 1000,
alpha = 0.025,
beta = 0.2,
p_le = 0.3227348,
p_lc = 0.2,
p_se = p_se[i],
p_sc = 0.2,
n = 200,
fr_lo = 0.25,
fr_sh = 0.5,
phi_e = phi_e_v2[i],
phi_c = phi_c_v2[i],
c = seq(0, 1, 0.01)))

output_cp_alt_fr_sh_075_p_se <- foreach(i = 1:length(phi_e_v2),
.packages= c("binfutssr","psych","mvtnorm")) %dopar%
(cp(nsim = 1000,
alpha = 0.025,
beta = 0.2,
p_le = 0.3227348,
p_lc = 0.2,
p_se = p_se[i],
p_sc = 0.2,
n = 200,
fr_lo = 0.25,
fr_sh = 0.75,
phi_e = phi_e_v2[i],
phi_c = phi_c_v2[i],
```

```
c = seq(0, 1, 0.01)))

create_corr_plots_p_se(output_cp_alt_fr_sh_05_p_se = output_cp_alt_fr_sh_05_p_se,
output_cp_alt_fr_sh_075_p_se = output_cp_alt_fr_sh_075_p_se,
plots_corr = plots_corr,
phi_e = phi_e_v2,
phi_c = phi_c_v2,
fr_lo = 0.25,
filetype = "pdf")
```

`create_corr_plots_p_se_low` *Plot for Overall Power Different t and Correlation P_SE Low*

Description

Obtain a plot with overall power for a range of correlations between S and L. The power is obtained for cut-off points for which probability to stop for futility under alternative hypothesis is equal to 10 Two cases are considered t_s = 0.5 and t_s = 0.75. Here however, c was mis-specified as probability of success for S in experimental arm is different than expected.

Usage

```
create_corr_plots_p_se_low(output_cp_alt_fr_sh_05_p_se_02,
  output_cp_alt_fr_sh_075_p_se_02, plots_corr, phi_e, phi_c, fr_lo, filetype)
```

Arguments

`output_cp_alt_fr_sh_05_p_se_02`	Output from simulation of a clinical trial under alternative hypothesis with fr_sh = 0.5 assuming no effect in p_se
`output_cp_alt_fr_sh_075_p_se_02`	Output from simulation of a clinical trial under alternative hypothesis with fr_sh = 0.75 assuming no effect in p_se
`plots_corr`	Output from 'create_corr_plots' function for p_se equal to p_le
`phi_e`	Correlation between S and L in the treatment group
`phi_c`	Correlation between S and L in the control group
`fr_lo`	Amount of information available at interim for the long-term endpoint, value between 0 and 1
`filetype`	- Filetype which the plot should be saved to. Options are: c("pdf", "eps", "none"). If none is chosen the plot is printed

Value

The function creates a plot with results of simulations based on the output from function cp(). It consists of 2 plots showing overall power for a case when the cut-off point is chosen based on probability to stop for futility under alternative hypothesis of 10"%" but where true value of p_se is different than it was assumed

Examples

```
cl <- makeCluster(1)
registerDoParallel(cl)

phi_e = c(0,0.2,0.5,0.7,0.9)
phi_c = c(0,0.2,0.5,0.7,0.9)

output_cp_alt_fr_sh_05 <- foreach(i = 1:length(phi_e), .packages="binfutssr") %dopar%
(cp(nsim = 1000,
alpha = 0.025,
beta = 0.2,
p_le = 0.3227348,
p_lc = 0.2,
p_se = 0.3227348,
p_sc = 0.2,
n = 200,
fr_lo = 0.25,
fr_sh = 0.5,
phi_e = phi_e[i],
phi_c = phi_c[i],
c = seq(0, 1, 0.01)))

output_cp_alt_fr_sh_075 <- foreach(i = 1:length(phi_e), .packages="binfutssr") %dopar%
 (cp(nsim = 1000,
 alpha = 0.025,
 beta = 0.2,
 p_le = 0.3227348,
 p_lc = 0.2,
 p_se = 0.3227348,
 p_sc = 0.2,
 n = 200,
 fr_lo = 0.25,
 fr_sh = 0.75,
 phi_e = phi_e[i],
 phi_c = phi_c[i],
 c = seq(0, 1, 0.01)))

plots_corr <- create_corr_plots(output_cp_alt_fr_sh_05 = output_cp_alt_fr_sh_05,
output_cp_alt_fr_sh_075 = output_cp_alt_fr_sh_075,
phi_e = phi_e,
phi_c = phi_c,
fr_lo = 0.25,
filetype = "none")

phi_e_v2 <- c(0, 0.2, 0.5, 0.7)
phi_c_v2 <- c(0, 0.2, 0.5, 0.7)

output_cp_alt_fr_sh_05_p_se_02 <- foreach(i = 1:length(phi_e_v2), .packages="binfutssr") %dopar%
(cp(nsim = 1000,
alpha = 0.025,
beta = 0.2,
p_le = 0.3227348,
p_lc = 0.2,
p_se = 0.2,
p_sc = 0.2,
n = 200,
```

```
fr_lo = 0.25,
fr_sh = 0.5,
phi_e = phi_e_v2[i],
phi_c = phi_c_v2[i],
c = seq(0, 1, 0.01)))

output_cp_alt_fr_sh_075_p_se_02 <- foreach(i = 1:length(phi_e_v2), .packages="binfutssr") %dopar%
 (cp(nsim = 1000,
 alpha = 0.025,
 beta = 0.2,
 p_le = 0.3227348,
 p_lc = 0.2,
 p_se = 0.2,
 p_sc = 0.2,
 n = 200,
 fr_lo = 0.25,
 fr_sh = 0.75,
 phi_e = phi_e_v2[i],
 phi_c = phi_c_v2[i],
 c = seq(0, 1, 0.01)))

create_corr_plots_p_se_low(output_cp_alt_fr_sh_05_p_se_02 = output_cp_alt_fr_sh_05_p_se_02,
output_cp_alt_fr_sh_075_p_se_02 = output_cp_alt_fr_sh_075_p_se_02,
plots_corr = plots_corr,
phi_e = phi_e_v2,
phi_c = phi_c_v2,
fr_lo = 0.25,
filetype = "pdf")
```

`create_fs_plots` *Plot for Probability to Stop for Futility*

Description

Obtain a plot with probability to stop for futility showing 12 different scenarios for a trial stopping for futility based on conditional power with fixed and observed effect. Three estimators are compared: use of L only, S only and combination of both S and L. Four effects in the short-term outcome in experimental group are considered: no effect, moderate effect, equal effect and large effect compared to long-term outcome in experimental group.

Usage

```
create_fs_plots(output_cp_alt, output_cp_mod, output_cp_null, phi_e, phi_c,
  filetype)
```

Arguments

`output_cp_alt`	output from simulation of a clinical trial under alternative hypothesis
`output_cp_mod`	output from simulation of a clinical trial for moderate effects achieving ~50"%" power
`output_cp_null`	output from simulation of a clinical trial under the null hypothesis
`phi_e`	Correlation between S and L in the treatment group

phi_c	Correlation between S and L in the control group
filetype	filetype which the plot should be saved to; options are: c("pdf", "eps", "none"). If none is chosen the plot is printed

Value

The function creates a plot with results of simulations based on the output from functions cp_alt, cp_mod, cp_null. It is a 3x4 plot showing probability to stop for futility under the null hypothesis, for moderate results and under alternative hypothesis.

Examples

```
cl <- makeCluster(1)
registerDoParallel(cl)

p_se = c(0.2, 0.285, 0.3227348, 0.365)

output_cp_alt <- foreach(i = 1:length(p_se), .packages="binfutssr") %dopar%
(cp(nsim = 1000,
alpha = 0.025,
beta = 0.2,
p_le = 0.3227348,
p_lc = 0.2,
p_se = p_se[i],
p_sc = 0.2,
n = 200,
fr_lo = 0.25,
fr_sh = 0.5,
phi_e = 0.5,
phi_c = 0.5,
c = seq(0, 1, 0.01)))

output_cp_mod <- foreach(i = 1:length(p_se), .packages="binfutssr") %dopar%
(cp(nsim = 1000,
alpha = 0.025,
beta = 0.2,
p_le = 0.285,
p_lc = 0.2,
p_se = p_se[i],
p_sc = 0.2,
n = 200,
fr_lo = 0.25,
fr_sh = 0.5,
phi_e = 0.5,
phi_c = 0.5,
c = seq(0, 1, 0.01)))

output_cp_null <- foreach(i = 1:length(p_se), .packages="binfutssr") %dopar%
(cp(nsim = 1000,
alpha = 0.025,
beta = 0.2,
p_le = 0.2,
p_lc = 0.2,
p_se = p_se[i],
p_sc = 0.2,
n = 200,
```

```
fr_lo = 0.25,
fr_sh = 0.5,
phi_e = 0.5,
phi_c = 0.5,
c = seq(0, 1, 0.01)))

create_fs_plots(output_cp_alt = output_cp_alt,
output_cp_mod = output_cp_mod,
output_cp_null = output_cp_null,
phi_e = 0.5,
phi_c = 0.5,
filetype = "pdf")
```

---

create_fs_plots_nested
Nested Plots Probability to Stop for Futility

Description

Obtain a plot with probability to stop for futility showing 12 different scenarios for a trial stopping for futility based on conditional power with fixed and observed effect. Three estimators are compared: use of L only, S only and combination of both S and L. Four effects in the short-term outcome in experimental group are considered: no effect, moderate effect, equal effect and large effect compared to long-term outcome in experimental group.

Usage

```
create_fs_plots_nested(output_cp_alt, output_cp_mod, output_cp_null, phi_e,
  phi_c, filetype)
```

Arguments

output_cp_alt	Output from simulation of a clinical trial under alternative hypothesis
output_cp_mod	Output from simulation of a clinical trial for moderate effects achieving ~50"%" power
output_cp_null	Output from simulation of a clinical trial under the null hypothesis
phi_e	Correlation between S and L in the treatment group
phi_c	Correlation between S and L in the control group
filetype	Filetype which the plot should be saved to. O Options are: c("pdf", "eps", "none"). If none is chosen the plot is printed

Value

The function creates a plot with results of simulations based on the output from functions cp_alt, cp_mod, cp_null. It is a 3x4 plot showing probability to stop for futility under the null hypothesis, for moderate results and under alternative hypothesis.

Examples

```
cl <- makeCluster(1)
registerDoParallel(cl)

alpha <- 0.025
n <- 200
p_lc <- 0.2
p_sc <- 0.35
p_le <- 0.3227348
p_se <- c(p_sc, power.prop.test(n = n, p1 = p_sc, sig.level = alpha,
power = 0.5, alternative = "one.sided")$p2,
power.prop.test(n = n, p1 = p_sc, sig.level = alpha,
power = 0.8, alternative = "one.sided")$p2,
power.prop.test(n = n, p1 = p_sc, sig.level = alpha,
power = 0.9, alternative = "one.sided")$p2)

phi_e <- floor((((p_le)-(p_le) * (p_se)) /
sqrt((p_le) * (1 - (p_le)) * (1 - (p_se)) * (p_se))) * 1000000) / 1000000
phi_c <- floor((((p_lc) - (p_lc) * (p_sc)) /
sqrt((p_lc) * (1 - (p_lc)) * (1 - (p_sc)) * (p_sc))) * 1000000) / 1000000

output_cp_alt <- foreach(i = 1:length(p_se), .packages="binfutssr") %dopar%
(cp(nsim = 1000,
alpha = 0.025,
beta = 0.2,
p_le = 0.3227348,
p_lc = 0.2,
p_se = p_se[i],
p_sc = 0.35,
n = 200,
fr_lo = 0.25,
fr_sh = 0.5,
phi_e = phi_e[i],
phi_c = phi_c,
c = seq(0, 1, 0.01)))

output_cp_mod <- foreach(i = 1:length(p_se), .packages="binfutssr") %dopar%
(cp(nsim = 1000,
alpha = 0.025,
beta = 0.2,
p_le = 0.285,
p_lc = 0.2,
p_se = p_se[i],
p_sc = 0.35,
n = 200,
fr_lo = 0.25,
fr_sh = 0.5,
phi_e = phi_e[i],
phi_c = phi_c,
c = seq(0, 1, 0.01)))

output_cp_null <- foreach(i = 1:length(p_se), .packages="binfutssr") %dopar%
(cp(nsim = 1000,
alpha = 0.025,
beta = 0.2,
p_le = 0.2,
```

```
p_lc = 0.2,
p_se = p_se[i],
p_sc = 0.35,
n = 200,
fr_lo = 0.25,
fr_sh = 0.5,
phi_e = phi_e[i],
phi_c = phi_c,
c = seq(0, 1, 0.01)))

create_fs_plots_nested(output_cp_alt = output_cp_alt,
output_cp_mod = output_cp_mod,
output_cp_null = output_cp_null,
phi_e = phi_e,
phi_c = phi_c,
filetype = "pdf")
```

---

create_power_plots *Plot for Overall Power*

Description

Obtain a plot with overall power/type 1 error showing 12 different scenarios for a trial stopping for futility based on conditional power with fixed and observed effect. Three estimators are compared: use of L only, S only and combination of both S and L. Four effects in the short-term outcome in experimental group are considered: no effect, moderate effect, equal effect and large effect compared to long-term outcome in experimental group.

Usage

```
create_power_plots(output_cp_alt, output_cp_mod, output_cp_null, phi_e, phi_c,
  filetype)
```

Arguments

output_cp_alt	Output from simulation of a clinical trial under alternative hypothesis
output_cp_mod	Output from simulation of a clinical trial for moderate effects achieving ~50"%" power
output_cp_null	Output from simulation of a clinical trial under the null hypothesis
phi_e	Correlation between S and L in the treatment group
phi_c	Correlation between S and L in the control group
filetype	Filetype which the plot should be saved to. O Options are: c("pdf", "eps", "none"). If none is chosen the plot is printed

Value

The function creates a plot with results of simulations based on the output from functions cp_alt, cp_mod, cp_null. It is a 3x4 plot showing type 1 error and overall power under the null hypothesis, for moderate results and under alternative hypothesis.

Examples

```
cl <- makeCluster(1)
registerDoParallel(cl)

p_se <- c(0.2, 0.285, 0.3227348, 0.365)

output_cp_alt <- foreach(i = 1:length(p_se), .packages="binfutssr") %dopar%
(cp(nsim = 1000,
alpha = 0.025,
beta = 0.2,
p_le = 0.3227348,
p_lc = 0.2,
p_se = p_se[i],
p_sc = 0.2,
n = 200,
fr_lo = 0.25,
fr_sh = 0.5,
phi_e = 0.5,
phi_c = 0.5,
c = seq(0, 1, 0.01)))

output_cp_mod <- foreach(i = 1:length(p_se), .packages="binfutssr") %dopar%
(cp(nsim = 1000,
alpha = 0.025,
beta = 0.2,
p_le = 0.285,
p_lc = 0.2,
p_se = p_se[i],
p_sc = 0.2,
n = 200,
fr_lo = 0.25,
fr_sh = 0.5,
phi_e = 0.5,
phi_c = 0.5,
c = seq(0, 1, 0.01)))

output_cp_null <- foreach(i = 1:length(p_se), .packages="binfutssr") %dopar%
(cp(nsim = 1000,
alpha = 0.025,
beta = 0.2,
p_le = 0.2,
p_lc = 0.2,
p_se = p_se[i],
p_sc = 0.2,
n = 200,
fr_lo = 0.25,
fr_sh = 0.5,
phi_e = 0.5,
phi_c = 0.5,
c = seq(0, 1, 0.01)))

create_power_plots(output_cp_alt = output_cp_alt,
output_cp_mod = output_cp_mod,
output_cp_null = output_cp_null,
phi_e = 0.5,
phi_c = 0.5,
```

```
filetype = "pdf")
```

---

create_power_plots_nested *Plots Nested Overall Power*

Description

Obtain a plot with overall power/type 1 error showing 12 different scenarios for a trial stopping for futility based on conditional power with fixed and observed effect. Three estimators are compared: use of L only, S only and combination of both S and L. Four effects in the short-term outcome in experimental group are considered: no effect, moderate effect, equal effect and large effect compared to long-term outcome in experimental group.

Usage

```
create_power_plots_nested(output_cp_alt, output_cp_mod, output_cp_null, phi_e,
  phi_c, filetype)
```

Arguments

output_cp_alt	Output from simulation of a clinical trial under alternative hypothesis
output_cp_mod	Output from simulation of a clinical trial for moderate effects achieving ~50"%" power
output_cp_null	Output from simulation of a clinical trial under the null hypothesis
phi_e	Correlation between S and L in the treatment group
phi_c	Correlation between S and L in the control group
filetype	Filetype which the plot should be saved to. O Options are: c("pdf", "eps", "none"). If none is chosen the plot is printed

Value

The function creates a plot with results of simulations based on the output from functions cp_alt, cp_mod, cp_null. It is a 3x4 plot showing type 1 error and overall power under the null hypothesis, for moderate results and under alternative hypothesis.

Examples

```
cl <- makeCluster(1)
registerDoParallel(cl)

alpha <- 0.025
n <- 200
p_lc <- 0.2
p_sc <- 0.35
p_le <- 0.3227348
p_se <- c(p_sc, power.prop.test(n = n, p1 = p_sc, sig.level = alpha,
power = 0.5, alternative = "one.sided")$p2,
power.prop.test(n = n, p1 = p_sc, sig.level = alpha,
power = 0.8, alternative = "one.sided")$p2,
power.prop.test(n = n, p1 = p_sc, sig.level = alpha,
```

```
power = 0.9, alternative = "one.sided")$p2)

phi_e <- floor((((p_le)-(p_le) * (p_se)) /
sqrt((p_le) * (1 - (p_le)) * (1 - (p_se)) * (p_se))) * 1000000) / 1000000
phi_c <- floor((((p_lc) - (p_lc) * (p_sc)) /
sqrt((p_lc) * (1 - (p_lc)) * (1 - (p_sc)) * (p_sc))) * 1000000) / 1000000

output_cp_alt <- foreach(i = 1:length(p_se), .packages="binfutssr") %dopar%
(cp(nsim = 1000,
alpha = 0.025,
beta = 0.2,
p_le = 0.3227348,
p_lc = 0.2,
p_se = p_se[i],
p_sc = 0.35,
n = 200,
fr_lo = 0.25,
fr_sh = 0.5,
phi_e = phi_e[i],
phi_c = phi_c,
c = seq(0, 1, 0.01)))

output_cp_mod <- foreach(i = 1:length(p_se), .packages="binfutssr") %dopar%
(cp(nsim = 1000,
alpha = 0.025,
beta = 0.2,
p_le = 0.285,
p_lc = 0.2,
p_se = p_se[i],
p_sc = 0.35,
n = 200,
fr_lo = 0.25,
fr_sh = 0.5,
phi_e = phi_e[i],
phi_c = phi_c,
c = seq(0, 1, 0.01)))

output_cp_null <- foreach(i = 1:length(p_se), .packages="binfutssr") %dopar%
(cp(nsim = 1000,
alpha = 0.025,
beta = 0.2,
p_le = 0.2,
p_lc = 0.2,
p_se = p_se[i],
p_sc = 0.35,
n = 200,
fr_lo = 0.25,
fr_sh = 0.5,
phi_e = phi_e[i],
phi_c = phi_c,
c = seq(0, 1, 0.01)))

create_power_plots_nested(output_cp_alt = output_cp_alt,
output_cp_mod = output_cp_mod,
output_cp_null = output_cp_null,
phi_e = phi_e,
phi_c = phi_c,
```

```
filetype = "pdf")
```

---

create_prob_cont_rej_plots
Plot Probability to Stop for Futility

Description

Obtain a plot with probability to reject the null hypothesis given the trial was continued showing 12 different scenarios for a trial stopping for futility based on conditional power with fixed and observed effect. Three estimators are compared: use of L only, S only and combination of both S and L. Four effects in the short-term outcome in experimental group are considered: no effect, moderate effect, equal effect and large effect compared to long-term outcome in experimental group.

Usage

```
create_prob_cont_rej_plots(output_cp_alt, output_cp_mod, output_cp_null, phi_e,
  phi_c, filetype)
```

Arguments

output_cp_alt	output from simulation of a clinical trial under alternative hypothesis
output_cp_mod	output from simulation of a clinical trial for moderate effects achieving ~50"%" power
output_cp_null	output from simulation of a clinical trial under the null hypothesis
phi_e	Correlation between S and L in the treatment group
phi_c	Correlation between S and L in the control group
filetype	filetype which the plot should be saved to; options are: c("pdf", "eps", "none"). If none is chosen the plot is printed

Value

The function creates a plot with results of simulations based on the output from functions cp_alt, cp_mod, cp_null. It is a 3x4 plot showing probability to reject the null hypothesis given the trial was continued under the null hypothesis, for moderate results and under alternative hypothesis.

Examples

```
cl <- makeCluster(1)
registerDoParallel(cl)

p_se = c(0.2, 0.285, 0.3227348, 0.365)

output_cp_alt <- foreach(i = 1:length(p_se), .packages="binfutssr") %dopar%
(cp(nsim = 1000,
alpha = 0.025,
beta = 0.2,
p_le = 0.3227348,
p_lc = 0.2,
p_se = p_se[i],
p_sc = 0.2,
```

```
n = 200,
fr_lo = 0.25,
fr_sh = 0.5,
phi_e = 0.5,
phi_c = 0.5,
c = seq(0, 1, 0.01)))

output_cp_mod <- foreach(i = 1:length(p_se), .packages="binfutssr") %dopar%
(cp(nsim = 1000,
alpha = 0.025,
beta = 0.2,
p_le = 0.285,
p_lc = 0.2,
p_se = p_se[i],
p_sc = 0.2,
n = 200,
fr_lo = 0.25,
fr_sh = 0.5,
phi_e = 0.5,
phi_c = 0.5,
c = seq(0, 1, 0.01)))

output_cp_null <- foreach(i = 1:length(p_se), .packages="binfutssr") %dopar%
(cp(nsim = 1000,
alpha = 0.025,
beta = 0.2,
p_le = 0.2,
p_lc = 0.2,
p_se = p_se[i],
p_sc = 0.2,
n = 200,
fr_lo = 0.25,
fr_sh = 0.5,
phi_e = 0.5,
phi_c = 0.5,
c = seq(0, 1, 0.01)))

create_prob_cont_rej_plots(output_cp_alt = output_cp_alt,
output_cp_mod = output_cp_mod,
output_cp_null = output_cp_null,
phi_e = 0.5,
phi_c = 0.5,
filetype = "pdf")
```

---

create_prob_cont_rej_plots_nested

Plots Nested Probability to Reject the Null Given Trial was Continued

---

Description

Obtain a plot with probability to reject the null hypothesis given the trial was continued showing 12 different scenarios for a trial stopping for futility based on conditional power with fixed and observed effect. Three estimators are compared: use of L only, S only and combination of both S and L. Four effects in the short-term outcome in experimental group are considered: no effect, moderate effect, equal effect and large effect compared to long-term outcome in experimental group.

Usage

```
create_prob_cont_rej_plots_nested(output_cp_alt, output_cp_mod, output_cp_null,
  phi_e, phi_c, filetype)
```

Arguments

output_cp_alt	Output from simulation of a clinical trial under alternative hypothesis
output_cp_mod	Output from simulation of a clinical trial for moderate effects achieving ~50"%" power
output_cp_null	Output from simulation of a clinical trial under the null hypothesis
phi_e	Correlation between S and L in the treatment group
phi_c	Correlation between S and L in the control group
filetype	Filetype which the plot should be saved to. O Options are: c("pdf", "eps", "none"). If none is chosen the plot is printed

Value

The function creates a plot with results of simulations based on the output from functions cp_alt, cp_mod, cp_null. It is a 3x4 plot showing probability to reject the null hypothesis given the trial was continued under the null hypothesis, for moderate results and under alternative hypothesis.

Examples

```
cl <- makeCluster(1)
registerDoParallel(cl)

alpha <- 0.025
n <- 200
p_lc <- 0.2
p_sc <- 0.35
p_le <- 0.3227348
p_se <- c(p_sc, power.prop.test(n = n, p1 = p_sc, sig.level = alpha,
power = 0.5, alternative = "one.sided")$p2,
power.prop.test(n = n, p1 = p_sc, sig.level = alpha,
power = 0.8, alternative = "one.sided")$p2,
power.prop.test(n = n, p1 = p_sc, sig.level = alpha,
power = 0.9, alternative = "one.sided")$p2)

phi_e <- floor((((p_le)-(p_le) * (p_se)) /
sqrt((p_le) * (1 - (p_le)) * (1 - (p_se)) * (p_se))) * 1000000) / 1000000
phi_c <- floor((((p_lc) - (p_lc) * (p_sc)) /
sqrt((p_lc) * (1 - (p_lc)) * (1 - (p_sc)) * (p_sc))) * 1000000) / 1000000

output_cp_alt <- foreach(i = 1:length(p_se), .packages="binfutssr") %dopar%
(cp(nsim = 1000,
alpha = 0.025,
beta = 0.2,
p_le = 0.3227348,
p_lc = 0.2,
p_se = p_se[i],
p_sc = 0.35,
n = 200,
fr_lo = 0.25,
fr_sh = 0.5,
```

```
phi_e = phi_e[i],
phi_c = phi_c,
c = seq(0, 1, 0.01)))

output_cp_mod <- foreach(i = 1:length(p_se), .packages="binfutssr") %dopar%
(cp(nsim = 1000,
alpha = 0.025,
beta = 0.2,
p_le = 0.285,
p_lc = 0.2,
p_se = p_se[i],
p_sc = 0.35,
n = 200,
fr_lo = 0.25,
fr_sh = 0.5,
phi_e = phi_e[i],
phi_c = phi_c,
c = seq(0, 1, 0.01)))

output_cp_null <- foreach(i = 1:length(p_se), .packages="binfutssr") %dopar%
(cp(nsim = 1000,
alpha = 0.025,
beta = 0.2,
p_le = 0.2,
p_lc = 0.2,
p_se = p_se[i],
p_sc = 0.35,
n = 200,
fr_lo = 0.25,
fr_sh = 0.5,
phi_e = phi_e[i],
phi_c = phi_c,
c = seq(0, 1, 0.01)))

create_prob_cont_rej_plots_nested(output_cp_alt = output_cp_alt,
output_cp_mod = output_cp_mod,
output_cp_null = output_cp_null,
phi_e = phi_e,
phi_c = phi_c,
filetype = "pdf")
```

---

create_prob_corr_dec_plots
Plot Probability to Make the Correct Decision

Description

Obtain a plot with probability of making the correct decision showing 12 different scenarios for a trial stopping for futility based on conditional power with fixed and observed effect. Three estimators are compared: use of L only, S only and combination of both S and L. Four effects in the short-term outcome in experimental group are considered: no effect, moderate effect, equal effect and large effect compared to long-term outcome in experimental group.

Usage

```
create_prob_corr_dec_plots(output_cp_alt, output_cp_mod, output_cp_null, phi_e,
  phi_c, filetype)
```

Arguments

`output_cp_alt`	output from simulation of a clinical trial under alternative hypothesis
`output_cp_mod`	output from simulation of a clinical trial for moderate effects achieving ~50"%" power
`output_cp_null`	output from simulation of a clinical trial under the null hypothesis
`phi_e`	Correlation between S and L in the treatment group
`phi_c`	Correlation between S and L in the control group
`filetype`	filetype which the plot should be saved to; options are: c("pdf", "eps", "none"). If none is chosen the plot is printed

Value

The function creates a plot with results of simulations based on the output from functions cp_alt, cp_mod, cp_null. It is a 3x4 plot showing probability to make the correct decision under the null hypothesis, for moderate results and under alternative hypothesis.

Examples

```
cl <- makeCluster(1)
registerDoParallel(cl)

p_se = c(0.2, 0.285, 0.3227348, 0.365)

output_cp_alt <- foreach(i = 1:length(p_se), .packages="binfutssr") %dopar%
(cp(nsim = 1000,
alpha = 0.025,
beta = 0.2,
p_le = 0.3227348,
p_lc = 0.2,
p_se = p_se[i],
p_sc = 0.2,
n = 200,
fr_lo = 0.25,
fr_sh = 0.5,
phi_e = 0.5,
phi_c = 0.5,
c = seq(0, 1, 0.01)))

output_cp_mod <- foreach(i = 1:length(p_se), .packages="binfutssr") %dopar%
(cp(nsim = 1000,
alpha = 0.025,
beta = 0.2,
p_le = 0.285,
p_lc = 0.2,
p_se = p_se[i],
p_sc = 0.2,
n = 200,
fr_lo = 0.25,
fr_sh = 0.5,
```

```
phi_e = 0.5,
phi_c = 0.5,
c = seq(0, 1, 0.01)))

output_cp_null <- foreach(i = 1:length(p_se), .packages="binfutssr") %dopar%
(cp(nsim = 1000,
alpha = 0.025,
beta = 0.2,
p_le = 0.2,
p_lc = 0.2,
p_se = p_se[i],
p_sc = 0.2,
n = 200,
fr_lo = 0.25,
fr_sh = 0.5,
phi_e = 0.5,
phi_c = 0.5,
c = seq(0, 1, 0.01)))

create_prob_corr_dec_plots(output_cp_alt = output_cp_alt,
output_cp_mod = output_cp_mod,
output_cp_null = output_cp_null,
phi_e = 0.5,
phi_c = 0.5,
filetype = "pdf")
```

---

create_prob_corr_dec_plots_nested
Plots Nested Correct Decision

Description

Obtain a plot with probability of making the correct decision showing 12 different scenarios for a trial stopping for futility based on conditional power with fixed and observed effect. Three estimators are compared: use of L only, S only and combination of both S and L. Four effects in the short-term outcome in experimental group are considered: no effect, moderate effect, equal effect and large effect compared to long-term outcome in experimental group.

Usage

```
create_prob_corr_dec_plots_nested(output_cp_alt, output_cp_mod, output_cp_null,
  phi_e, phi_c, filetype)
```

Arguments

output_cp_alt	Output from simulation of a clinical trial under alternative hypothesis
output_cp_mod	Output from simulation of a clinical trial for moderate effects achieving ~50"%" power
output_cp_null	Output from simulation of a clinical trial under the null hypothesis
phi_e	Correlation between S and L in the treatment group
phi_c	Correlation between S and L in the control group
filetype	Filetype which the plot should be saved to. O Options are: c("pdf", "eps", "none"). If none is chosen the plot is printed

Value

The function creates a plot with results of simulations based on the output from functions cp_alt, cp_mod, cp_null. It is a 3x4 plot showing probability of making the correct decision under the null hypothesis, for moderate results and under alternative hypothesis.

Examples

```
cl <- makeCluster(1)
registerDoParallel(cl)

alpha <- 0.025
n <- 200
p_lc <- 0.2
p_sc <- 0.35
p_le <- 0.3227348
p_se <- c(p_sc, power.prop.test(n = n, p1 = p_sc, sig.level = alpha,
power = 0.5, alternative = "one.sided")$p2,
power.prop.test(n = n, p1 = p_sc, sig.level = alpha,
power = 0.8, alternative = "one.sided")$p2,
power.prop.test(n = n, p1 = p_sc, sig.level = alpha,
power = 0.9, alternative = "one.sided")$p2)

phi_e <- floor((((p_le)-(p_le) * (p_se)) /
sqrt((p_le) * (1 - (p_le)) * (1 - (p_se)) * (p_se))) * 1000000) / 1000000
phi_c <- floor((((p_lc) - (p_lc) * (p_sc)) /
sqrt((p_lc) * (1 - (p_lc)) * (1 - (p_sc)) * (p_sc))) * 1000000) / 1000000

output_cp_alt <- foreach(i = 1:length(p_se), .packages="binfutssr") %dopar%
(cp(nsim = 1000,
alpha = 0.025,
beta = 0.2,
p_le = 0.3227348,
p_lc = 0.2,
p_se = p_se[i],
p_sc = 0.35,
n = 200,
fr_lo = 0.25,
fr_sh = 0.5,
phi_e = phi_e[i],
phi_c = phi_c,
c = seq(0, 1, 0.01)))

output_cp_mod <- foreach(i = 1:length(p_se), .packages="binfutssr") %dopar%
(cp(nsim = 1000,
alpha = 0.025,
beta = 0.2,
p_le = 0.285,
p_lc = 0.2,
p_se = p_se[i],
p_sc = 0.35,
n = 200,
fr_lo = 0.25,
fr_sh = 0.5,
phi_e = phi_e[i],
phi_c = phi_c,
c = seq(0, 1, 0.01)))
```

```
output_cp_null <- foreach(i = 1:length(p_se), .packages="binfutssr") %dopar%
(cp(nsim = 1000,
alpha = 0.025,
beta = 0.2,
p_le = 0.2,
p_lc = 0.2,
p_se = p_se[i],
p_sc = 0.35,
n = 200,
fr_lo = 0.25,
fr_sh = 0.5,
phi_e = phi_e[i],
phi_c = phi_c,
c = seq(0, 1, 0.01)))

create_prob_corr_dec_plots_nested(output_cp_alt = output_cp_alt,
output_cp_mod = output_cp_mod,
output_cp_null = output_cp_null,
phi_e = phi_e,
phi_c = phi_c,
filetype = "pdf")
```

---

create_prob_stop_not_rej_plots

Plot Probabilty Not to Reject Given Trial was Stopped

Description

Obtain a plot with probability of having rejected the null hypothesis given the trial was stopped had it been continued showing 12 different scenarios for a trial stopping for futility based on conditional power with fixed and observed effect. Three estimators are compared: use of L only, S only and combination of both S and L. Four effects in the short-term outcome in experimental group are considered: no effect, moderate effect, equal effect and large effect compared to long-term outcome in experimental group.

Usage

```
create_prob_stop_not_rej_plots(output_cp_alt, output_cp_mod, output_cp_null,
  phi_e, phi_c, filetype)
```

Arguments

`output_cp_alt`	output from simulation of a clinical trial under alternative hypothesis
`output_cp_mod`	output from simulation of a clinical trial for moderate effects achieving ~50"%" power
`output_cp_null`	output from simulation of a clinical trial under the null hypothesis
`phi_e`	Correlation between S and L in the treatment group
`phi_c`	Correlation between S and L in the control group
`filetype`	filetype which the plot should be saved to; options are: c("pdf", "eps", "none"). If none is chosen the plot is printed

Value

The function creates a plot with results of simulations based on the output from functions cp_alt, cp_mod, cp_null. It is a 3x4 plot showing probability of having rejected the null hypothesis given the trial was stopped had it been continued under the null hypothesis, for moderate results and under alternative hypothesis.

Examples

```
cl <- makeCluster(1)
registerDoParallel(cl)

p_se = c(0.2, 0.285, 0.3227348, 0.365)

output_cp_alt <- foreach(i = 1:length(p_se), .packages="binfutssr") %dopar%
(cp(nsim = 1000,
alpha = 0.025,
beta = 0.2,
p_le = 0.3227348,
p_lc = 0.2,
p_se = p_se[i],
p_sc = 0.2,
n = 200,
fr_lo = 0.25,
fr_sh = 0.5,
phi_e = 0.5,
phi_c = 0.5,
c = seq(0, 1, 0.01)))

output_cp_mod <- foreach(i = 1:length(p_se), .packages="binfutssr") %dopar%
(cp(nsim = 1000,
alpha = 0.025,
beta = 0.2,
p_le = 0.285,
p_lc = 0.2,
p_se = p_se[i],
p_sc = 0.2,
n = 200,
fr_lo = 0.25,
fr_sh = 0.5,
phi_e = 0.5,
phi_c = 0.5,
c = seq(0, 1, 0.01)))

output_cp_null <- foreach(i = 1:length(p_se), .packages="binfutssr") %dopar%
(cp(nsim = 1000,
alpha = 0.025,
beta = 0.2,
p_le = 0.2,
p_lc = 0.2,
p_se = p_se[i],
p_sc = 0.2,
n = 200,
fr_lo = 0.25,
fr_sh = 0.5,
phi_e = 0.5,
phi_c = 0.5,
```

```
c = seq(0, 1, 0.01)))

create_prob_stop_not_rej_plots(output_cp_alt = output_cp_alt,
output_cp_mod = output_cp_mod,
output_cp_null = output_cp_null,
phi_e = 0.5,
phi_c = 0.5,
filetype = "pdf")
```

---

create_prob_stop_not_rej_plots_nested
Plots Nested Probability Not to Reject Given Trial was Stopped

---

Description

Obtain a plot with probability of having rejected the null hypothesis given the trial was stopped showing 12 different scenarios for a trial stopping for futility based on conditional power with fixed and observed effect. Three estimators are compared: use of L only, S only and combination of both S and L. Four effects in the short-term outcome in experimental group are considered: no effect, moderate effect, equal effect and large effect compared to long-term outcome in experimental group.

Usage

```
create_prob_stop_not_rej_plots_nested(output_cp_alt, output_cp_mod,
  output_cp_null, phi_e, phi_c, filetype)
```

Arguments

output_cp_alt	Output from simulation of a clinical trial under alternative hypothesis
output_cp_mod	Output from simulation of a clinical trial for moderate effects achieving ~50"%" power
output_cp_null	Output from simulation of a clinical trial under the null hypothesis
phi_e	Correlation between S and L in the treatment group
phi_c	Correlation between S and L in the control group
filetype	Filetype which the plot should be saved to. O Options are: c("pdf", "eps", "none"). If none is chosen the plot is printed

Value

The function creates a plot with results of simulations based on the output from functions cp_alt, cp_mod, cp_null. It is a 3x4 plot showing probability of having rejected the null hypothesis given the trial was stopped under the null hypothesis, for moderate results and under alternative hypothesis.

Examples

```
cl <- makeCluster(1)
registerDoParallel(cl)

alpha <- 0.025
n <- 200
```

```
p_lc <- 0.2
p_sc <- 0.35
p_le <- 0.3227348
p_se <- c(p_sc, power.prop.test(n = n, p1 = p_sc, sig.level = alpha,
power = 0.5, alternative = "one.sided")$p2,
power.prop.test(n = n, p1 = p_sc, sig.level = alpha,
power = 0.8, alternative = "one.sided")$p2,
power.prop.test(n = n, p1 = p_sc, sig.level = alpha,
power = 0.9, alternative = "one.sided")$p2)

phi_e <- floor((((p_le)-(p_le) * (p_se)) /
sqrt((p_le) * (1 - (p_le)) * (1 - (p_se)) * (p_se))) * 1000000) / 1000000
phi_c <- floor((((p_lc) - (p_lc) * (p_sc)) /
sqrt((p_lc) * (1 - (p_lc)) * (1 - (p_sc)) * (p_sc))) * 1000000) / 1000000

output_cp_alt <- foreach(i = 1:length(p_se), .packages="binfutssr") %dopar%
(cp(nsim = 1000,
alpha = 0.025,
beta = 0.2,
p_le = 0.3227348,
p_lc = 0.2,
p_se = p_se[i],
p_sc = 0.35,
n = 200,
fr_lo = 0.25,
fr_sh = 0.5,
phi_e = phi_e[i],
phi_c = phi_c,
c = seq(0, 1, 0.01)))

output_cp_mod <- foreach(i = 1:length(p_se), .packages="binfutssr") %dopar%
(cp(nsim = 1000,
alpha = 0.025,
beta = 0.2,
p_le = 0.285,
p_lc = 0.2,
p_se = p_se[i],
p_sc = 0.35,
n = 200,
fr_lo = 0.25,
fr_sh = 0.5,
phi_e = phi_e[i],
phi_c = phi_c,
c = seq(0, 1, 0.01)))

output_cp_null <- foreach(i = 1:length(p_se), .packages="binfutssr") %dopar%
(cp(nsim = 1000,
alpha = 0.025,
beta = 0.2,
p_le = 0.2,
p_lc = 0.2,
p_se = p_se[i],
p_sc = 0.35,
n = 200,
fr_lo = 0.25,
fr_sh = 0.5,
phi_e = phi_e[i],
```

```
phi_c = phi_c,
c = seq(0, 1, 0.01)))

create_prob_stop_not_rej_plots_nested(output_cp_alt = output_cp_alt,
output_cp_mod = output_cp_mod,
output_cp_null = output_cp_null,
phi_e = phi_e,
phi_c = phi_c,
filetype = "pdf")
```

---

create_ssr_fix_cp_table

Table Sample Size Reassessment Based on Fixed CP, c Stopping rule

---

Description

Function creating a latex table showing operating characteristics of a trial with sample size reassessment based on conditional power. Futility stopping is based on the cut-off point of fixed effect conditional power.

Usage

```
create_ssr_fix_cp_table(output_cp, phi_e, phi_c, save)
```

Arguments

output_cp	Output from simulation of a clinical trial with sample size reassessment and stopping based on c
phi_e	Correlation between S and L in the treatment group
phi_c	Correlation between S and L in the control group
save	If TRUE then the latex output is saved into a .txt file. Otherwise it is printed

Value

The function creates a table with operating characteristics: overall power, probability to stop for futility and average sample size with its standard deviation (brackets)

Examples

```
cl <- makeCluster(1)
registerDoParallel(cl)

p_le <- c(0.2, 0.285, 0.3227348, 0.365)
p_se <- c(0.2, 0.285, 0.3227348, 0.365)

output_cp <- foreach(i = 1:length(p_le), .combine = rbind, .packages="binfutssr") %dopar%
ssr_fixed_cp(nsim = 1000,
alpha = 0.025,
beta = 0.2,
p_le = p_le[i],
p_lc = 0.2,
p_se = p_se[i],
```

```
p_sc = 0.2,
n = 200,
fr_lo = 0.25,
fr_sh = 0.5,
phi_e = 0.5,
phi_c = 0.5,
c = 0.3)

create_ssr_fix_cp_table(output_cp = output_cp,
    phi_e = 0.5,
    phi_c = 0.5,
save = FALSE)
```

`create_ssr_fix_pval_table` *Table Sample Size Reassessment Fixed Effect, p-val Stopping*

Description

Function creating a latex table showing operating characteristics of a trial with sample size reassessment based on conditional power. Futility stopping is based on the p-value of one of the estimators.

Usage

```
create_ssr_fix_pval_table(output_pval_zl, output_pval_zb, output_pval_zs, pval,
  save)
```

Arguments

output_pval_zl	Output from simulation of a clinical trial with sample size reassessment and stopping based on p-value of p_l
output_pval_zb	Output from simulation of a clinical trial with sample size reassessment and stopping based on p-value of p_b
output_pval_zs	Output from simulation of a clinical trial with sample size reassessment and stopping based on p-value of p_s
pval	P-value which will act as the stopping rule benchmark. Takes values from 0 to 1
save	If TRUE then the latex output is saved into a .txt file. Otherwise it is printed

Value

The function creates a table with operating characteristics: overall power, probability to stop for futility and average sample size with its standard deviation (brackets)

Examples

```
cl <- makeCluster(1)
registerDoParallel(cl)

p_le <- c(0.2, 0.285, 0.3227348, 0.365)
p_se <- c(0.2, 0.285, 0.3227348, 0.365)

output_pval_zl <- foreach(i = 1:length(p_le), .combine = rbind, .packages="binfutssr") %dopar%
```

```
ssr_fixed_z(nsim = 1000,
alpha = 0.025,
beta = 0.2,
p_le = p_le[i],
p_lc = 0.2,
p_se = p_se[i],
p_sc = 0.2,
n = 200,
fr_lo = 0.25,
fr_sh = 0.5,
phi_e = 0.5,
phi_c = 0.5,
pval = 0.45,
stop_type = "zl")

output_pval_zb <- foreach(i = 1:length(p_le), .combine = rbind, .packages="binfutssr") %dopar%
ssr_fixed_z(nsim = 1000,
alpha = 0.025,
beta = 0.2,
p_le = p_le[i],
p_lc = 0.2,
p_se = p_se[i],
p_sc = 0.2,
n = 200,
fr_lo = 0.25,
fr_sh = 0.5,
phi_e = 0.5,
phi_c = 0.5,
pval = 0.45,
stop_type = "zb")

output_pval_zs <- foreach(i = 1:length(p_le), .combine = rbind, .packages="binfutssr") %dopar%
ssr_fixed_z(nsim = 1000,
alpha = 0.025,
beta = 0.2,
p_le = p_le[i],
p_lc = 0.2,
p_se = p_se[i],
p_sc = 0.2,
n = 200,
fr_lo = 0.25,
fr_sh = 0.5,
phi_e = 0.5,
phi_c = 0.5,
pval = 0.45,
stop_type = "zs")


create_ssr_fix_pval_table(output_pval_zl = output_pval_zl,
output_pval_zb = output_pval_zb,
output_pval_zs = output_pval_zs,
pval = 0.45,
save=TRUE)
```

create_ssr_fix_pval_weight_table
Table Sample Size Reassessment Based on Fixed CP, p-val Stopping rule, Equal Weight

Description

Function creating a latex table showing operating characteristics of a trial with sample size reassessment based on conditional power. The futility stopping is based the p-value of a given estimator after the first stage. The weight for the combination function for all the estimators is set to be equal. If the trial is continued, e.g. p_l<pval, sample size reassessment is performed. The procedure is based on the fixed effect conditional power.

Usage

```
create_ssr_fix_pval_weight_table(output_pval_w_zl, output_pval_w_zb,
  output_pval_w_zs, w, pval, save)
```

Arguments

`output_pval_w_zl`
: Output from simulation of a clinical trial with sample size reassessment and stopping based on p-value of p_l

`output_pval_w_zb`
: Output from simulation of a clinical trial with sample size reassessment and stopping based on p-value of p_b

`output_pval_w_zs`
: Output from simulation of a clinical trial with sample size reassessment and stopping based on p-value of p_s

`w`
: Weight to be set to the combination function

`pval`
: P-value which will act as the stopping rule benchmark. Takes values from 0 to 1

`save`
: If TRUE then the latex output is saved into a .txt file. Otherwise it is printed

Value

The function creates a table with operating characteristics: overall power, probability to stop for futility and average sample size with its standard deviation (brackets). Different set of weights are considered here, varying from 0 to 1.

Examples

```
cl <- makeCluster(1)
registerDoParallel(cl)

w <- seq(0, 1, 0.1)

output_pval_w_zl <- foreach(i = 1:length(w), .combine = rbind, .packages="binfutssr") %dopar%
ssr_fixed_z_weight(nsim = 1000,
alpha = 0.025,
beta = 0.2,
p_le = 0.3227348,
p_lc = 0.2,
```

```
p_se = 0.3227348,
p_sc = 0.2,
n = 200,
fr_lo = 0.25,
fr_sh = 0.5,
phi_e = 0.5,
phi_c = 0.5,
pval = 0.45,
w = w[i],
stop_type = "zl")

output_pval_w_zb <- foreach(i = 1:length(w), .combine = rbind, .packages="binfutssr") %dopar%
ssr_fixed_z_weight(nsim = 1000,
alpha = 0.025,
beta = 0.2,
p_le = 0.3227348,
p_lc = 0.2,
p_se = 0.3227348,
p_sc = 0.2,
n = 200,
fr_lo = 0.25,
fr_sh = 0.5,
phi_e = 0.5,
phi_c = 0.5,
pval = 0.45,
w = w[i],
stop_type = "zb")

output_pval_w_zs <- foreach(i = 1:length(w), .combine = rbind, .packages="binfutssr") %dopar%
ssr_fixed_z_weight(nsim = 1000,
alpha = 0.025,
beta = 0.2,
p_le = 0.3227348,
p_lc = 0.2,
p_se = 0.3227348,
p_sc = 0.2,
n = 200,
fr_lo = 0.25,
fr_sh = 0.5,
phi_e = 0.5,
phi_c = 0.5,
pval = 0.45,
w = w[i],
stop_type = "zs")

create_ssr_fix_pval_weight_table(output_pval_w_zl = output_pval_w_zl,
output_pval_w_zb = output_pval_w_zb,
output_pval_w_zs = output_pval_w_zs,
w = w,
pval = 0.45,
save=FALSE)
```

---

create_ssr_obs_cp_table

Table Sample Size Reassessment Based on Observed CP, p-val Stopping rule

Description

Function creating a latex table showing operating characteristics of a trial with sample size reassessment based on conditional power. Futility stopping is based on the cut-off point of observed effect conditional power.

Usage

```
create_ssr_obs_cp_table(output_cp, phi_e, phi_c, save)
```

Arguments

output_cp	Output from simulation of a clinical trial with sample size reassessment and stopping based on c
phi_e	Correlation between S and L in the treatment group
phi_c	Correlation between S and L in the control group
save	If TRUE then the latex output is saved into a .txt file. Otherwise it is printed

Value

The function creates a table with operating characteristics: overall power, probability to stop for futility and average sample size with its standard deviation (brackets)

Examples

```
cl <-  makeCluster(1)
registerDoParallel(cl)

p_le <- c(0.2, 0.285, 0.3227348, 0.365)
p_se <- c(0.2, 0.285, 0.3227348, 0.365)

output_cp <- foreach(i = 1:length(p_le), .combine = rbind, .packages="binfutssr") %dopar%
ssr_obs_cp(nsim = 1000,
alpha = 0.025,
beta = 0.2,
p_le = p_le[i],
p_lc = 0.2,
p_se = p_se[i],
p_sc = 0.2,
n = 200,
fr_lo = 0.25,
fr_sh = 0.5,
phi_e = 0.5,
phi_c = 0.5,
c = 0.3)

create_ssr_obs_cp_table(output_cp = output_cp,
phi_e = 0.5,
phi_c = 0.5,
save = FALSE)
```

create_ssr_obs_pval_table
Table Sample Size Reassessment Based on Observed CP, p-val Stopping rule

Description

Function creating a latex table showing operating characteristics of a trial with sample size reassessment based on observed effect conditional power. Futility stopping is based on the p-value of one of the estimators.

Usage

```
create_ssr_obs_pval_table(output_pval_zl, output_pval_zb, output_pval_zs, pval,
  save)
```

Arguments

output_pval_zl	Output from simulation of a clinical trial with sample size reassessment and stopping based on p-value of p_l
output_pval_zb	Output from simulation of a clinical trial with sample size reassessment and stopping based on p-value of p_b
output_pval_zs	Output from simulation of a clinical trial with sample size reassessment and stopping based on p-value of p_s
pval	P-value which will act as the stopping rule benchmark. Takes values from 0 to 1
save	If TRUE then the latex output is saved into a .txt file. Otherwise it is printed

Value

The function creates a table with operating characteristics: overall power, probability to stop for futility and average sample size with its standard deviation (brackets)

Examples

```
cl <- makeCluster(1)
registerDoParallel(cl)

p_le <- c(0.2, 0.285, 0.3227348, 0.365)
p_se <- c(0.2, 0.285, 0.3227348, 0.365)

output_pval_zl <- foreach(i = 1:length(p_le), .combine = rbind, .packages="binfutssr") %dopar%
ssr_obs_z(nsim = 1000,
alpha = 0.025,
beta = 0.2,
p_le = p_le[i],
p_lc = 0.2,
p_se = p_se[i],
p_sc = 0.2,
n = 200,
fr_lo = 0.25,
fr_sh = 0.5,
```

```
phi_e = 0.5,
phi_c = 0.5,
pval = 0.45,
stop_type = "zl")

output_pval_zb <- foreach(i = 1:length(p_le), .combine = rbind, .packages="binfutssr") %dopar%
ssr_obs_z(nsim = 1000,
alpha = 0.025,
beta = 0.2,
p_le = p_le[i],
p_lc = 0.2,
p_se = p_se[i],
p_sc = 0.2,
n = 200,
fr_lo = 0.25,
fr_sh = 0.5,
phi_e = 0.5,
phi_c = 0.5,
pval = 0.45,
stop_type = "zb")

output_pval_zs <- foreach(i = 1:length(p_le), .combine = rbind, .packages="binfutssr") %dopar%
ssr_obs_z(nsim = 1000,
alpha = 0.025,
beta = 0.2,
p_le = p_le[i],
p_lc = 0.2,
p_se = p_se[i],
p_sc = 0.2,
n = 200,
fr_lo = 0.25,
fr_sh = 0.5,
phi_e = 0.5,
phi_c = 0.5,
pval = 0.45,
stop_type = "zs")


create_ssr_obs_pval_table(output_pval_zl,
output_pval_zb,
output_pval_zs,
pval = 0.45,
save= TRUE)
```

`create_ssr_obs_pval_weight_table`

Table Sample Size Reassessment Based on Observed CP, p-val Stopping rule, Equal Weight

---

Description

Function creating a latex table showing operating characteristics of a trial with sample size reassessment based on conditional power. The futility stopping is based the p-value of a given estimator after the first stage. The weight for the combination function for all the estimators is set to be equal.

If the trial is continued, e.g. p_l<pval, sample size reassessment is performed. The procedure is based on the observed effect conditional power.

Usage

```
create_ssr_obs_pval_weight_table(output_pval_w_zl, output_pval_w_zb,
  output_pval_w_zs, w, pval, save)
```

Arguments

`output_pval_w_zl`
: Output from simulation of a clinical trial with sample size reassessment and stopping based on p-value of p_l

`output_pval_w_zb`
: Output from simulation of a clinical trial with sample size reassessment and stopping based on p-value of p_b

`output_pval_w_zs`
: Output from simulation of a clinical trial with sample size reassessment and stopping based on p-value of p_s

`w`
: Weight to be set to the combination function

`pval`
: P-value which will act as the stopping rule benchmark. Takes values from 0 to 1

`save`
: If TRUE then the latex output is saved into a .txt file. Otherwise it is printed

Value

The function creates a table with operating characteristics: overall power, probability to stop for futility and average sample size with its standard deviation (brackets). Different set of weights are considered here, varying from 0 to 1.

Examples

```
cl <- makeCluster(1)
registerDoParallel(cl)

w <- seq(0, 1, 0.1)

output_pval_w_zl <- foreach(i = 1:length(w), .combine = rbind, .packages="binfutssr") %dopar%
ssr_obs_z_weight(nsim = 1000,
alpha = 0.025,
beta = 0.2,
p_le = 0.3227348,
p_lc = 0.2,
p_se = 0.3227348,
p_sc = 0.2,
n = 200,
fr_lo = 0.25,
fr_sh = 0.5,
phi_e = 0.5,
phi_c = 0.5,
pval = 0.45,
w = w[i],
stop_type = "zl")

output_pval_w_zb <- foreach(i = 1:length(w), .combine = rbind, .packages="binfutssr") %dopar%
```

```
ssr_obs_z_weight(nsim = 1000,
alpha = 0.025,
beta = 0.2,
p_le = 0.3227348,
p_lc = 0.2,
p_se = 0.3227348,
p_sc = 0.2,
n = 200,
fr_lo = 0.25,
fr_sh = 0.5,
phi_e = 0.5,
phi_c = 0.5,
pval = 0.45,
w = w[i],
stop_type = "zb")

output_pval_w_zs <- foreach(i = 1:length(w), .combine = rbind, .packages="binfutssr") %dopar%
ssr_obs_z_weight(nsim = 1000,
alpha = 0.025,
beta = 0.2,
p_le = 0.3227348,
p_lc = 0.2,
p_se = 0.3227348,
p_sc = 0.2,
n = 200,
fr_lo = 0.25,
fr_sh = 0.5,
phi_e = 0.5,
phi_c = 0.5,
pval = 0.45,
w = w[i],
stop_type = "zs")

create_ssr_obs_pval_weight_table(output_pval_w_zl = output_pval_w_zl,
output_pval_w_zb = output_pval_w_zb,
output_pval_w_zs = output_pval_w_zs,
w = w,
pval = 0.45,
save=FALSE)
```

`fixed_to_obs_plot` *Plot Showing Equivalence of Cut-Off Points*

Description

Obtain a plot showing equivalent cut-off points depending on the information fraction, type 1 error and power.

Usage

```
fixed_to_obs_plot(alpha, beta, t, c, filetype)
```

Arguments

alpha	Type 1 error level
beta	Type 2 error level such that 1-beta is the power
t	Information fraction for which the equivalent cut-off points are to be obtained. Can be a vector
c	The cut-off point to be considered for the conditional power, can be a single value or a vector of values between 0 and 1
filetype	Filetype which the plot should be saved to. O ptions are: c("pdf", "eps", "none"). If none is chosen the plot is printed

Value

The function creates a plot with equivalent cut-off points for conditional power.

Examples

```
fixed_to_obs_plot(alpha = 0.025,
                  beta = 0.2,
                  t = c(0.1, 0.25, 0.5, 0.75, 0.9),
                  c = seq(0, 1, 0.01),
                  filetype = "none")
```

---

ssr_fixed_cp *Sample Size Reassessment Based on Fixed CP, c Stopping rule*

Description

This function performs an adaptive trial with futility stopping and sample size reassessment The futility stopping is based the conditional power based on the fixed effect. If CP is below c then the trial is stopped for futility. The weight for the combination function is equal to information fractions. If the trial is continued, i.e. CP>c, sample size reassessment is performed. The procedure is based on the fixed effect conditional power

Usage

```
ssr_fixed_cp(nsim, alpha, beta, p_le, p_lc, p_se, p_sc, n, fr_lo, fr_sh, phi_e,
  phi_c, c)
```

Arguments

nsim	Number of simulated trials to be run
alpha	Type 1 error level
beta	Type 2 error level
p_le	Probability of success in the treatment group for the long-term endpoint L
p_lc	Probability of success in the control group for the long-term endpoint L
p_se	Probability of success in the trearment group for the short-term endpoint S
p_sc	Probability of success in the control group for the short-term endpoint S
n	Sample size per treatment group

fr_lo	Amount of information available at interim for the long-term endpoint, value between 0 and 1
fr_sh	Amount of information available at interim for the short-term endpoint, value between 0 and 1
phi_e	Correlation between S and L in the treatment group
phi_c	Correlation between S and L in the control group
c	Cut-off point for the conditional power stopping rule; if CP<c the trial is stopped for futility

Value

The function returns a data frame with operating characteristics of a simulated trial including the overall power/type I error, power if the trial is continued, expected sample size and its standard error

Examples

```
ssr_fixed_cp(nsim = 1000,
alpha = 0.025,
beta = 0.2,
p_le = 0.3227348,
p_lc = 0.2,
p_se = 0.3227348,
p_sc = 0.2,
n = 200,
fr_lo = 0.25,
fr_sh = 0.5,
phi_e = 0.5,
phi_c = 0.5,
c = 0.3)
```

---

ssr_fixed_z *Sample Size Reassessment Fixed Effect, p-val Stopping*

Description

This function performs an adaptive trial with futility stopping and sample size reassessment The futility stopping is based the p-value of a given estimator after the first stage. Stopping is based on the p-value of the z-statistic of of a given estimator. Note that the Z-statistics of estimators using short-term information are not the ones is used at the end of the trial for the combination test. The weight for the combination function is equal to information fractions. If the trial is continued, e.g. p_s<pval, sample size reassessment is performed.. The procedure is based on the fixed effect conditional power

Usage

```
ssr_fixed_z(nsim, alpha, beta, p_le, p_lc, p_se, p_sc, n, fr_lo, fr_sh, phi_e,
  phi_c, pval, stop_type)
```

Arguments

nsim	Number of simulated trials to be run
alpha	Type 1 error level
beta	Type 2 error level
p_le	Probability of success in the treatment group for the long-term endpoint L
p_lc	Probability of success in the control group for the long-term endpoint L
p_se	Probability of success in the trearment group for the short-term endpoint S
p_sc	Probability of success in the control group for the short-term endpoint S
n	Sample size per treatment group
fr_lo	Amount of information available at interim for the long-term endpoint, value between 0 and 1
fr_sh	Amount of information available at interim for the short-term endpoint, value between 0 and 1
phi_e	Correlation between S and L in the treatment group
phi_c	Correlation between S and L in the control group
pval	P-value which will act as the stopping rule benchmark. Takes values from 0 to 1
stop_type	Type of the futility stopping approach. It can be based on the value of z_l, z_s or z_b. Options are c("zl", "zs", "zb")

Value

The function returns a data frame with operating characteristics of a simulated trial including the overall power/type I error, power if the trial is continued, expected sample size and its standard error

Examples

```
ssr_fixed_z(nsim=1000,
alpha = 0.025,
beta = 0.2,
p_le = 0.3227348,
p_lc = 0.2,
p_se = 0.3227348,
p_sc = 0.2,
n = 200,
fr_lo = 0.25,
fr_sh = 0.5,
phi_e = 0.5,
phi_c = 0.5,
pval = 0.45,
stop_type = "zs")
```

ssr_fixed_z_weight — *Sample Size Reassessment Fixed Effect, p-val Stopping, Equal Weight*

Description

This function performs an adaptive trial with futility stopping and sample size reassessment The futility stopping is based the p-value of a given estimator after the first stage. Stopping is based on the p-value of the z-statistic of of a given estimator. Note that the Z-statistics of estimators using short-term information are not the ones is used at the end of the trial for the combination test.n, and is not the one that is used at the end of the trial for the combination test. The weight for the combination function for all the estimators is set to be equal. If the trial is continued, e.g. p_s<pval, sample size reassessment is performed. The procedure is based on the fixed effect conditional power

Usage

```
ssr_fixed_z_weight(nsim, alpha, beta, p_le, p_lc, p_se, p_sc, n, fr_lo, fr_sh,
  phi_e, phi_c, pval, w, stop_type)
```

Arguments

nsim	Number of simulated trials to be run
alpha	Type 1 error level
beta	Type 2 error level
p_le	Probability of success in the treatment group for the long-term endpoint L
p_lc	Probability of success in the control group for the long-term endpoint L
p_se	Probability of success in the trearment group for the short-term endpoint S
p_sc	Probability of success in the control group for the short-term endpoint S
n	Sample size per treatment group
fr_lo	Amount of information available at interim for the long-term endpoint, value between 0 and 1
fr_sh	Amount of information available at interim for the short-term endpoint, value between 0 and 1
phi_e	Correlation between S and L in the treatment group
phi_c	Correlation between S and L in the control group
pval	P-value which will act as the stopping rule benchmark. Takes values from 0 to 1
w	Weight to be set to the combination function
stop_type	Type of the futility stopping approach. It can be based on the value of z_l, z_s or z_b. Options are c("zl", "zs", "zb")

Value

The function returns a data frame with operating characteristics of a simulated trial including the overall power/type I error, probability to reject the null given the trial is continued, average sample size and its standard deviation

Examples

```
ssr_fixed_z_weight(nsim = 1000,
alpha = 0.025,
beta = 0.2,
p_le = 0.3227348,
p_lc = 0.2,
p_se = 0.3227348,
p_sc = 0.2,
n = 200,
fr_lo = 0.25,
fr_sh = 0.5,
phi_e = 0.5,
phi_c = 0.5,
pval = 0.45,
w = 0.5,
stop_type = "zl")
```

---

ssr_obs_cp *Sample Size Reassessment Observed Effect, c Stopping*

Description

This function performs an adaptive trial with futility stopping and sample size reassessment The futility stopping is based the conditional power based on the observed effect. If CP is below c then the trial is stopped for futility. The weight for the combination function is equal to information fractions. If the trial is continued, i.e. CP>c, sample size reassessment is performed. The procedure is based on the observed effect conditional power

Usage

```
ssr_obs_cp(nsim, alpha, beta, p_le, p_lc, p_se, p_sc, n, fr_lo, fr_sh, phi_e,
  phi_c, c)
```

Arguments

nsim	Number of simulated trials to be run
alpha	Type 1 error level
beta	Type 2 error level
p_le	Probability of success in the treatment group for the long-term endpoint L
p_lc	Probability of success in the control group for the long-term endpoint L
p_se	Probability of success in the trearment group for the short-term endpoint S
p_sc	Probability of success in the control group for the short-term endpoint S
n	Sample size per treatment group
fr_lo	Amount of information available at interim for the long-term endpoint, value between 0 and 1
fr_sh	Amount of information available at interim for the short-term endpoint, value between 0 and 1
phi_e	Correlation between S and L in the treatment group
phi_c	Correlation between S and L in the control group
c	Cut-off point for the conditional power stopping rule; if CP<c the trial is stopped for futility

Value

The function returns a data frame with operating characteristics of a simulated trial including the overall power/type I error, power if the trial is continued, expected sample size and its standard error

Examples

```
ssr_obs_cp(nsim = 1000,
alpha = 0.025,
beta = 0.2,
p_le = 0.3227348,
p_lc = 0.2,
p_se = 0.3227348,
p_sc = 0.2,
n = 200,
fr_lo = 0.25,
fr_sh = 0.5,
phi_e = 0.5,
phi_c = 0.5,
c = 0.3)
```

`ssr_obs_z` *Sample Size Reassessment Observed Effect, p-val Stopping*

Description

This function performs an adaptive trial with futility stopping and sample size reassessment Stopping is based on the p-value of the z-statistic of of a given estimator. Note that the Z-statistics of estimators using short-term information are not the ones is used at the end of the trial for the combination test.n, and is not the one that is used at the end of the trial for the combination test. The weight for the combination function is equal to information fractions. If the trial is continued, e.g. p_s<pval, sample size reassessment is performed. The procedure is based on the observed effect conditional power

Usage

```
ssr_obs_z(nsim, alpha, beta, p_le, p_lc, p_se, p_sc, n, fr_lo, fr_sh, phi_e,
  phi_c, pval, stop_type)
```

Arguments

`nsim`	Number of simulated trials to be run
`alpha`	Type 1 error level
`beta`	Type 2 error level
`p_le`	Probability of success in the treatment group for the long-term endpoint L
`p_lc`	Probability of success in the control group for the long-term endpoint L
`p_se`	Probability of success in the trearment group for the short-term endpoint S
`p_sc`	Probability of success in the control group for the short-term endpoint S
`n`	Sample size per treatment group
`fr_lo`	Amount of information available at interim for the long-term endpoint, value between 0 and 1

fr_sh	Amount of information available at interim for the short-term endpoint, value between 0 and 1
phi_e	Correlation between S and L in the treatment group
phi_c	Correlation between S and L in the control group
pval	P-value which will act as the stopping rule benchmark. Takes values from 0 to 1
stop_type	Type of the futility stopping approach. It can be based on the value of z_l, z_s or z_b. Options are c("zl", "zs", "zb")

Value

The function returns a data frame with operating characteristics of a simulated trial including the overall power/type I error, power if the trial is continued, expected sample size and its standard error

Examples

```
ssr_obs_z(nsim=1000,
alpha = 0.025,
beta = 0.2,
p_le = 0.3227348,
p_lc = 0.2,
p_se = 0.3227348,
p_sc = 0.2,
n = 200,
fr_lo = 0.25,
fr_sh = 0.5,
phi_e = 0.5,
phi_c = 0.5,
pval = 0.45,
stop_type = "zl")
```

---

ssr_obs_z_weight — *Sample Size Reassessment Observed Effect, p-val Stopping, Equal Weight*

Description

This function performs an adaptive trial with futility stopping and sample size reassessment The futility stopping is based the p-value of a given estimator after the first stage. Stopping is based on the p-value of the z-statistic of of a given estimator. Note that the Z-statistics of estimators using short-term information are not the ones is used at the end of the trial for the combination test.n, and is not the one that is used at the end of the trial for the combination test. The weight for the combination function for all the estimators is set to be equal. If the trial is continued, e.g. p_s<pval, sample size reassessment is performed. The procedure is based on the observed effect conditional power

Usage

```
ssr_obs_z_weight(nsim, alpha, beta, p_le, p_lc, p_se, p_sc, n, fr_lo, fr_sh,
  phi_e, phi_c, pval, w, stop_type)
```

Arguments

nsim	Number of simulated trials to be run
alpha	Type 1 error level
beta	Type 2 error level
p_le	Probability of success in the treatment group for the long-term endpoint L
p_lc	Probability of success in the control group for the long-term endpoint L
p_se	Probability of success in the trearment group for the short-term endpoint S
p_sc	Probability of success in the control group for the short-term endpoint S
n	Sample size per treatment group
fr_lo	Amount of information available at interim for the long-term endpoint, value between 0 and 1
fr_sh	Amount of information available at interim for the short-term endpoint, value between 0 and 1
phi_e	Correlation between S and L in the treatment group
phi_c	Correlation between S and L in the control group
pval	P-value which will act as the stopping rule benchmark. Takes values from 0 to 1
w	Weight to be set to the combination function
stop_type	Type of the futility stopping approach. It can be based on the value of z_l, z_s or z_b. Options are c("zl", "zs", "zb")

Value

The function returns a data frame with operating characteristics of a simulated trial including the overall power/type I error, probability to reject the null given the trial is continued, average sample size and its standard deviation

Examples

```
ssr_obs_z_weight(nsim = 1000,
alpha = 0.025,
beta = 0.2,
p_le = 0.3227348,
p_lc = 0.2,
p_se = 0.3227348,
p_sc = 0.2,
n = 200,
fr_lo = 0.25,
fr_sh = 0.5,
phi_e = 0.5,
phi_c = 0.5,
pval = 0.45,
w = 0.5,
stop_type = "zl")
```

Index

cp, 2
create_corr_plots, 3
create_corr_plots_p_se, 12
create_corr_plots_p_se_low, 14
create_corr_plots_prob_cont_rej_p_se, 4
create_corr_plots_prob_corr_dec_p_se, 7
create_corr_plots_prob_stop_not_p_se, 9
create_fs_plots, 17
create_fs_plots_nested, 18
create_power_plots, 20
create_power_plots_nested, 22
create_prob_cont_rej_plots, 24
create_prob_cont_rej_plots_nested, 26
create_prob_corr_dec_plots, 28
create_prob_corr_dec_plots_nested, 29
create_prob_stop_not_rej_plots, 31
create_prob_stop_not_rej_plots_nested, 33
create_ssr_fix_cp_table, 35
create_ssr_fix_pval_table, 36
create_ssr_fix_pval_weight_table, 38
create_ssr_obs_cp_table, 40
create_ssr_obs_pval_table, 41
create_ssr_obs_pval_weight_table, 43

fixed_to_obs_plot, 45

ssr_fixed_cp, 46
ssr_fixed_z, 47
ssr_fixed_z_weight, 48
ssr_obs_cp, 49
ssr_obs_z, 50
ssr_obs_z_weight, 52
