# Supplementary material for: Interim analysis incorporating short‐ and long‐term binary endpoints
Source: Biom J. 2019 Jan 29;61(3):665–87. doi: 10.1002/bimj.201700281 (PMC6590444; doi:10.1002/bimj.201700281)
Supplement: Supplementary file 1 — Supporting Information [file BIMJ-61-665-s001.zip › Reproducibility_Julia_Niewczas/binfutssr_0.1.0.tar.gz!/binfutssr/FSPhi065shp202py202.pdf]

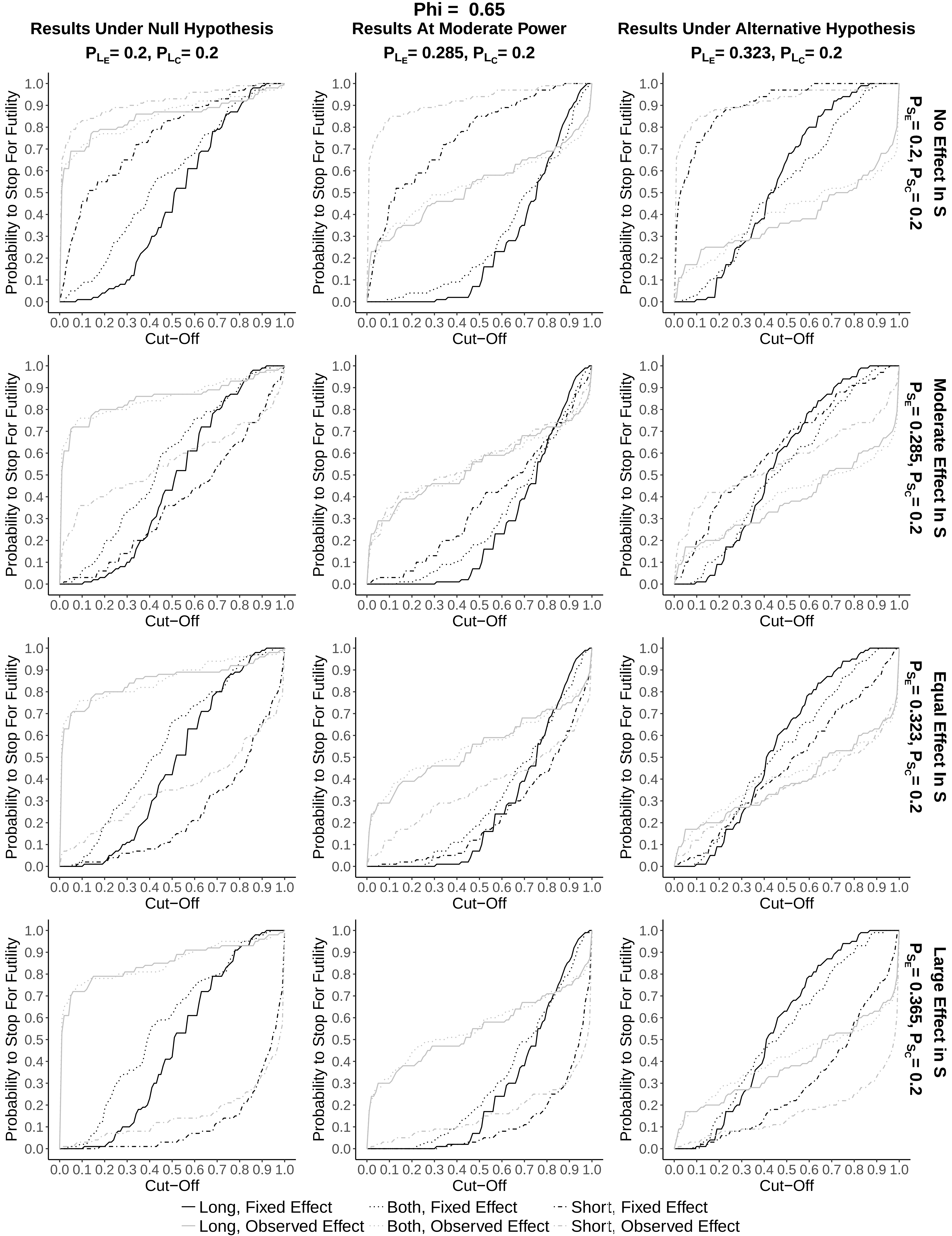
Phi = 0.65
Results Under Null Hypothesis
P_{L_E}= 0.2, P_{L_C}= 0.2
Results At Moderate Power
P_{L_E}= 0.285, P_{L_C}= 0.2
Results Under Alternative Hypothesis
P_{L_E}= 0.323, P_{L_C}= 0.2
No Effect In S
P_{S_E}= 0.2, P_{S_C}= 0.2
Moderate Effect In S
P_{S_E}= 0.285, P_{S_C}= 0.2
Equal Effect In S
P_{S_E}= 0.323, P_{S_C}= 0.2
Large Effect in S
P_{S_E}= 0.365, P_{S_C}= 0.2
Probability to Stop For Futility
Cut−Off
0.0 0.1 0.2 0.3 0.4 0.5 0.6 0.7 0.8 0.9 1.0
Long, Fixed Effect
Long, Observed Effect
Both, Fixed Effect
Both, Observed Effect
Short, Fixed Effect
Short, Observed Effect
