# Supplementary material for: Interim analysis incorporating short‐ and long‐term binary endpoints
Source: Biom J. 2019 Jan 29;61(3):665–87. doi: 10.1002/bimj.201700281 (PMC6590444; doi:10.1002/bimj.201700281)
Supplement: Supplementary file 1 — Supporting Information [file BIMJ-61-665-s001.zip › Reproducibility_Julia_Niewczas/binfutssr_0.1.0.tar.gz!/binfutssr/FixedCCorrPSEVariedlo025plc02psc02ple03227348phie0to09phic0to09.pdf]

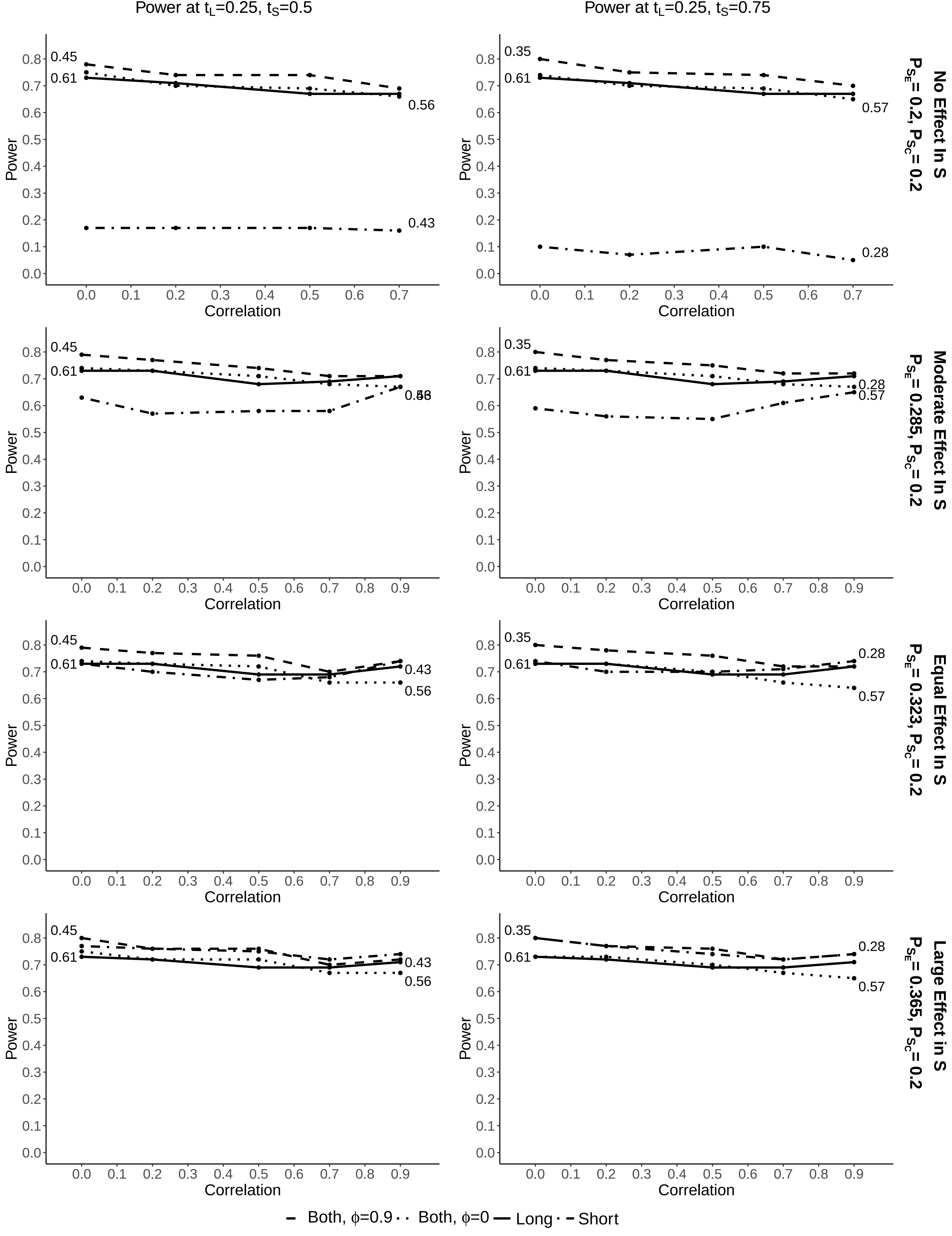

Power at tL=0.25, tS=0.5
Power at tL=0.25, tS=0.75
No Effect In S PSE= 0.2, PSC= 0.2
Moderate Effect In S PSE= 0.285, PSC= 0.2
Equal Effect In S PSE= 0.323, PSC= 0.2
Large Effect in S PSE= 0.365, PSC= 0.2
Power
Correlation
0.45
0.61
0.56
0.43
0.35
0.57
0.28
Both, ϕ=0.9
Both, ϕ=0
Long
Short
