# Supplementary material for: Interim analysis incorporating short‐ and long‐term binary endpoints
Source: Biom J. 2019 Jan 29;61(3):665–87. doi: 10.1002/bimj.201700281 (PMC6590444; doi:10.1002/bimj.201700281)
Supplement: Supplementary file 1 — Supporting Information [file BIMJ-61-665-s001.zip › Reproducibility_Julia_Niewczas/binfutssr_0.1.0.tar.gz!/binfutssr/FixedCCorrlo025plc02psc02ple03227348phie0to07phic0to07.pdf]

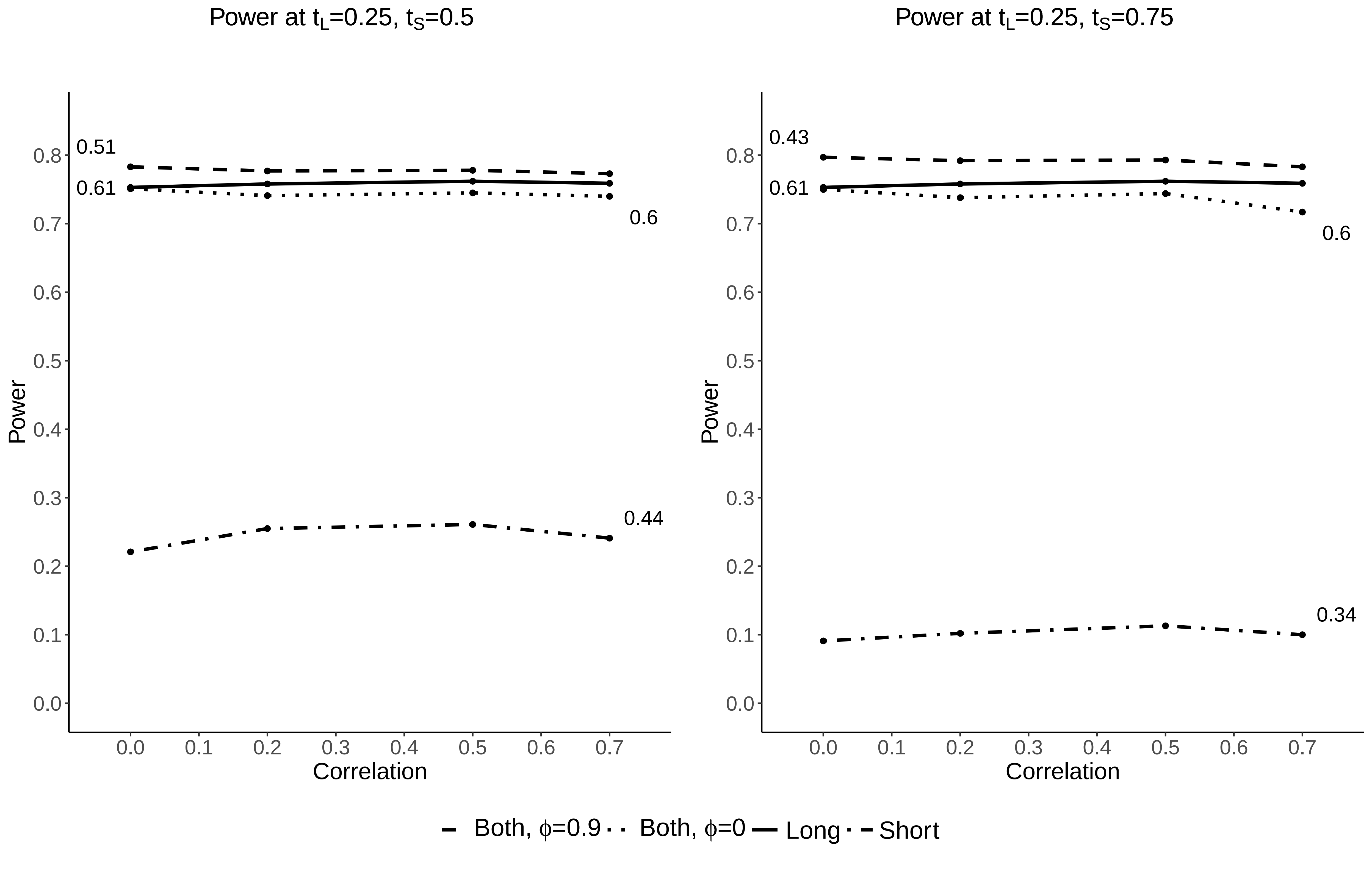

Power at t_L=0.25, t_S=0.5
Power at t_L=0.25, t_S=0.75
Power
Correlation
0.0
0.1
0.2
0.3
0.4
0.5
0.6
0.7
0.8
0.51
0.61
0.6
0.44
0.43
0.61
0.6
0.34
Both, ϕ=0.9
Both, ϕ=0
Long
Short
