# Supplementary material for: Interim analysis incorporating short‐ and long‐term binary endpoints
Source: Biom J. 2019 Jan 29;61(3):665–87. doi: 10.1002/bimj.201700281 (PMC6590444; doi:10.1002/bimj.201700281)
Supplement: Supplementary file 1 — Supporting Information [file BIMJ-61-665-s001.zip › Reproducibility_Julia_Niewczas/binfutssr_0.1.0.tar.gz!/binfutssr/NestedNotRejStopPhi0681385to0490433shp202py2035.pdf]

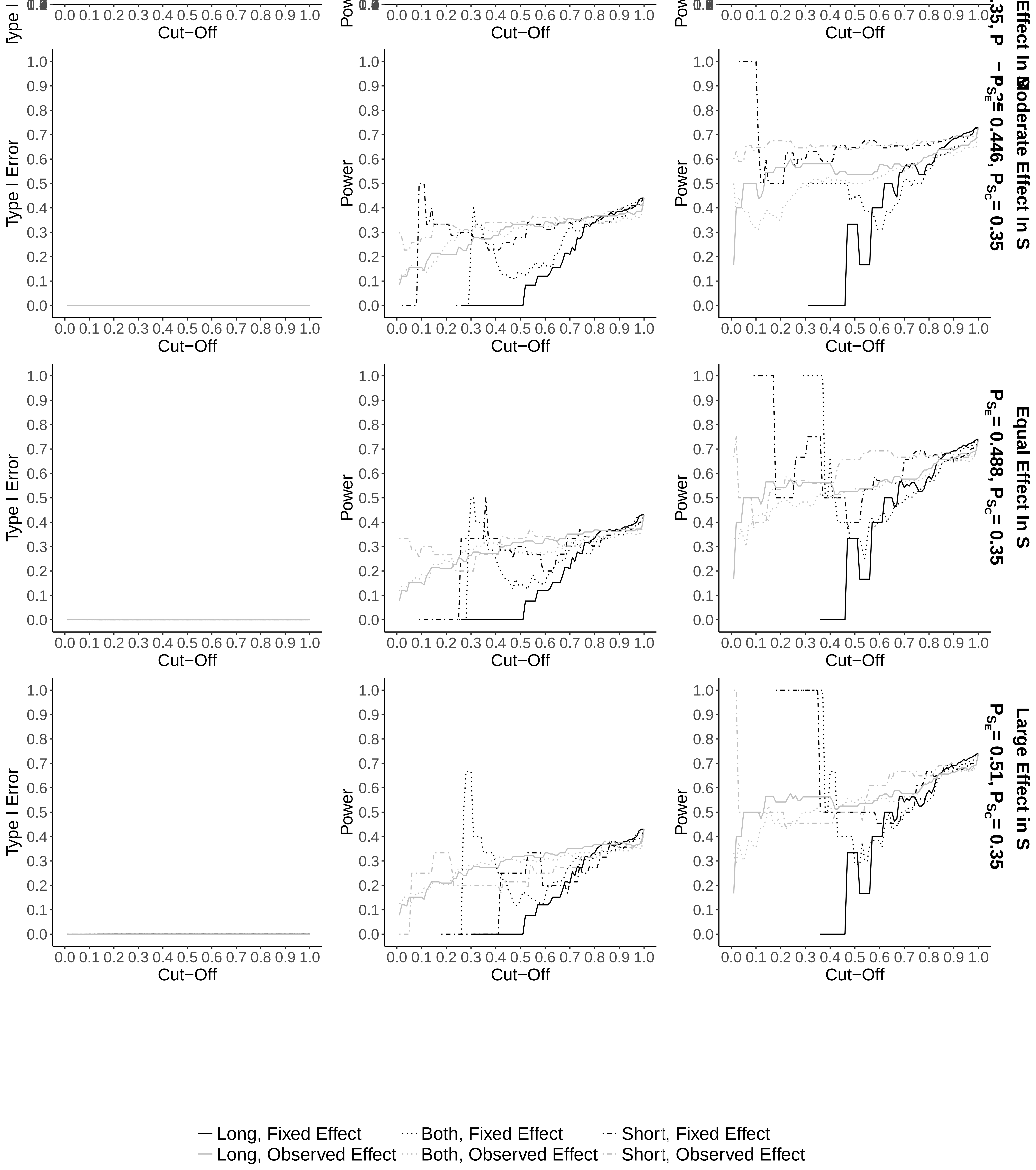

Type I Error
Power
Cut−Off
Moderate Effect In S
P_{S_E} = 0.446, P_{S_C} = 0.35
Equal Effect In S
P_{S_E} = 0.488, P_{S_C} = 0.35
Large Effect in S
P_{S_E} = 0.51, P_{S_C} = 0.35
Long, Fixed Effect
Both, Fixed Effect
Short, Fixed Effect
Long, Observed Effect
Both, Observed Effect
Short, Observed Effect
