# Supplementary material for: Interim analysis incorporating short‐ and long‐term binary endpoints
Source: Biom J. 2019 Jan 29;61(3):665–87. doi: 10.1002/bimj.201700281 (PMC6590444; doi:10.1002/bimj.201700281)
Supplement: Supplementary file 1 — Supporting Information [file BIMJ-61-665-s001.zip › Reproducibility_Julia_Niewczas/binfutssr_0.1.0.tar.gz!/binfutssr/ProbRejContFixedCCorrPSEVariedlo025plc02psc02ple03227348phie0to09phic0to09.pdf]

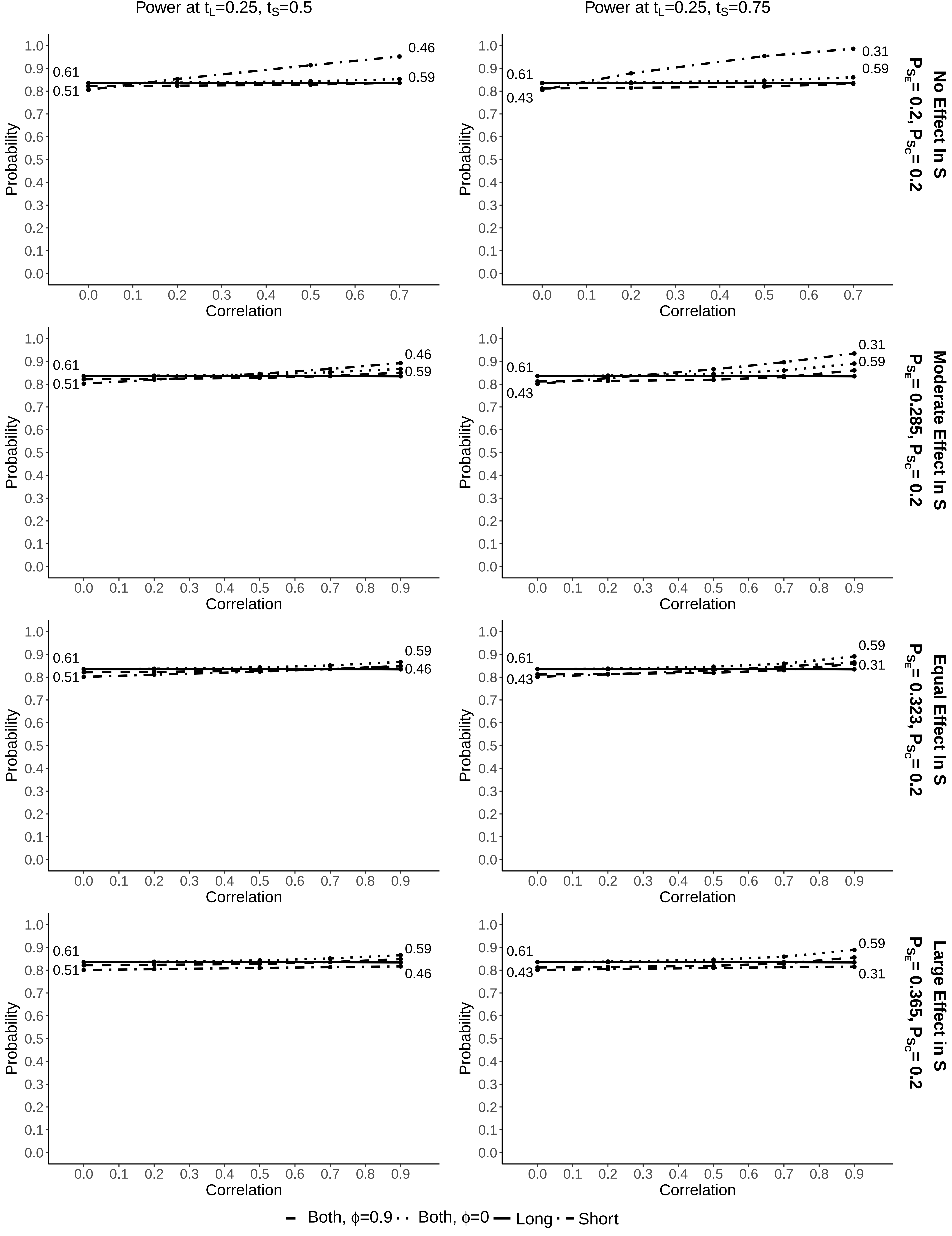

Power at t_L=0.25, t_S=0.5
Power at t_L=0.25, t_S=0.75
No Effect In S P_{S_E}= 0.2, P_{S_C}= 0.2
Moderate Effect In S P_{S_E}= 0.285, P_{S_C}= 0.2
Equal Effect In S P_{S_E}= 0.323, P_{S_C}= 0.2
Large Effect in S P_{S_E}= 0.365, P_{S_C}= 0.2
Probability
Correlation
0.61
0.51
0.46
0.59
0.43
0.31
Both, ϕ=0.9
Both, ϕ=0
Long
Short
