# Supplementary material for: Interim analysis incorporating short‐ and long‐term binary endpoints
Source: Biom J. 2019 Jan 29;61(3):665–87. doi: 10.1002/bimj.201700281 (PMC6590444; doi:10.1002/bimj.201700281)
Supplement: Supplementary file 1 — Supporting Information [file BIMJ-61-665-s001.zip › Reproducibility_Julia_Niewczas/binfutssr_0.1.0.tar.gz!/binfutssr/interim.html]

Output: Interim analysis incorporating short- and long-term binary endpoints


# Output: Interim analysis incorporating short- and long-term binary endpoints

#### *Julia Niewczas*

#### *11/23/2018*

## R Markdown

This is an R Markdown document. Markdown is a simple formatting syntax for authoring HTML, PDF, and MS Word documents. For more details on using R Markdown see http://rmarkdown.rstudio.com.

When you click the **Knit** button a document will be generated that includes both content as well as the output of any embedded R code chunks within the document. You can embed an R code chunk like this:

```
summary(cars)
```

```
##      speed           dist       
##  Min.   : 4.0   Min.   :  2.00  
##  1st Qu.:12.0   1st Qu.: 26.00  
##  Median :15.0   Median : 36.00  
##  Mean   :15.4   Mean   : 42.98  
##  3rd Qu.:19.0   3rd Qu.: 56.00  
##  Max.   :25.0   Max.   :120.00
```

## Including Plots

You can also embed plots, for example:

Note that the `echo = FALSE` parameter was added to the code chunk to prevent printing of the R code that generated the plot.
