# Supplementary material for: Interim analysis incorporating short‐ and long‐term binary endpoints
Source: Biom J. 2019 Jan 29;61(3):665–87. doi: 10.1002/bimj.201700281 (PMC6590444; doi:10.1002/bimj.201700281)
Supplement: Supplementary file 1 — Supporting Information [file BIMJ-61-665-s001.zip › Reproducibility_Julia_Niewczas/binfutssr_0.1.0.tar.gz!/binfutssr/normCorrlo025plc02psc02pse03227348phie0to09phic0to09.pdf]

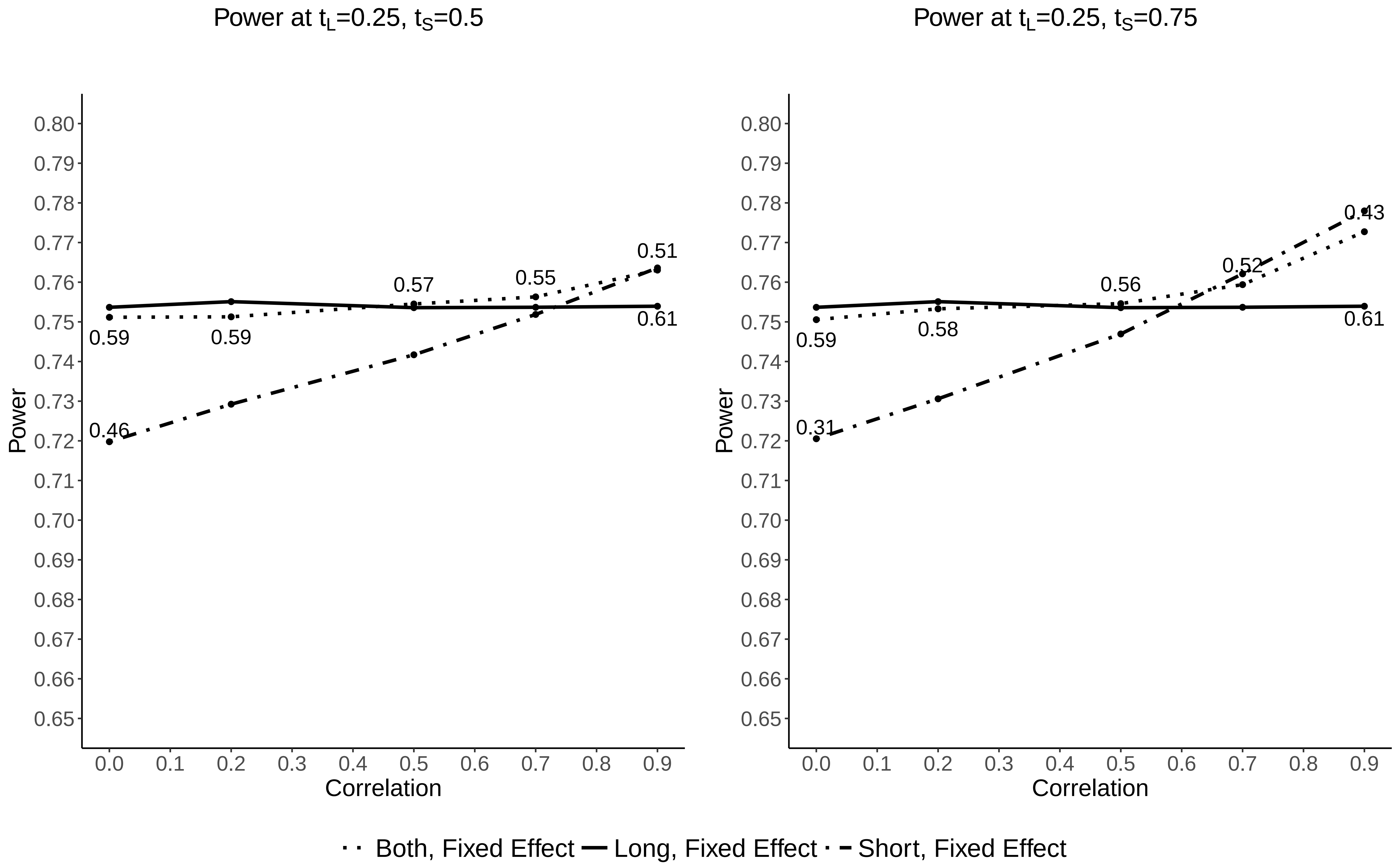

Power at t_L=0.25, t_S=0.5
Power at t_L=0.25, t_S=0.75
Power
Correlation
0.80
0.79
0.78
0.77
0.76
0.75
0.74
0.73
0.72
0.71
0.70
0.69
0.68
0.67
0.66
0.65
0.0
0.1
0.2
0.3
0.4
0.5
0.6
0.7
0.8
0.9
0.51
0.57
0.55
0.59
0.59
0.61
0.46
0.43
0.52
0.56
0.59
0.58
0.61
0.31
Both, Fixed Effect
Long, Fixed Effect
Short, Fixed Effect
