# Supplementary material for: Interim analysis incorporating short‐ and long‐term binary endpoints
Source: Biom J. 2019 Jan 29;61(3):665–87. doi: 10.1002/bimj.201700281 (PMC6590444; doi:10.1002/bimj.201700281)
Supplement: Supplementary file 1 — Supporting Information [file BIMJ-61-665-s001.zip › Reproducibility_Julia_Niewczas/binfutssr_0.1.0.tar.gz!/binfutssr/untitiled9.pdf]

Supplementary Materials

Interim analysis incorporating short- and long-term binary endpoints

Julia Niewczas and Franz König*

Center for Medical Statistics, Informatics, and Intelligent Systems, Medical University of Vienna, Spitalgasse 23, 1090 Vienna, Austria

Cornelia Ursula Kunz

Department of Mathematics and Statistics, Lancaster University Lancaster LA1 4YF, UK

*franz.koenig@meduniwien.ac.at; Corresponding author

1 Mathematical Formulae

We compare three ways of estimating the response rate at the interim analysis:

- use of long-term data only, $\hat{P}^{(1)}_{\mathbf{L}}$,
- use of short-term data only, $\hat{P}^{(1)}_{\mathbf{S}}$,
- or combination of both, $\hat{P}^{(1)}_{\mathbf{B}}$.

There are n_{L_i} patients in each treatment group at interim for the primary endpoint, and $n_{S_i}(\geq n_{L_i})$ patients in each treatment group for the secondary endpoint. @WatsonCrick1953Adjusted significance levels for subgroup analyses in clinical trials

1.1 Information Fraction of $\hat{P}^{(1)}_{\mathbf{L}}$

The information fraction of $\hat{P}^{(1)}_{\mathbf{L}}$ is given by:

$$
\begin{aligned}
t_L &= \frac{\frac{\bar{P}^{(1)}_L(1-\bar{P}^{(1)}_L)}{N_C} + \frac{\bar{P}^{(1)}_L(1-\bar{P}^{(1)}_L)}{N_E}}{\frac{\bar{P}^{(1)}_L(1-\bar{P}^{(1)}_L)}{n_{L_E}} + \frac{\bar{P}^{(1)}_L(1-\bar{P}^{(1)}_L)}{n_{L_C}}} \\
&= \frac{\bar{P}^{(1)}_L(1-\bar{P}^{(1)}_L)\left(\frac{1}{N_C}+\frac{1}{N_E}\right)}{\bar{P}^{(1)}_L(1-\bar{P}^{(1)}_L)\left(\frac{1}{n_{L_C}}+\frac{1}{n_{L_E}}\right)} \\
&= \frac{\frac{1}{N_C}+\frac{1}{N_E}}{\frac{1}{n_{L_C}}+\frac{1}{n_{L_E}}}
\end{aligned}
$$

where $\bar{P}^{(1)}_L = \frac{\hat{P}^{(1)}_{L_E}+\hat{P}^{(1)}_{L_C}}{2}$ (under the assumption of null hypothesis). This simplifies to n_L/N when design is balanced, that is when $n_{L_E} = n_{L_C}$ and $N_E = N_C$.

1.2 Z-Statistic and t_S for $\hat{P}^{(1)}_{S_i}$

The Z-statistic and information fraction t_S for $\hat{P}^{(1)}_{S_i}$ can be obtained:

$$
Z^{(1)}_S = \frac{\hat{P}^{(1)}_{S_E} - \hat{P}^{(1)}_{S_C}}{\sqrt{\bar{P}^{(1)}_S(1-\bar{P}^{(1)}_S)\left(\frac{1}{n_{S_E}}+\frac{1}{n_{S_C}}\right)}},
$$

where $\bar{P}^{(1)}_S = \frac{\hat{P}^{(1)}_{S_C}+\hat{P}^{(1)}_{S_E}}{2}$. The information fraction is equal to

$$
t_S = \frac{1/N_E + 1/N_C}{1/n_{S_E} + 1/n_{S_C}}.
$$

Again, if $n_{S_E} = n_{S_C}$ and $N_E = N_C$, the formula simplifies to n_S/N.

1.3 Estimator Incorporating Both S and L

Estimator that combines information from both S and L is derived from three-binomial distributions (Marschner and Becker, 2001) of $P^{(1)}_{S_i}$, $P^{(1)}_{L_i}$ and $P^{(1)}_{SL_i} = Pr(L_i = 1|S_i = 1)$. Consider patients for whom L has been observed and define n_{SL_i} to be the number of patients for whom $S_i = 1$ and similarly s_{SL_i} to be the number of patients for whom $S_i = 0$. Then, define m_{SL_i} and r_{SL_i} to be the number of subjects for whom $(L_i = 1, S_i = 1)$ and $(L_i = 1, S_i = 0)$ respectively. As

$Pr(L_i=1|S_i=0)=\frac{P^{(1)}_{L_i}-P^{(1)}_{SL_i}P^{(1)}_{S_i}}{1-P^{(1)}_{S_i}}$, the likelihood function is given by Marschner and Becker (2001):

$$L(P^{(1)}_{L_i},P^{(1)}_{S_i},P^{(1)}_{SL_i}|\mathbf{L_i},\mathbf{S_i}) =(P^{(1)}_{S_i})^{m_{S_i}}(1-P^{(1)}_{S_i})^{n_{S_i}-m_{S_i}}\left(\frac{P^{(1)}_{L_i}-P^{(1)}_{S_i}P^{(1)}_{SL_i}}{1-P^{(1)}_{S_i}}\right)^{r_{SL_i}}$$
$$\times\left(\frac{1-P^{(1)}_{L_i}-(1-P^{(1)}_{SL_i})P^{(1)}_{S_i}}{1-P^{(1)}_{S_i}}\right)^{s_{SL_i}-r_{SL_i}}(P^{(1)}_{SL_i})^{m_{SL_i}}(1-P^{(1)}_{SL_i})^{n_{SL_i}-m_{SL_i}},$$

with corresponding observations of $(m_{S_i},r_{SL_i},m_{SL_i})$. In case of $\hat{P}^{(1)}_{\mathbf{B}}$, the variance is obtained from the asymptotic distribution of the likelihood function (see Marschner and Becker (2001)) and it can be simplified to the following form:

$$\mathrm{Var}(\hat{P}^{(1)}_{B_i})=\frac{1}{n_{L_i}}\hat{P}^{(1)}_{B_i}\left(1-\hat{P}^{(1)}_{B_i}\right)\left(1-\hat{\phi}_i{}^2\times\left(1-\frac{n_{L_i}}{n_{S_i}}\right)\right),$$

where $\hat{\phi}_i$ is the estimate of the correlation (Phi coefficient) between S and L (defined by Cramér (1946)):

$$\hat{\phi}_i=\frac{\hat{P}^{(1)}_{SL_i}\hat{P}^{(1)}_{S_i}-\hat{P}^{(1)}_{B_i}\hat{P}^{(1)}_{S_i}}{\sqrt{\hat{P}^{(1)}_{B_i}(1-\hat{P}^{(1)}_{B_i})\hat{P}^{(1)}_{S_i}(1-\hat{P}^{(1)}_{S_i})}}.$$

1.4 Information Fraction For $\hat{P}^{(1)}_{\mathbf{B}}$

Using methodology of Proschan et al. (2006) and Stallard (2010) for balanced design:

$$\begin{aligned}t_B&=\frac{\frac{\bar{P}^{(1)}_B(1-\bar{P}^{(1)}_B)}{N_E}+\frac{\bar{P}^{(1)}_B(1-\bar{P}^{(1)}_B)}{N_C}}{\frac{\bar{P}^{(1)}_B(1-\bar{P}^{(1)}_B)}{n_{L_E}}\left(1-\hat{\phi}^2_E\left(1-\frac{n_{L_E}}{n_{S_E}}\right)\right)+\frac{\bar{P}^{(1)}_B(1-\bar{P}^{(1)}_B)}{n_{L_C}}\left(1-\hat{\phi}^2_C\left(1-\frac{n_{L_C}}{n_{S_C}}\right)\right)}\\&=\frac{\bar{P}^{(1)}_B(1-\bar{P}^{(1)}_B)\left(\frac{1}{N_C}+\frac{1}{N_E}\right)}{\bar{P}^{(1)}_B(1-\bar{P}^{(1)}_B)\left(\frac{1-\hat{\phi}^2_E\left(1-\frac{n_{L_E}}{n_{S_E}}\right)}{n_{L_E}}+\frac{1-\hat{\phi}^2_C\left(1-\frac{n_{L_C}}{n_{S_C}}\right)}{n_{L_C}}\right)}\\&=\frac{\frac{1}{N_C}+\frac{1}{N_E}}{\frac{1-\hat{\phi}^2_E\left(1-\frac{n_{L_E}}{n_{S_E}}\right)}{n_{L_E}}+\frac{1-\hat{\phi}^2_C\left(1-\frac{n_{L_C}}{n_{S_C}}\right)}{n_{L_C}}},\end{aligned}$$

where $\bar{P}^{(1)}_B=\frac{\hat{P}^{(1)}_{B_E}+\hat{P}^{(1)}_{B_C}}{2}$. If we assume that $N_E=N_C=N$, $n_{L_E}=n_{L_C}=n_L$ and $n_{S_E}=n_{S_C}=n_S$, the above simplifies to

$$\begin{aligned}t_B&=\frac{\frac{1}{N}+\frac{1}{N}}{\frac{1-\hat{\phi}^2_E\left(1-\frac{n_L}{n_S}\right)}{n_L}+\frac{1-\hat{\phi}^2_C\left(1-\frac{n_L}{n_S}\right)}{n_L}}\\&=\frac{\frac{2}{N}}{\frac{1}{n_L}\left(1-\hat{\phi}^2_E\left(1-\frac{n_L}{n_S}\right)+1-\hat{\phi}^2_C\left(1-\frac{n_L}{n_S}\right)\right)}\\&=\frac{2n_L}{N\left(2-\left(1-\frac{n_L}{n_S}\right)(\hat{\phi}^2_E+\hat{\phi}^2_C)\right)}.\end{aligned}$$

1.5 Conditional Power

The conditional power for is obtained as follows. We know that the test statistic at the end of the trial can be written as:

$$Z_i=\sqrt{t_i}Z^{(1)}_i+\sqrt{1-t_i}Z^{(2)}_i,$$

where $i=\{L,S,B\}$ corresponds to a given estimator. We assume that sample sizes in experimental and control treatment groups are equal so that $N_E=N_C=N$. We want to calculate the probability:

$$Pr(Z_i \geq z_{1-\alpha}|Z_i^{(1)}) = Pr(\sqrt{t_i}Z_i^{(1)} + \sqrt{1-t_i}Z_i^{(2)}|Z_i^{(1)})$$
$$=Pr\left(Z_i^{(2)} \geq \frac{z_{1-\alpha} - \sqrt{t_i}Z_i^{(1)}}{\sqrt{1-t_i}}\right).$$

Now, if the effect for the second stage data is assumed to be equal to the one from the planning stage (fixed effect), we obtain conditional power:

$$CP_{\theta_D} = 1 - \Phi\left(\frac{z_{1-\alpha} - \sqrt{t_i}Z_i^{(1)}}{\sqrt{1-t_i}} - \frac{P_{L_E} - P_{L_C}}{\sqrt{2\bar{P}_L(1-\bar{P}_L)\frac{2}{n_{i_2}}}}\right),$$

where n_{i_2} corresponds to the second stage sample size for a given estimator such that $n_{i_2} = n_{i_{C_2}} = n_{i_{E_2}}$.

For the fixed effect we assume the effect, $P_{L_E} - P_{L_C} = \Delta$, from the planning stage, which is equal to

$$\Delta = \frac{(z_{1-\alpha} + z_{1-\beta})\sqrt{2\bar{P}_L(1-\bar{P}_L)}}{\sqrt{N}}$$

which comes from the sample size formula for 2 proportions. This can be then substituted into the conditional power equation:

$$\begin{aligned}
CP_{\theta_D} =& 1 - \Phi\left(\frac{z_{1-\alpha} - \sqrt{t_i}Z_i^{(1)}}{\sqrt{1-t_i}} - \frac{P_{L_E} - P_{L_C}}{\sqrt{\bar{P}_L(1-\bar{P}_L))\frac{2}{n_{i_2}}}}\right) \\
=& 1 - \Phi\left(\frac{z_{1-\alpha} - \sqrt{t_i}Z_i^{(1)}}{\sqrt{1-t_i}} - \frac{(z_{1-\alpha} + z_{1-\beta})\sqrt{2\bar{P}_L(1-\bar{P}_L)}}{\sqrt{N}} \times \frac{1}{\sqrt{2\bar{P}_L(1-\bar{P}_L)\frac{2}{n_{i_2}}}}\right) \\
=& 1 - \Phi\left(\frac{z_{1-\alpha} - \sqrt{t_i}Z_i^{(1)}}{\sqrt{1-t_i}} - \frac{(z_{1-\alpha} + z_{1-\beta})\sqrt{n_{i_2}}}{\sqrt{N}}\right).
\end{aligned}$$

We assume for all estimators that $n_{i_2}/N = 1 - t_i$. We therefore obtain:

$$CP_{\theta_D} = 1 - \Phi\left(\frac{z_{1-\alpha} - \sqrt{t_i}Z_i^{(1)}}{\sqrt{1-t_i}} - (z_{1-\alpha} + z_{1-\beta})\sqrt{1-t_i}\right).$$

For the observed effect instead of plugging in Δ from the planning stage, we plug in the effect from a Z-statistic obtained at interim. At first let us consider the case for $\hat{P}_{\mathbf{L}}^{(1)}$ and $\hat{P}_{\mathbf{S}}^{(1)}$ (assuming equal sample sizes in both treatment groups so that $n_{E_k} = n_{C_k} = n_k$:

$$Z_k^{(1)} = \frac{\hat{P}_{k_E}^{(1)} - \hat{P}_{k_C}^{(1)}}{\sqrt{\bar{P}_k^{(1)}(1-\bar{P}_k^{(1)})\frac{2}{n_k}}},$$

where $k = \{L, S\}$ so that

$$\hat{P}_{k_E}^{(1)} - \hat{P}_{k_C}^{(1)} = Z_k^{(1)}\sqrt{\bar{P}_k^{(1)}(1-\bar{P}_k^{(1)})\frac{2}{n_k}},$$

where n_k ($k = \{L, S\}$ is the first stage sample size and can be equal to n_L or n_S. This can be again substituted into the conditional power equation:

$$\begin{aligned}
CP_{\hat{\theta}} =& 1 - \Phi\left(\frac{z_{1-\alpha} - \sqrt{t_k}Z_k^{(1)}}{\sqrt{1-t_k}} - \frac{\hat{P}_{k_E}^{(1)} - \hat{P}_{k_C}^{(1)}}{\sqrt{\bar{P}_k^{(1)}(1-\bar{P}_k^{(1)})\frac{2}{n_{k_2}}}}\right) \\
=& 1 - \Phi\left(\frac{z_{1-\alpha} - \sqrt{t_k}Z_k^{(1)}}{\sqrt{1-t_i}} - \frac{Z_k^{(1)}\sqrt{2\bar{P}_k^{(1)}(1-\bar{P}_k^{(1)})}}{\sqrt{n_k}} \times \frac{\sqrt{n_{k_2}}}{\sqrt{2\bar{P}_k^{(1)}(1-\bar{P}_k^{(1)})}}\right).
\end{aligned}$$

As $n_{k_2} = (1-t_k)N$ and $n_k = t_kN$, we can substitute the values into the conditional power equation:

$$\begin{aligned} CP_{\hat{\theta}} =& 1 - \Phi\left(\frac{z_{1-\alpha} - \sqrt{t_k}Z_k^{(1)}}{\sqrt{1-t_k}} - \frac{Z_k^{(1)}\sqrt{n_{k_2}}}{\sqrt{n_k}}\right) \\ =& 1 - \Phi\left(\frac{z_{1-\alpha} - \sqrt{t_k}Z_k^{(1)}}{\sqrt{1-t_k}} - \frac{Z_k^{(1)}\sqrt{(1-t_k)}}{\sqrt{t_k}}\right) \\ =& 1 - \Phi\left(\frac{z_{1-\alpha} - Z_k^{(1)}/\sqrt{t_k}}{\sqrt{1-t_k}}\right). \end{aligned}$$

Similarly, we have for $\hat{P}_{\mathbf{B}}^{(1)}$. The Z-statistic for the estimator is equal to (again assuming equal sample sizes in experimental and treatment groups):

$$\begin{aligned} Z_B^{(1)} &= \frac{\hat{P}_{B_E}^{(1)} - \hat{P}_{B_C}^{(1)}}{\sqrt{\bar{P}_B^{(1)}(1-\bar{P}_B^{(1)})\left(\frac{1-\hat{\phi_E}^2\times\left(1-\frac{n_L}{n_S}\right)}{n_L} + \frac{1-\hat{\phi_C}^2\times\left(1-\frac{n_L}{n_S}\right)}{n_L}\right)}} \\ &= \frac{\hat{P}_{B_E}^{(1)} - \hat{P}_{B_C}^{(1)}}{\sqrt{\bar{P}_B^{(1)}(1-\bar{P}_B^{(1)})\left(\frac{2-(\hat{\phi_E}^2+\hat{\phi_C}^2)\times\left(1-\frac{n_L}{n_S}\right)}{n_L}\right)}}. \end{aligned}$$

We know that the information fraction for $\hat{P}_{\mathbf{B}}^{(1)}$ is:

$$t_B = \frac{2n_L}{N\left(2 - \left(1-\frac{n_L}{n_S}\right)(\hat{\phi}_E^2 + \hat{\phi}_C^2)\right)},$$

so we can rewrite the equation for the Z-statistic to be:

$$Z_B^{(1)} = \frac{\hat{P}_{B_E}^{(1)} - \hat{P}_{B_C}^{(1)}}{\sqrt{\bar{P}_B^{(1)}(1-\bar{P}_B^{(1)})\frac{2}{t_BN}}}.$$

This can be then plugged into the conditional power equation:

$$\begin{aligned} CP_{\hat{\theta}} =& 1 - \Phi\left(\frac{z_{1-\alpha} - \sqrt{t_B}Z_B^{(1)}}{\sqrt{1-t_B}} - \frac{Z_B^{(1)}\sqrt{2\bar{P}_B^{(1)}(1-\bar{P}_B^{(1)})}}{\sqrt{t_BN}} \times \frac{\sqrt{n_{B_2}}}{\sqrt{2\bar{P}_B^{(1)}(1-\bar{P}_B^{(1)})}}\right) \\ =& 1 - \Phi\left(\frac{z_{1-\alpha} - \sqrt{t_B}Z_B^{(1)}}{\sqrt{1-t_B}} - \frac{Z_B^{(1)}\sqrt{(1-t_B)N}}{\sqrt{t_BN}}\right) \\ =& 1 - \Phi\left(\frac{z_{1-\alpha} - \sqrt{t_B}Z_B^{(1)}}{\sqrt{1-t_B}} - \frac{Z_B^{(1)}\sqrt{(1-t_B)}}{\sqrt{t_B}}\right) \\ =& 1 - \Phi\left(\frac{z_{1-\alpha} - Z_B^{(1)}/\sqrt{t_B}}{\sqrt{1-t_B}}\right), \end{aligned}$$

as in the above example for $\hat{P}_{\mathbf{S}}^{(1)}$ and $\hat{P}_{\mathbf{L}}^{(1)}$.

1.6 Cut-off Point Equivalence

The equivalent cut off-points can be found by equalising the Z-statistics and solving for a chosen c. Let $Z^{(1)}$ be the Z-statistic calculated at interim, $\theta_D = (z_{1-\alpha} + z_{1-\beta})$ and $z_{1-\alpha}$ is the $(1-\alpha)$ quantile of the standard normal distribution.

We have the following for formula rearrangement for the fixed effect:

$$
\begin{aligned}
c_{\theta_D} &= 1-\Phi\left(\frac{z_{1-\alpha}-Z^{(1)}\times\sqrt{t}-(z_{1-\alpha}+z_{1-\beta})(1-t)}{\sqrt{1-t}}\right)\\
\iff\quad \Phi^{-1}(1-c_{\theta_D}) &= \frac{z_{1-\alpha}-Z^{(1)}\times\sqrt{t}-(z_{1-\alpha}+z_{1-\beta})(1-t)}{\sqrt{1-t}}\\
\iff\quad \Phi^{-1}(1-c_{\theta_D})\sqrt{1-t} &= z_{1-\alpha}-Z^{(1)}\times\sqrt{t}-(z_{1-\alpha}+z_{1-\beta})(1-t)\\
\iff\quad Z^{(1)}\times\sqrt{t} &= z_{1-\alpha}-(z_{1-\alpha}+z_{1-\beta})(1-t)-\Phi^{-1}(1-c_{\theta_D})\sqrt{1-t}\\
\iff\quad Z^{(1)} &= \frac{z_{1-\alpha}-(z_{1-\alpha}+z_{1-\beta})(1-t)-\Phi^{-1}(1-c_{\theta_D})\sqrt{1-t}}{\sqrt{t}}.
\end{aligned}
$$

Similarly, for the observed effect power approach, the Z-statistic can be obtained:

$$
\begin{aligned}
c_{\hat{\theta}} &= 1-\Phi\left(\frac{z_{1-\alpha}-Z^{(1)}/\sqrt{t}}{\sqrt{1-t}}\right)\\
\iff\quad \Phi^{-1}(1-c_{\hat{\theta}}) &= \frac{z_{1-\alpha}-Z^{(1)}/\sqrt{t}}{\sqrt{1-t}}\\
\iff\quad \Phi^{-1}(1-c_{\hat{\theta}})\sqrt{1-t} &= z_{1-\alpha}-Z^{(1)}/\sqrt{t}\\
\iff\quad Z^{(1)}/\sqrt{t} &= z_{1-\alpha}-\Phi^{-1}(1-c_{\hat{\theta}})\sqrt{1-t}\\
\iff\quad Z^{(1)} &= \left(z_{1-\alpha}-\Phi^{-1}(1-c_{\hat{\theta}})\sqrt{1-t}\right)\sqrt{t}.
\end{aligned}
$$

Now by equalising for $Z^{(1)}$, we can find an equivalent cut-off point for alternative hypothesis conditional power given the fixed effect:

$$
\begin{aligned}
\left(z_{1-\alpha}-\Phi^{-1}(1-c_{\hat{\theta}})\sqrt{1-t}\right)\sqrt{t} &= \frac{z_{1-\alpha}-\Phi^{-1}(1-c_{\theta_D})\sqrt{1-t}-(z_{1-\alpha}+z_{1-\beta})(1-t)}{\sqrt{t}}\\
\iff\quad \left(z_{1-\alpha}-\Phi^{-1}(1-c_{\hat{\theta}})\sqrt{1-t}\right)t &= z_{1-\alpha}-\Phi^{-1}(1-c_{\theta_D})\sqrt{1-t}-(z_{1-\alpha}+z_{1-\beta})(1-t)\\
\iff\quad \Phi^{-1}(1-c_{\theta_D})\sqrt{1-t} &= z_{1-\alpha}-(z_{1-\alpha}+z_{1-\beta})(1-t)-\left(z_{1-\alpha}-\Phi^{-1}(1-c_{\hat{\theta}})\sqrt{1-t}\right)t\\
\iff\quad 1-c_{\theta_D} &= \Phi\left(\frac{z_{1-\alpha}-(z_{1-\alpha}+z_{1-\beta})(1-t)-\left(z_{1-\alpha}-\Phi^{-1}(1-c_{\hat{\theta}})\sqrt{1-t}\right)t}{\sqrt{1-t}}\right)\\
\iff\quad c_{\theta_D} &= 1-\Phi\left(\frac{z_{1-\alpha}-(z_{1-\alpha}+z_{1-\beta})(1-t)-\left(z_{1-\alpha}-\Phi^{-1}(1-c_{\hat{\theta}})\sqrt{1-t}\right)t}{\sqrt{1-t}}\right),
\end{aligned}
$$

and similarly for observed effect given the fixed effect approach:

$$
\begin{aligned}
\left(z_{1-\alpha}-\Phi^{-1}(1-c_{\hat{\theta}})\sqrt{1-t}\right)\sqrt{t} &= \frac{z_{1-\alpha}-\Phi^{-1}(1-c_{\theta_D})\sqrt{1-t}-(z_{1-\alpha}+z_{1-\beta})(1-t)}{\sqrt{t}}\\
\iff\quad \left(z_{1-\alpha}-\Phi^{-1}(1-c_{\hat{\theta}})\sqrt{1-t}\right) &= \frac{z_{1-\alpha}-\Phi^{-1}(1-c_{\theta_D})\sqrt{1-t}-(z_{1-\alpha}+z_{1-\beta})(1-t)}{t}\\
\iff\quad \Phi^{-1}(1-c_{\hat{\theta}})\sqrt{1-t} &= z_{1-\alpha}-\frac{z_{1-\alpha}-\Phi^{-1}(1-c_{\theta_D})\sqrt{1-t}-(z_{1-\alpha}+z_{1-\beta})(1-t)}{t}\\
\iff\quad \Phi^{-1}(1-c_{\hat{\theta}}) &= \frac{z_{1-\alpha}}{\sqrt{1-t}}-\frac{z_{1-\alpha}-\Phi^{-1}(1-c_{\theta_D})\sqrt{1-t}-(z_{1-\alpha}+z_{1-\beta})(1-t)}{t\sqrt{1-t}}\\
\iff\quad c_{\hat{\theta}} &= 1-\Phi\left(\frac{z_{1-\alpha}}{\sqrt{1-t}}-\frac{z_{1-\alpha}-\Phi^{-1}(1-c_{\theta_D})\sqrt{1-t}-(z_{1-\alpha}+z_{1-\beta})(1-t)}{t\sqrt{1-t}}\right).
\end{aligned}
$$

1.7 Second Stage Sample Size Derivation

For sample size reassessment formula, we use similar methodology as for obtaining conditional power. Here, however, the assumption on the second stage data will be written in terms of the second stage sample size. As the combination test assumes independence of data from the two stages, the first stage sample size for $\hat{P}_{\mathbf{B}}^{(1)}$ will be assumed to be equal to n_S. What is more, the final Z-statistic is constructed using pre-specified weights of the combination test, so that:

$$Z=\sqrt{w}Z^{(1)}+\sqrt{1-w}Z^{(2)}.$$

In such a case the weights can be chosen arbitrarily as long as $0 \leq w \leq 1$. Let n_2 correspond to second-stage sample size. For the fixed effect we have:

$$CP_{\theta_D} = 1 - \Phi\left(\frac{z_{1-\alpha} - \sqrt{w}Z^{(1)}}{\sqrt{1-w}} - \frac{(z_{1-\alpha} + z_{1-\beta})\sqrt{n_2}}{\sqrt{N}}\right).$$

Now, rewrite the second stage sample size to be equal to $\tilde{N} - n_k$, which corresponds to the adapted total sample size and the first stage sample size and $k = \{S, L\}$. The conditional power equations will be set to be equal to $1-\beta$, i.e. the power from the planning stage, and then rearranged in order to be solved for the second stage sample size, $\tilde{N} - n_k$:

$$\begin{aligned}
1-\beta &= 1 - \Phi\left(\frac{z_{1-\alpha} - \sqrt{w}Z^{(1)}}{\sqrt{1-w}} - \frac{(z_{1-\alpha} + z_{1-\beta})\sqrt{\tilde{N} - n_k}}{\sqrt{N}}\right) \\
\iff \quad \Phi^{-1}(\beta) &= \frac{z_{1-\alpha} - \sqrt{w}Z^{(1)}}{\sqrt{1-w}} - \frac{(z_{1-\alpha} + z_{1-\beta})}{\sqrt{N}}\sqrt{\tilde{N} - n_k} \\
\iff \quad \sqrt{\tilde{N} - n_k} &= \frac{\frac{z_{1-\alpha} - \sqrt{w}Z^{(1)}}{\sqrt{1-w}} - \Phi^{-1}(\beta)}{(z_{1-\alpha} + z_{1-\beta})/\sqrt{N}} \\
\iff \quad \tilde{N} - n_k &= \left(\frac{\frac{z_{1-\alpha} - \sqrt{w}Z^{(1)}}{\sqrt{1-w}} - \Phi^{-1}(\beta)}{(z_{1-\alpha} + z_{1-\beta})/\sqrt{N}}\right)^2
\end{aligned}$$

For the observed effect we follow the same steps. Similarly, let $\tilde{N} - n_k$ denote the adapted second stage sample size:

$$CP_{\hat{\theta}} = 1 - \Phi\left(\frac{z_{1-\alpha} - \sqrt{w}Z^{(1)}}{\sqrt{1-w}} - \frac{Z^{(1)}\sqrt{\tilde{N} - n_k}}{\sqrt{tN}}\right),$$

Now, again rearranging for $\tilde{n}_2$ we obtain:

$$\begin{aligned}
1-\beta &= 1 - \Phi\left(\frac{z_{1-\alpha} - \sqrt{w}Z^{(1)}}{\sqrt{1-w}} - \frac{Z^{(1)}\sqrt{\tilde{N} - n_k}}{\sqrt{tN}}\right) \\
\Phi^{-1}(\beta) &= \frac{z_{1-\alpha} - \sqrt{w}Z^{(1)}}{\sqrt{1-w}} - \frac{Z^{(1)}}{\sqrt{tN}}\sqrt{\tilde{N} - n_k} \\
\sqrt{\tilde{N} - n_k} &= \frac{\frac{z_{1-\alpha} - \sqrt{w}Z^{(1)}}{\sqrt{1-w}} - \Phi^{-1}(\beta)}{Z^{(1)}/\sqrt{tN}} \\
\tilde{N} - n_k &= \left(\frac{\frac{z_{1-\alpha} - \sqrt{w}Z^{(1)}}{\sqrt{1-w}} - \Phi^{-1}(\beta)}{Z^{(1)}/\sqrt{tN}}\right)^2.
\end{aligned}$$

Note that for each approach, t corresponds to the information fraction of a given estimator. $Z^{(1)}$ corresponds to the Z-statistic calculated at interim and can correspond to a Z-statistic for any estimator. t and $Z^{(1)}$ should however correspond to the information fraction and a Z-statistic from use of the same approach.

2 Conditional Probabilities For Interim Analyses with Futility Stopping Only

In this Section we report the conditional probabilities given the interim decision in the trial. These include the probability to reject the null hypothesis at the end of the trial given it was continued, the probability that we would have failed to reject the null hypothesis for the trials that were stopped for futility, and the probability of making the correct decision, i.e. rejecting the trial given it was continued and failed to reject the trial would have been continued (for the trials that were stopped for futility).

In the first subsection we present results from the case presented in the main body of the article with the following operating characteristics of the trial: one-sided $\alpha = 0.025$, $1-\beta = 0.8$, $t_L = 0.25$, $t_S = 0.5$, $N = 200$, $P_{L_C} = P_{S_C} = 0.2$, $P_{L_E} \approx 0.323$, $\phi_E = \phi_C = 0.5$. We considered four types of outcomes for the short-term outcome in the experimental treatment group:

no effect, moderate effect, effect equal to long-term outcome, and larger effect than for the long-term outcome with the following probabilities: $(0.2, 0.285, 0.323, 0.365)$.

In the next subsection results for $\phi_E = \phi_C = 0.2$ and $\phi_E = \phi_C = 0.65$ are presented. The figures show the power, probability to stop for futility, the probability of rejecting the null hypothesis at the end of the trial given it was continued, the probability that we would have failed to reject the null hypothesis for the trials that were stopped for futility probability of making the correct decision.

For the remainder of the supplementary materials, the code for reproducing the output using package `binfutssr` will be also provided. The package consists of a set of functions which calculate the operating characteristics of simulated clinical trials.

In order to create the first plot, load the package `binfutssr` (with its dependencies) and type in the following code. One scenario of 100,000 simulations takes approximately 2 min for interim analysis with futility stopping and 2.5 min for sample size reassessments. If one has access to multiple cores, the packages `parallel` and `doParallel` can be loaded and the number of cores can be specified using function `cl <- makeCluster(m)` with m coresponding to the number of cores. The cores can be registered using `registerDoParallel(cl)`. The code for provision of graphics uses the `foreach` function which if more than 1 core is specified parallises the procedure.

At first, fix the setting for trial simulation

```
library(psych)
library(ggplot2)
```

```
##
## Attaching package: 'ggplot2'
```

```
## The following objects are masked from 'package:psych':
##
##     %+%, alpha
```

```
library(foreach)
library(mvtnorm)
library(stringr)
library(gridExtra)
library(parallel)
library(doParallel)
```

```
## Loading required package: iterators
```

```
library(xtable)
library(knitr)
library(kableExtra)
library(binfutssr) # need to install this locally from .tar.gz file
```

```
## Loading required package: grid
```

2.1 Varying Correlation between S and L, $\phi_E = \phi_C = 0.2$

```
p_se <- c(0.2, 0.285, 0.3227348, 0.365)

m <- length(p_se) # if multiple cores are available set this value to length of p_se
cl <- parallel::makeCluster(m)
doParallel::registerDoParallel(cl)

output_cp_alt <- foreach(i = 1:length(p_se), .packages="binfutssr") %dopar%
  (cp(nsim = 100000,        #number of simulations
      alpha = 0.025,         # one-sided alpha
      beta = 0.2,            # type 2 error such that 1-beta is the power
      p_le = 0.3227348,      # probability of success for long-term endpoint in E
      p_lc = 0.2,            # probability of success for long-term endpoint in C
      p_se = p_se[i],        # i-th probability of success for short-term endpoint in E
```

```
      p_sc = 0.2,             # probability of success for short-term endpoint in C
      n = 200,                # sample size per treatment arm
      fr_lo = 0.25,           # amounut of long-term information available at interim
      fr_sh = 0.5,            # amounut of short-term information available at interim
      phi_e = 0.2,            # correlation in E
      phi_c = 0.2,            # correlation in C
      c = seq(0, 1, 0.01)))  #sequence of cut-off points for stopping based on cp


#Simulate clinical trial for moderate power (~50%)



output_cp_mod <- foreach(i = 1:length(p_se), .packages="binfutssr") %dopar%
  (cp(nsim = 100000,
      alpha = 0.025,
      beta = 0.2,
      p_le = 0.285,
      p_lc = 0.2,
      p_se = p_se[i],
      p_sc = 0.2,
      n = 200,
      fr_lo = 0.25,
      fr_sh = 0.5,
      phi_e = 0.2,
      phi_c = 0.2,
      c = seq(0, 1, 0.01)))


#Simulate clinical trial under the null hypothesis


output_cp_null <- foreach(i = 1:length(p_se), .packages="binfutssr") %dopar%
  (cp(nsim = 100000,
      alpha = 0.025,
      beta = 0.2,
      p_le = 0.2,
      p_lc = 0.2,
      p_se = p_se[i],
      p_sc = 0.2,
      n = 200,
      fr_lo = 0.25,
      fr_sh = 0.5,
      phi_e = 0.2,
      phi_c = 0.2,
      c = seq(0, 1, 0.01)))
```

2.1.1 Overall Power

```
power_plots_phi_02 <- create_power_plots(output_cp_alt = output_cp_alt,
                                         output_cp_mod = output_cp_mod,
                                         output_cp_null = output_cp_null,
                                         phi_e = 0.2,
                                         phi_c = 0.2,
                                         filetype = "none")
```

```r
grid.arrange(power_plots_phi_02)
```

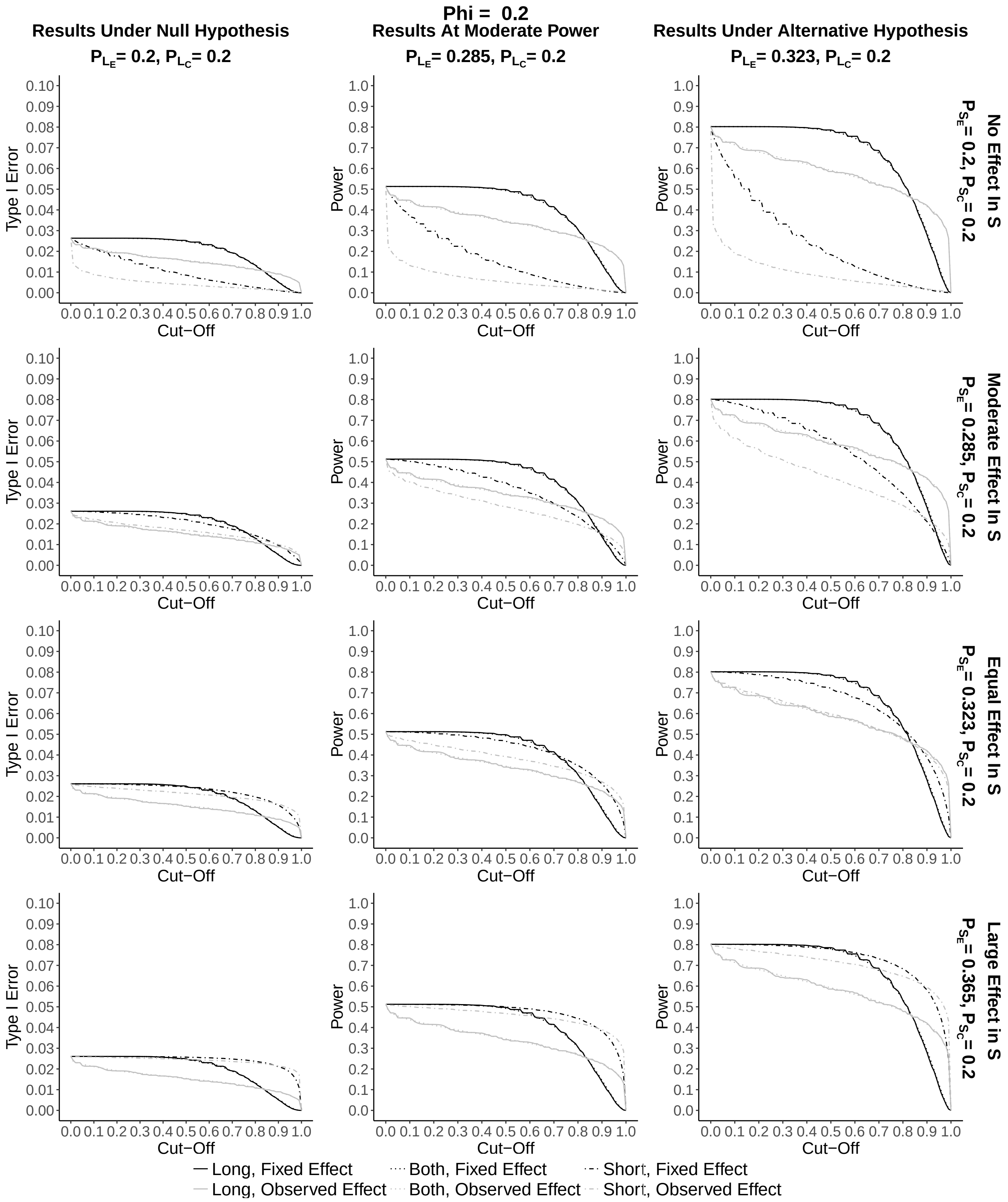

Figure 1: Plots showing the power plotted against cut-off points for different effect sizes in P_{S_E} for $\phi_E = \phi_C = 0.2$. First column corresponds to the results under the null hypothesis, middle column to the simulations at moderate power and right to the simulations under alternative hypothesis. The rows correspond to no effect, moderate effect, effect equal to the one P_{L_E} under the alternative hypothesis, and a higher effect than for P_{L_E} respectively. Grey lines correspond to observed effect conditional power, $CP_{\hat{\theta}}$, whereas black to fixed effect conditional power, CP_{θ_D}. $\hat{P}_{\mathbf{B}}^{(1)}$ is denoted by dotted lines, $\hat{P}_{\mathbf{L}}^{(1)}$ by solid and $\hat{P}_{\mathbf{S}}^{(1)}$ by dot-dashed. Note that the scale in the first column is from 0 to 0.1.

2.1.2 Probability to Stop for Futility

```
fs_plots_phi_02 <- create_fs_plots(output_cp_alt = output_cp_alt,
                                   output_cp_mod = output_cp_mod,
                                   output_cp_null = output_cp_null,
                                   phi_e = 0.2,
                                   phi_c = 0.2,
                                   filetype = "none")
```

```
grid.arrange(fs_plots_phi_02)
```

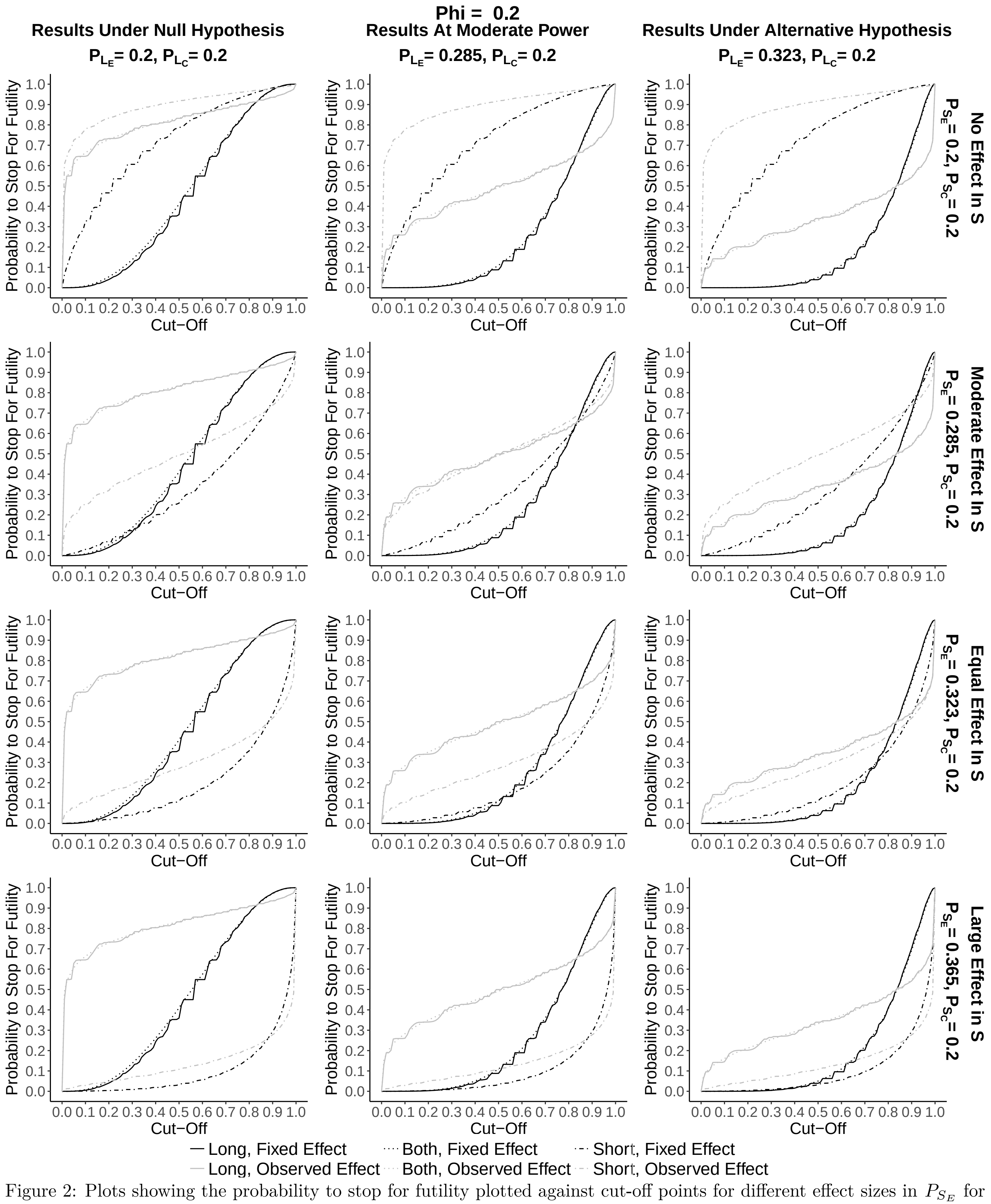

Figure 2: Plots showing the probability to stop for futility plotted against cut-off points for different effect sizes in P_{S_E} for $\phi_E = \phi_C = 0.2$. First column corresponds to the results under the null hypothesis, middle column to the simulations at moderate power and right to the simulations under alternative hypothesis. The rows correspond to no effect, moderate effect, effect equal to the one P_{L_E} under the alternative hypothesis, and a higher effect than for P_{L_E} respectively. Grey lines correspond to observed effect conditional power, $CP_{\hat{\theta}}$, whereas black to fixed effect conditional power, CP_{θ_D}. $\hat{P}^{(1)}_{\mathbf{B}}$ is denoted by dotted lines, $\hat{P}^{(1)}_{\mathbf{L}}$ by solid and $\hat{P}^{(1)}_{\mathbf{S}}$ by dot-dashed. Note that the scale in the first column is from 0 to 0.1.

2.1.3 Probability to Reject the Null Hypothesis Given Trial Was Continued

```
prob_cont_rej_plots_phi_02 <- create_prob_cont_rej_plots(
  output_cp_alt = output_cp_alt,
  output_cp_mod = output_cp_mod,
  output_cp_null = output_cp_null,
  phi_e = 0.2,
  phi_c = 0.2,
  filetype = "none")
```

```
grid.arrange(prob_cont_rej_plots_phi_02)
```

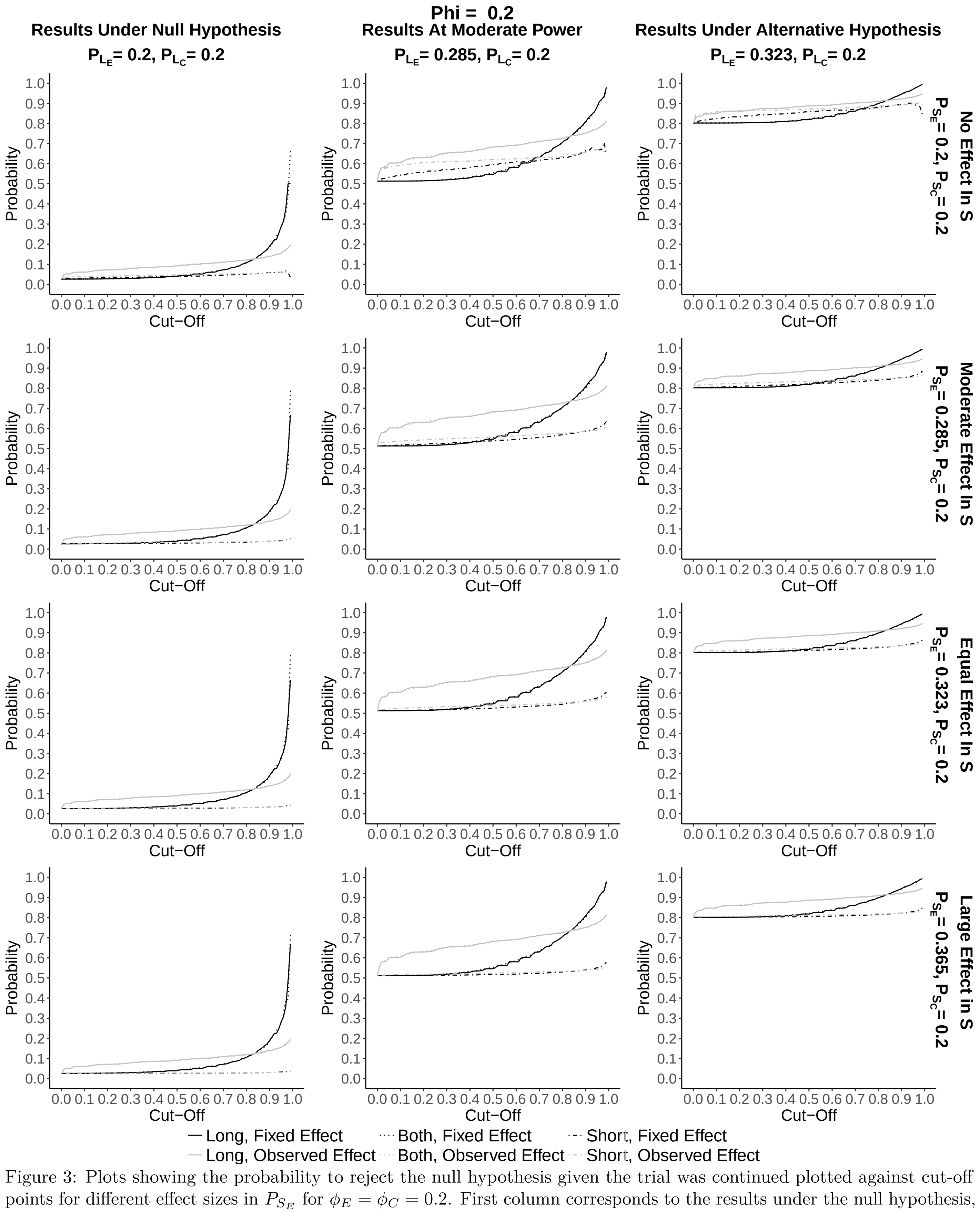

Figure 3: Plots showing the probability to reject the null hypothesis given the trial was continued plotted against cut-off points for different effect sizes in P_{S_E} for $\phi_E = \phi_C = 0.2$. First column corresponds to the results under the null hypothesis, middle column to the simulations at moderate power and right to the simulations under alternative hypothesis. The rows correspond to no effect, moderate effect, effect equal to the one P_{L_E} under the alternative hypothesis, and a higher effect than for P_{L_E} respectively. Grey lines correspond to observed effect conditional power, $CP_{\hat{\theta}}$, whereas black to fixed effect conditional power, CP_{θ_D}. $\hat{P}^{(1)}_{\mathbf{B}}$ is denoted by dotted lines, $\hat{P}^{(1)}_{\mathbf{L}}$ by solid and $\hat{P}^{(1)}_{\mathbf{S}}$ by dot-dashed. Note that the scale in the first column is from 0 to 0.1.

2.1.4 Probability to Fail to Reject the Null Hypothesis Given The Trial Was Stopped Had it Been Continued

```
prob_stop_not_rej_plots_phi_02 <- create_prob_stop_not_rej_plots(
  output_cp_alt = output_cp_alt,
  output_cp_mod = output_cp_mod,
  output_cp_null = output_cp_null,
  phi_e = 0.2,
  phi_c = 0.2,
  filetype = "none")
```

```
grid.arrange(prob_stop_not_rej_plots_phi_02)
```

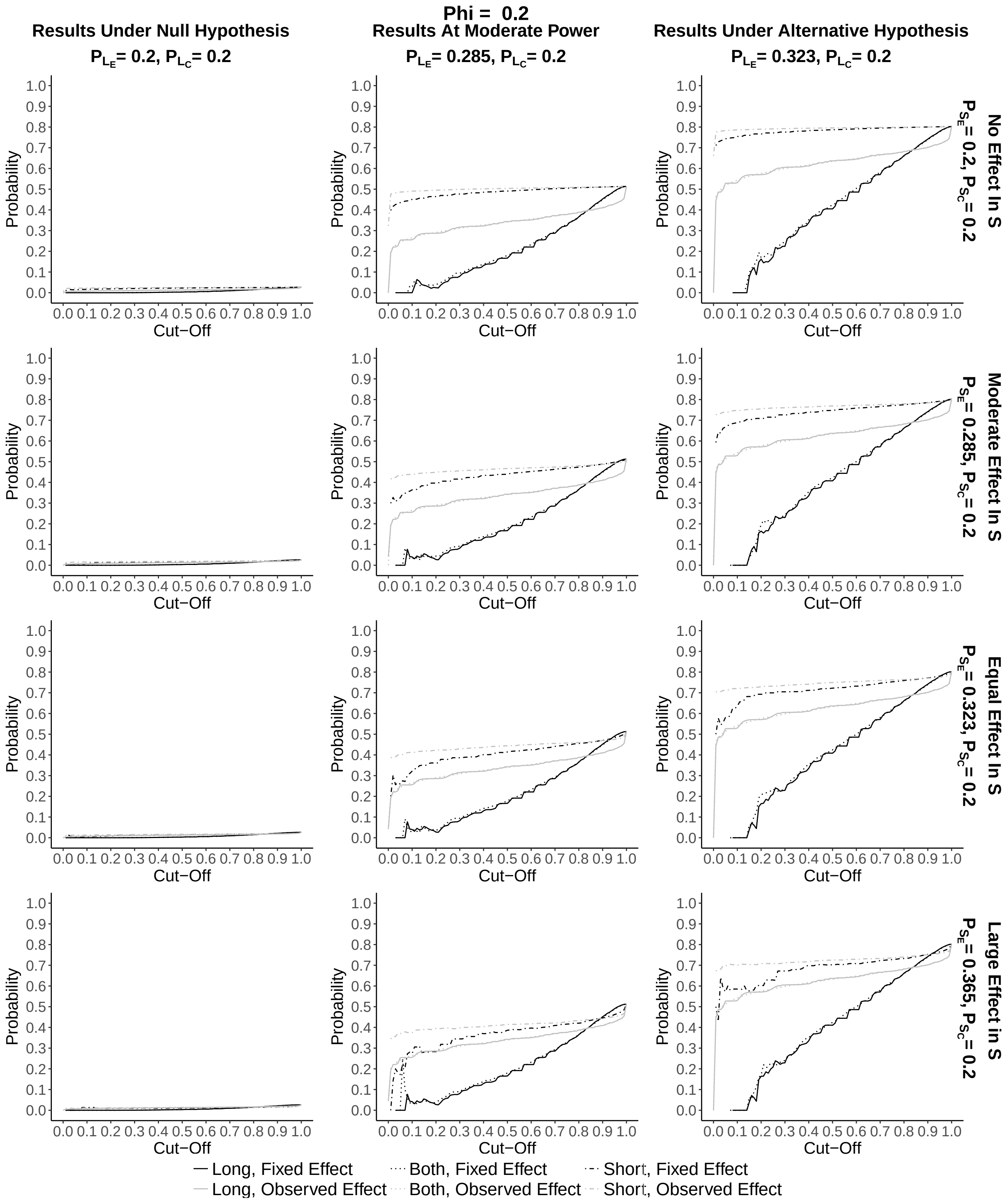

Figure 4: Plots showing the probability of not having rejected the null hypothesis, if the trial had been continued for the cases where the interim decision was to stop the trial for futility. The values were plotted against cut-off points for different effect sizes in P_{S_E} for $\phi_E = \phi_C = 0.2$. First column corresponds to the results under the null hypothesis, middle column to the simulations at moderate power and right to the simulations under alternative hypothesis. The rows correspond to no effect, moderate effect, effect equal to the one P_{L_E} under the alternative hypothesis, and a higher effect than for P_{L_E} respectively. Grey lines correspond to observed effect conditional power, $CP_{\hat{\theta}}$, whereas black to fixed effect conditional power, CP_{θ_D}. $\hat{P}_{\mathbf{B}}^{(1)}$ is denoted by dotted lines, $\hat{P}_{\mathbf{L}}^{(1)}$ by solid and $\hat{P}_{\mathbf{S}}^{(1)}$ by dot-dashed. Note that the scale in the first column is from 0 to 0.1.

2.1.5 Probability to Make the Correct Decision

```
prob_corr_dec_plots_phi_02 <- create_prob_corr_dec_plots(
  output_cp_alt = output_cp_alt,
  output_cp_mod = output_cp_mod,
  output_cp_null = output_cp_null,
  phi_e = 0.2,
  phi_c = 0.2,
  filetype = "none")
```

```
grid.arrange(prob_corr_dec_plots_phi_02)
```

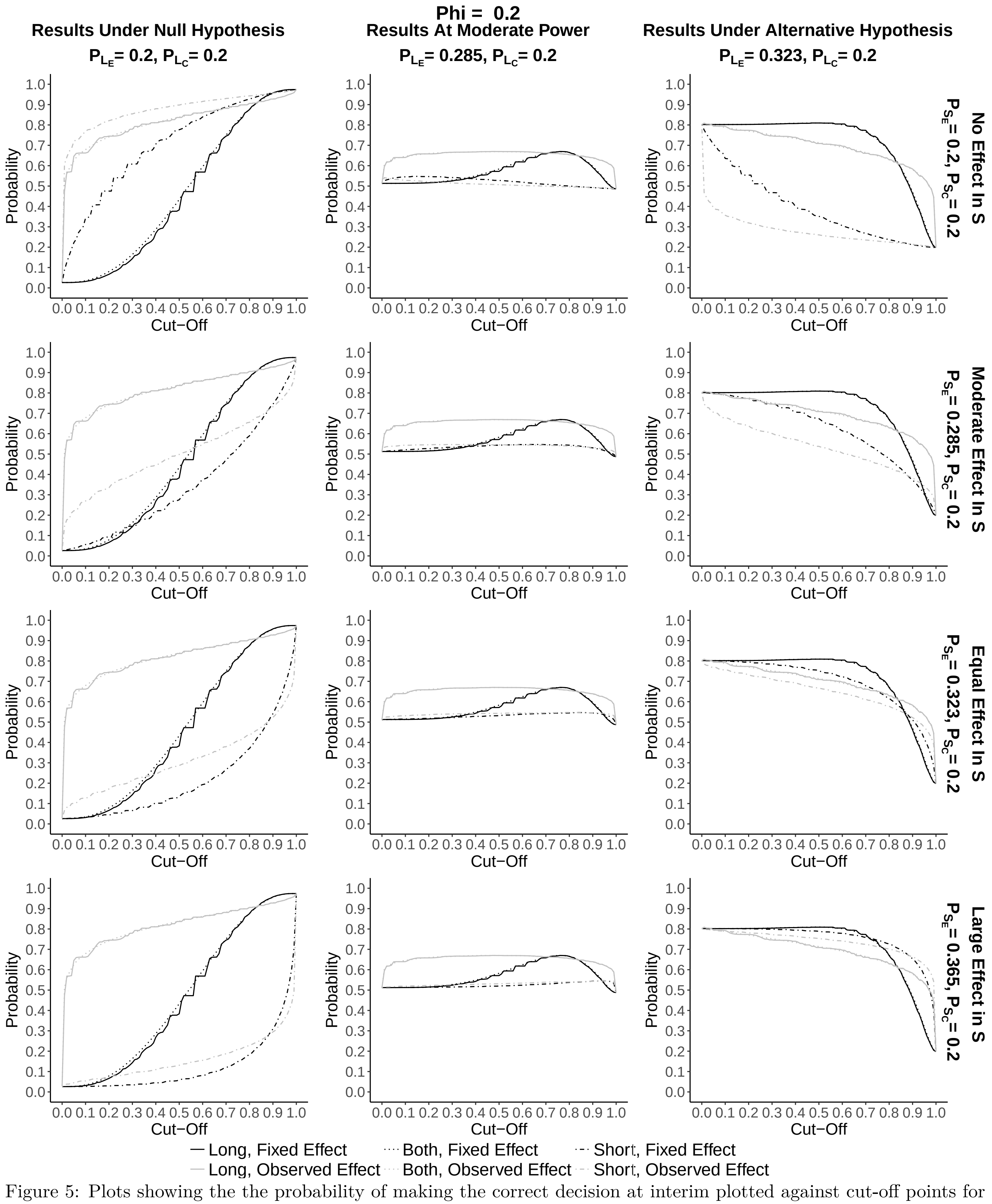

Figure 5: Plots showing the the probability of making the correct decision at interim plotted against cut-off points for different effect sizes in P_{S_E} for $\phi_E = \phi_C = 0.2$. First column corresponds to the results under the null hypothesis, middle column to the simulations at moderate power and right to the simulations under alternative hypothesis. The rows correspond to no effect, moderate effect, effect equal to the one P_{L_E} under the alternative hypothesis, and a higher effect than for P_{L_E} respectively. Grey lines correspond to observed effect conditional power, $CP_{\hat{\theta}}$, whereas black to fixed effect conditional power, CP_{θ_D}. $\hat{P}_{\mathbf{B}}^{(1)}$ is denoted by dotted lines, $\hat{P}_{\mathbf{L}}^{(1)}$ by solid and $\hat{P}_{\mathbf{S}}^{(1)}$ by dot-dashed. Note that the scale in the first column is from 0 to 0.1.
